# Supplementary material for: Revision of the Late Jurassic deep-water teleosauroid crocodylomorph Teleosaurus megarhinus Hulke, 1871 and evidence of pelagic adaptations in Teleosauroidea
Source: PeerJ. 2019 Apr 2;7:e6646. doi: 10.7717/peerj.6646 (PMC6450380; doi:10.7717/peerj.6646)
Supplement: Data S1 [file peerj-07-6646-s001.docx]

# ONLINE SUPPLEMENTARY MATERIAL FOR:

Revision of the Late Jurassic deep-water teleosauroid crocodylomorph *Teleosaurus megarhinus* Hulke, 1871 and evidence of pelagic adaptations in Teleosauroidea

Davide Foffa,^1, 2*^ Michela M. Johnson,^2^ Mark T. Young,^2^ Lorna Steel,^3^ and Stephen L. Brusatte^2,1^

^1^ Department of Natural Sciences, National Museums Scotland, Edinburgh, United Kingdom

^2^ School of Geosciences, University of Edinburgh, Edinburgh, United Kingdom

^3^ Department of Earth Sciences, Natural History Museum, London, United Kingdom

^*^Corresponding author: Davide Foffa, [davidefoffa@gmail.com](mailto:davidefoffa@gmail.com); [d.foffa@nms.ac.uk](mailto:d.foffa@nms.ac.uk)

S0) Phylogenetic analysis protocol

S1) CrocSuperMatrix Project Overview

S2) Dataset: Hastings + Young (H+Y)

S2.1) H+Y dataset – general information and scoring sources of the OTUs

S2.2) H+Y dataset – character list

S3) Character and OTUs breakdowns of the merged, and parent, datasets

S4) Supplementary References

S5) List of institutional abbreviations

**S0) Phylogenetic analysis protocol**

The dataset phylogenetic dataset was subjected to a cladistic analysis under maximum parsimony in TNT 1.5 Willi Hennig Society Edition (Goloboff et al. 2008; Goloboff and Catalano 2016), following the methodology used in Young et al. (2016). Memory settings were increased with General RAM set to 900 Mb and the maximum number of trees to be held set to 99,999. Tree space was searched first using the ‘New Technology search’ option in TNT (Sectorial Search, Ratchet, Drift, and Tree fusing) with 1000 random-addition replicates (RAS). In addition, we increased the default setting for the iterations of each method (except for Tree fusing, which was kept at 3 rounds). In the Sectorial Search we ran 1000 Drift cycles (for selections of above 75) and 1000 starts and fuse trees 1000 times (for selections below 75), as well as 1000 rounds of Consensus Sectorial Searches (CSSs) and Exclusive Sectorial Searches (XSSs). For Ratchet, the program used 1000 ratchet iterations set to stop the perturbation when 1000 substitutions were made or 99% of the swapping was reached. Lastly, in Drift, the analysis included 1000 Drift cycles were set to stop the perturbation when 1000 substitutions were made or 99% of the swapping was reached. A final round of TBR branch swapping was perfomed after the New Technology search. The collapsing rule used was 50%.

**S1) CrocSuperMatrix Project Overview**

Thus far two datasets have been successfully merged, those of Alexander Hastings and Mark Young (see Ristevski *et al*., 2018). This has formed the Hastings and Young dataset (referred to herein as the H+Y matrix). Currently, four datasets are in the process of being merged; however herein only one dataset will be used. The modified version of the Andrade *et al*. (2011) dataset (herein referred to as the mA matrix), alongside the first iteration of the H+Y matrix, was first published in Ristevski *et al*. (2018).

The third dataset, is a modification of the dataset published by Wilberg (2017), (currently being worked on). A fourth dataset has also been added, the “basal crocodylomorph” or BC dataset. This is an expansion of the dataset first published by Clark *et al*. (2000), and recently elaborated upon by Pol *et al*. (2013) and Leardi *et al*. (2017). However, using it to look into teleosauroid internal relationships is beyond the scope of this manuscript.

Note that here we have created two new sub-sections: 1) internal neuroanatomy, sensory systems and cranial exocrine glands, and 2) craniomandibular pneumaticity.

The characters for both datasets have been organised into a common anatomical order, and broken down into the same 20 sub-sections:

1. skull geometry and dimensions
2. craniomandibular ornamentation
3. internal neuroanatomy, sensory systems and cranial exocrine glands
4. craniomandibular pneumaticity
5. rostral neurovascular foramina
6. cranial rostrum
7. skull roof
8. orbit and temporal region
9. palate and perichoanal structures
10. occipital
11. braincase, basicranium and suspensorium
12. mandibular geometry
13. mandible
14. dentition and alveolar morphologies
15. axial post-cranial skeleton
16. appendicular skeleton: pectoral girdle and forelimbs
17. appendicular skeleton: pelvic girdle and hind limbs
18. dermal ossifications: osteoderms
19. dermal ossifications: gastralia
20. soft tissue

**S2) Dataset one: Hastings + Young (H+Y)**

***S2.1) H+Y dataset – general information and scoring sources of the OTUs***

The present list includes information for each operational taxonomic unit (OTU) included in the matrix. Fragmentary taxa (i.e. ones that are highly incomplete) are mentioned as: [fragmentary taxon].

**Outgroup taxon**

Rauisuchidae (1 OTU)

(1) *Postosuchus kirkpatricki* Chatterjee, 1985

Data from: Nesbitt (2011), Weinbaum (2011), Weinbaum (2013).

Locality: Post (=Miller) Quarry, Texas, USA.

Formation: Cooper Canyon Formation, Dockum Group.

Age: Norian, Late Triassic.

**Ingroup taxa**

Basal Crocodylomorphs (= ‘sphenosuchians’ *sensu lato*) (5 OTUs)

(2) *Dromicosuchus grallator* Sues *et al*., 2003

Data from: Sues *et al*. (2003), Nesbitt (2011).

Locality: West Genlee, Durham County, North Carolina, USA.

Formation: Mudstone of Lithofacies Association II, Newark Super-Group. South-central region of Durham sub-basin of Deep River Basin.

Age: upper Carnian or lower Norian, Late Triassic.

(3) *Hesperosuchus* cf. *agilis*

Data from: CM 29894; Clark *et al*. (2000), Nesbitt (2011).

Locality: *Coelophysis* Quarry, Ghost Ranch, northern New Mexico, USA.

Formation: “siltstone member”, Chinle Formation.

Age: upper Norian*–*?Rhaetian, Late Triassic.

(4) *Terrestrisuchus gracilis* Crush, 1984

Data from: Crush (1984), Nesbitt (2011).

Locality: Pant-y-ffynon Quarry, Cowbridge, Glamorgan, Wales, UK.

Formation: fissure fills in Carboniferous limestone.

Age: ?Rhaetian, Late Triassic.

(5) *Dibothrosuchus elaphros* Simmons, 1965

Data from: Wu (1986); Nesbitt (2011).

Locality: Huangchiatien, Lufeng, Yunnan, China.

Formation: Zhangjiawa Formation, Lower Lufeng Group.

Age: Sinemurian*–*Pliensbachian, Lower Jurassic.

(6) *Junggarsuchus sloani* Clark *et al*., 2004

Data from: photographs of the holotype provided by Eric Wilberg; Clark *et al*. (2004).

Locality: Wucaiwan, Altay Prefecture, Xinjiang Province, NW China.

Formation: lower part of the Shishugou Formation (= Wucaiwan Formation).

Age: Bathonian*–*Callovian, Middle Jurassic.

Basal crocodyliforms: ‘Protosuchians’ *sensu lato* (4 otUs)

(7) *Hemiprotosuchus leali* Bonaparte, 1971

Data from: Bonaparte (1971).

Locality: Quebrada de los Jachaleros, W La Rioja Province, Argentina.

Formation: Los Colorados Formation.

Age: Coloradense, Norian, Upper Triassic.

(8) *Protosuchus richardsoni* Brown, 1933

Data from: Colbert & Mook (1951), Nesbitt (2011).

Locality: Ward’s Terrace, Arizona, USA.

Formation: upper half of the Moenave Formation, Glen Canyon Group.

Age: Hettangian, Lower Jurassic.

(9) *Protosuchus haughtoni* (Busbey & Gow, 1984)

Data from: Gow (2000), Nesbitt (2011).

Locality: South Africa.

Formation: Upper Elliot Formation.

Age: Lower Jurassic.

(10) *Eopneumatosuchus colberti* Crompton & Smith, 1980

Data from: Crompton & Smith (1980); high-resolution images of the holotype provided by Lawrence Witmer.

Locality: 11 miles NE of Cameron, Coconino County, Arizona, USA.

Formation: ‘Silty facies’, Kayenta Formation, Glen Canyon Group.

Age: Sinemurian-Pliensbachian, Lower Jurassic.

Basal crocodyliforms: Shartegosuchidae (1 OTU)

(11) *Fruitachampsa callisoni* Clark, 2011

Data from: Clark (2011).

Locality: Fruita, Colorado, USA.

Formation: Morrison Formation.

Age: Upper Jurassic.

Notosuchia: ‘Notosuchidae’ (2 OTUs)

(12) *Notosuchus terrestris* Woodward, 1896

Data from: MACN-Pv-N-22, MACN-Pv-N-23, MACN-Pv-N-24, MACN-Pv-N-43, MACN-Pv-N-107, MACN-Pv-RN-1015, MACNPv-RN-1037, MACN-Pv-RN-1038, MACN-Pv-RN-1039, MACN-Pv-RN-1040, MACN-Pv-RN-1041, MACN-Pv-RN-1043, MACN-Pv-RN-1044, MACN-Pv-RN-1045, MACN-Pv-RN-1046, MACN-Pv-RN-1047, MACN-Pv-RN-1048, MACN-Pv-RN-1118, MACN-Pv-RN-1119, MLP-64-IV-16-1, MLP-64-IV-16-5(253) (lectotype), MLP-64-IV-16-6(203), MLP-64-IV-16-7(219), MLP-64-IV-16-8(209), MLP-64-IV-16-9(201), MLP-64-IV-16-10(221), MLP-64-IV-16-11, MLP-64-IV-16-12, MLP-64-IV-16-13, MLP-64-IV-16-14, MLP-64-IV-16-15, MLP-64-IV-16-16, MLP-64-IV-16-17, MLP-64-IV-16-18, MLP-64-IV-16-20, MLP-64-IV-16-21, MLP-64-IV-16-22, MLP-64-IV-16-23, MLP-64-IV-16-24, MLP-64-IV-16-25, MLP-64-IV-16-28, MLP-64-IV-16-30, MLP-64-IV-16-31(206), MPCA-Pv-528; MPCA-Pv-789/1; MPCA-Pv-791; Woodward (1896), Gasparini (1971), Bonaparte (1991, 1996), Andrade & Bertini (2008b), Fiorelli & Calvo (2008).

Localites: several outcrops in the Neuquén and Rio Negro provinces, Argentina

Formation: Bajo de La Carpa Formation, Neuquén Group. Neuquén Basin.

Age: Santonian–Campanian, Upper Cretaceous.

(13) *Mariliasuchus amarali* Carvalho & Bertini, 1999

Data from: MN-6298-V, MN-6756-V, UFRJ-DG-50-R(type), UFRJ-DG-56-R, UFRJ-DG-105-R, UFRJ-DG-106-R, UFRJ-DG-115-R, URC-R-67, URC-R-68, URC-R-69; Carvalho & Bertini (1999), Andrade (2005), Vasconcellos & Carvalho (2005).

Locality: Rio do Peixe, São Paulo State, Brazil.

Formation: Aracatuba Formation, Bauru Group. Bauru Basin.

Age: Campanian, Upper Cretaceous.

Notosuchia: Sphagesauridae (3 OTUs)

(14) *Adamantinasuchus navae* Nobre & Carvalho, 2006

Data from: UFRJ-DG-107-R (type), UFRJ-DG-216-R; Nobre & Carvalho (2006).

Locality: Rio do Peixe, São Paulo State, Brazil.

Formation: Aracatuba Formation, Bauru Group. Bauru Basin.

Age: Campanian, Upper Cretaceous.

(15) *Sphagesaurus huenei* Price, 1950

Data from: Pol (2003).

Locality: N São Paulo State, Brazil.

Formation: Adamantina Formation, Bauru Group. Bauru Basin.

Age: Campanian–Maastrichtian, Upper Cretaceous.

(16) *Caipirasuchus montealtensis* (Andrade & Bertini, 2008a)

Data from: Andrade (2005), Andrade & Bertini (2008a), Iori *et al*. (2016).

Locality: Monte Alto, N São Paulo State, Brazil.

Formation: Adamantina Formation, Bauru Group. Bauru Basin.

Age: Campanian–Maastrichtian, Upper Cretaceous.

Notosuchia: Baurusuchidae (1 OTU)

(17) *Baurusuchus pachecoi* Price, 1945

Data from: FEF-R-1-9; Price (1945), Carvalho *et al.* (2005; MPMA 62-0001-02).

Locality: 72 km SW of Vila do Veadinho (type locality), Paulo de Faria city. and several other localities spread at the N-NW São Paulo State, Brazil.

Formation: Adamantina Formation, Bauru Group. Bauru Basin.

Age: Campanian-Maastrichtian, Upper Cretaceous.

Observation: Here *B. salgadoensis* Carvalho *et al.* 2005 is treated as a subjective junior synonym of *B. pachecoi*.

Notosuchia: ‘Uruguaysuchidae’ (1 OTU)

(18) *Araripesuchus patagonicus* Ortega *et al.*, 2000

Data from: MUCPv-267, MUCPv-268, MUCPv-269 (holotype); Ortega *et al.* (2000).

Locality: El Chocon (Embalse Ezequiel Ramos Mexia), Neuquén Province, NW Patagonia, W Argentina.

Formation: Candeleros Member, Rio Limay Formation, Neuquén Group. Neuquén Basin.

Age: Albian-Cenomanian, ‘mid’ Cretaceous.

Notosuchia: Peirosauridae (2 OTUs)

(19) *Montealtosuchus arrudacamposi* Carvalho *et al*., 2007

Data from: Carvalho *et al.* (2007)

Locality: Monte Alto, N São Paulo State, Brazil.

Formation: Adamantina Formation, Bauru Group. Bauru Basin.

Age: Campanian–Maastrichtian, Upper Cretaceous.

(20) *Uberabasuchus terreficus* Carvalho *et al*., 2004

Data from: Carvalho *et al.* (2004).

Locality: Caieira outcrop, Peiropolis, Uberaba Municipality, S Minas Gerais State, SE Brazil.

Formation: Marilia Formation, Bauru Group. Bauru Basin.

Age: Campanian–Maastrichtian, Upper Cretaceous.

Notosuchia: ‘trematochampsidae’ (1 OTU)

(21) cf. *Hamadasuchus rebouli* Buffetaut, 1994

Data from: This OTU was scored for specimens referred to *H. rebouli* by Larsson & Sues (2007; mainly ROM-52620), not the type material. Therefore, the use of cf. *H. rebouli*.

Locality: SE Morocco.

Formation: Kem Kem beds.

Age: Albian–Cenomanian, ‘mid’ Cretaceous.

Notosuchia: Sebecidae (1 OTU)

(22) *Sebecus icaeorhinus* Simpson, 1937

Data from: AMNH 3160 (cast); Larsson & Sues (2007).

Locality: Canadon Hondo and Canadon Vaca, tributaries to the Rio Chico del Chubut, Chubut, Patagonia, Argentina.

Formation: Casamayor Formation.

Age: early–middle Eocene, Paleogene.

Notosuchia: Mahajangasuchidae (1 OTU)

(23) *Mahajangasuchus insignis* Buckley & Brochu, 1999

Data from: Buckley & Brochu (1999), Turner & Buckley (2008).

Locality: 1km SW Berivotra Village, SW Mahajanga, NW Madagascar.

Formation: Maevarano Formation. Mahajanga Basin.

Age: Campanian–Maastrichtian, Upper Cretaceous.

Neosuchia: atoposauridae (2 OTUs)

(24) *Alligatorium meyeri* Gervais, 1871

Data from: photographs of the holotype provided by Jon Tennant.

Locality: Cerin, France.

Formation: Cerin Lagerstätte.

Age: upper Kimmeridgian, Upper Jurassic.

(25) *Theriosuchus pusillus* Owen, 1878

Data from: NHMUK PV OR 48216 (lectotype), NHMUK PV OR 48330 (paratype), NHMUK PV OR 48262; Tennant *et al*. (2016).

Locality: Durlston Bay, Swanage, Dorset County, Jurassic Coast, S-SW England, UK.

Formation: “Beccles’ residuary marls” (beds 83–93; Clements, 1993), Worbarrow Tout Member (sensu Westhead & Mather, 1996), Lulworth Formation, Purbeck Limestone Group.

Age: Berriasian, Lower Cretaceous.

Neosuchia: Goniopholididae (8 OTUs)

(26) *Eutretauranosuchus* *delfsi* Mook, 1967

Data from: CM 8028 (holotype); Smith *et al*. (2010).

Locality: Canon City, Colorado, USA.

Formation: Morrison Formation. Morrison Basin.

Age: Kimmeridgian, Upper Jurassic.

(27) *Amphicotylus stovalli* (Mook, 1964)

Data from: CMC VP7798 (cast).

Locality: V97, Cimarron County, Oklahoma, USA.

Formation: Morrison Formation.

Age: ?Kimmeridgian, Upper Jurassic.

(28) *Goniopholis* *baryglyphaeus* Schwarz, 2002

Data from: Schwarz (2002).

Locality: Guimarota coal mine, Leiria, Portugal.

Formation: Lower lignite coal layer (`Fundschichten'), `Guimarota Strata', Alcobaca Formation.

Age: Kimmeridgian, Upper Jurassic.

(29) *Goniopholis kiplingi* Andrade *et al*., 2011.

Data from: DORCM 12154 (holotype); Andrade *et al*. (2011).

Locality: Durlston Bay, Swanage, Dorset County, Jurassic Coast, SSW England, UK.

Formation: Bed 129b (Clements 1993), Intemarine beds (sensu Wimbledon, 1995), Stair Hole Member (sensu Westhead & Mather 1996), Durlston Formation, Purbeck Limestone Group.

Age: Berriasian, Lower Cretaceous.

(30) *Goniopholis simus* Owen, 1878

Data from: NHMUK PV OR 41098 (type), NHMUK PV R 5814.

Localities: Swanage, Dorset County, Jurassic Coast, S-SW England; further referred materials from Schaumburg-Lippe Region, NW Germany.

Formations: Purbeck Limestone Group (UK) and Obernkirchen Sandstone, Buckeburg Member (Germany).

Age: Berriasian, Lower Cretaceous.

(31) *Anteophthalmosuchus hooleyi* Salisbury & Naish, 2011

Data from: NHMUK PV R 3876 (holotype); Salisbury & Naish (2011).

Locality: near the “Tie Pits”, Atherfield Point, Isle of Wight, UK.

Formation: Shepherd’s Chine Member, Vectis Formation, Wealden Group.

Age: Barremian to early Aptian, Lower Cretaceous.

(32) *Anteophthalmosuchus epikrator* Ristevski *et al*., 2018.

Data from: IWCMS 2001.446, IWCMS 2005.127; Martin *et al*. (2016).

Locality: Hanover Point, Isle of Wight, UK.

Formation: upper part of Wessex Formation, Wealden Group.

Age: Barremian, Lower Cretaceous.

Tethysuchia: Pholidosauridae (11 OTUs)

(33) *Elosuchus cherifensis* (Lavocat, 1955)

Data from: MNHN.F MRS 340, MNHN Escuillé collection; de Lapparent de Broin (2002), Meunier & Larsson (2016).

Locality: Hamadas, Morocco.

Formation: Kem Kem beds, Ifezouanae and Aoufous Formations.

Age: Cenomanian, Upper Cretaceous.

(34) *Elosuchus broinae* Meunier & Larsson, 2016

Data from: MNHN.F SAM 129 (holotype), de Lapparent de Broin (2002); Meunier & Larsson (2016).

Locality: Gara Samani, Algeria.

Formation: unnamed formation.

Age: upper Albian, Lower Cretaceous.

(35) *Vectisuchus leptognathus* Buffetaut & Hutt, 1980

Data from: SMNS 50984 (holotype).

Locality: Isle of Wight, UK.

Formation: Vectis Formation, Wealden Group. Wessex Sub-basin.

Age: Barremian–?early Aptian, Lower Cretaceous.

(36) *Pholidosaurus schaumburgensis* von Meyer, 1841

Data from: casts of the Koken (1887) specimens (including MB.R.1965, MB.R.1966, MB.R.1970.304); the natural external and internal moulds of Bückeburg specimens (MB.R.2025.1, two MB.R.unumbered specimens); Koken, 1887.

Locality: quarry near Harrel im Furstentum, Schaumburg-Lippe Region, NW Germany.

Formation: Obernkirchen Member, Bückeburg Formation.

Age: Berriasian, Lower Cretaceous.

Observation: Only specimens from the Bückeburg Formation are used to score this OTU.

(37) *Pholidosaurus* sp. (Charente)

Data from: Martin *et al*. (2016b).

Locality: Cherves-de-Cognac, Carrière de Champblanc, Charente Department, SW France.

Formation: Horizon C36.

Age: Berriasian, Lower Cretaceous.

(38) *Meridiosaurus vallisparadisi* Fortier *et al*., 2011

Data from: Fortier *et al*. (2011).

Locality: Valle Edén locality, near Tacuarembó city, Uruguay.

Formation: fluviolacustrine sandstone facies of the Batoví Member, Tacuarembó Formation

Age: ?Kimmeridgian-Tithonian, Upper Jurassic.

(39) *Chalawan thailandicus* (Buffetaut & Ingavat, 1980)

Data from: Buffetaut & Ingavat (1980), Martin *et al*. (2014).

Localities: Nong Bua Lam Phu (type locality) and Kham Phok, NE Thailand.

Formation: upper part of Phu Kradung Formation, Khorat Group. Khorat Basin.

Age: Early Cretaceous.

(40) *Sarcosuchus hartti* (Marsh, 1896) [fragmentary taxon]

Data from: NHMUK PV R 3423; Buffetaut & Taquet (1977).

Locality: outcrop in the vicinity of Setubal, Bahia State, NE Brazil.

Formation: unclear.

Age: Lower Cretaceous.

Observation: This OTU is scored solely for the lower jaw referred to *S. hartti* by Buffetaut & Taquet (1977).

(41) *Sarcosuchus imperator* de Broin & Taquet, 1966

Data from: MNHN.F GDF 662; de Broin & Taquet (1966), Buffetaut & Taquet (1977), Sereno *et al.* (2001).

Locality: outcrop in the vicinities of the Gadoufaoua, Agadez Province, Niger.

Formation: Elrhaz Formation. Tegama Basin.

Age: Aptian, Lower Cretaceous.

(42) cf. *Terminonaris robusta* Mook, 1934

Data from: Wu *et al*. (2001b), Larsson & Sues (2007).

Locality: SMNH locality 63E04-001, approximately 5km east of Highway 23, the southern bank of the Carrot River, southwest of the Pasquia Hills, Saskatchewan, Canada.

Formation: Keld Member, Favel Formation.

Age: upper Cenomanian? to lower Turonian, Upper Cretaceous.

Observation: This OTU is based solely on the Canadian material referred to *T. robusta*.

(43) *Oceanosuchus boecensis* Hua *et al*., 2007

Data from: Hua *et al*. (2007), Lepage *et al*. (2008).

Locality: La Boëce, near Mortagne-au-Perche, Orne, Vasse-Normandie, France.

Formation: base of hard-ground Coulimer 2.

Age: lower Cenomanian, Upper Cretaceous.

Tethysuchia: Basal Dyrosauroidea (2 OTUs)

(44) *Pholidosaurus purbeckensis* (Mansel-Pleydell, 1888)

Data from: DORCM G.27, DORCM G.97 (holotype), NHMUK PV OR 28432, NHMUK PV R 3414, NHMUK PV R 3956, NHMUK PV R 36721.

Locality: type locality unclear, thought to be Isle of Purbeck, UK.

Formation: Purbeck Formation, Purbeck Limestone Group.

Age: Berriasian, Lower Cretaceous.

(45) *Fortignathus felixi* Young *et al*., 2016 [fragmentary taxon]

Data from: MNHN.F INA 21, MNHN.F INA 22, MNHN.F INA 25 (holotype).

Locality: West of In Abangharit, Agadez District, Niger.

Formation: Echkar Formation, Tegma Series.

Age: upper Albian to lower Cenomanian, ‘mid’ Cretaceous.

Tethysuchia: Dyrosauridae (15 OTUs)

(46) *Acherontisuchus guajiraensis* Hastings *et al*., 2011 [fragmentary taxon]

Data from: UF/IGM 34 (holotype), UF/IGM 35, UF/IGM 36, UF/IGM 37, UF/IGM 38 & UF/IGM 39; Hastings *et al*. (2011).

Locality: below Coal Seam 85 in the La Puente Pit, Cerrejón coal mine, Guajira Department, north-eastern Colombia.

Formation: Cerrejón Formation.

Age: middle–late Paleocene, Palaeogene.

(47) *Anthracosuchus balrogus* Hastings *et al*., 2015

Data from: UF/IGM 67 (holotype), UF/IGM 68 (paratype), UF/IGM 69 & UF/IGM 70; Hastings *et al*. (2015).

Locality: clay layer below Coal Seam 90 in the La Puente Pit, Cerrejón coal mine, Guajira Department, north-eastern Colombia.

Formation: Cerrejón Formation.

Age: middle–late Paleocene, Palaeogene.

(48) *Arambourgisuchus khouribgaensis* Jouve *et al*., 2005a.

Data from: Jouve *et al*. (2005a).

Locality: Phosphate mine in ‘Sidi Chenane’ area, in NE part of Ouled Aboun Basin, Morocco.

Formation: couche (= bed/layer) 2a.

Age: Thanetian, Paleocene, Palaeogene.

(49) *Atlantosuchus coupatezi* Buffetaut, 1979

Data from: Jouve *et al*. (2008).

Locality: ‘Sidi Chenane’ area, in NE part of Ouled Aboun Basin, Morocco.

Formation: not given.

Age: Danian, Paleocene, Palaeogene.

(50) *Cerrejinosuchus improcerus* Hastings *et al*., 2010

Data from: UF/IGM 29 (holotype), UF/IGM 30, UF/IGM 31 & UF/IGM 32; Hastings *et al*. (2010).

Locality: clay layer below Coal Seam 90 in the La Puente Pit, Cerrejón coal mine, Guajira Department, north-eastern Colombia.

Formation: Cerrejón Formation.

Age: middle–late Paleocene, Palaeogene.

(51) *Chenanisuchus* *lateroculi* Jouve *et al*., 2005b

Data from: Jouve *et al*. (2005b).

Locality: ‘Sidi Chenane’ area, in NE part of Ouled Aboun Basin, Morocco.

Formation: couche (= bed/layer) 2a.

Age: Thanetian, Paleocene, Palaeogene.

(52) *Congosaurus bequaerti* Dollo, 1914

Data from: Jouve & Schwarz (2004), Schwarz *et al.* (2006), Schwarz-Wings *et al.* (2009).

Locality: Cacongo, Cabinda Province, Angola.

Formation: Bed no. 8.

Age: Danian, Paleocene, Palaeogene.

(53) *Dyrosaurus maghribensis* Jouve *et al*., 2006

Data from: Jouve *et al.* (2006).

Locality: phosphate mine of Mera el Arech, in Oulad Abdoun Basin, Morocco.

Formation: couche (= bed/layer) 1.

Age: Ypresian, lower Eocene, Palaeogene.

(54) *Dyrosaurus phosphaticus* (Thomas, 1893)

Data from: MNHN.F ALG 1, MNHN.F ALG 2; Jouve (2005).

Localities: north of Djebel Teldj, near Metlaoui, Tunisia and Tébessa, north-east Algeria.

Formation: “phosphate layer” (Tunisia).

Age: Ypresian, lower Eocene, Palaeogene.

(55) *Guarinisuchus munizi* Barbosa *et al*., 2008

Data from: Barbosa *et al.* (2008).

Locality: Poty Quarry, Paulista, NE of Pernambuco State, Brazil.

Formation: Maria Farinha Formation. Paraiba Basin.

Age: upper Danian, Lower Paleocene, Palaeogene.

(56) *Hyposaurus rogersii* Owen, 1849

Data from: Troxell (1925), Denton *et al*. (1997).

Localities: Numerous, including: Inversand Company Marl Pit, Gloucester County, New Jersey, USA; Santee rediversion canal, St. Stephen, Berkeley County, South Carolina, USA.

Formation: Hornerstown Formation (NJ), Williamsburg Formation (SC).

Age: Maastrichtian, Upper Cretaceous (NJ), upper Paleocene, Palaeogene (SC).

(57) *Phosphatosaurus gavialoides* Bergounioux, 1955

Data from: Buffetaut (1978), Hill *et al*. (2008).

Locality: near Metlaoui, Tunisia and ‘Mali-20’, south of Tamaguélet, Tilemsi valley region, Mali.

Formation: “phosphate layer” (Tunisia) and unnamed formation in Taoudeni Basin (Mali).

Age: Ypresian, lower Eocene, Palaeogene.

(58) *Rhabdognathus keiniensis* Jouve, 2007.

Data from: Jouve (2007).

Locality: Cheit Keini and In Farghas, Tilemsi valley region, Mali.

Formation: unnamed formation in Taoudeni Basin.

Age: Paleocene, Palaeogene.

(59) *Rhabdognathus aslerensis* Jouve, 2007

Data from: Brochu *et al*. (2002), Jouve (2007).

Locality: ‘Mali-5’, near Asler, north-west of Tamaguélet, Tilemsi valley region, Mali.

Formation: unnamed formation in Taoudeni Basin.

Age: Maastrichtian or Paleocene.

(60) *Sabinosuchus coahuiliensis* Shiller *et al*., 2016 [fragmentary taxon]

Data from: Shiller *et al*. (2016).

Locality: El Rancho Soledad, Coahuila, Mexico.

Formation: Escondido Formation.

Age: Maastrichtian, Upper Cretaceous.

(61) *Sokotosuchus ianwilsoni* Halstead, 1975

Data from: Buffetaut (1979).

Locality: Sokoto area, NW Nigeria.

Formation: Dukamaje Formation.

Age: Maastrichtian, Upper Cretaceous.

Neosuchia: Bernissartiidae (2 OTUs)

(62) *Bernissartia fagesii* Dollo, 1883

Data from: Norell & Clark (1990).

Locality: Sainte-Barbe coal mine, Bernissart, Belgium.

Formation: Sainte-Barbe Clays Formation.

Age: Berriasian–Barremian, Lower Cretaceous.

(63) *Koumpiodontosuchus aprosdokiti* Sweetman *et al*., 2015

Data from: IWCMS 2012.203 and IWCMS 2012.204 (holotype), Sweetman *et al*. (2015).

Locality: The foreshore near Yaverland, SE coast of Isle of Wight, UK.

Formation: from one of the plant debris beds occurring between beds 26 and 38, Wessex Formation.

Age: Barremian, Lower Cretaceous.

Neosuchia: Susisuchidae (2 OTUs)

(64) *Susisuchus anatoceps* Salisbury *et al*., 2003

Data from: SMNK PAL3804 (holotype); Salisbury *et al.* (2003, 2006).

Locality: Araripe Plateau, NE Brazil.

Formation: Crato Member, Santana Formation. Araripe Basin.

Age: Aptian–Albian, Lower Cretaceous.

(65) *Isisfordia duncani* Salisbury *et al*., 2006

Data from: Salisbury *et al.* (2006; QM-F-36211, QM-F-44320).

Locality: outcrop near Isisford, Queensland, Australia.

Formation: Winton Formation.

Age: Albian–Cenomanian, ‘mid’ Cretaceous.

Eusuchia: Hylaeochampsidae *sensu lato* (3 OTUs)

(66) *Iharkutosuchus makadii* Ősi *et al*., 2007

Data from: MTM 2006.52.1 (holotype), MTM 2006.53.1, MTM PAL 2013.51.1, MTM PAL 2013.58.1; Ősi *et al*. (2007), Ősi (2008), Ősi (2014).

Locality: Iharkút, Bakony Mountains, western Hungary.

Formation: Csehbánya Formation.

Age: Santonian, Upper Cretaceous.

(67) *Pachycheilosuchus trinquei* Rogers, 2003

Data from: Rogers (2003); osteoderms re-scored based on Buscalioni *et al*. (2011).

Locality: SMU locality 331, Erath County, Texas, USA.

Formation: Glen Rose Formation.

Age: Albian, Lower Cretaceous.

(68) *Pietraroiasuchus ormezzanoi* Buscalioni *et al*., 2011

Data from: Buscalioni *et al*. (2011).

Locality: locality of ‘Civita di Pietraroia’, Mt Matese, southern Italy.

Formation: ‘Civita di Pietraroia Cave’.

Age: lower Albian, Lower Cretaceous.

Eusuchia: Crocodylia (4 OTUs)

(69) *Gavialis gangeticus* (Gmelin, 1879)

Data from: comparative collection held in the Palaeontology and Zoology departments of NHMUK.

Distribution: river systems of Brahmaputra, Indus, Ganges, Mahanadi; Burma, Buthan, India, Nepal and Pakistan.

Age: extant – Holocene, Quaternary.

(70) *Crocodylus niloticus* (Laurenti, 1768)

Data from: comparative collection held in the Palaeontology and Zoology departments of NHMUK; and in the Life Sciences Faculty, Ohio University.

Distribution: river systems of several African countries, especially the Nile River, Egypt.

Age: extant – Holocene, Quaternary.

(71) *Crocodylus porosus* (Schneider, 1801)

Data from: comparative collection held in the Palaeontology and Zoology departments of NHMUK; and in the Life Sciences Faculty, Ohio University.

Distribution: freshwater to brackish areas of several countries, from SE Asia to Australia.

Age: extant – Holocene, Quaternary.

(72) *Alligator mississippiensis* (Daudin, 1802)

Data from: NHMUK ZD 290, NHMUK ZD 1973-2-21-2, NHMUK ZD 1974-3010, NHMUK ZD 1975-1424, NHMUK ZD II-1-I.

Distribution: swamp to low-energy river systems of SE USA, most noticeably in Florida.

Age: extant – Holocene, Quaternary.

Thalattosuchia: Teleosauroidea (18 OTUs)

(73) *Aeolodon priscus* (von Sömmerring, 1814)

Data from: NMHUK PV R 1086 (holotype), MNHN.F CNJ 78a.

Localities: Daiting, S Germany, and Canjuers, Var, France.

Formation: Mörnsheim Formation (type locality) and Canjuers consveration Lagerstätte.

Age: lower Tithonian, Upper Jurassic.

(74) *Machimosaurus buffetauti* Young *et al*., 2015

Data from: SMNS 91415 (holotype); Young *et al*. (2014).

Locality: Am Hörnle Quarry, Neuffen, Baden-Württemberg, Germany.

Formation: Lacunosamergel Formation.

Age: *Ataxioceras hypselocyclum* Sub-Mediterranean ammonite Zone (=Weißer Jura gamma 2), lower Kimmeridgian, Upper Jurassic.

Observation: The correct nominal authority is the short taxonomic note Young *et al*., 2015 not Young *et al*. 2014 (where the new taxon was described).

(75) *Machimosaurus hugii* von Meyer, 1837

Data from: Young *et al*. (2014).

Locality: Kreuzen Quarry at St. Verena, near Solothurn, Canton Solothurn, Switzerland (lectotype locality) and Guimarota coal mine, Leiria, NW Portugal.

Formation: Solothurn Turtle Limestone, Reuchenette Formation (lectotype locality) and Guimarota Strata, Alcobaça Formation.

Age: Kimmeridgian, Upper Jurassic.

(76) *Machimosaurus mosae* Sauvage & Liénard, 1879

Data from: Hua (1999), Young *et al*. (2014).

Locality: beach near Ambleteuse, Boulonnais, Département du Pas-de-Calais, Nord Pas-de-Calais, France (neotype locality).

Formation: Argiles de Châtillon Formation (neotype locality).

Age: From either the *Aulacostephanus. autissiodorensis* Sub-Boreal ammonite Zone, uppermost Kimmeridgian, or the *Gravesia. gigas*/*Pectinaties elegans* Sub-Boreal ammonite Zone, lowermost Tithonian; Upper Jurassic (neotype locality).

(77) *Machimosaurus rex* Fanti *et al*., 2016

Data from: Fanti *et al*. (2016); high-resolution images of the holotype provided by Andrea Cau.

Locality: Touil el Mhahir, Tataouine Governorate, Tunisia.

Formation: Douiret Sand Member, Douiret Formation.

Age: Hauterivian, Lower Cretaceous.

(78) *Mycterosuchus nasutus* Andrews, 1913

Data from: NHMUK PV R 2617 (holotype), Andrews (1913).

Locality: Peterborough, UK.

Formation: Peterborough Member, Oxford Clay Formation, Ancholme Group.

Age: middle Callovian, Middle Jurassic.

(79) Chinese teleosauroid skull referred to *Peipehsuchus teleorhinus* by Li (1993)

Data from: specimen photographs of IVPP V 10098 provided by Eric Wilberg.

Locality: Daxian, Szechuan, China.

Formation: Ziliujing Formation.

Age: Lower Jurassic.

(80) *Platysuchus multiscrobiculatus* (Berckhemer, 1929)

Data from: SMNS 9930 (holotype).

Locality: Holzmaden, Baden-Württemberg, Germany.

Formation: Posidonia Shale Formation.

Age: lower Toarcian, Lower Jurassic.

(81) *Steneosaurus bollensis* (von Jäger, 1828)

Data from: NHMUK PV R 324, NHMUK PV R 756, NHMUK PV R 1088, NHMUK PV R 5703, NHMUK PV OR 14436, NHMUK PV OR 14438, SMNS 849, SMNS 9427, SMNS 9428, SMNS 17484, SMNS 20280, SMNS 53422.

Localities: Baden-Württemberg, Germany; Yorkshire, UK.

Formation: Posidonia Shale Formation (Germany) and Whitby Mudstone Formation (UK).

Age: lower Toarcian, Lower Jurassic.

(82) *Steneosaurus brevior* Blake, 1876

Data from: NHMUK PV OR 14781 (holotype).

Locality: Whitby, Yorkshire, UK.

Formation: Mulgrave Shale Member, Whitby Mudstone Formation, Lias Group.

Age: *Harpoceras serpentinum* Sub-Boreal ammonite Zone, lower Toarcian, Lower Jurassic.

(83) *Steneosaurus edwardsi* Eudes-Deslongchamps, 1868a

Data from: NHMUK PV R 2074, NHMUK PV R 2865, NHMUK PV R 3701.

Locality: Peterborough, UK.

Formation: Peterborough Member, Oxford Clay Formation, Ancholme Group.

Age: middle Callovian, Middle Jurassic.

(84) *Steneosaurus gracilirostris* Westphal, 1961

Data from: NHMUK PV OR 14792 (holotype), NHMUK PV OR 15500 (paratype).

Locality: Whitby, Yorkshire, UK.

Formation: Alum Shale Member, Whitby Mudstone Formation, Lias Group.

Age: *Hildoceras bifrons* Sub-Boreal ammonite Zone, lower Toarcian, Lower Jurassic.

(85) *Steneosaurus larteti* (Eudes-Deslongchamps, 1868b)

Data from: OUMNH J29850.

Locality: Enslow Bridge, Oxfordshire, UK.

Formation: Great Oolite Group.

Age: Bathonian, Middle Jurassic.

(86) *Steneosaurus leedsi* Andrews, 1909

Data from: NHMUK PV R 2619, NHMUK PV R 3320 (holotype), NHMUK PV R 3806.

Locality: Peterborough, UK.

Formation: Peterborough Member, Oxford Clay Formation, Ancholme Group.

Age: middle Callovian, Middle Jurassic.

(87) *Steneosaurus heberti* Morel de Glasville, 1876

Data from: MNHN.F 13.1890 (holotype).

Locality: Villers-sur-mer, Calvados, France.

Formation: Marnes de Dives Formation.

Age: upper Callovian, Middle Jurassic.

(88) *Lemmysuchus obtusidens* Andrews, 1909

Data from: NHMUK PV R 3168 (holotype).

Locality: Peterborough, UK.

Formation: Peterborough Member, Oxford Clay Formation, Ancholme Group.

Age: middle Callovian, Middle Jurassic.

(89) *Teleosaurus cadomensis* (Lamouroux, 1820)

Data from: MNHN.F AC 8746, MNHN.F RJN 464, NHMUK PV OR 119, NHMUK PV OR 32588, NHMUK PV OR 32657, NHMUK PV OR 32680, casts: NHMUK PV R 880 and NHMUK PV R 880a; Eudes-Deslongchamps (1867-69); Jouve (2009).

Locality: Allemagne, 3km south of Caen, Calvados, Normandy, France.

Formation: “Calcaire de Caen”.

Age: Bathonian, Middle Jurassic.

(90) *Teleosaurus megarhinus* Hulke, 1871

Data from: NHMUK PV OR 43086 (holotype), DORCM G.05067i-v, Vignaud (1995).

Locality: Kimmeridge, Dorset, UK.

Formation: Dorset succession, lower Kimmeridge Clay Formation, Ancholme Group.

Age: *Aulacostephanus* *autossiodorensis* Sub-Boreal ammonite Zone, upper Kimmeridgian, Upper Jurassic.

Thalattosuchia: Basal MetriorhynchoidAE (8 OTUs)

(91) *Eoneustes bathonicus* (Mercier, 1933) Young *et al*., 2010

Data from: Mercier (1933).

Locality: Port-en-Bessin, Calvados, Normandy, France.

Formation: “Calcaire de Caen”.

Age: Bathonian, Middle Jurassic.

(92) *Eoneustes gaudryi* (Collot, 1905) Young *et al*., 2010

Data from: NHMUK PV R 3353 (holotype).

Locality: Saint-Seine-l'Abbaye, Département du Cote d'Or, Bourgogne, France.

Formation: “Calcaires blancs jaunâtres des de Bourgogne”.

Age: lower Bathonian, Middle Jurassic.

(93) Hungarian thalattosuchian

Data from: MTM V.97.

Locality: eastern Gerecse Mountains, Hungary.

Formation: Bed 13, uppermost Kisgerecse Marl Formation.

Age: *Grammoceras striatulum* ammonite Subzone, *Grammoceras thouarense* ammonite Zone, upper Toarcian, Early Jurassic.

(94) Metriorhynchoidea indeterminate (Chile) [fragmentary taxon]

Data from: Gasparini *et al*. (2000).

Locality: Quebrada La Iglesia, Copiapo, Central-east Chile.

Formation: upper part of the Lautaro Formation.

Age: lower Bajocian, Middle Jurassic.

(95) *Zoneait nargorum* Wilberg, 2015a

Data from: Wilberg (2015a).

Locality: near Suplee, Oregon, USA.

Formation: Weberg Member, Snow-shoe Formation.

Age: uppermost Aalenian or lowermost Bajocian, Middle Jurassic.

(96) *Peipehsuchus teleorhinus* Young, 1948 [fragmentary taxon]

Data from: photographs of the holotype provided by Eric Wilberg.

Locality: Beipei, Szechuan, China.

Formation: Ziliujing Formation.

Age: Lower Jurassic.

Observation: This OTU is solely based on the holotype, with the skull referred to *Peipehsuchus teleorhinus* treated as a separate OTU.

(97) *Pelagosaurus typus* Bronn, 1841

Data from: BRLSI M.1415, BRLSI M.1416, BRLSI M.1420, MNHN.F RJN 463, MTM V.52.2516, NHMUK PV OR 19735, NHMUK PV OR 32599, SMNS 8666, SMNS 17758, SMNS 50374, SMNS 80066; Pierce & Benton (2006).

Localities: Numerous, including: Amaye-sur-Orne, Caen, and Curcy, France; Nabern near Kirchheim, S Germany; Holzmaden, Bad Boll, Ohmden and Ohmdenhausen, Swabian Jura, S Germany; Ilminster, Somerset, UK; Whitby, Yorkshire, England.

Formations: Numerous, including: Posidonia Shale Formation (Germany) and Whitby Mudstone Formation (UK).

Age: lower Toarcian, Lower Jurassic.

Observation: this OTU includes *P. moorei* as a subjective junior synonym of *P. typus*, following Pierce & Benton (2006).

(98) *Teleidosaurus calvadosii* (Eudes-Deslongchamps, 1866)

Data from: NHMUK PV R 2619 (plastoholotype); Eudes-Deslongchamps (1867-69).

Locality: Allemagne, 3km south of Caen, Calvados, Normandy, France.

Formation: “Calcaire de Caen”.

Age: Bathonian, Middle Jurassic.

Thalattosuchia: Metriorhynchidae: Metriorhynchinae (18 OTUs)

(99) '*Dakosaurus*' *lissocephalus* Seeley, 1869

Data from: CAMSM J29419 (holotype).

Locality: Ely, Cambridgeshire, UK

Formation: lower Kimmeridge Clay Formation, Ancholme Group.

Age: upper Kimmeridgian, Upper Jurassic.

(100) *Cricosaurus araucanensis* (Gasparini & Dellapé, 1976) Young & Andrade, 2009

Data from: MLP-72-IV-7-1 (holotype), MLP-72-IV-7-2; Gasparini & Dellapé (1976), Fernández & Gasparini (2000, 2008), Fernández & Herrera (2009), Herrera *et al.* (2009).

Locality: Argentina.

Formation: Vaca Muerta Formation, Mendoza Group. Neuquén Basin.

Age: lower Tithonian, Upper Jurassic.

(101) *Cricosaurus elegans* (Wagner, 1852) Wagner, 1858

Data from: BSPG AS I 504.

Locality: Daiting, near Monheim, Bayern, Germany.

Formations: Mörnsheim Formation.

Age: *Hybonoticeras* *hybonotum* Tethys ammonite Zone, lower Tithonian, Upper Jurassic.

(102) *Cricosaurus lithographicus* Herrera *et al*., 2013

Data from: Herrera *et al*. (2013).

Locality: El Ministerio Quarry, Los Catutos Area, Zapala Department, Neuquén Province, Argentina.

Formation: Los Catutos Member, Vaca Muerta Formation, Mendoza Group. Neuquén Basin.

Age: upper lower or middle upper Tithonian, Upper Jurassic.

(103) '*Cricosaurus*' *macrospondylus* (Koken, 1883) Young & Andrade, 2009

Data from: Hua *et al*. (2000).

Locality: Barret-le-Bas, Département du Hautes-Alpes, Provence-Alpes-Côte d'Azur, France.

Formation: not given.

Age: *Busnardoites* *campylotoxus* ammonite Zone, lower Valanginian, Lower Cretaceous.

Observation: This OTU is solely based on the French referred specimen.

(104) '*Cricosaurus*' *saltillensis* (Buchy *et al*., 2006) Young & Andrade, 2009

Data from: Buchy *et al*. (2006); Buchy *et al*. (2013).

Locality: Sierra de Buñuelas, near Gomez Farías, State of Coahuila, Mexico.

Formation: La Caja Formation.

Age: lower Tithonian, Upper Jurassic.

(105) *Cricosaurus schroederi* (Kuhn, 1936) Young & Andrade, 2009

Data from: Karl *et al*. (2006b); photographs of the holotype provided by Nils Knötschke.

Locality: Sachsenhagen, Lower Saxony, Germany.

Formation: ‘*Platylenticeras* beds’.

Age: lower Valanginian, Lower Cretaceous.

(106) *Cricosaurus suevicus* (Fraas, 1901) Young & Andrade, 2009

Data from: SMNS 9808 (lectotype), SMNS 90513; Fraas (1901, 1902).

Locality: Nusplingen, Zollernalbkreis, Baden-Württemberg, Germany.

Formation: Nusplingen Plattenkalk.

Age: *Hybonoticeras* *beckeri* Tethys ammonite Zone (= Malm Zeta 1), upper Kimmeridgian, Upper Jurassic.

(107) *Cricosaurus* sp. (Cuba)

Data from: Gasparini & Iturralde-Vinent (2001).

Locality: Viñales Valley, western Cuba.

Formation: Jagua Vieja Member, Jagua Vieja Formation.

Age: middle or upper Oxfordian, Upper Jurassic.

(108) *Cricosaurus vignaudi* (Frey *et al*., 2002) Young & Andrade, 2009

Data from: Frey *et al*. (2002).

Locality: Mazatepec, State of Puebla, Mexico.

Formation: La Pimienta Formation.

Age: ‘middle’ Tithonian, Upper Jurassic.

(109) *Gracilineustes acutus* (Lennier, 1887) Young *et al*., 2010

Data from: Lennier (1887).

Locality: Cap de la Hève, Département du Seine-Maritime, Haute-Normandie, France.

Formation: Marnes de Bléville Formation.

Age: *Rasenia cymodoce* Sub-Boreal ammonite Zone, lower Kimmeridgian, Upper Jurassic.

(110) *Gracilineustes leedsi* (Andrews, 1913) Young *et al*., 2010

Data from: CAMSM J64297, GLAHM V973, GLAHM V974, GLAHM V975, PETMG R24, PETMG R72, NHMUK PV R 2031, NHMUK PV R 2042, NHMUK PV R 3014, NHMUK PV R 3015, NHMUK PV R 3540 (holotype), NHMUK PV R 3899, NHMUK PV R 5793.

Locality: Peterborough, UK.

Formation: Peterborough Member, Oxford Clay Formation, Ancholme Group.

Age: middle Callovian, Middle Jurassic.

(111) *Maledictosuchus riclaensis* Parrilla-Bel *et al*., 2013

Data from: Parrilla-Bel *et al*. (2013).

Locality: ‘‘Barranco de la Paridera’’, Ricla, Zaragoza, Spain.

Formation: Ágreda Formation.

Age: *Erymnoceras coronatum* Sub-Mediterranean ammonite Zone, Middle Callovian, Middle Jurassic.

(112) Metriorhynchinae indeterminate (Cuba) [fragmentary taxon]

Data from: USNM 419640.

Locality: Viñales Valley, western Cuba.

Formation: Jagua Vieja Member, Jagua Vieja Formation.

Age: middle or upper Oxfordian, Upper Jurassic.

(113) *Metriorhynchus geoffroyii* von Meyer, 1832 [fragmentary taxon]

Data from: MHNG V-2232 (holotype).

Locality: Le Havre, Département de Seine-Maritime, Haute-Normandie, France.

Formation: not given.

Age: Kimmeridgian, Upper Jurassic.

(114) '*Metriorhynchus*' *palpebrosus* (Phillips, 1871)

Data from: OUMNH J.29823 (holotype).

Locality: Shotover Hill, Oxfordshire, UK.

Formation: Kimmeridge Clay Formation.

Age: most likely lower Tithonian, Upper Jurassic.

(115) *Metriorhynchus superciliosus* (de Blainville, 1853)

Data from: AMNH 997, GLAHM V942, GLAHM V963, GLAH V964, GLAHM V965, GLAHM V966, GLAHM V971, GLAHM V982, GLAHM V983, GLAHM V984, GLAHM V985, GLAHM V987, GLAHM V988, GLAHM V989, GLAHM V996, GLAHM V1004, GLAHM V1015, GLAHM V1027, GLAHM V1140, GLAHM V1142, GLAHM V1143, NHMUK PV R 1666, NHMUK PV R 2030, NHMUK PV R 2032, NHMUK PV R 2036, NHMUK PV R 2044, NHMUK PV R 2051, NHMUK PV R 2053, NHMUK PV R 2054, NHMUK PV R 2055, NHMUK PV R 2058, NHMUK PV R 2067, NHMUK PV R 3900, NHMUK PV R 6859, NHMUK PV R 6860, PETMG R10, PETMG R17, PETMG R18, PETMG R20, PETMG R42, PETMG R180, RMS M150, SMNS 10115, SMNS 10116, SMNS 81689; Andrews (1913).

Localities: outcrops from England and France.

Formations: Primarily: Oxford Clay Formation and Marnes de Dives Formation.

Age: lower Callovian to lower Oxfordian, Middle-Upper Jurassic.

(116) *Rhacheosaurus gracilis* von Meyer, 1831

Data from: AMNH 4804 and NHMUK PV R3961 (plastoholotypes), NHMUK PV R 3948.

Localities: Daiting (type locality) and Eichstätt, S Germany.

Formations: Mörnsheim Formation (type locality) and Solnhofen Formation.

Age: *Hybonoticeras* *hybonotum* Tethys ammonite Zone, lower Tithonian, Upper Jurassic.

Thalattosuchia: Metriorhynchidae: Geosaurinae (24 OTUs)

(117) cf. *Torvoneustes* [fragmentary taxon]

Data from: MANCH J6459.

Locality: Headington, Oxfordshire, UK.

Formation: most likely Beckley Sand Member, Kingston Formation.

Age: middle Oxfordian, Upper Jurassic.

(118) *Torvoneustes* *mexicanus* (Wieland, 1910) [fragmentary taxon]

Data from: Barrientos-Lara *et al*. (2016).

Locality: inprecise, but likley near Tlaxiaco, Oaxaca, Mexico.

Formation: suggested to be Sabinal Formation.

Age: suggested to be Kimmeridgian, Upper Jurassic.

(119) *Dakosaurus andiniensis* Vignaud & Gasparini, 1996

Data from: Gasparini *et al.* (2006), Pol & Gasparini (2009).

Localities: in the provinces of Neuquén and Mendoza, Argentina.

Formations: Vaca Muerta Formation, Mendoza Group and Neuquén Group. Neuquén Basin.

Age: upper Tithonian, Upper Jurassic. Possibly also Berriasian, Lower Cretaceous.

(120) *Dakosaurus maximus* (Plieninger, 1846)

Data from: NHMUK PV OR 33186, NHMUK PV OR 35766, NHMUK PV OR 35835-7, SMNS 8203 (neotype), SMNS 80148, SMNS 82043; Plieninger, 1846, Young & Andrade (2009), Andrade (2010), Andrade *et al.* (2010).

Localities: Numerous outcrops in England, Germany and France.

Formations: Numerous, including: Kimmeridge Clay Formation, Solnhofen Formation, Mergelstätten Formation and Nusplingen Plattenkalk.

Age: upper Kimmeridgian-lower Tithonian, Upper Jurassic.

(121) Geosaurinae indeterminate (Argentina) [fragmentary taxon]

Data from: Gasparini *et al*. (2005).

Locality: Chacay Melehue, Neuquén Province, Argentina.

Formation: Los Molles Formation.

Age: upper Bathonian, Middle Jurassic.

(122) *Geosaurus giganteus* (von Sömmerring, 1816)

Data from: NHMUK PV R 1229 (holotype), NHMUK PV R 1230, NHMUK PV OR 37016, NHMUK PV OR 37020; Young & Andrade (2009), Andrade (2010), Andrade *et al.* (2010).

Localities: Daiting (type locality) and Eichstätt, Southern Germany.

Formations: Mörnsheim Formation (type locality) and Solnhofen Formation.

Age: *Hybonoticeras* *hybonotum* Tethys ammonite Zone, lower Tithonian, Upper Jurassic.

(123) *Geosaurus grandis* (Wagner, 1858)

Data from: BSPG AS-VI-1 (holotype); Young & Andrade (2009), Andrade (2010), Andrade *et al.* (2010).

Locality: Daiting, near Monheim, Bayern, Germany.

Formations: Mörnsheim Formation.

Age: *Hybonoticeras* *hybonotum* Tethys ammonite Zone, lower Tithonian, Upper Jurassic.

(124) *Geosaurus lapparenti* (Debelmas & Strannoloubsky, 1957)

Data from: Debelmas (1952), Debelmas & Strannoloubsky (1957).

Locality: La Martre, Département du Var, Provence-Alpes-Côte d'Azur, France.

Formation: not given.

Age: *Neocomites peregrinus* ammonite Zone, upper Valanginian, Lower Cretaceous.

(125) *Ieldraan melkshamensis* Foffa *et al*., 2017

Data from: NHMUK PV OR 46797.

Locality: Melksham, Wiltshire, UK.

Formation: Oxford Clay Formation, Ancholme Group.

Age: Callovian, Middle Jurassic.

(126) '*Metriorhynchus*' *brachyrhynchus* (Eudes-Deslongchamps, 1868c)

Data from: GLAHM V978, GLAHM V995, NHMUK PV R 3541, NHMUK PV R 3699, NHMUK PV R 3700 (neotype), NHMUK PV R 3804, NHMUK PV R 4763, PETMG R19.

Locality: Peterborough, UK.

Formation: Peterborough Member, Oxford Clay Formation, Ancholme Group.

Age: middle Callovian, Middle Jurassic.

(127) '*Metriorhynchus*' *casamiquelai* Gasparini & Chong, 1977

Data from: Gasparini & Chong (1977).

Locality: Quebrada Sajasa, Región de Antofagasta, Chile.

Formation: not given.

Age: Callovian, Middle Jurassic.

(128) '*Metriorhynchus*' *hastifer* (Eudes-Deslongchamps, 1868d)

Data from: MNHN.F RJN 199 (holotype); Eudes-Deslongchamps (1867–1869); Lepage *et al*. (2008).

Locality: Cap de la Hève (type locality), and Octeville-sur-Mer, Département du Seine-Maritime, Haute-Normandie, France.

Formation: Marnes de Bléville Formation.

Age: *Rasenia cymodoce* Sub-Boreal ammonite Zone, lower Kimmeridgian, Upper Jurassic.

(129) '*Metriorhynchus*' *westermanni* Gasparini, 1980

Data from: Gasparini *et al*. (2008), Fernández *et al*. (2011).

Locality: Placilla de Caracoles (type locality), and Sierra del Medio, Región de Antofagasta, Chile

Formation: Mina Chica Formation (type locality) and Vergara Formation.

Age: Callovian and Oxfordian, Middle and Upper Jurassic.

(130) Mr Leeds’ Dakosaur

Data from: NHMUK PV R 3321, NHMUK PV R 4696, NHMUK PV R 4763.

Locality: Peterborough, UK.

Formation: Peterborough Member, Oxford Clay Formation, Ancholme Group.

Age: middle Callovian, Middle Jurassic.

(131) Mr Passmore’s Specimen

Data from: OUMNH J1583.

Locality: Swindon, Wiltshire, UK.

Formation: upper Kimmeridge Clay Formation.

Age: lower Tithonian, Upper Jurassic.

(132) *Neptunidraco ammoniticus* Cau & Fanti, 2011

Data from: Cau & Fanti (2011).

Locality: unknown, but near Sant’Ambrogio di Valpolicella, Verona, Italty.

Formation: pseudonodular facies of lowermost Rosso Ammonitico Veronese Formation.

Age: *Parkinsonia parkinsoni* ammonite Zone, uppermost Bajocian, Middle Jurassic.

(133) *Plesiosuchus manselii* (Hulke, 1870)

Data from: NHMUK PV OR 40103 and NHMUK PV OR 40103a (holotype), NHMUK PV R 1089, MJML K181, MJML K434.

Localities: Westbury, Wiltshire; and Kimmeridge, Dorset (type locality), England, UK.

Formation: Kimmeridge Clay Formation, Ancholme Group.

Age: *Aulacostephanus* *eudoxus* Sub-Boreal ammonite Zone, upper Kimmeridgian, to *Pectinatites wheatleyensis* Sub-Boreal ammonite Zone, lower Tithonian, Upper Jurassic.

(134) *Purranisaurus potens* Rusconi, 1948

Data from: Herrera et al. (2015); high quality photographs of the holotype by Yanina Herrera.

Locality: Arroyo del Arroyo del Cajón Grande, southwest Malargüe Department, Mendoza Province, Argentina.

Formation: Vaca Muerta Formation, Mendoza Group. Neuquén Basin.

Age: *Substeueroceras koeneni* ammonite Zone, upper Tithonian or lower Berriasian, Upper Jurassic or Lower Cretaceous.

(135) *Suchodus durobrivensis* Lydekker, 1890

Data from: NHMUK PV R 1994 (holotype), NHMUK PV R 2039

Locality: Peterborough, UK.

Formation: Peterborough Member, Oxford Clay Formation, Ancholme Group.

Age: middle Callovian, Middle Jurassic.

(136) *Torvoneustes* *carpenteri* (Wilkinson *et al.*, 2008)

Data from: BRSMG Ce 17365 (holotype), BRSMG Cd 7203; Wilkinson *et al.* (2008), Andrade (2010), Andrade *et al.* (2010).

Locality: Westbury, Wiltshire, England, UK.

Formation: lower Kimmeridge Clay Formation, Ancholme Group.

Age: *Aulacostephanus* *eudoxus* Sub-Boreal ammonite Zone, upper Kimmeridgian, Upper Jurassic.

(137) *Torvoneustes coryphaeus* Young *et al*., 2013b

Data from: MJML K1863 (holotype).

Locality: Swindon, Wiltshire, UK.

Formation: lower Kimmeridge Clay Formation, Ancholme Group.

Age: *Pictonia baylei* Sub-Boreal ammonite Zone, lower Kimmeridgian, Upper Jurassic.

(138) *Torvoneustes* sp. [fragmentary taxon]

Data from: MJML K1707.

Locality: Kimmeridge Bay, Dorset, UK.

Formation: Dorset succession, lower Kimmeridge Clay Formation, Ancholme Group.

Age: *Aulacostephanus* *autossiodorensis* Sub-Boreal ammonite Zone, upper Kimmeridgian, Upper Jurassic.

(139) *Tyrannoneustes lythrodectikos* Young *et al*., 2013a

Data from: GLAHM V972 (holotype), GLAHM V1145, NHMUK PV R 3939, PETMG R176.

Locality: Peterborough, UK.

Formation: Peterborough Member, Oxford Clay Formation, Ancholme Group.

Age: middle Callovian, Middle Jurassic.

(140) Vaches Noire Dakosaur

Data from: MNHN.F RJN 134a, ME 2012.4.68.

Locality: Vaches Noires cliffs, Calvados, France

Formation: Marnes de Villers Formation (possibly also Marnes de Dives Formation).

Age: ?Callovian and Oxfordian, Middle? and Upper Jurassic.

***S2.2) H+Y dataset – character list***

The character list (454 characters) for the Hastings + Young (H+Y) dataset used for one of the phylogenetic analyses herein. The characters are organised into the anatomical order listed in section S1. Comments on the characters and scoring are in italics, and precede the description of states. Osteological craniomandibular and dental characters constitute 73568% (334/454) of the character list, osteological post-cranial characters contribute 25.551% (116/454), while soft-tissue characters contribute 0.881% (4/454).

Characters that are not applicable (i.e. cannot be scored) for all taxa are marked with an asterisk **(*)** following the character description. Characters treated as additive for the ordered-character analysis are denoted by **(ORDERED)** following the character description.

**Abbreviation: ch.**, character; **ds**, dataset.

**Skull geometry and dimensions** (Ch. 1 – 10; 2.203% of characters)

| # | Description |
| --- | --- |
| 1 | **Skull height, in posterior view:**  *Clark (1994, ch. 3 modified); Andrade & Bertini (2008a, ch. 2); Andrade et al. (2011, ch. 1); Ristevski et al. (2018, ds 1, ch. 1); Smith et al. (in review, ds 2, ch. 1); Ősi et al. (in review, ds 1, ch. 1).*  0. skull higher than wide, or subequal  1. skull evidently wider than high |
| 2 | **Skull geometry, relative position of tooth row, quadrate articular facet and occipital condyle:**  *Wu & Sues (1996, ch. 24 modified); Sereno et al. (2003, ch. 46 modified); Pol (2003, ch. 104 modified); Turner & Buckley (2008, ch. 105 modified); Andrade et al. (2011, ch. 2); Ristevski et al. (2018, ds 1, ch. 2); Smith et al. (in review, ds 2, ch. 2); Ősi et al. (in review, ds 1, ch. 2).*  *In its original format, this character assumed that the tooth row was always below the occipital condyle, which is not always true (e.g.,* Pelagosaurus*). The original format was modified by Andrade et al. (2011) because in Mesoeucrocodylia each of its components (height of occipital condyle, quadrate condyle and tooth row) will relate to each other independently, therefore demanding more than the original three states to reflect their geometrical relationships. Note also differences from the original scorings, and also the lack of agreement on the scorings by different authors, for the original format.*  0. tooth row and quadrate condyle aligned, both at a lower level than the occipital condyle  1. tooth row at a lower level than the quadrate condyle, which is aligned to the occipital condyle  2. tooth row quadrate and occipital condyle all aligned in the same plane  3. tooth row and occipital condyle aligned, but quadrate condyle at a slightly lower level  4. tooth row and quadrate condyle unaligned and quadrate at a lower level, but both below the occipital condyle  5. tooth row and quadrate condyle unaligned and tooth row at a lower level, but both below the occipital condyle |
| 3 | **Skull geometry, relative position of tooth row and occipital condyle:**  *Wu & Sues (1996, ch. 24 modified); Sereno et al. (2003, ch. 46 modified); Pol (2003, ch. 104 modified); Turner & Buckley (2008, ch. 105 modified); Andrade et al. (2011, ch. 3); Ristevski et al. (2018, ds 1, ch. 3); Smith et al. (in review, ds 2, ch. 3); Ősi et al. (in review, ds 1, ch. 3).*  0. unaligned, tooth row at a lower level than occipital condyle  1. tooth row and occipital condyle aligned in the same plane |
| 4 | **Skull geometry, relative position of quadrate condyles and occipital condyle:**  *Wu & Sues (1996, ch. 24 modified); Sereno et al. (2003, ch. 46 modified); Pol (2003, ch. 104 modified); Turner & Buckley (2008, ch. 105 modified); Andrade et al. (2011, ch. 4); Young et al. (2016, ds 2, ch. 148), Ristevski et al. (2018, ds 2, ch. 6); Smith et al. (in review, ds 1, ch. 7); Ősi et al. (in review, ds 1, ch. 4).*  *State (1) occurs in Neosuchia (with reversals in marine crocodyliforms, e.g. Dyrosauridae and cf.* Terminonaris robusta*).*  0. unaligned, quadrate condyles are at a lower level than the occipital condyle  1. quadrate condyles and occipital condyle aligned on the same plane |
| 5 | **Skull width to length ratio:**  *Young et al. (2012, ch. 1); Young (2014, ch. 1); Young et al. (2016, ds 2, ch. 1); Ristevski et al. (2018, ds 2, ch. 1); Smith et al. (in review, ds 1, ch. 1); Ősi et al. (in review, ds 1, ch. 5).*  *= maximum width between the lateral-most points of the quadrates : basicranial length*  0. 0.26 or lower  1. between 0.27 and 0.4  2. 0.4 or greater |
| 6 | **Snout elongation:**  *Jouve (2005, ch. 5 modified); Hastings et al. (2010, ch. 1 modified); Ristevski et al. (2018, ds 2, ch. 2); Smith et al. (in review, ds 1, ch.2); Ősi et al. (in review, ds 1, ch. 6).*  *State (2) can only be scored for taxa where snout elongation is the result of the anteroposterior elongation of the maxilla (with the maxillae contact along their medial margins along the dorsal surface).*  *State (1) occurs in most pholidosaurids, most dyrosaurids and gavialoids.*  *State (2) occurs in Thalattosuchia and* Meridiosaurus*.*  0. both the nasals and maxillae not elongated  1. nasals and maxillae both elongated (having the sublongirostrine or longirostrine condition)  2. maxillae elongated, contacting each other along their medial margins. No elongation of the nasals (having the sublongirostrine or longirostrine condition) |
| 7 | **Rostrum, relation between height and width: (ORDERED)**  *Clark (1994, ch. 3 modified); Young (2006, ch. 8 modified); Wilkinson et al. (2008, ch. 25 modified); Young & Andrade (2009, ch. 25 modified); Andrade et al. (2011, ch. 6 modified); Young et al. (2011, ch. 25 modified); Young et al. (2013a, ch. 1 modified); Young et al. (2012, ch. 2 modified); Young (2014, ch. 2 modified); Young et al. (2016, ds 2, ch. 2 modified), Ristevski et al. (2018, ds 2, ch. 3); Smith et al. (in review, ds 1, ch.3); Ősi et al. (in review, ds 1, ch. 7).*  *State (0) does not imply the platyrostral condition, although that is the most likely morphology.*  *State (1) does not imply the rostrum will be tubular, although a tubular rostrum is most likely (1) in proportion.*  *State (2) does not imply the oreinirostral condition, although that is the most likely morphology.*  0. wider than high (lateromedial axis greater than dorsoventral axis, by more than 10%)  1. height and width subequal (lateromedial & dorsoventral axes subequal ±10%)  2. higher than wide (dorsoventral axis greater than lateromedial axis, by more than 10%) |
| 8 | **Rostrum, in dorsal view – amblygnathy (“bullet-shaped”, with the rostrum retaining its width along almost all its length):**  *Young et al. (2012, ch. 3); Young (2014, ch. 3); Young et al. (2016, ds 2, ch. 3), Ristevski et al. (2018, ds 2, ch. 4); Smith et al. (in review, ds 1, ch.4); Ősi et al. (in review, ds 1, ch. 8).*  *State (1) is a putative apomorphy of* Dakosaurus + *Mr Leeds’ Dakosaur.*  0. no  1. yes |
| 9 | **Rostrum, presence of distinct flattening of the cranial rostrum dorsal surface and symphyseal dentary ventral surface:**  *Smith et al. (in review, ds 1, ch. 5); Ősi et al. (in review, ds 1, ch. 9).*  *State (1) occurs in* Sarcosuchus *and* Chalawan.  *This character can be scored based on either the cranial or mandibular rostrum.*  *This character scores the almost planar dentary symphyseal region, and the flattening of the cranial rostrum. Note, this character does not score for the ‘duck’-billed morphology seen in some crocodylomorphs, only the flattening seen in the giant pholidosaurids.*  0. no  1. yes |
| 10 | **Rostrum narrows markedly in dorsal view, immediately in front of the orbits**  *Young et al. (2016, ds 2, ch. 4), Ristevski et al. (2018, ds 2, ch. 5); Smith et al. (in review, ds 1, ch. 6); Ősi et al. (in review, ds 1, ch. 10).*  *In Thalattosuchia, state (1) occurs in* Aeolodon priscus, Mycterosuchus nasutus, Teleosaurus megarhinus *and* Teleosaurus cadomensis. *Note that in many* Steneosaurus bollensis *specimens the dorsoventral compression of the skulls exaggerates the width of the temporal region.*  0. no  1. yes |

**Craniomandibular ornamentation** (Ch. 11 – 16; 1.322% of characters)

| # | Description |
| --- | --- |
| 11 | **Ornamentation (maxilla in dorsal view = external surface):**  *Young & Andrade (2009, ch. 84 modified); Young et al. (2011, ch. 84 modified); Young et al. (2013a, ch. 2 modified); Young et al. (2012, ch. 4 modified); Young (2014, ch. 4); Young et al. (2016, ds 2, ch. 5), Ristevski et al. (2018, ds 2, ch. 7); Smith et al. (in review, ds 1, ch. 8); Ősi et al. (in review, ds 1, ch. 11).*  0. no conspicuous ornamentation, or ornamented with an irregular pattern of ridges, rugosities and anastomosing grooves  1. conspicuous circular-to-polygonally pitted pattern  2. conspicuous grooved-ridged pattern  3. conspicuous pits and grooves |
| 12 | **Ornamentation (frontal):**  *Young (2006, ch. 1 modified); Wilkinson et al. (2008, ch. 1 modified); Young & Andrade (2009, ch. 1 modified); Young et al. (2011, ch. 1 modified); Young et al. (2013a, ch. 46 modified); Young et al. (2012, ch. 55 modified); Young (2014, ch. 57); Young et al. (2016, ds 2, ch. 65), Ristevski et al. (2018, ds 2, ch. 8); Smith et al. (in review, ds 1, ch. 9); Ősi et al. (in review, ds 1, ch. 12).*  *In metriorhynchids, the main body of the frontal can be largely or entirely 'smooth', while the anteromedial process is ornamented. If this process is ornamented, the taxon was still scored from states (0–2).*  0. yes, with shallow to deep elliptical pits and shallow to deep grooves  1. yes, shallow to deep elliptical pits  2. yes, shallow to deep grooves  3. no |
| 13 | **Ornamentation (dorsal surface of the medial temporal region, typically the intertemporal bar):**  *Jouve et al. (2005b, ch. 30 modified); Jouve et al. (2008, ch. 30 modified); Hastings et al. (2010, ch. 8 modified), Ristevski et al. (2018, ds 2, ch. 9 modified); Smith et al. (in review, ds 1, ch. 10 modified); Ősi et al. (in review, ds 1, ch. 13).*  *Note, herein we have re-worded this character to score for ornamentation along the dorsal surface of the medial temporal region, and not the intertemporal bar. This allows taxa that lack supratemporal fenestrae (such as* Iharkutosuchus*) to be scored for this character.*  0. ornamented  1. unornamented |
| 14 | **Ornamentation (parietal in dorsal view):**  *Jouve et al. (2005b, ch. 27 modified); Jouve et al. (2008, ch. 27 modified); Hastings et al. (2010, ch. 45 modified), Ristevski et al. (2018, ds 2, ch. 10); Smith et al. (in review, ds 1, ch. 11); Ősi et al. (in review, ds 1, ch. 14).*  0. no conspicuous ornamentation  1. slight ornamentation  2. strongly ornamented with deep and/or numerous pits |
| 15 | **Sculpturing, palatal surface of maxilla:**  *Ortega et al. (2000, ch. 2); Andrade et al. (2011, ch. 20); Ristevski et al. (2018, ds 1, ch. 20); Smith et al. (in review, ds 2, ch. 20); Ősi et al. (in review, ds 1, ch. 15).*  *State (1) was also registered for* Sichuanosuchus*,* Shantungosuchus *and* Fruitachampsa *by Ortega et al. (2000), but the absence (0) in* Hemiprotosuchus *cannot be confirmed, as the specimen is preserved with mandible in occlusion. Palatal sculpturing is also present in a few notosuchians.*  0. absent, palatal surface smooth  1. present, palatal surface ornamented with ridges |
| 16 | **Sculpturing, presence on the palatal surface of pterygoid:**  *Clark (1994, ch. 40); Andrade et al. (2011, ch. 21); Ristevski et al. (2018, ds 1, ch. 21); Smith et al. (in review, ds 2, ch. 21); Ősi et al. (in review, ds 1, ch. 16).*  *State (1) is present in Protosuchidae.*  0. absent, surface smooth  1. present |

**Internal neuroanatomy, sensory systems and cranial exocrine glands** (Ch. 17; 0.220% of characters)

*[Scoring any OTU for these characters can come from: CT scan datasets or specimens with the cranium broken showing said cavity. All characters in this section refer to internal anatomy, principally internal cavities and structures. Thus are not included in the sections referring to bones visible externally]*

| # | Description |
| --- | --- |
| 17 | **Internal enlarged cephalic exocrine glands, presence:**  *Andrade et al. (2011, ch. 485 modified); Ristevski et al. (2018, ds 2, ch. 386 modified); Smith et al. (in review, ds 1, ch. 392 modified); Ősi et al. (in review, ds 1, ch. 453).*  *The evidence for internal large cephalic exocrine glands is well supported (e.g. Fernández & Gasparini, 2000, 2008; Gandola et al., 2006; Fernández & Herrera, 2009), and interpreted as structures for salt excretion. In fossil specimens, lobulations for glands must show a regular pattern, and have no trabecular bones, which otherwise indicate the presence of pneumatic cells of air sinuses (Fernández & Herrera, 2009).*  *Note that in metriorhynchids the chambers housing these enlarged glands indicate their presence.*  *These enlarged nasal glands are also associated with gland drainage ducts.*  *State (1) occurs in Metriorhynchidae.*  0. absent, nasal glands not enlarged  1. present, nasal glands enlarged (= hypertrophied), being bound externally by the nasal, prefrontal, lachrymal, maxilla and jugal |

**Craniomandibular pneumaticity** (Ch. 18 – 21; 0.881% of characters)

*[Scoring any OTU for these characters can come from: CT scan datasets or specimens with the cranium broken showing said cavity. All characters in this section refer to internal pneumatic cavities or the enclosure of pneumatic structures by bone. Thus are not included in the sections referring to bones visible externally]*

| # | Description |
| --- | --- |
| 18 | **Supraoccipital, internal presence of the cavity for the intertympantic diverticulum of the pharyngotympanic sinus system (= the “mastoid antrum”):**  *Clark (1994, ch. 63 modified);* *Andrade et al. (2011, ch. 282 modified); Ristevski et al. (2018, ds 2, ch. 165); Smith et al. (in review, ds 1, ch. 169); Ősi et al. (in review, ds 1, ch. 186).*  *As discussed by Wilberg (2015b), this character has been scored to unite Pholidosauridae and Dyrosauridae with Thalattosuchia. The natural external and internal mould* Pholidosaurus schaumburgensis *Bückeburg specimens held in Berlin show the cavity for this diverticulum (also see Wilberg, 2015b Figure 7c).*  *Scoring any OTU as state (1) can come from CT scan datasets, or fossil specimens with a broken supraoccipital that show the cavity. However, scoring an OTU can only reliably come from CT scan datasets, or acid prepared specimens that have the braincase preserved. While this limits the number of OTUs that can be scored, it helps prevent potential mis-scorings.*  *Here* Dyrosaurus*,* Sarcosuchus *and* Terminonaris *are scored as (?) until CT scans conclusively show the lack of this diverticulum.*  *State (0) occurs in Thalattosuchia.*  0. absent (in Thalattosuchia this diverticulum is lost)  1. present |
| 19 | **Quadrate, openings on the dorsal surface at the proximal end (= subtympanic foramina; = quadrate fenestrae):**  *Young & Andrade (2009, ch. 158 modified); Young et al. (2011, ch. 158 modified); Young et al. (2013a, ch. 104 modified); Young et al. (2012, ch. 121 modified); Young (2014, ch. 124 modified); Young et al. (2016, ds 2, ch. 145 modified); Ristevski et al. (2018, ds 2, ch. 198); Smith et al. (in review, ds 1, ch. 202); Ősi et al. (in review, ds 1, ch. 220).*  *This character scores the presence of foramina on the proximal quadrate for the infundibular diverticula of the pharyngotympanic sinus system contacting the tympanum.*  *State (2) occurs in Thalattosuchia.*  0. multiple subtympanic foramina  1. single subtympanic foramen  2. lacks subtympanic foramina |
| 20 | **Quadrate (and articular), foramina aërum presence:**  *Ristevski et al. (2018, ds 2, ch. 199); Smith et al. (in review, ds 1, ch. 203); Ősi et al. (in review, ds 1, ch. 221).*  *This character scores the presences of the aërum foramina on the dorsal or mediodorsal surface of the distal quadrate, and the associated opening on the dorsal or medial surface of the retroarticular process of the mandible. These foramina are for the siphonium connecting the quadrate and articular diverticula of the pharyngotympanic sinus system.*  *Note that in large adults the articular diverticula can completely regress, thus the quadrate aërum foramen may be the best indicator of the structure's presence.*  *Following Nesbitt (2011; discussion on ch. 159), basal crocodylomorphs (i.e. ‘sphenosuchians’) the large medial articular foramina are not considered to be articular aërum foramina. Whether basal crocodylomorphs had articular diverticula is currently unknown.*  *State (0) occurs in Thalattosuchia (basal crocodylomorphs are scored as ‘?’).*  *State (1) is currently only known to occur in Crocodyliformes.*  0. absent  1. present |
| 21 | **Medial pharyngeal and pharyngotympanic tubes (= “Eustachian tubes”), relation to basioccipital and basisphenoid: (ORDERED)**  *Clark (1994, ch. 52 modified); Andrade et al. (2011, ch. 290 modified); Nesbitt (2011, ch. 121 – based on Gower 2002, ch. 13); Young et al. (2013a, ch. 108); Young et al. (2012, ch. 126); Young (2014, ch. 130); Young et al. (2016, ds 2, ch. 152); Ristevski et al. (2018, ds 2, ch. 206); Smith et al. (in review, ds 1, ch. 210); Ősi et al. (in review, ds 1, ch. 228)*.  *State (1) occurs in* Postosuchus *and ‘sphenosuchians’.*  *State (2) occurs in Crocodyliformes.*  0. not enclosed by bone  1. partially enclosed between the basioccipital and basisphenoid  2. entirely enclosed between the basioccipital and basisphenoid |

**Rostral neurovascular foramina** (Ch. 22 – 27; 1.322% of characters)

| # | Description |
| --- | --- |
| 22 | **Neurovascular foramina, presence of an expanded network of openings on the dorsal surface of the rostrum and ventral-lateral surfaces of the mandible:**  *Andrade et al. (2011, ch. 22), Ristevski et al. (2018, ds 2, ch. 11); Smith et al. (in review, ds 1, ch. 12); Ősi et al. (in review, ds 1, ch. 17).*  *Based on the data by Soares (2002), where neurovascular foramina are related to the presence of dome pressure receptors (DPR).*  *Teleosauroids score as state (1), even though usually only basal single line of foramina is evident on the maxillae. In all thalattosuchians the dentary foramina are greater in number, and are easier to score for. In teleosauroids with no/little premaxillary/maxillary ornamentation, the accessory foramina are visible on the premaxilla and on the anterior maxillae. In Machimosaurini these foramina are much more numerous, and therefore easier to identify.*  *Metriorhynchids however clearly have accessory foramina on the premaxillae, maxillae and dentaries, although they do not have the ‘beehive-like’ arrangement mentioned for extant taxa. The maxillary foramina can be observed across the element, and are not restricted to the anterior maxilla as in teleosauorids.* Pelagosaurus typus *has clear accessory foramina on the premaxillae and anterior dentaries, and is here scored as (1).*  *It is unclear whether the thalattosuchian condition is homologous to that seen in neosuchians.*  *This character might need to be re-evaluated, as George & Holliday (2013) have questioned the utility of using facial neurovascular foramina as osteological correlates for the DPR system.*  0. absent, neurovascular openings limited to a single line, near the ventral margin of the rostrum and dorsal margin of dentary  1. present at least at the premaxillae, maxillae and dentaries |
| 23 | **Neurovascular foramina (premaxilla), overall distance to the alveolar margin and teeth:**  *Andrade & Bertini (2008, ch. 17 part); Andrade et al. (2011, ch. 23); Ristevski et al. (2018, ds 1, ch. 23); Smith et al. (in review, ds 2, ch. 23); Ősi et al. (in review, ds 1, ch. 18).*  *Note that Andrade et al. (2011) substantially re-scored this character from the original (Andrade & Bertini 2008, ch17), and that complementary characters on neurovascular foramina are present.*  0. ventral-most foramina reach area next to the alveolar margin, close to teeth  1. ventral-most foramina clearly apart from the alveolar margin, distant to the teeth |
| 24 | **Neurovascular foramina (anterior maxilla), overall distance to the alveolar margin and teeth:**  *Andrade & Bertini (2008, ch. 17 part); Andrade et al. (2011, ch. 24); Ristevski et al. (2018, ds 1, ch. 24); Smith et al. (in review, ds 2, ch. 24); Ősi et al. (in review, ds 1, ch. 19).*  *State (0) is putative apomorphy of derived eusuchians, but is also present in other mesoeucrocodylian clades.*  *State (1) is a common condition in Crocodylomorpha, occurring even in basal eusuchians.*  0. ventral-most foramina reach area next to the alveolar margin, close to teeth  1. ventral-most foramina clearly apart from the alveolar margin, distant to the teeth |
| 25 | **Neurovascular foramina (mid maxilla) forming a strongly arched line at mid-rostrum, at maturity:**  *Andrade et al. (2011, ch. 25); Ristevski et al. (2018, ds 1, ch. 25); Smith et al. (in review, ds 2, ch. 25); Ősi et al. (in review, ds 1, ch. 20).*  *State (1) is putative apomorphy of* Araripesuchus  0. absent, line of foramina follows the overall outline of the margin  1. present, ample area of smooth margin ventral to the arched line of foramina |
| 26 | **Neurovascular foramina (posterior maxilla), distribution on the alveolar margin:**  *Andrade et al. (2011, ch. 26); Young et al. (2016, ds 2, ch. 26), Ristevski et al. (2018, ds 2, ch. 12); Smith et al. (in review, ds 1, ch. 13); Ősi et al. (in review, ds 1, ch. 21).*  *State (1) occurs in goniopholidids*  0. ventral-most foramina not high on the maxillary margin, either close or next to the alveoli  1. ventral-most foramina high on the maxilla (up to twice the distance from other foramina), very distant to the alveoli |
| 27 | **Neurovascular foramina (dentary), distribution of neurovascular foramina relative to the alveolar margin, in non-tubular snouted forms: (*)**  *Andrade et al. (2011, ch. 27); Ristevski et al. (2018, ds 1, ch. 27); Smith et al. (in review, ds 2, ch. 27); Ősi et al. (in review, ds 1, ch. 22).*  *This character is not applicable for taxa that have tubular snouts.*  *State (1) occurs in Crocodylia.*  0. foramina form a simple straight to ventrally-arched line  1. foramina form a sinusoid line, following the dorsal fluttings, when fluttings are present |

**Cranial rostrum** (Ch. 28 – 85; 12.775% of characters)

*[external nares, dermatocranial bones (= os præmaxillare, ossa nasalia, os maxillare and ossa lacrimalia), antorbital cavity]*

| # | Description |
| --- | --- |
| 28 | **Perinarial crests, presence and morphology:**  *Andrade et al. (2011, ch. 29); Ristevski et al. (2018, ds 1, ch. 29); Smith et al. (in review, ds 2, ch. 29); Ősi et al. (in review, ds 1, ch. 23).*  *State (1) is present within Goniopholididae (*Anteophthalmosuchus, Hulkeopholis, Goniopholis *and* Amphicotylus*).*  0. absent, surface even or bearing a perinarial fossa  1. present as well defined and distinct ridges, cornering the lateral to posterior borders of the naris |
| 29 | **External nares orientation:**  *Turner & Pritchard (2015, ch. 6; modified from Clark 1994, ch. 6); Young et al. (2016, ds 2, ch. 8), Ristevski et al. (2018, ds 2, ch. 14); Smith et al. (in review, ds 1, ch. 15); Ősi et al. (in review, ds 1, ch. 24).*  *In Thalattosuchia, state (0) occurs in the teleosauroids* Mycterosuchus nasutus, *the Chinese teleosauroid referred to* Peipehsuchus teleorhinus*,* Platysuchus multiscrobiculatus*,* Steneosaurus brevior *and* Teleosaurus megarhinus.  0. orientated anteriorly, anterodorsally, or anterolaterally  1. orientated mainly dorsally, or dorsolaterally |
| 30 | **External nares, shape in dorsal view:**  *Young (2006, ch. 6 modified); Wilkinson et al. (2008, ch. 23 modified); Young & Andrade (2009, ch. 23 modified); Young et al. (2011, ch. 23 modified); Young et al. (2013a, ch. 4 modified); Young et al. (2012, ch. 6 modified); Young (2014, ch. 6 modified); Young et al. (2016, ds 2, ch. 9), Ristevski et al. (2018, ds 2, ch. 15); Smith et al. (in review, ds 1, ch. 16); Ősi et al. (in review, ds 1, ch. 25).*  *State (4) is a putative apomorphy of Susisuchidae.*  0. subcircular (diameter in any direction does not vary by more than ± 10%)  1. oval (dorsal width >10% longer than anteroposterior length)  2. ‘D-shaped’, with posterior edge straight  3. spoon-shaped elongate ellipse (dorsal width <40% of anteroposterior length)  4. pear-shaped  5. external nares not exposed in dorsal view |
| 31 | **External nares, shape in anterior view: (NEW)**  *State (1) occurs in* Aeolodon priscus*,* Bathysuchus megarhinus*,* Steneosaurus brevior*, and the Chinese teleosauroid.*  0. subcircular or ‘B-shaped’ (the anterior margin is relatively straight)  1. noticabely ‘8-shaped’ |
| 32 | **Medial tubercles of external nares on the posterior margin:**  *Hastings et al. (2010, ch. 2 modified), Ristevski et al. (2018, ds 2, ch. 16); Smith et al. (in review, ds 1, ch. 17); Ősi et al. (in review, ds 1, ch. 26).*  *States (1+2) are putative apomorphies of Dyrosauridae.*  0. absent  1. dorsal  2. ventral |
| 33 | **Thickness of the anterior margin of the external nares: (*)**  *Hastings et al. (2010, ch. 3 modified), Ristevski et al. (2018, ds 2, ch. 17); Smith et al. (in review, ds 1, ch. 18); Ősi et al. (in review, ds 1, ch. 27).*  *State (1) occurs in basal dyrosaurids.*  *This character is not applicable for taxa that have posterodorsally retracted external nares (i.e. rhacheosaurin metriorhynchids).*  0. less than half anteroposterior length  1. greater than half anteroposterior length, or in species with a broad snout the anterior premaxilla is noticeably thick with the external nares posterior to the P1 alveoli |
| 34 | **External nares, posterodorsal retraction in relation to the tooth-row: (ORDERED)**  *Young (2006, ch. 16 modified); Wilkinson et al. (2008, ch. 38 modified); Young & Andrade (2009, ch. 38 modified); Young et al. (2011, ch. 38 modified); Young et al. (2013a, ch. 5 modified); Young et al. (2012, ch. 7 modified); Young (2014, ch. 7 modified); Young et al. (2016, ds 2, ch. 10), Ristevski et al. (2018, ds 2, ch. 18); Smith et al. (in review, ds1, ch. 19); Ősi et al. (in review, ds 1, ch. 28).*  *This character was designed to quantify the degree of posterodorsal retraction of the external nares in Metriorhynchidae. Its level relative to the tooth-row is used in this regard.*  *Previous states (4–6) of this character were removed by Young et al. (2016) as the maxillary tooth count is too variable.*  0. at the tip of the snout, with its posterior-margin not exceeding the first premaxillary alveolus  1. at the tip of the snout, but its posterior-margin does exceed the last premaxillary alveolus  2. the posterior-margin reaches to the beginning of the 1st maxillary alveolus  3. posterodorsally displaced, anterior-margin begins posterior to the 1st premaxillary alveolus while the posterior-margin exceeds the beginning of the 1st maxillary alveolus |
| 35 | **Perinarial crests, presence and morphology:**  *Andrade et al. (2011, ch. 29), Ristevski et al. (2018, ds 2, ch. 19); Smith et al. (in review, ds 1, ch. 20); Ősi et al. (in review, ds 1, ch. 29).*  *State (1) is present within Goniopholididae (*Anteophthalmosuchus, Goniopholis *and* Amphicotylus*).*  0. absent, surface even or bearing a perinarial fossa  1. present as well defined and distinct ridges, cornering the lateral to posterior borders of the naris |
| 36 | **Intranarial fossa, presence at the lateral walls, inside narial cavity, at the vestibulum:**  *Andrade et al. (2011, ch. 42), Ristevski et al. (2018, ds 2, ch. 20); Smith et al. (in review, ds 1, ch. 21); Ősi et al. (in review, ds 1, ch. 30).*  *State (1) is putative apomorphy of Thalattosuchia.*  *The internarial fossa is an additional chamber that creates an internal border of the external naris; must not be mistaken with the naso-oral fossa, or with the perinarial fossa.*  *Note, unlike Andrade et al. (2011), we consider this to present in all thalattosuchians. A distinct fossa within the nasal cavity is seen in all teleosauroids and* Pelagosaurus typus*, however due to dorsoventral crushing the fossa can be obscured.*  0. absent  1. present |
| 37 | **Premaxilla, dorsal/anterodorsal projection of the anterodorsal margin (anterior to the external nares):**  *Young et al. (2016, ds 2, ch. 11), Ristevski et al. (2018, ds 2, ch. 21); Smith et al. (in review, ds 1, ch. 22); Ősi et al. (in review, ds 1, ch. 31).*  *State (1) occurs in derived pholidosaurids*, *as well as* *in eusuchians.*  0. present  1. absent |
| 38 | **Premaxilla, lateral expansion anterior to the premaxilla-maxilla suture due to the enlargement of the P3 alveoli, with a constriction immediately posterior to the expansion:**  *Hastings et al. (2010, ch. 14 modified), Ristevski et al. (2018, ds 2, ch. 22); Smith et al. (in review, ds 1, ch. 23); Ősi et al. (in review, ds 1, ch. 33).*  *State (1) occurs in basal dyrosaurids.*  *Note that unlike other lateral expansions of the premaxilla, this does not correlate with a lateral expansion of the dentary.*  0. absent  1. present |
| 39 | **Premaxilla, length compared to width: (*)**  *Jouve et al. (2008, ch. 41 modified); Hastings et al. (2010, ch. 22 modified), Ristevski et al. (2018, ds 2, ch. 23); Smith et al. (in review, ds 1, ch. 24); Ősi et al. (in review, ds 1, ch. 34).*  *State (1) occurs in derived dyrosaurids.*  *This character is not applicable for taxa that have posterodorsally retracted external nares.*  0. slightly longer than wide  1. nearly three times longer than wide, or more than three times longer than wide |
| 40 | **Premaxilla, ventral surface, presence of large depressions/notches for reception of the D1 teeth:**  *Ristevski et al. (2018, ds 2, ch. 24); Smith et al. (in review, ds 1, ch. 25); Ősi et al. (in review, ds 1, ch. 35).*  *State (1)* *occurs in the pholidosaurids* Terminonaris, Meridiosaurus, Sarcosuchus *and* Oceanosuchus, *goniopholididids* Anteophthalmosuchus sp., Amphicotylus stovalli *and* Calsoyasuchus, *and* *basal* *dyrosaurids* (e.g. Cerrejonisuchus).  *State (2) occurs in* Elosuchus cherifiensis *and* E. broinae.  0. absent  1. occurs posterior to either the P1–P2 (or just the P2) alveoli, and are ventral to the external nares  2. occurs between, and separates, the P1–P2 alveoli from the P3–P4 alveoli |
| 41 | **Premaxilla, when seen in lateral view: (ORDERED)**  *Young et al. (2016, ds 2, ch. 13 modified), Ristevski et al. (2018, ds 2, ch. 26); Smith et al. (in review, ds 1, ch. 27); Ősi et al. (in review, ds 1, ch. 36).*  *This character scores the ‘pholidosaurid beak’. However,* Meridiosaurus *does not have a fully sub-vertical ‘beak’, but do have an intermediate morphology. This morphology is herein considered homologous to the ventral alveolar row of goniopholidids and basal dyrosaurids.*  *This character is not applicable for those with a strongly orientated anteroventral premaxilla (e.g. in teleosauroids).*  *State (1) occurs in* Meridiosaurus, Elosuchus, *and the French* Pholidosaurus, *and in the goniopholidids* Anteophthalmosuchus sp., Amphicotylus stovalli *and* Goniopholis kiplingi, *and the basal dyrosaurid* Cerrejonisuchus.  *State (2) occurs in the pholidosaurids* Chalawan, Sarcosuchus, Terminonaris *and* Oceanosuchus,  0. the anterior and anterolateral margins are not sub-vertical, and do not extend ventrally when compared to the rest of the premaxilla (i.e. the dentigerous margins)  1. the anterior and anterolateral margins are slightly sub-vertical, and slightly extend ventrally to the rest of the element  2. the anterior and anterolateral margins are fully sub-vertical and extend ventrally to the rest of the element |
| 42 | **Premaxilla, when seen in lateral view:**  *Ristevski et al. (2018, ds 2, ch. 27); Smith et al. (in review, ds 1, ch. 28); Ősi et al. (in review, ds 1, ch. 37).*  *State (1) occurs in the teleosauroids* Mycterosuchus nasutus, *the Chinese teleosauroid referred to* Peipehsuchus teleorhinus*,* Platysuchus multiscrobiculatus*,* Steneosaurus brevior *and* Teleosaurus megarhinus.  *This character is not homologous to the pholidosaurid ventral verticalisation of the premaxilla, as in this sub-set of teleosauroids the premaxilla is strongly orientated anteroventrally in lateral view.*  *This character is not applicable for those with the ventral verticalisation of the premaxilla (e.g. in pholidosaurids).*  0. the anterior and anterolateral margins are either not sub-vertical, or do not extend ventrally when compared to the rest of the premaxilla (i.e. the dentigerous margins)  1. the anterior and anterolateral margins are orientated anteroventrally and extend ventrally to the rest of the element. |
| 43 | **Premaxilla, proportion of total length posterior to the external nares:**  *Wilkinson et al. (2008, ch. 21); Young & Andrade (2009, ch. 21); Young et al. (2011, ch. 21); Young et al. (2013a, ch. 6); Young et al. (2012, ch. 8); Young (2014, ch. 8); Young et al. (2016, ds 2, ch. 14) Ristevski et al. (2018, ds 2, ch. 28); Smith et al. (in review, ds 1, ch. 29); Ősi et al. (in review, ds 1, ch. 38).*  0. greater than 67% of premaxilla total length is posterior to the external nares  1. between 50–65%  2. between 36–45%  3. 28% or less |
| 44 | **Premaxilla, posterodorsal (= maxillary, = subnarial) process, termination:**  *Nesbitt & Desojo (2017, ch. 415); Ősi et al. (in review, ds 1, ch. 39).*  *State (1) occurs in Crocodylomorpha.*  0. anterior to or at the posterior end of the external naris  1. posterior of the posterior extension of the external naris |
| 45 | **Premaxilla, posterodorsal process: (*)**  *Young (2014, ch. 9); Young et al. (2016, ds 2, ch. 15); Ristevski et al. (2018, ds 2, ch. 29); Smith et al. (in review, ds 1, ch. 30); Ősi et al. (in review, ds 1, ch. 40).*  *State (1) occurs in* Tyrannoneustes lythrodectikos, Torvoneustes, 'Metriorhynchus' hastifer *and* *Mr Passmore's specimen.*  *This character is not applicable for taxa that retract their external nares (i.e. rhacheosaurin metriorhynchids).*  0. short, terminates level to the fourth maxillary alveolus, or more anteriorly  1. long, terminates level to the end of the fourth maxillary alveolus, or more posteriorly |
| 46 | **Premaxilla, development of premaxillary septum:**  *Young (2006, ch. 7 modified); Wilkinson et al. (2008, ch. 24 modified); Young & Andrade (2009, ch. 24 modified); Young et al. (2011, ch. 24 modified); Young et al. (2013a, ch. 7); Young et al. (2012, ch. 9); Young (2014, ch. 10); Young et al. (2016, ds 2, ch. 16); Ristevski et al. (2018, ds 2, ch. 30); Smith et al. (in review, ds 1, ch. 31); Ősi et al. (in review, ds 1, ch. 41).*  *State (1) scores the premaxillary septum of Metriorhynchidae.*  *Terminonaris currently scored as ‘?’, as it is unclear whether there was also a separating septum present.*  *Young et al. (2013a) changed this character from a multi-state to its present binary form.*  *Currently, only Rhacheosaurini metriorhynchids are definitively known to have had a full premaxillary septum, however specimens of* Metriorhynchus superciliosus*, ‘*M*.’* brachyrhynchus, *Mr Passmore’s Specimen and* Tyrannoneustes lythrodectikos *have preserved: the proximal end of the bar, and the raised distal articulation region on the premaxilla associated with the anterior end of the bar in Rhacheosaurini.Thus, they have been scored as (1). It is possible that only Rhacheosaurini has a fully ossified premaxillary bar, or the incomplete bar could be due to post-mortem damage.*  *It is not homologous with other crocodylomorph septa, which are either partially formed by the nasals, or do not originate on the external surface of the premaxilla immediately anterior to the nasal fossa.*  0. no septum, with a single undivided external naris, or a divided external naris not formed solely by a premaxillary septum  1. external nares dorsally divided by a midline premaxillary septum |
| 47 | **Rostrum, morphology of the external surface of premaxilla and maxilla:**  *based on Pol (1999, ch. 153); Andrade et al. (2011, ch. 55); Ristevski et al. (2018, ds 2, ch. 31); Smith et al. (in review, ds 1, ch. 32); Ősi et al. (in review, ds 1, ch. 42).*  *State (1) is putative apomorphy of Notosuchidae + Sphagesauridae.*  *Most commonly in state (1), the ventral plane will face laterally and slightly ventrally; the dorsal plane will face laterodorsally.*  0. rostrum with a continuous surface, either convex or plain  1. rostrum with distinct ventral and dorsal surfaces, plain and separated by a somewhat distinct anteroposterior ridge or edge |
| 48 | **Rostrum, type of contriction at the premaxilla-maxilla suture:**  *Clark (1994, ch. 9 modified);* *Wilkinson et al. (2008, ch. 20 modified); Young & Andrade (2009, ch. 20 modified); Andrade et al. (2011, ch. 57); Young et al. (2011, ch. 20 modified); Young et al. (2013a, ch. 75 modified); Young et al. (2012, ch. 88 modified); Young (2014, ch. 90 modified); Young et al. (2016, ds 2, ch. 108 modified); Ristevski et al. (2018, ds 2, ch. 32); Smith et al. (in review, ds 1, ch. 33); Ősi et al. (in review, ds 1, ch. 43).*  *State (0) is a putative apomorphy of* Araripesuchus*.*  *The vast majority of crocodylomorphs can be considered as (1), but highly predaceous forms will show a well-defined notch at the premaxilla-maxilla suture (2).*  0. narrow slit  1. wide, poorly-defined concavity, or not constricted at all  2. well-defined notch |
| 49 | **Premaxillae anterior to naris, morphology:**  *Clark (1995, ch. 5 modified);* *Andrade et al. (2011, ch. 62); Ristevski et al. (2018, ds 2, ch. 33); Smith et al. (in review, ds 1, ch. 34); Ősi et al. (in review, ds 1, ch. 44).*  *State (0) is putative apomorphy of Notosuchidae + Sphagesauridae.*  *State (1) is a putative apomorphy of* Araripesuchus *+* Libycosuchus*.*  0. anterior rami of premaxillae do not meet medially, anterior/ventral to naris, with both premaxillae in contact only through palatine rami  1. anterior rami of premaxillae meet anterior to naris, through a very narrow band, but not projecting vertically  2. anterior rami of premaxillae broadly meet anterior to naris, forming a vertical wall, which may be straight or slightly convex |
| 50 | **Premaxilla, type of contact with maxilla:**  *Clark (1994, ch. 8); Andrade et al. (2011, ch. 63); Ristevski et al. (2018, ds 2, ch. 34); Smith et al. (in review, ds 1, ch. 35); Ősi et al. (in review, ds 1, ch. 45).*  *State (1) is a putative apomorphy of Crocodyliformes.*  0. premaxilla loosely overlies maxilla on face  1. premaxilla and maxilla suture together along butt joint |
| 51 | **Distance between premaxilla and nasal:**  *Young (2006, ch. 5 modified); Wilkinson et al. (2008, ch. 22 modified); Young & Andrade (2009, ch. 22 modified); Young et al. (2011, ch. 22 modified); Young et al. (2013a, ch. 8 modified); Young et al. (2012, ch. 10); Young (2014, ch. 11); Young et al. (2016, ds 2, ch. 17); Ristevski et al. (2018, ds 2, ch. 35); Smith et al. (in review, ds 1, ch. 36); Ősi et al. (in review, ds 1, ch. 46).*  *State (2) occurs in* Meridiosaurus *and* Gavialis.  *States (1+2) are putative apomorphies of Thalattosuchia. But with reversals, some specimens of* ‘Metriorhynchus’ brachyrhynchus *have contact between these elements, and the posterodorsal retraction of the external nares in* ‘Cricosaurus’ macrospondylus *results in contact between these elements.*  0. none, premaxilla and nasal contact  1. small, less than half the midline length of the premaxilla  2. large, approximately 80% to more than 100% of the midline length of the premaxilla |
| 52 | **Nasal contribution to the margin of the external nares:**  *Young et al. (2012, ch. 11); Young (2014, ch. 12); Young et al. (2016, ds 2, ch. 18); Ristevski et al. (2018, ds 2, ch. 36); Smith et al. (in review, ds 1, ch. 37); Ősi et al. (in review, ds 1, ch. 47).*  0. present  1. absent |
| 53 | **Anterior process of the nasals, anterior margin relative to the first maxillary alveoli: (*)**  *Jouve et al. (2008, ch. 42 modified); Hastings et al. (2010, ch. 33 modified); Ristevski et al. (2018, ds 2, ch. 37); Smith et al. (in review, ds 1, ch. 38); Ősi et al. (in review, ds 1, ch. 48).*  *State (0) occurs in pholidosaurids and derived dyrosaurids.*  *Note that this character scores the posterior-ward position of the anterior margin of the nasal anterior process, due to the elongation of the premaxillary posterior process only.*  *This character is not applicable for taxa that: 1) have posterodorsally retracted external nares (e.g. Rhacheosaurini), 2) lack a midline premaxillary posterior process (e.g.* Iharkutosuchus*) or 3) have the maxillae elongated and contacting along their midline (e.g. Thalattosuchia).*  0. posterior  1. anterior |
| 54 | **Nasals, morphology in dorsal view: (ORDERED)**  *Andrade & Bertini (2008a, ch. 21); Young & Andrade (2009, ch. 160 modified);* *Andrade et al. (2011, ch. 73); Young et al. (2011, ch. 160 modified); Young et al. (2013a, ch. 9 modified); Young et al. (2012, ch. 12 modified); Young (2014, ch. 13 modified); Young et al. (2016, ds 2, ch. 19 modified); Ristevski et al. (2018, ds 2, ch. 38); Smith et al. (in review, ds 1, ch. 39); Ősi et al. (in review, ds 1, ch. 49).*  *State (0) is a putative apomorphy of both Thalattosuchia and Notosuchia.*  *State (2) is present in* Simosuchus*.*  0. triangular, lateral margins strongly confluent anteriorly  1. rectangular or subrectangular, lateral margins mostly parallel, or lateral margins poorly confluent anteriorly  2. triangular, lateral margins diverging anteriorly |
| 55 | **Nasal, lateroposterior** **processes:**  *Young (2014, ch. 14); Young et al. (2016, ds 2, ch. 20); Ristevski et al. (2018, ds 2, ch. 39); Smith et al. (in review, ds 1, ch. 40); Ősi et al. (in review, ds 1, ch. 50).*  *State (1) is a putative apomorphy of Metriorhynchidae.*  *These processes suture with the anteroventral and anterior margin of the prefrontal, and the posterodorsal margin of the lachrymal.*  0. absent  1. present |
| 56 | **Nasals, fusion at maturity:**  *Gasparini et al. (2006, ch. 257); Sereno & Larsson (2009, ch. 10);* *Hastings et al. (2010, ch. 32 modified);*  *Andrade et al. (2011, ch. 77); Tennant et al. (2016, ch. 65); Ristevski et al. (2018, ds 2, ch. 40); Smith et al. (in review, ds 1, ch. 41); Ősi et al. (in review, ds 1, ch. 51).*  *State (1) is putative apomorphy of Dyrosauridae, but with some species having individuals with fused and unfused nasals, and some specimens with only the anterior nasals fused. Due to this variability, the character from Hastings et al. (2010) has been changed from an ordered multistate into the current binary character.*  *In Thalattosuchia state (1) also occurs in* Lemmysuchus obtusidens*. As in Dyrosauridae, some individuals have fused nasals, while specimens have partially fused nasals. It is currently unclear whether the variation is ontogenetic or individual.*  *State (1) is also present in Mahajangasuchidae.*  0. absent, nasals unfused  1. present, nasals at least partially fused (note that some species have variability in this character, such as in dyrosaurids) |
| 57 | **Nasals, posterior portion at the midline:**  *Nesbitt (2011, ch. 34); Young et al. (2013a, ch. 10 modified); Young et al. (2012, ch. 13 modified); Young (2014, ch. 15 modified); Young et al. (2016, ds 2, ch. 21); Ristevski et al. (2018, ds 2, ch. 41); Smith et al. (in review, ds 1, ch. 42); Ősi et al. (in review, ds 1, ch. 52).*  *This character tests the homology of the metriorhynchoid and (most) teleosauroid "midline trench" and "depression" features, with a similar depression (state 1) seen in "rauisuchians" and "sphenosuchians".*  *The morphology of* Calsoyasuchus *might be distinct, as it has two raised ridges running parallel, at either side of the midline depression, beginning on the frontal.*  *Note that in some ‘sphenosuchians’ (i.e.* Sphenosuchus *and* Junggarsuchus*) the raised frontal ridge can continue onto the posterior nasal, and result in this depression forming around it.*  0. lacks a midline concavity or 'midline trench' - nasals are flat or convex  1. has a concavity at the midline, or a 'midline trench' |
| 58 | **Nasal contact with the prefrontal, in dorsal view: (*)**  *Young & Andrade (2009, ch. 92); Young et al. (2011, ch. 92); Young et al. (2013a, ch. 11); Young et al. (2012, ch. 14); Young (2014, ch. 16); Young et al. (2016, ds 2, ch. 22); Ristevski et al. (2018, ds 2, ch. 42); Smith et al. (in review, ds 1, ch. 43); Ősi et al. (in review, ds 1, ch. 53).*  *This character is not applicable for taxa that lack a sutural contact between the nasals and the prefrontals.*  *State (1) is a putative apomorphy of the* Cricosaurus araucanensis*.*  0. irregular  1. smooth curve with a concavity directed posterolaterally |
| 59 | **Nasal-prefrontal contact:**  *Young et al. (2012, ch. 15); Young (2014, ch. 17); Young et al. (2016, ds 2, ch. 23); Ristevski et al. (2018, ds 2, ch. 43); Smith et al. (in review, ds 1, ch. 44); Ősi et al. (in review, ds 1, ch. 54).*  *State (1) occurs in crocodylomorphs.*  0. absent  1. present |
| 60 | **Premaxilla–maxilla lateral fossa excavating alveolus of last premaxillary tooth:**  *Young & Andrade (2009, ch. 163); Young et al. (2011, ch. 163); Young et al. (2013a, ch. 12); Young et al. (2012, ch. 16); Young (2014, ch. 18); Young et al. (2016, ds 2, ch. 24); Ristevski et al. (2018, ds 2, ch. 44); Smith et al. (in review, ds 1, ch. 45); Ősi et al. (in review, ds 1, ch. 55).*  0. no  1. yes |
| 61 | **Maxilla, ventrolateral edge:**  *Young & Andrade (2009, ch. 115); Young et al. (2011, ch. 115); Young et al. (2013a, ch. 13); Young et al. (2012, ch. 17); Young (2014, ch. 19); Young et al. (2016, ds 2, ch. 25); Ristevski et al. (2018, ds 2, ch. 45); Smith et al. (in review, ds 1, ch. 46); Ősi et al. (in review, ds 1, ch. 56).*  0. straight  1. single convexity  2. double convexity (‘festooned’) |
| 62 | **Position of the posterior-most maxillae: (ORDERED)**  *Hastings et al. (2010, ch. 29 modified); Ristevski et al. (2018, ds 2, ch. 46); Smith et al. (in review, ds 1, ch. 47); Ősi et al. (in review, ds 1, ch. 57).*  *State (1+2) are putative apomorphies of Dyrosauridae.*  *State (2) is a putative apomorphy of* Cerrejonisuchus.  0. anterior to, or even with, the postorbital bars  1. even with the anteroposterior midlength of the supratemporal fenestrae  2. even with, or posterior to, the posterior margins of the supratemporal fenestrae |
| 63 | **Maxilla/jugal, presence of enlarged foramina and associated fossae on the lateral margin of the posterior maxillae and/or the anterior process of the jugal. These foramina are positioned near the maxillojugal suture. These structures are anteroposteriorly aligned (note that the foramina and associated fossae are not always contiguous):** *Ristevski et al. (2018, ds 2, ch. 47); Smith et al. (in review, ds 1, ch. 48); Ősi et al. (in review, ds 1, ch. 58).*  *State (1) occurs in goniopholidids and most tethysuchians (in dyrosaurids the foramen is only present on the jugal). Note that the anterior position of the ‘maxillary depressions’ in* Calsoyasuchus *are not consistent with this character.*  0. absent  1. present |
| 64 | **Posterior maxilla, presence of lateral fossa/fossae next to the alveolar margin, anterior to the jugal and ventral to the lachrymal:**  *Young & Andrade (2009, ch. 135 modified); Andrade et al. (2011, ch. 87 modified); Young et al. (2011, ch. 135 modified); Young et al. (2013a, ch. 14 modified); Young et al. (2012, ch. 18 modified); Young (2014, ch. 20 modified); Young et al. (2016, ds 2, ch. 27); Ristevski et al. (2018, ds 2, ch. 48); Smith et al. (in review, ds 1, ch. 49); Ősi et al. (in review, ds 1, ch. 59).*  *This character is a modification of the goniopholidid+tethysuchian enlarged foramina + associated fossae character, in which there are paired depressions on either maxilla, which are anteroposteriorly elongated, dorsoventrally high, complex and entirely supported by the maxilla.*  *State (1) occurs in Goniopholididae.*  *As noted for the maxilla/jugal presence of an enlarged foramina character, the anterior position of the ‘maxillary depressions’ in* Calsoyasuchus *are also not consistent with this character.*  0. absent, maxillary bony surface convex or flat  1. present |
| 65 | **Maxilla, morphology of anterior border of maxillary depressions:**  *Andrade et al. (2011, ch. 90); Ristevski et al. (2018, ds 2, ch. 49); Smith et al. (in review, ds 1, ch. 50); Ősi et al. (in review, ds 1, ch. 60).*  *State (1) is present within Goniopholididae (*Anteophthalmosuchus *and* Goniopholis*).*  0. shallow, anterior edge of depression usually poorly defined, or maxillary depression is absent  1. deep, anterior border always well-defined relative to dermal surface of maxilla |
| 66 | **Posterior maxilla, presence of a lateral fossa/fossae that crosses the maxillojugal suture:**  *Young et al. (2016, ds 2, ch. 28 modified); Ristevski et al. (2018, ds 2, ch. 50); Smith et al. (in review, ds 1, ch. 51); Ősi et al. (in review, ds 1, ch. 61).*  *This character is a modification of the goniopholidid + tethysuchian enlarged foramina + associated fossae character, in which there are paired depressions on either maxilla-jugal, which are anteroposteriorly elongated, dorsoventrally narrow, and contiguous on both the maxilla and jugal.*  *State (1) occurs in Pholidosauridae*  0. absent, maxillary bony surface convex or flat  1. present |
| 67 | **Maxilla, aligned set of large foramina extending posteroventrally from the antorbital/preorbital fossa:**  *Young et al. (2013a, ch. 15 modified); Young et al. (2012, ch. 19 modified); Young (2014, ch. 21); Young et al. (2016, ds 2, ch. 29); Ristevski et al. (2018, ds 2, ch. 51); Smith et al. (in review, ds 1, ch. 52); Ősi et al. (in review, ds 1, ch. 62).*  *State (1) is a putative apomorphy of Mr Leeds* Dakosaurus *+* Dakosaurus.  0. absent  1. present |
| 68 | **Maxilla-lachrymal, contact: (*)**  *Pol (1999, ch. 145); Young & Andrade (2009, ch. 141); Young et al. (2011, ch. 141); Young et al. (2013a, ch. 16); Young et al. (2012, ch. 20); Young (2014, ch. 22); Young et al. (2016, ds 2, ch. 30); Ristevski et al. (2018, ds 2, ch. 52); Smith et al. (in review, ds 1, ch. 53); Ősi et al. (in review, ds 1, ch. 63).*  *This character is not applicable for taxa that lack the antorbital/preorbital fossae.*  0. partially included in antorbital/preorbital fossa  1. completely included |
| 69 | **Lachrymal, contact with the nasal:**  *Young & Andrade (2009, ch. 97); Young et al. (2011, ch. 97); Young et al. (2013a, ch. 17); Young et al. (2012, ch. 21); Young (2014, ch. 23); Young et al. (2016, ds 2, ch. 31); Ristevski et al. (2018, ds 2, ch. 53); Smith et al. (in review, ds 1, ch. 54); Ősi et al. (in review, ds 1, ch. 64).*  0. nasal only contacts the dorsal margin of the lachrymal  1. nasal primarily contacts the anterior margin of the lachrymal  2. no contact between the nasals and lachrymals |
| 70 | **Nasal-lachrymal suture, length compared to nasal-prefrontal suture (in dorsal view): (*)**  *Young & Andrade (2009, ch. 136 modified); Young et al. (2011, ch. 136 modified); Young et al. (2013a, ch. 18 modified); Young et al. (2012, ch. 22 modified); Young (2014, ch. 24 modified); Young et al. (2016, ds 2, ch. 32 modified); Ristevski et al. (2018, ds 2, ch. 54); Smith et al. (in review, ds 1, ch. 55); Ősi et al. (in review, ds 1, ch. 65).*  *Ristevski et al. (2018) added a new character state.*  *This character is not applicable for taxa that lack the nasal-lachrymal contact.*  0. short – nasolachrymal suture is approximately 60% of the nasoprefrontal suture  1. the two sutures are sub-equal (± 25%)  2. long – nasolachrymal suture is approximately twice the length of the nasoprefrontal suture (i.e. elongation of the lachrymals) |
| 71 | **Lachrymal, dorsal exposure:**  *Young (2006, ch. 13); Wilkinson et al. (2008, ch. 33); Young & Andrade (2009, ch. 33); Young et al. (2011, ch. 33); Young et al. (2013a, ch. 19); Young et al. (2012, ch. 23); Young (2014, ch. 25); Young et al. (2016, ds 2, ch. 33); Ristevski et al. (2018, ds 2, ch. 55); Smith et al. (in review, ds 1, ch. 56); Ősi et al. (in review, ds 1, ch. 66).*  0. present, can be observed in both dorsal and lateral view  1. absent, only visible in lateral view (lachrymal vertically orientated) |
| 72 | **Lachrymal, dorsal surface lateral development:**  *Ristevski et al. (2018, ds 2, ch. 56); Smith et al. (in review, ds 1, ch. 57); Ősi et al. (in review, ds 1, ch. 67).*  *This character scores a slight lachrymal overhang of the orbits. These structures are the anterior palpebral sutural attachements, which are medially positioned.*  *State (1) occurs in* *goniopholidids + tethysuchians (except dyrosaurids,* Terminonaris *and* Oceanosuchus*)*  0. flush with the rim of the orbit  1. enlarged, extending laterally over the orbit |
| 73 | **Lachrymal, size:**  *Young (2006, ch. 14); Wilkinson et al. (2008, ch. 34); Young & Andrade (2009, ch. 34); Young et al. (2011, ch. 34); Young et al. (2013a, ch. 20); Young et al. (2012, ch. 24); Young (2014, ch. 26); Young et al. (2016, ds 2, ch. 34); Ristevski et al. (2018, ds 2, ch. 57); Smith et al. (in review, ds 1, ch. 58); Ősi et al. (in review, ds 1, ch. 68).*  0. large, in lateral view at least 45% of orbit height  1. small, less than 40% of orbit height |
| 74 | **Antorbital cavity, presence:**  *Clark (1994, ch. 67 modified);* *Young & Andrade (2009, ch. 88 part); Andrade et al. (2011, ch. 43 modified); Young et al. (2011, ch. 88 part); Young et al. (2013a, ch. 21 part); Young et al. (2012, ch. 23 part); Young (2014, ch. 27 part); Young et al. (2016, ds 2, ch. 35 part); Ristevski et al. (2018, ds 2, ch. 58 part); Smith et al. (in review, ds 1, ch. 59 part); Ősi et al. (in review, ds 1, ch. 69).*  *Antorbital cavity (CA), internal and external antorbital fenestra (FAO, FAOE, FAOI) as in Witmer (1997). The antorbital cavity or the FAOE must not be confounded with the shallow fossa located directly in front of the eyes (=prefrontal-lachymal fossa sensu Young & Andrade, 2009; =lachrymal fossa sensu Andrade et al., 2011)*.  *Note that here we have modified this character so that the presence of the antorbital cavity implies in the presence of a fenestra connecting the fossa with the internal antorbital sinuses (see Fernández & Herrera, 2009). We have not created a multi-state for this character with state (2) scoring for the preorbital condition, as it is unclear whether basal thalattosuchians had the antorbital fenestrae as openings for both the antorbital sinus and for the drainage duct of the hypertrophied nasal exocrine glands.*  0. absent (internalised, or the opening does not communicate with the antorbital sinus)  1. present (non-internalised, and the antorbital fenestra communicates with the antorbital sinus) |
| 75 | **Antorbital/preorbital cavity:**  *Young et al. (2013a, ch. 25 modified); Young et al. (2012, ch. 31 modified); Young (2014, ch. 33 modified); Young et al. (2016, ds 2, ch. 41 modified); Ristevski et al. (2018, ds 2, ch. 64 modified); Smith et al. (in review, ds 1, ch. 65 modified); Ősi et al. (in review, ds 1, ch. 70).*  *If hypothesis 2 of Fernández & Herrera (2009) is correct, and in metriorhynchids the antorbital cavity is internalised and the opening classically referred to as the “*antorbital fenestra*” are in fact neomorphic openings for the excretion of salt; then those taxa will score as (1) here, and (0) for the previous character on the presence/absence of the antorbital cavity.*  *However, should the preorbital and antorbital fenestrae be found to be homologous, the scoring distinction currently made would still be valid. Basal metriorhynchoids which have an intermediate condition, with the openings communicating with both the antorbital sinus and for the drainage duct of the hypertrophied nasal exocrine glands would score as (1) for both characters.*  *The current character construction thus does not favour one hypothesis over the other.*  *The preorbital fenestra itself is typically much smaller than realised, being a small sub-circular opening at the posterior-end of the deep fossa (where the lachrymals, nasals, jugals and maxillae converge). The deep concavity in this region can sometimes be filled with matrix, making the fenestra itself appear much larger than it really is.*  *Note that in Metriorhynchidae these fenestrae are set between the lachrymal, jugal and maxilla; typically, the jugal anterior ramus overlaps the maxilla externally, such that both bones contribute to the foraminal opening anteriorly. The inclusion of the nasal to the fenestra is unclear. It could be present in* Dakosaurus *and* Maledictosuchus*, but preservation in this region makes it hard to discern.*  0. absent (internalised, or the opening communicates solely with the antorbital sinus)  1. present (non-internalised, and the antorbital/preorbital fenestra communicates with the duct to the nasal exocrine gland) |
| 76 | **Antorbital cavity, relation between external and internal antorbital/preorbital fenestrae: (*)**  *Andrade et al. (2011, ch. 45 modified); Ristevski et al. (2018, ds 1, ch. 45 modified); Smith et al. (in review, ds 2, ch. 45 modified); Ősi et al. (in review, ds 1, ch. 71).*  *State (2) is putative apomorphy of* Eoneustes *+ Metriorhynchidae.*  *This character is not* *applicable for taxa lacking antorbital/preorbital fenestrae.*  0. external and internal fenestrae subequal or not distinguishable  1. external fenestra larger than internal fenestra, but no more than twice its area  2. external fenestra much larger than internal fenestra, or external fenestra present and internal fenestra closed |
| 77 | **Antorbital/preorbital cavity, shape: (*)**  *Young (2006, ch. 19 modified); Wilkinson et al. (2008, ch. 41); Young & Andrade (2009, ch. 41); Andrade et al. (2011, ch. 46 modified); Young et al. (2011, ch. 41); Young et al. (2013a, ch. 23); Young et al. (2012, ch. 28); Young et al. (2016, ch. 38); Ristevski et al. (2018, ch. 61); Smith et al. (in review, ch. 62); Ősi et al. (in review, ds 1, ch. 72).*  *Note that this version of the character does not score for the elongate antorbital/preorbital cavity of metriorhynchoids. That morphological complex is scored by another character, relating to the presence of a sulcus anterior to the cavity. This means however, that any metriorhynchoid in which the cavity itself is elongated (such as as in the teleosauroid* Steneosaurus gracilirostris*) can be scored as state (1) for this character as well as for the sulcus character.*  *This character is not applicable for taxa that lack antorbital fenestrae.*  0. subcircular, subtriangular or lozenge-shaped  1. anteroposteriorly elongated |
| 78 | **Antorbital/preorbital cavity, presence of a sulcus anterior to the cavity:**  Gasparini *et al. (2006, ch. 246 modified);* *Young (2006, ch. 19 modified part); Wilkinson et al. (2008, ch. 41 modified part); Young & Andrade (2009, ch. 41 modified part); Andrade et al. (2011, ch. 46 modified part); Young et al. (2011, ch. 41 modified part); Young et al. (2013a, ch. 23 modified part); Ősi et al. (in review, ds 1, ch. 73).*  *State (1) is putative apomorphy of Metriorhynchoidea.*  *In* Pelagosaurus typus *the sulcus is present (see Witmer, 1997), but it can be easily missed due to preservation as it is shallow when compared to the morphology seen in the clade* Eoneustes *_+_ Metriorhynchidae*.  *In well preserved specimens the distinction between the anterior sulcus and the external antorbital/preorbital fenestra is distinct (see* Dakosaurus andiniensis and Torvoneustes coryphaeus*).*  *The external antorbital/preorbital fenestrae in the clade* Eoneustes *_+_ Metriorhynchidae are bound by the jugal, lachrymal, nasal and maxilla. The anterior fossa continues anteriorly as a sulcus or fossa, but is largely present on the lateral surface of the maxilla.*  *This character is not* *applicable for taxa lacking external antorbital/preorbital fenestrae.*  0. absent  1. present |
| 79 | **Antorbital cavity, size (area) of external antorbital/preorbital fenestra, relative to the orbit:**  *Clark (1994, ch. 67 modified);* *Andrade et al. (2011, ch. 47 modified); Ősi et al. (in review, ds 1, ch. 74).*  *States (0-1) occur in Crocodyliformes.*  *State (2) occurs in non-crocodyliforms.*  0. small, being much smaller than the orbit area, or the antorbital cavity absent  1. moderately large, being at least half the diameter of the orbit  2. large, almost as large as the orbit |
| 80 | **Antorbital cavity, size (length) of internal antorbital/preorbital fenestra relative to the orbit:**  *Clark (1994, ch. 67 modified);* *Young & Andrade (2009, ch. 88 modified part); Andrade et al. (2011, ch. 48); Young et al. (2011, ch. 88 modified part); Young et al. (2013a, ch. 21 modified part); Young et al. (2012, ch. 23 modified part); Young (2014, ch. 27 modified part); Young et al. (2016, ds 2, ch. 35 modified part); Ristevski et al. (2018, ds 2, ch. 58 modified part); Smith et al. (in review, ds 1, ch. 59 modified part); Ősi et al. (in review, ds 1, ch. 75).*  *States (0-1) occur in Crocodyliformes.*  *State (2) occurs in* Junggarsuchus *(with reversals in some crocodyliforms, such as* Calsoyasuchus*).*  *State (3) occurs in non-crocodyliforms.*  0. small, internal fenestra is less than 25% of the length of the orbit, or internal fenestra is absent  1. medium, internal fenestra is approximately 25-50% of the length of the orbit  2. large, internal fenestra is more than 50% of the length of the orbit  3. very large, internal fenestra approximately the same size as the orbit |
| 81 | **Antorbital cavity, nasal participation in the internal antorbital/preorbital fenestra: (*)**  *Ortega et al. (2000, ch. 70 modified);**Wilkinson et al. (2008, ch. 40); Young & Andrade (2009, ch. 40); Andrade et al. (2011, ch. 49 modified); Young et al. (2011, ch. 40); Young et al. (2013a, ch. 22 modified); Young et al. (2012, ch. 29 modified); Young (2014, ch. 31 modified); Young et al. (2016, ds 2, ch. 39 modified); Ristevski et al. (2018, ds 2, ch. 62 modified); Smith et al. (in review, ds 1, ch. 63 modified); Ősi et al. (in review, ds 1, ch. 76).*  *State (1) is a putative apomorphy of Metriorhynchidae. It also occurs in* Calsoyasuchus *and* Gracilisuchus*.*  *This character is not* *applicable for taxa lacking antorbital/preorbital fenestrae.*  0. absent, nasals excluded from the internal fenestra by a maxillo-lachrymal contact  1. present, nasals broadly reach the internal fenestra (or reach deep into the fossa, if the internal fenestra is closed or preorbital) |
| 82 | **Antorbital cavity, jugal participation in the external antorbital/preorbital fenestra: (*)**  *Wu & Sues (1996, ch. 14 revised); Clark et al. (2000, ch. 4); Ortega et al. (2000, ch. 71 revised);**Clark & Sues (2002, ch. 4); Sues et al. (2003, ch. 4); Clark et al. (2004, ch. 4);Young (2006, ch. 17); Wilkinson et al. (2008, ch. 39); Young & Andrade (2009, ch. 39); Andrade et al. (2011, ch. 50); Young et al. (2013a ch. 24 part); Young et al. (2012, ch. 30); Pol et al. (2013, ch. 4); Young (2014, ch. 32); Young et al. (2016, ds 2, ch. 40); Leardi et al. (2017, ch. 4);Ristevski et al. (2018, ds 2, ch. 63); Smith et al. (in review, ds 1, ch. 64); Ősi et al. (in review, ds 1, ch. 77).*  *Should be scored alongside the characters regarding the antorbital fenestra, not jugal, to facilitate cross-checking of inapplicable states due to the absence of the antorbital fenestra.*  *This character is not* *applicable for taxa lacking external antorbital/preorbital fenestrae.*  0. absent, jugal excluded from the external fenestra by a maxillary-lachrymal contact  1. present, jugal takes part in the external fenestra (or reach deep into the fossa, if the internal fenestra is closed or preorbital) |
| 83 | **Antorbital cavity, position relative to the rostrum: (*)**  *Andrade et al. (2011, ch. 51 modified); Ristevski et al. (2018, ds 2, ch. 66); Smith et al. (in review, ds 1, ch. 67); Ősi et al. (in review, ds 1, ch. 78).*  *State (1) is putative apomorphy of Thalattosuchia.*  *Ristevski et al. (2018) modified state (1) to say ‘approximately equidistant…’, as in some teleosauroids (*e.g. Steneosaurus brevior, Platysuchus multiscrobiculatus*) the cavity is almost equidistant between the orbits and alveolar margin. But, these taxa still have the antorbital cavity being noticeably anterior to the orbits, as with other thalattosuchians that have not closed these cavities.*  *This character is not* *applicable for taxa lacking external antorbital/preorbital fenestrae.*  0. closer to the orbit than to the alveolar margin  1. closer to the alveolar margin than to the orbit, or approximately equidistant (but with the cavity still noticeably anterior to the orbit) |
| 84 | **Antorbital cavity, position relative to the orbit: (*)**  *Andrade et al. (2011, ch. 52); Ősi et al. (in review, ds 1, ch. 79).*  *This character is not* *applicable for taxa lacking antorbital/preorbital fenestrae.*  0. close to the orbit, with lachrymal narrow between orbit and antorbital cavity  1. distant to the orbit, with lachrymal wide between orbit and antorbital cavity |
| 85 | **Prefrontal-lachrymal fossae:**  *Young & Andrade (2009, ch. 150); Young et al. (2011, ch. 150); Young et al. (2013a, ch. 27); Young et al. (2012, ch. 33); Young (2014, ch. 35); Young et al. (2016, ds 2, ch. 43); Ristevski et al. (2018, ds 2, ch. 67); Smith et al. (in review, ds 1, ch. 68); Ősi et al. (in review, ds 1, ch. 80).*  *Andrade et al. (2011, ch. 30) scores for a similar character, namely the presence of a lachrymal crest anterior to the orbit.*  *The prefrontal-lachrymal fossa (sensu Young & Andrade, 2009) refers to a shallow depression immediately anterior to the orbit, present on both the prefrontal and lachyrmal. It is situated posterior to the preorbital fenestra, and never contacts the preorbital fossa. There is a crest within this fossa that is present along the prefrontal-lachrymal contact (scored for by Andrade et al. 2011, ch. 30).*  *State (1) is a putative apomorphy of Metriorhynchidae.*  0. absent  1. present, with ridge following the sutural contact between these elements |

**Skull roof** (Ch. 86 – 137; 11.454% of characters)

*[skull roof proportions and arrangement, supratemporal fenestrae, dermatocranial bones (= ossa præfrontalia, os frontale, ossa postorbitalia, ossa squamosal and os parietale)]*

| # | Description |
| --- | --- |
| 86 | **Supratemporal skull roof, dorsal surface:**  *Clark (1994, ch. 24);* *Young (2006, ch. 10 modified); Wilkinson et al. (2008, ch. 29); Young & Andrade (2009, ch. 29); Andrade et al. (2011, ch. 118); Young et al. (2011, ch. 29); Young et al. (2013a, ch. 28); Young et al. (2012, ch. 34); Young (2014, ch. 36); Young et al. (2016, ds 2, ch. 44); Ristevski et al. (2018, ds 2, ch. 68); Smith et al. (in review, ds 1, ch. 69); Ősi et al. (in review, ds 1, ch. 81).*  *State (1) is a putative apomorphy of Crocodyliformes (reversal in Thalattosuchia).*  0. surface complex  1. flat skull table present, formed by flattened and levelled surfaces of frontal, postorbital, squamosal and parietal |
| 87 | **Posterior skull table:**  *Young & Andrade (2009, ch. 99); Young et al. (2011, ch. 99); Young et al. (2013a, ch. 29); Young et al. (2012, ch. 35); Young (2014, ch. 37); Young et al. (2016, ds 2, ch. 45); Ristevski et al. (2018, ds 2, ch. 69); Smith et al. (in review, ds 1, ch. 70); Ősi et al. (in review, ds 1, ch. 82).*  *Note that* Sphagesaurus *scores differently in this character, and for the preceding character.*  0. non-planar (squamosal ventral to horizontal level of postorbital and parietal)  1. planar (postorbital, squamosal, and parietal on same horizontal plane) |
| 88 | **Cranial table width relative to ventral portion of skull:**  *Young & Andrade (2009, ch. 113); Young et al. (2011, ch. 113); Young et al. (2013a, ch. 30); Young et al. (2012, ch. 36); Young (2014, ch. 38); Young et al. (2016, ds 2, ch. 46); Ristevski et al. (2018, ds 2, ch. 70); Smith et al. (in review, ds 1, ch. 71); Ősi et al. (in review, ds 1, ch. 83).*  0. nearly as wide  1. narrower |
| 89 | **Supratemporal skull roof, dorsal curvature and elongation of squamosal prongs, at maturity:**  *Brochu (1999, ch. 140);* *Young & Andrade (2009, ch. 148); Andrade et al. (2011, ch. 119); Young et al. (2011, ch. 148); Young et al. (2013a, ch. 31); Young et al. (2012, ch. 37); Young (2014, ch. 39); Young et al. (2016, ds 2, ch. 47); Ristevski et al. (2018, ds 2, ch. 71); Smith et al. (in review, ds 1, ch. 72); Ősi et al. (in review, ds 1, ch. 84).*  0. short posterolateral process of the squamosal  1. mature skull table with nearly horizontal sides; significant posterolateral process of the squamosal |
| 90 | **Supratemporal fenestrae, presence:**  *Ősi et al. (in review, ds 1, ch. 85)*  *State (1) occurs in Gobiosuchidae.*  *State (2) is a putative autapomorphy of* Iharkutosuchus makadii*.*  0. present as an evident fenestra  1. presence variable during ontogeny, with the fenestrae possibly open during early ontogenetic stages (only closing later), or with there being a distinct ‘depression’ in the supratemporal region with the fenestrae themselves being reduced to a small foramen or completely closed  2. absent throughout ontogeny (i.e. supratemporal fenestrae are closed by the frontal and parietal suturing from an early ontogenetic state, with no ‘depression’ in the region) |
| 91 | **Supratemporal fossa, presence of “infratemporal flanges”: (*)**  *Young & Andrade (2009, ch. 142 modified); Nesbitt (2011, ch. 144 modified); Young et al. (2011, ch. 142); Young et al. (2013a, ch. 36); Young et al. (2012, ch. 44 modified); Young (2014, ch. 46 modified); Young et al. (2016, ds 2, ch. 48); Ristevski et al. (2018, ds 2, ch. 72); Smith et al. (in review, ds 1, ch. 73); Ősi et al. (in review, ds 1, ch. 86).*  *This character tests the homology of metriorhynchid "infratemporal flanges" and the teleosauroid anteromedial supratemporal fossae, with the anterior extension seen in basal crocodylomorphs.*  *State (0) is a putative apomorphy of Crocodyliformes (reversal in Thalattosuchia)*  *Note, this character scores for the ‘flat platform’ formed by the frontal, and not the concavity that can form in neosuchians.*  *This character is not* *applicable for taxa lacking supratemporal fenestrae.*  0. absent anterior to, and anteromedially to, the supratemporal fenestra  1. present anterior to, or anteromedially to, the supratemporal fenestra |
| 92 | **Supratemporal fossa, anterior margin in dorsal view: (*)**  *Young (2006, ch. 9 modified); Wilkinson et al. (2008, ch. 28); Young & Andrade (2009, ch. 28); Young et al. (2011, ch. 28); Young et al. (2013a, ch. 32); Young et al. (2012, ch. 38); Young (2014, ch. 40); Young et al. (2016, ds 2, ch. 49); Ristevski et al. (2018, ds 2, ch. 73); Smith et al. (in review, ds 1, ch. 74); Ősi et al. (in review, ds 1, ch. 87).*  *This character was designed to quantify the anterior extent of the supratemporal fossae. In Metriorhynchidae, the fossae begin to invade the dorsal surface of the orbital region. In* Dakosaurus*,* Purranisaurus potens, Cricosaurus saltillensis, *and* C. schroederi *the supratemporal fossae extend as far anteriorly as the minimum interorbital distance (state 3).*  *This character is not* *applicable for taxa lacking supratemporal fenestrae.*  0. anterior margin terminates posterior to the postorbital  1. anterior margin terminates between the anterior and posterior points of the frontal-postorbital suture  2. anterior margin terminates level to the postorbital anterior margin  3. anterior margin projects more anteriorly than the postorbital and reaches the interorbital minimum distance |
| 93 | **Supratemporal fossae, overall shape: (*)**  *Young & Andrade (2009, ch. 110 + 120 part modified); Andrade et al. (2011, ch. 111 modified); Young et al. (2011, ch. 110 + 120 part modified); Young et al. (2013a, ch. 33 part modified);*  *Young et al. (2012, ch. 39 + 40 + 41 modified); Young (2014, ch. 41 + 42 + 43); Young et al. (2016, ds 2, ch. 50 + 51 + 52 modified); Ristevski et al. (2018, ds 2, ch. 74); Smith et al. (in review, ds 1, ch. 75); Ősi et al. (in review, ds 1, ch. 88).*  *This character is an amalgam of character 111 from Andrade et al. (2011), and characters 50, 51 and 52 from Young et al. (2016, ds 2).*  *In Thalattosuchia, state (1) is a putative apomorphy for* Teleosaurus cadomensis *and* Maledictosuchus ricalensis.  *State (2) occurs in* Elosuchus *and* Vectisuchus*.*  *In Thalattosuchia, state (4) is a putative apomorphy of* Cricosaurus araucanensis *and* C. vignaudi.  *State (6) is a putative apomorphy of Machimosaurini*.  *This character is not* *applicable for taxa lacking supratemporal fenestrae.*  0. longitudinal ellipsoid/sub-rectangular (anteroposterior axis more than 10% longer than the lateromedial axis)  1. square-shaped to sub-rectangular (anteroposterior axis more than 10% longer than the lateromedial axis)  2. transverse triangle-shaped, with the axis converging medially (lateromedial axis more than 10% longer than the anteroposterior axis  3. circular to sub-circular  4. triangle-shaped, axis converging medially  5. parallelogram: lateral and medial margins, and anterior and posterior margins are sub-parallel |
| 94 | **Supratemporal fossa/fenestra, anterior margin shape, anterolateral expansion: (*)**  *Ristevski et al. (2018, ds 2, ch. 75); Smith et al. (in review, ds 1, ch. 76); Ősi et al. (in review, ds 1, ch. 89).*  *State (1) occurs in the teleosauroids* Mycterosuchus nasutus, Peipehsuchus teleorhinus*,* Platysuchus multiscrobiculatus*,* Teleosaurus cadomensis *and* Steneosaurus brevior.  *This character is not* *applicable for taxa lacking supratemporal fenestrae.*  0. no anterolateral expansion of the supratemporal fenestrae/fossae  1. anterior margin of the supratemporal fossae are noticeably inclined anterolaterally, such that the anterolateral corners of the supratemporal fossae are noticeably more anterior than the anteromedial corners of the supratemporal fossae |
| 95 | **Supratemporal fenestra, overall anteroposterior elongation: (*)**  *Ristevski et al. (2018, ds 2, ch. 76); Smith et al. (in review, ds 1, ch. 77); Ősi et al. (in review, ds 1, ch. 90).*  *State (1) occurs in derived teleosauroids.*  *This character is not homologous to the anteroposterior elongation of the supratemporal fenestrae in other clades, as it is caused by the extreme anteroposterior elongation of the proötics, laterosphenoids, postorbital posterior processes, parietal anterior process and frontal posterior process.*  *This character is not* *applicable for taxa lacking supratemporal fenestrae.*  0. length is either less than, or approximately sub-equal to the anterior width  1. length is twice as long as the anterior width, or more. In *Machimosaurus*, the width of the supratemporal fenestrae increases, however the extreme elongation of the bones is still present. |
| 96 | **Supratemporal fenestra, overall anteroposterior elongation: (*)**  *Ristevski et al. (2018, ds 2, ch. 77); Smith et al. (in review, ds 1, ch. 78); Ősi et al. (in review, ds 1, ch. 91).*  *State (1) occurs in dyrosaurids.*  *This character is not homologous to the anteroposterior elongation of the supratemporal fenestrae in teleosauroids, as it is caused by the anteroposterior elongation of the laterosphenoids, postorbital posterior processes, squamosal anterior processes and parietal anterior process.*  *This character is not* *applicable for taxa lacking supratemporal fenestrae.*  0. length is either less than, or approximately sub-equal to the width at the middle of the fenestra (± 25%)  1. length is greater than the width of the fenestra (greater than 125%) |
| 97 | **Supratemporal fenestra, in dorsal view, size relative to orbits: (*)**  *Young (2006, ch. 11); Wilkinson et al. (2008, ch. 30); Young & Andrade (2009, ch. 30); Young et al. (2011, ch. 30); Young et al. (2013a, ch. 34); Young et al. (2012, ch. 42); Young (2014, ch. 44); Young et al. (2016, ds 2, ch. 53); Ristevski et al. (2018, ds 2, ch. 78); Smith et al. (in review, ds 1, ch. 79); Ősi et al. (in review, ds 1, ch. 92).*  *This character is not* *applicable for taxa lacking supratemporal fenestrae.*  0. longer in length than the orbit (supratemporal length 110% or more of orbit length)  1. subequal in length as the orbit (± 5%)  2. smaller than the orbits (supratemporal length less than 90% of orbit length) |
| 98 | **Supratemporal fenestra, in dorsal view, posterior limit: (*)**  *Wilkinson et al. (2008, ch. 31 modified); Young & Andrade (2009, ch. 31 modified); Young et al. (2011, ch. 31 modified); Young et al. (2013a, ch. 35 modified); Young et al. (2012, ch. 43); Young (2014, ch. 45); Young et al. (2016, ds 2, ch. 54); Ristevski et al. (2018, ds 2, ch. 79); Smith et al. (in review, ds 1, ch. 80); Ősi et al. (in review, ds 1, ch. 93).*  *State (2) is a putative apomorphy of the* Dakosaurus+Plesiosuchus *sub-clade.*  *Note, scoring of this character should be done carefully, it may not be possible to score for skulls that have suffered taphonomic dorsoventral compression/shearing.*  *This character is not* *applicable for taxa lacking supratemporal fenestrae.*  0. terminates well before the posterior-most point of the parietal  1. either terminates near the posterior-most point of the parietal or exceeds it, but never reaches the supraoccipital  2. more posterior than intertemporal bar |
| 99 | **Supratemporal fenestra/fossae, posterior margin in dorsal view: (*)**  *Jouve et al. (2005b, ch. 10 modified), Jouve (2005, ch. 6 modified), Jouve et al. (2008, ch. 10 modified), Hastings et al. (2010, ch. 10 modified); Ristevski et al. (2018, ds 2, ch. 80); Smith et al. (in review, ds 1, ch. 81); Ősi et al. (in review, ds 1, ch. 94).*  *State (1) occurs in derived dyrosaurids.*  *This character is not applicable for taxa that lack the ‘skull table’ temporal morphotype, or taxa that lack supratemporal fenestrae.*  0. supratemporal fenestral posterior wall largely vertical and barely visible in dorsal view  1. supratemporal fenestral posterior wall posterodorsally inclined, creating a posterior fossa that is visible in dorsal view |
| 100 | **Supratemporal arch, medial margin in dorsal view: (*)**  *Young & Andrade (2009, ch. 91); Young et al. (2011, ch. 91); Young et al. (2013a, ch. 37); Young et al. (2012, ch. 45); Young (2014, ch. 47); Young et al. (2016, ds 2, ch. 55); Ristevski et al. (2018, ds 2, ch. 81); Smith et al. (in review, ds 1, ch. 82); Ősi et al. (in review, ds 1, ch. 95).*  *State (1) is a putative apomorphy of* ‘Dakosaurus’ lissocephalus + Cricosaurus*.*  *This character is not* *applicable for taxa lacking supratemporal fenestrae.*  0. not convex  1. convex |
| 101 | **Supratemporal arch, dorsal margin in lateral view: (*)**  *Young & Andrade (2009, ch. 98); Young et al. (2011, ch. 98); Young et al. (2013a, ch. 38); Young et al. (2012, ch. 46); Young (2014, ch. 48); Young et al. (2016, ds2, ch. 56); Ristevski et al. (2018, ds 2, ch. 82); Smith et al. (in review, ds 1, ch. 83); Ősi et al. (in review, ds 1, ch. 96).*  *This character is not* *applicable for taxa lacking supratemporal fenestrae.*  0. concave  1. straight  2. convex |
| 102 | **Supratemporal arch, width in dorsal view: (*)**  *Jouve et al. (2005b, ch. 16 modified), Jouve et al. (2008, ch. 16 modified), Hastings et al. (2010, ch. 11 modified); Ristevski et al. (2018, ds 2, ch. 83); Smith et al. (in review, ds 1, ch. 84); Ősi et al. (in review, ds 1, ch. 97).*  *State (1) scores the thin supratemporal arches of Dyrosauridae (with some reversals).*  *This character is not applicable for taxa that lack the ‘skull table’ temporal morphotype, or taxa that lack supratemporal fenestrae.*  0. thick  1. thin |
| 103 | **Prefrontal, dorsal surface lateral development: (ORDERED)**  *Gasparini et al. (2006, ch. 247 modified);* *Young (2006, ch. 2 modified); Wilkinson et al. (2008, ch. 12); Jouve (2009, ch. 255 modified); Young & Andrade (2009, ch. 12); Andrade et al. (2011, ch. 125 modified); Young et al. (2011, ch. 12); Young et al. (2013a, ch. 39); Young et al. (2012, ch. 47); Young (2014, ch. 49); Young et al. (2016, ds 2, ch. 57); Ristevski et al. (2018, ds 2, ch. 84); Smith et al. (in review, ds 1, ch. 85); Ősi et al. (in review, ds 1, ch. 98).*  *The transverse development of the prefrontal is a classic characteristic of Metriorhynchidae.*  *State (1) is a putative apomorphy of* Eoneustes*, however it could be more widespread among basal metriorhynchoids.*  *State (2) is a putative apomorphy of Metriorhynchidae.*  0. reduced, flush with the rim of the orbit  1. incipient enlargement (extending laterally over the orbit by approximately 5% of its width)  2. enlarged (extending laterally over the orbit by more than 15% of its width) |
| 104 | **Prefrontal, lateral development relative to the posterolateral corner of the supratemporal fossa in dorsal view: (*)**  *Wilkinson et al. (2008, ch. 13 modified); Young & Andrade (2009, ch. 13 modified); Young et al. (2011, ch. 13 modified); Young et al. (2013a, ch. 40); Young et al. (2012, ch. 48); Young (2014, ch. 50); Young et al. (2016, ds 2, ch. 58); Ristevski et al. (2018, ds 2, ch. 85); Smith et al. (in review, ds 1, ch. 86); Ősi et al. (in review, ds 1, ch. 99).*  *This character is not* *applicable for taxa lacking supratemporal fenestrae.*  0. prefrontal does not expand laterally so that it is in the same plane as the posterolateral corner of the supratemporal fossa  1. prefrontal expands further laterally than the posterolateral corner of the supratemporal fossa |
| 105 | **Prefrontal, shape in dorsal view:**  *Wilkinson et al. (2008, ch. 14 modified part); Young & Andrade (2009, ch. 14 modified part); Young et al. (2011, ch. 14 modified part); Young et al. (2013a, ch. 41 modified part); Young et al. (2012, ch. 49); Young (2014, ch. 51); Young et al. (2016, ds 2, ch. 59); Ristevski et al. (2018, ds 2, ch. 86); Smith et al. (in review, ds 1, ch. 87); Ősi et al. (in review, ds 1, ch. 100).*  *State (1) is a putative apomorphy of Metriorhynchidae.*  0. quadrilateral with irregular outline  1. teardrop-shaped |
| 106 | **Prefrontal, morphology of the lateral border in dorsal view: (*)**  *Wilkinson et al. (2008, ch. 14 modified part); Young & Andrade (2009, ch. 14 modified part); Young et al. (2011, ch. 14 modified part); Young et al. (2013a, ch. 41 modified part); Young et al. (2012, ch. 50); Young (2014, ch. 52); Young et al. (2016, ds 2, ch. 60); Ristevski et al. (2018, ds 2, ch. 87); Smith et al. (in review, ds 1, ch. 88); Ősi et al. (in review, ds 1, ch. 101).*  *This character describes the shape of the prefrontal in Metriorhynchidae, and thus is not applicable for taxa that do not have the lateral expansion of the prefrontal.*  Eoneustes*, metriorhynchines and basal geosaurines score as state (0).*  *State (1) is a putative apomorphy of Geosaurini.*  *State (2) is a putative apomorphy of* Dakosaurus *(a modification of the Geosaurini condition).*  0. continuous convex curve, inflexion point approximately 80–90 degree angle from the anteroposterior axis of the skull  1. continuous convex curve, inflexion point approximately 60–70 degree angle from the anteroposterior axis of the skull  2. continuous convex curve, inflexion point approximately 50 degree angle from the anteroposterior axis of the skull |
| 107 | **Prefrontal, dimensions in dorsal view:**  *Wilkinson et al. (2008, ch. 15); Young & Andrade (2009, ch. 15); Young et al. (2011, ch. 15); Young et al. (2013a, ch. 42); Young et al. (2012, ch. 51); Young (2014, ch. 53); Young et al. (2016, ds2, ch. 61); Ristevski et al. (2018, ds 2, ch. 88); Smith et al. (in review, ds 1, ch. 89); Ősi et al. (in review, ds 1, ch. 102).*  0. longer than wide  1. length/width is subequal (± 5%) |
| 108 | **Prefrontal, anterior to the orbits:**  *Wilkinson et al. (2008, ch. 16); Young & Andrade (2009, ch. 16); Young et al. (2011, ch. 16); Young et al. (2013a, ch. 43); Young et al. (2012, ch. 52); Young (2014, ch. 54); Young et al. (2016, ds 2, ch. 62); Ristevski et al. (2018, ds 2, ch. 89); Smith et al. (in review, ds 1, ch. 90); Ősi et al. (in review, ds 1, ch. 103).*  0. elongate, oriented parallel to antero-posterior axis of the skull  1. short and broad |
| 109 | **Prefrontal, nasal-prefrontal suture has a pronounced, rectangular ‘concavity’ (directed posteriorly):**  *Young & Andrade (2009, ch. 93); Young et al. (2011, ch. 93); Young et al. (2013a, ch. 44); Young et al. (2012, ch. 53); Young (2014, ch. 55); Young et al. (2016, ds 2, ch. 63); Ristevski et al. (2018, ds 2, ch. 90); Smith et al. (in review, ds 1, ch. 91); Ősi et al. (in review, ds 1, ch. 104).*  *State (1) is a putative apomorphy of* Eoneustes*.*  0. absent  1. present |
| 110 | **Prefrontal, nasal-prefrontal suture has a posteriorly directed ‘V’-shape:**  *Young & Andrade (2009, ch. 140); Young et al. (2011, ch. 140); Young et al. (2013a, ch. 45); Young et al. (2012, ch. 54); Young (2014, ch. 56); Young et al. (2016, ds 2, ch. 64); Ristevski et al. (2018, ds 2, ch. 91); Smith et al. (in review, ds 1, ch. 92); Ősi et al. (in review, ds 1, ch. 105).*  *State (1) is a putative autapomorphy of* Cricosaurus macrospondylus*.*  0. absent  1. present |
| 111 | **Frontal, dorsal surface along the midline:**  *Nesbitt (2011, ch. 42 modified); Young et al. (2016, ds 2, ch. 66); Ristevski et al. (2018, ds 2, ch. 92); Smith et al. (in review, ds 1, ch. 93); Ősi et al. (in review, ds 1, ch. 106).*  *State (0) is a putative apomorphy of Crocodyliformes (although there is a reversal in numerous neosuchian clades)*  0. flat  1. an incomplete longitudinal ridge along the midline  2. a longitudinal ridge that proceeds along the entire length of the midline |
| 112 | **Frontal, dorsal surface:**  *Young et al. (2016, ds 2, ch. 67); Ristevski et al. (2018, ds 2, ch. 93); Smith et al. (in review, ds 1, ch. 94); Ősi et al. (in review, ds 1, ch. 107).*  *State (1) occurs in* Hesperosuchus *cf.* agilis, Dromicosuchus grallator, *and among many tethysuchians (except derived dyrosaurids)*  0. slightly convex or flat  1. concave, with the medial borders of the orbit upturned |
| 113 | **Frontal, anteromedial process length: (*)**  *Jouve et al. (2008, ch. 31 modified), Hastings et al. (2010, ch. 38 modified); Ristevski et al. (2018, ds 2, ch. 94); Smith et al. (in review, ds 1, ch. 95); Ősi et al. (in review, ds 1, ch. 108).*  *This character is not applicable for* Anthracosuchus *and* Cerrejonisuchus *as the anterior region of the frontal is elongated and the prefrontals are reduced (i.e. there is no elongation of the anteromedial process).*  0. the anteromedial process is approximately level to, or slightly posterior to, the prefrontals  1. the anteromedial process is noticeably posterior to the prefrontals |
| 114 | **Frontal, anteromedial process:**  *Young et al. (2016, ds 2, ch. 68); Ristevski et al. (2018, ds 2, ch. 95); Smith et al. (in review, ds 1, ch. 96); Ősi et al. (in review, ds 1, ch. 109).*  *State (1) is a putative apomorphy of Sebecia, also occurs in some basal dyrosaurids, bernissartiids and hylaeochampsids.*  0.frontal anteromedial process has an acute anterior margin, which separates the left and right nasals along their posterior margin  1. frontal anteromedial process lacks an acute anterior margin, with the nasal posterior margin with the frontal being either transversely straight, or is slightly convex or concave (in taxa where the prefrontals expand anterolaterally, there can sometimes be posteromedial processes of the nasals) |
| 115 | **Frontal, contribution to the intertemporal bar: (*)**  *Smith et al. (in review, ds 1, ch. 97); Ősi et al. (in review, ds 1, ch. 110).*  *This character is not applicable for taxa that lack supratemporal fenestrae.*  *Note that in many crocodyliforms the frontal only forms the very anterior region of the intersupratemporal fenestral area. We only score taxa as state (1) if the frontal is clearly anterior to the bar.*  *State (1) occurs in* Protosuchus*,* Mahajangasuchus*,* Elosuchus*,* Vectisuchus*,* Chalawan*,* Sarcosuchus*, and Crocodylia.*  0. frontal contributes to the anterior part of the intertemporal bar  1. frontal is excluded from the intertemporal bar, with the bar being solely composed by the parietal |
| 116 | **Frontal, angle between posteromedial and posterolateral processes: (*)**  *Wilkinson et al. (2008, ch. 26 modified); Young & Andrade (2009, ch. 26 modified); Andrade et al. (2011, ch. 98 modified); Young et al. (2011, ch. 26); Young et al. (2013a, ch. 47); Young et al. (2012, ch. 56); Young (2014, ch. 58); Young et al. (2016, ds 2, ch. 69); Ristevski et al. (2018, ds 2, ch. 96); Smith et al. (in review, ds 1, ch. 98); Ősi et al. (in review, ds 1, ch. 111).*  *See diagrammatic explanation for this character in Wilkinson et al. (2008: p.1311, Fig. 4).*  *This character is not applicable for taxa that lack supratemporal fenestrae (which help form the distinct posterior processes of the frontal).*  0. approximately 90 degree angle, or obtuse  1. approximately 70–60 degree angle  2. approximately 45 degree angle, or more acute |
| 117 | **Frontal, minimum width between orbits in dorsal view compared to the supratemporal fossa: (*)**  *Young & Andrade (2009, ch. 121); Young et al. (2011, ch. 121); Young et al. (2013a, ch. 48); Young et al. (2012, ch. 57); Young (2014, ch. 59); Young et al. (2016, ds 2, ch. 70); Ristevski et al. (2018, ds 2, ch. 97); Smith et al. (in review, ds 1, ch. 99); Ősi et al. (in review, ds 1, ch. 112).*  *This character is not applicable for taxa that lack supratemporal fenestrae.*  0. greater than, or equal to, the width of one supratemporal fossa and the intertemporal bar  1. subequal to width of one supratemporal fossa |
| 118 | **Frontal, minimum width between orbits in dorsal view compared to the orbits:**  *Young & Andrade (2009, ch. 137); Young et al. (2011, ch. 137); Young et al. (2013a, ch. 49); Young et al. (2012, ch. 58); Young (2014, ch. 60); Young et al. (2016, ds 2, ch. 71); Ristevski et al. (2018, ds 2, ch. 98); Smith et al. (in review, ds 1, ch. 100); Ősi et al. (in review, ds 1, ch. 113).*  0. broader than orbital width  1. subequal with orbital width  2. narrower than orbital width |
| 119 | **Frontal-parietal, between supratemporal fossa in dorsal view (intertemporal bar): (*)**  *Wilkinson et al. (2008, ch. 2); Young & Andrade (2009, ch. 2); Young et al. (2011, ch. 2); Young et al. (2013a, ch. 50); Young et al. (2012, ch. 59); Young (2014, ch. 61); Young et al. (2016, ds 2, ch. 72); Ristevski et al. (2018, ds 2, ch. 99); Smith et al. (in review, ds 1, ch. 101); Ősi et al. (in review, ds 1, ch. 114).*  *This character is not applicable for taxa that lack supratemporal fenestrae (as there is no intertemporal bar).*  0. frontal and parietal subequal in width (± 5%)  1. frontal width is wider than the parietal. Can be extreme (greater than 75%) |
| 120 | **Frontal-postorbital suture: (*)**  *Wilkinson et al. (2008, ch. 27 modified); Young & Andrade (2009, ch. 27 modified); Young et al. (2011, ch. 27); Young et al. (2013a, ch. 51); Young et al. (2012, ch. 60); Young (2014, ch. 62); Young et al. (2016, ds 2, ch. 73); Ristevski et al. (2018, ds 2, ch. 100); Smith et al. (in review, ds 1, ch. 102); Ősi et al. (in review, ds 1, ch. 115).*  *This character is not applicable for taxa that lack supratemporal fenestrae (as there is no intertemporal bar).*  0. level with the intertemporal bar  1. lower than the intertemporal bar |
| 121 | **Frontal-postorbital suture, in dorsal view:**  *Wilkinson et al. (2008, ch. 3 modified); Young & Andrade (2009, ch. 3 modified); Hastings et al. (2010, ch. 40 modified); Young et al. (2011, ch. 3 modified); Young et al. (2013a, ch. 52 modified); Young et al. (2012, ch. 61 modified); Young (2014, ch. 63 modified); Young et al. (2016, ds 2, ch. 74 modified); Ristevski et al. (2018, ds 2, ch. 101); Smith et al. (in review, ds 1, ch. 103); Ősi et al. (in review, ds 1, ch. 116).*  *This character is an amalgam of the Hastings et al. (2010, ch. 40) and Young et al. (2016, ds 2, ch. 74) characters.*  *State (1) is a putative apomorphy of Metriorhynchidae.*  *State (2) scores the dyrosaurid morphotype.*  0. irregular and straight or gently curved  1. frontal overlaps the postorbital, creating a ‘V’-shape directed posteriorly.  2. strongly interdigitating in dorsal view (largely in one plane) |
| 122 | **Postorbital, shape in dorsal view:**  *Young & Andrade (2009, ch. 118); Young et al. (2011, ch. 118); Young et al. (2013a, ch. 53); Young et al. (2012, ch. 62); Young (2014, ch. 64); Young et al. (2016, ds 2, ch. 75); Ristevski et al. (2018, ds 2, ch. 102); Smith et al. (in review, ds 1, ch. 104); Ősi et al. (in review, ds 1, ch. 117).*  0. the outer margin is convex where the postorbital curves posteriorly forming the supratemporal arch  1. forms a 90 degree angle  2. anterior extension from the corner |
| 123 | **Postorbital, anterolateral extension:**  *Young & Andrade (2009, ch. 138); Young et al. (2011, ch. 138); Young et al. (2013a, ch. 54); Young et al. (2012, ch. 63); Young (2014, ch. 65); Young et al. (2016, ds 2, ch. 76); Ristevski et al. (2018, ds 2, ch. 103); Smith et al. (in review, ds 1, ch. 105); Ősi et al. (in review, ds 1, ch. 118).*  *State (1) of this character, and state (2) of the character “anterior extension from the postorbital corner” do not necessarily occur in the same taxon (e.g.* Oceanosuchus*).*  0. small or absent  1. very large, appearing in lateral view to contact the dorsal surface of the jugal |
| 124 | **Postorbital and squamosal, relative lengths in dorsal view:**  *Young (2006, ch. 15); Wilkinson et al. (2008, ch. 37); Young & Andrade (2009, ch. 37); Young et al. (2011, ch. 37); Young et al. (2013a, ch. 55); Young et al. (2012, ch. 64); Young (2014, ch. 66); Young et al. (2016, ds 2, ch. 77); Ristevski et al. (2018, ds 2, ch. 104); Smith et al. (in review, ds 1, ch. 106); Ősi et al. (in review, ds 1, ch. 119).*  *State (1) is a putative apomorphy of Thalattosuchia.*  0. squamosal is longer  1. postorbital is longer |
| 125 | **Supratemporal arch (= upper temporal bar), relative participation of the postorbital:**  *Ortega et al. (2000, ch. 33 modified);* *Andrade et al. (2011, ch. 151); Ristevski et al. (2018, ds 2, ch. 105); Smith et al. (in review, ds 1, ch. 107); Ősi et al. (in review, ds 1, ch. 120).*  *Young & Andrade (2009, ch. 127); Young et al. (2011, ch. 127); Young et al. (2013a, ch. 57); Young et al. (2012, ch. 66); Young (2014, ch. 68) and Young et al. (2016, ch. 79) score for the same morphology, however they used the squamosal contribution to the supratemporal arch.*  *State (1) is putative apomorphy of Thalattosuchia.*  *Note that a similar morphology also evolves in some derived dyrosaurids (elongatation of the postorbital posterior processes). In these taxa however, the character relating to the relative participation of the postorbital is not affected (i.e. the squamosal in dorsal view is still longer anteroposteriorly than the postorbital). The postorbital being longer overall, and makes a greater proportional contribution to the supratemporal arch than the squamosal, only co-occurs in Thalattosuchia.*  0. small, postorbital represents approximately 30% of the bar  1. extensive, postorbital represents approximately 50% (or more) of the bar |
| 126 | **Posterior margin of the squamosal lateral to post-temporal fenestrae:**  *Jouve et al. (2005b, ch. 29), Jouve et al. (2008, ch. 29), Hastings et al. (2010, ch. 48); Ristevski et al. (2018, ds 2, ch. 106); Smith et al. (in review, ds 1, ch. 108); Ősi et al. (in review, ds 1, ch. 121).*  *State (1) occurs in derived dyrosaurids.*  0. straight  1. anteriorly concave |
| 127 | **Squamosal, projects further posteriorly than the occipital condyle:**  *Young & Andrade (2009, ch. 125); Young et al. (2011, ch. 125); Young et al. (2013a, ch. 56); Young et al. (2012, ch. 65); Young (2014, ch. 67); Young et al. (2016, ds 2, ch. 78); Ristevski et al. (2018, ds 2, ch. 107); Smith et al. (in review, ds 1, ch. 109); Ősi et al. (in review, ds 1, ch. 122).*  0. no  1. yes |
| 128 | **Squamosal dorsolateral edge, longitudinal groove:**  *Young & Andrade (2009, ch. 112 part); Nesbitt (2011, ch. 53); Young et al. (2011, ch. 112 part); Young et al. (2013a, ch. 58 part); Young et al. (2012, ch. 67 part); Young (2014, ch. 69 part); Young et al. (2016, ds 2, ch. 80); Ristevski et al. (2018, ds 2, ch. 108); Smith et al. (in review, ds 1, ch. 110); Ősi et al. (in review, ds 1, ch. 123).*  *State (1) is a putative apomorphy of Crocodyliformes (reversals in Thalattosuchia and* Iharkutosuchus makadii*), but also occurs in some ‘sphenosuchians’.*  0. absent  1. present |
| 129 | **Squamosal dorsolateral edge, longitudinal groove margins: (*)**  *Young & Andrade (2009, ch. 112 part); Young et al. (2011, ch. 112 part); Young et al. (2013a, ch. 58 part); Young et al. (2012, ch. 67 part); Young (2014, ch. 69 part); Young et al. (2016, ds 2, ch. 81); Ristevski et al. (2018, ds 2, ch. 109); Smith et al. (in review, ds 1, ch. 111); Ősi et al. (in review, ds 1, ch. 124).*  *This character is not applicable for taxa that lack the squamosal longitudinal groove.*  0. ventral margin of the groove projects more laterally than the dorsal margin  1. ventral margin is directly underneath the dorsal margin |
| 130 | **Parietals, in presumed adults:**  *Nesbitt (2011, ch. 58); Young et al. (2016, ds 2, ch. 82); Ristevski et al. (2018, ds 2, ch. 110); Smith et al. (in review, ds 1, ch. 111); Ősi et al. (in review, ds 1, ch. 125).*  0. separate  1. interparietal suture partially or completely absent (i.e. surface fusion) |
| 131 | **Parietals, supratemporal (= dorsotemporal) fenestrae separated by: (*)**  *Nesbitt (2011, ch. 59 modified); Young et al. (2016, ds 2, ch. 83 modified); Ristevski et al. (2018, ds 2, ch. 111); Smith et al. (in review, ds 1, ch. 113); Ősi et al. (in review, ds 1, ch. 126).*  *State (3) added here.*  *State (3) occurs in* Dromicosuchus *and* Hesperosuchus *cf*. agilis*.*  *This character is not applicable for taxa that lack the supratemporal fenestrae.*  0. broad, flat area  1. supratemporal fossa separated by a mediolaterally thin strip of flat bone  2. supratemporal fossa separated by a ‘‘sagittal crest’’ (which may be divided by the interparietal suture)  3. supratemporal fossa separated by a median longitudinal groove between paired parietal crests |
| 132 | **Intertemporal bar (=frontoparietal), modification of the “sagittal crest”: (*)**  *Ristevski et al. (2018, ds 2, ch. 112); Smith et al. (in review, ds 1, ch. 114); Ősi et al. (in review, ds 1, ch. 127).*  *Character following Jouve et al. (2005a: figure 8), Hastings et al. (2010, ch. 9).*  *Note this character scores the distinct thin intertemporal bar of derived dyrosaurids. In Thalattosuchia the bar is not consistently thin along its entire length (being noticeably broad anteriorly).*  *This character is not applicable for taxa that lack the supratemporal fenestrae.*  0. either not a “sagittal crest”, or does not have the derived dyrosaurid morphotype  1. has the derived dyrosaurid morphotype: the intertemporal bar is composed of the frontal posterior process anteriorly and the parietal anterior process in the middle-and-posterior region, with a consistently thin bar along its entire length, and lateral margins deeply excavated creating a broad lateral supratemporal fossa |
| 133 | **Parietal, bifurcation of the parietal in dorsal view, immediately posterior to the intertemporal bar:**  *Young et al. (2016, ds 2, ch. 84); Ristevski et al. (2018, ds 2, ch. 113); Smith et al. (in review, ds 1, ch. 115); Ősi et al. (in review, ds 1, ch. 128).*  *State (1) is found in* ‘Dakosaurus’ lissocephalus, Cricosaurus araucanensis*,* C. elegans, C. lithographicus, C. schroederi *and* C. vignaudi.  *This character replaces the character that described the posterior margin of the parietal-squamosal in dorsal view – Wilkinson et al. (2008, ch. 42); Young & Andrade (2009, ch. 42); Young et al. (2011, ch. 42); Young et al. (2013a, ch. 59); Young et al. (2012, ch. 68); Young (2014, ch. 70).*  0. absent  1. present |
| 134 | **Parietals, posterodorsal margin:**  *Jouve (2005, ch. 7 modified), Jouve et al. (2005b, ch. 11 modified), Jouve et al. (2008, ch. 11 modified), Hastings et al. (2010, ch. 42 modified); Ristevski et al. (2018, ds 2, ch. 114); Smith et al. (in review, ds 1, ch. 116); Ősi et al. (in review, ds 1, ch. 129).*  *State (1) occurs in derived dyrosaurids.*  0. transversely oriented  1. indented anteriorly |
| 135 | **Parietals, posteroventral edge:**  *Nesbitt (2011, ch. 60); Young et al. (2016, ds 2, ch. 85); Ristevski et al. (2018, ds 2, ch. 115); Smith et al. (in review, ds 1, ch. 117); Ősi et al. (in review, ds 1, ch. 130).*  *State (1) is a putative apomorphy of Crocodyliformes.*  0. extending more than half the width of the occiput  1. extending less than half the width of the occiput |
| 136 | **Post-temporal fenestrae obscured in dorsal view by an overhanging posterior extension of the parietal:**  *Jouve et al. (2008, ch. 34 modified); Hastings et al. (2010, ch. 46 modified); Ristevski et al. (2018, ds 2, ch. 116); Smith et al. (in review, ds 1, ch. 118); Ősi et al. (in review, ds 1, ch. 131).*  *State (1) occurs in derived dyrosaurids.*  0. absent  1. present |
| 137 | **Parietal in occipital view:**  *Jouve et al. (2008, ch. 32 modified); Hastings et al. (2010, ch. 44 modified); Ristevski et al. (2018, ds 2, ch. 117); Smith et al. (in review, ds 1, ch. 119); Ősi et al. (in review, ds 1, ch. 132).*  0. ‘W-shaped’  1. concave  2. flat or convex |

**Orbit and temporal region** (Ch. 138 – 166; 6.388% of characters)

*[orbit, circumorbital contributions, ossa palpebralia, ossa scleroticalia, dermatocranial bones (= ossa jugalia, ossa postfrontalia, postorbital bars and ossa quadratojugalia), infratemporal fenestrae]*

| # | Description |
| --- | --- |
| 138 | **Orbit, position:**  *Young (2006, ch. 3 modified); Wilkinson et al. (2008, ch. 18 modified); Young & Andrade (2009, ch. 18 modified); Andrade et al. (2011, ch. 157 modified); Young et al. (2011, ch. 18); Young et al. (2013a, ch. 60); Young et al. (2012, ch. 69); Young (2014, ch. 71); Young et al. (2016, ds 2, ch. 86); Ristevski et al. (2018, ds 2, ch. 118); Smith et al. (in review, ds 1, ch. 120); Ősi et al. (in review, ds 1, ch. 133).*  *Note, when scoring the orientation of the orbits, the palpebrals must not be considered.*  0. fully dorsal  1. mainly dorsal, but with slight inclination  2. lateral, but slightly inclined dorsally, usually visible in dorsal view  3. fully lateral with orbit shape only clear in lateral view |
| 139 | **Orbit, shape:**  *Young & Andrade (2009, ch. 96); Young et al. (2011, ch. 96); Young et al. (2013a, ch. 61); Young et al. (2012, ch. 70); Young (2014, ch. 72); Young et al. (2016, ds 2, ch. 87); Ristevski et al. (2018, ds 2, ch. 119); Smith et al. (in review, ds 1, ch. 121); Ősi et al. (in review, ds 1, ch. 134).*  0. circular, anteroposterior and dorsoventral axes subequal (± 5%)  1. longitudinal ellipsoid, anteroposterior axis more than 10% longer than mediolateral axis  2. transverse ellipsoid, mediolateral axis more than 10% longer than anteroposterior axis |
| 140 | **Circumorbital dorsal margin, shape:**  *Brochu (1999, ch. 103 modified); Salas-Gismondi et al. (2016, ch. 137 modified); Smith et al. (in review, ds 1, ch. 122); Ősi et al. (in review, ds 1, ch. 135).*  *See explanation for this character see Figure 7 in Salas-Gismondi et al. (2016).*  *State (1) occurs in the French* Pholidosaurus*,* Elosuchus*,* Teleosaurus*,* Mycterosuchus*.*  *State (2) occurs in* Vectisuchus*,* Sarcosuchus*,* Gavialis*.*  Chalawan *has evidence of the dorsal medial margin being upturned, but the posterior margins of the orbits are not preserved (Martin et al., 2014)*.  *Note this character is not equivalent to having a concave frontal, as here it is the upturning of the orbital margins that are being scored. Among many taxa with ‘telescoped’ orbits the frontal is also concave, but not all tethysuchians with concave frontals have the ‘telescoped’ orbit condition.*  *This character helps to quantify the ‘telescoped’ orbit morphology.*  0. dorsal margins of orbits are flush with the skull dorsal surface  1. dorsal margins of orbits upturned (prominent along the orbital medial margin in dorsal view, with the frontal interorbital margins being upturned)  2. dorsal and posterior margins are upturned (the frontal lateral process anterior margins are also upturned) |
| 141 | **Circumorbital ventral margin, shape:**  *Salas-Gismondi et al. (2016, ch. 138 modified); Smith et al. (in review, ds 1, ch. 123); Ősi et al. (in review, ds 1, ch. 136).*  *State (1) occurs in* Vectisuchus*,* Sarcosuchus*,* Gavialis*.*  Chalawan *has evidence of the dorsal medial margin being upturned, but the anterior margins of the orbits are not preserved (Martin et al., 2014)*.  *State (1) is caused by the ‘upturning’ of the preorbital bones (in particular the lachrymals), changing the shape of the anterior orbit margin. As shown by Salas-Gismondi et al. (2016) the accumulation of characters relating to orbital ‘telescoping’ is gradual, thus not all taxa will score for all character states relating to this morphofunctional complex.*  *This character helps to quantify the ‘telescoped’ orbit morphology.*  0. ventral margin of the orbit is either concave or sub-straight  1. ventral margin of the orbit has a prominent notch |
| 142 | **Orbit, anterodorsal margin and the lachrymal:**  *Young & Andrade (2009, ch. 124 part); Young et al. (2011, ch. 124 part); Young et al. (2013a, ch. 62 part); Young et al. (2012, ch. 71); Young (2014, ch. 73); Young et al. (2016, ds 2, ch. 88); Ristevski et al. (2018, ds 2, ch. 120); Smith et al. (in review, ds 1, ch. 124); Ősi et al. (in review, ds 1, ch. 137).*  *In Thalattosuchia, state (1) is a putative autapomorphy of* Teleidosaurus calvadosii  0. lachrymal is excluded from the orbit anterodorsal margin  1. lachrymal reaches the orbit anterodorsal margin |
| 143 | **Orbit, posterodorsal margin and the postorbital:**  *Young & Andrade (2009, ch. 124 part); Young et al. (2011, ch. 124 part); Young et al. (2013a, ch. 62 part); Young et al. (2012, ch. 72); Young (2014, ch. 74); Young et al. (2016, ds 2, ch. 89); Ristevski et al. (2018, ds 2, ch. 121); Smith et al. (in review, ds 1, ch. 125); Ősi et al. (in review, ds 1, ch. 138).*  *In Thalattosuchia, state (1) is a putative apomorphy of the clade* Teleidosaurus *+ Metriorhynchidae*  0. postorbital is excluded from the orbit posterodorsal margin  1. postorbital reaches the orbit posterodorsal margin |
| 144 | **Orbit, anteroventral margin and the lachrymal:**  *Young & Andrade (2009, ch. 95 part); Young et al. (2011, ch. 95 part); Young et al. (2013a, ch. 63 part); Young et al. (2012, ch. 73); Young (2014, ch. 75); Young et al. (2016, ds 2, ch. 90); Ristevski et al. (2018, ds 2, ch. 122); Smith et al. (in review, ds 1, ch. 126); Ősi et al. (in review, ds 1, ch. 139).*  0. lachrymal is excluded from the orbit anteroventral margin  1. lachrymal reaches the orbit anteroventral margin |
| 145 | **Orbit, anterior margin and the jugal anterior process:**  *Ristevski et al. (2018, ds 2, ch. 123); Smith et al. (in review, ds 1, ch. 127); Ősi et al. (in review, ds 1, ch. 140).*  *State (1) is a putative apomorphy of* Goniopholis *and* Anteophthalmosuchus.  0. the jugal anterior process does not contribute to the anterior margin of the orbit  1. the jugal anterior process, along with the lachrymal, forms the anterior margin of the orbit.  Note that the broad anterior expansion of the jugal anterior process only occurs in *Goniopholis*, as *Anteophthalmosuchus* has a narrow jugal anterior process. |
| 146 | **Orbit, anterior margin and the broadening of the jugal anterior process:**  *Ristevski et al. (2018, ds 2, ch. 124); Smith et al. (in review, ds 1, ch. 128); Ősi et al. (in review, ds 1, ch. 141).*  *State (1) is a putative apomorphy of* Goniopholis  0. the jugal anterior process does not help form the anterior margin of the orbit, or as in *Anteophthalmosuchus*, it does help for the anterior margin of the orbit – but the jugal anterior process is still narrow  1. the jugal anterior process, along with the lachrymal, forms the anterior margin of the orbit, but it is distinctly broad dorsoventrally – expanded having a broad contact with the lachrymal dorsally and the maxilla anteriorly, much more so than in other derived goniopholidids. |
| 147 | **Orbit, posteroventral margin and the postorbital:**  *Young & Andrade (2009, ch. 95 part); Young et al. (2011, ch. 95 part); Young et al. (2013a, ch. 63 part); Young et al. (2012, ch. 74); Young (2014, ch. 76); Young et al. (2016, ds 2, ch. 91); Ristevski et al. (2018, ds 2, ch. 125); Smith et al. (in review, ds 1, ch. 129); Ősi et al. (in review, ds 1, ch. 142).*  *In Thalattosuchia, state (1) occurs in basal teleosauroids (*Steneosaurus brevior, *the Chinese skull referred to* Peipehsuchus teleorhinus, Platysuchus multiscrobiculatus *and* Teleosaurus cadomensis). *Note that some dorsoventral crushed skulls also look as though they have state (1), e.g.* S. bollensis.  0. postorbital is excluded from the orbit posteroventral margin, or only present in the posteroventral margin  1. postorbital reaches the orbit posteroventral margin (with the postorbital overlapping the jugal), and extensively forms part of the orbit ventral margin (in some instances excluding the jugal) |
| 148 | **Orbit, ventral margin and the jugal:**  *Mueller-Töwe (2006, ch. 139 modified);* *Young & Andrade (2009, ch. 95 part); Andrade et al. (2011, ch. 171 modified); Young et al. (2011, ch. 95 part); Young et al. (2013a, ch. 63 part); Young et al. (2012, ch. 75); Young (2014, ch. 77); Young et al. (2016, ds 2, ch. 92); Ristevski et al. (2018, ds 2, ch. 126); Smith et al. (in review, ds 1, ch. 130); Ősi et al. (in review, ds 1, ch. 143).*  *In Thalattosuchia, state (1) is a putative autapomorphy of* Platysuchus multiscrobiculatus  0. jugal participates in the orbit ventral margin  1. jugal excluded from the orbit by lachrymal-postorbital contact |
| 149 | **Supraorbital notch in dorsal view, deeply excavated creating an approximately semi-circular shape, resulting in the frontal being broadly exposed along the lateral margin of the orbits: (*)**  *Young et al. (2016, ds 2, ch. 93); Ristevski et al. (2018, ds 2, ch. 127); Smith et al. (in review, ds 1, ch. 131); Ősi et al. (in review, ds 1, ch. 144).*  *State (1) is a putative apomorphy of a subclade within Rhacheosaurini.*  *This character is not applicable for non-metriorhynchids, due to the unique formation of the supraorbital notch in Metriorhynchidae.*  0. absent  1. present |
| 150 | **Supraorbital notch in dorsal view, very small, being a tight "U"-shape, created by the prefrontal being expanded posteriorly. This results in the prefrontal making a larger contribution to the orbit dorsal margin and the frontal contribution to the orbit dorsal margin is greatly reduced, and in some taxa being excluded from the centre of the orbital dorsal margin: (*)**  *Young et al. (2016, ds 2, ch. 94); Ristevski et al. (2018, ds 2, ch. 128); Smith et al. (in review, ds 1, ch. 132); Ősi et al. (in review, ds 1, ch. 145).*  *State (1) is occurs in* Metriorhynchus palpebrosus*,* Cricosaurus saltillensis *and* C. macrospondylus.  *This character is not applicable for non-metriorhynchids, due to the unique formation of the supraorbital notch in Metriorhynchidae.*  0. absent  1. present |
| 151 | **Palpebrals, presence and number:**  *Clark (1994, ch. 65 modified); Young (2006, ch. 52 modified); Turner & Buckley (2008, ch. 65);* *Wilkinson et al. (2008, ch. 17 modified); Young & Andrade (2009, ch. 17 modified); Andrade et al. (2011, ch. 186); Young et al. (2011, ch. 17 modified); Young et al. (2013a, ch. 64 modified); Young et al. (2012, ch. 76 modified); Young (2014, ch. 78 modified); Young et al. (2016, ds2, ch. 95 modified); Ristevski et al. (2018, ds 2, ch. 129); Smith et al. (in review, ds 1, ch. 133); Ősi et al. (in review, ds 1, ch. 146).*  *Modified to exclude information about size, which can be sampled as a separate character. The presence and morphology of palpebrals is here considered to be highly devious within the analysis, always poorly sampled and including assumptions (e.g., putative fusion with prefrontals X putative loss in thalattosuchians). Preservation and incomplete descriptions contribute to a poor use of information as a character. Scores were considered only for taxa that actually show meaningful information. The putative absence of palpebrals in thalattosuchians has long been assumed (e.g., Fraas, 1901; Andrews, 1913), but it is actually not possible to exclude that this element may be deeply fused with prefrontal, leading to this modified version of state (0).*  *Can be determined by the sutural contacts along the periorbital margin.*  0. absent, or (anterior) palpebral is deeply fused with prefrontal  1. one large (anterior) palpebral present  2. two large palpebrals (anterior and posterior) present |
| 152 | **Orbits, presence of sclerotic ossicles (composing the sclerotic ring):**  *Young (2006, ch. 4); Wilkinson et al. (2008, ch. 19); Young & Andrade (2009, ch. 19); Andrade et al. (2011, ch. 159); Young et al. (2011, ch. 19); Young et al. (2013a, ch. 65); Young et al. (2012, ch. 77); Young (2014, ch. 79); Young et al. (2016, ds 2, ch. 96); Ristevski et al. (2018, ds 2, ch. 130); Smith et al. (in review, ds 1, ch. 134); Ősi et al. (in review, ds 1, ch. 147).*  *Within Thalattosuchia, state (1) is a putative apomorphy of* Pelagosaurus *+ Metriorhynchidae.*  0. absent  1. present |
| 153 | **Jugal, width of anterior process relative to posterior process:**  *Young & Andrade (2009, ch. 111); Young et al. (2011, ch. 111); Young et al. (2013a, ch. 66); Young et al. (2012, ch. 78); Young (2014, ch. 80); Young et al. (2016, ds 2, ch. 97); Ristevski et al. (2018, ds 2, ch. 131); Smith et al. (in review, ds 1, ch. 135); Ősi et al. (in review, ds 1, ch. 148).*  0. subequal  1. about twice as broad |
| 154 | **Jugal, anterior process is sigmoidal with a noticeable convexity along its dorsal margin:**  *Ristevski et al. (2018, ds 2, ch. 132); Smith et al. (in review, ds 1, ch. 136); Ősi et al. (in review, ds 1, ch. 149).*  *State (1) is found in* Dakosaurus *+ the Vaches Noire Dakosaur*.  0. absent  1. present |
| 155 | **Jugal, extends anteriorly in front of the prefrontal:**  *Young & Andrade (2009, ch. 94); Young et al. (2011, ch. 94); Young et al. (2013a, ch. 67); Young et al. (2012, ch. 79); Young (2014, ch. 81); Young et al. (2016, ds 2, ch. 98); Ristevski et al. (2018, ds 2, ch. 133); Smith et al. (in review, ds 1, ch. 137); Ősi et al. (in review, ds 1, ch. 150).*  0. no  1. yes |
| 156 | **Postorbital bar, inclination:**  *Jouve et al. (2008, ch. 35 modified); Young & Andrade (2009, ch. 85 modified); Hastings et al. (2010, ch. 50 modified); Young et al. (2011, ch. 85 modified); Young et al. (2013a, ch. 68 modified); Young et al. (2012, ch. 80 modified); Young (2014, ch. 82 modified); Ristevski et al. (2018, ds 2, ch. 134); Smith et al. (in review, ds 1, ch. 138); Ősi et al. (in review, ds 1, ch. 151).*  0. strongly anterodorsally inclined  1. slightly anterodorsally inclined  2. nearly vertical  3. posterodorsally inclined |
| 157 | **Jugal, well-developed (i.e. greatly enlarged) foramen on the anterior ramus:**  *Ristevski et al. (2018, ds 2, ch. 135); Smith et al. (in review, ds 1, ch. 139); Ősi et al. (in review, ds 1, ch. 152).*  *State (1) occurs in derived dyrosarids.*  0. no  1. yes |
| 158 | **Postfrontal:**  *Nesbitt (2011, ch. 44); Young et al. (2012, ch. 81); Young (2014, ch. 83); Young et al. (2016, ds 2, ch. 100); Ristevski et al. (2018, ds 2, ch. 136); Smith et al. (in review, ds 1, ch. 140); Ősi et al. (in review, ds 1, ch. 153).*  *State (1) is a putative apomorphy of Crocodylomorpha.*  0. present  1. absent |
| 159 | **Postorbital bar, morphology of dorsal end:**  *Young & Andrade (2009, ch. 90); Young et al. (2011, ch. 90); Young et al. (2013a, ch. 69); Young et al. (2012, ch. 82); Young (2014, ch. 84); Young et al. (2016, ds 2, ch. 101); Ristevski et al. (2018, ds 2, ch. 137); Smith et al. (in review, ds 1, ch. 141); Ősi et al. (in review, ds 1, ch. 154).*  0. dorsal end of the postorbital bar broadens dorsally, continuous with dorsal part of the postorbital  1. dorsal part of the postorbital bar constricted, distinct from the dorsal part of the postorbital |
| 160 | **Postorbital bar (postorbital), presence of a vascular opening at the lateral edge of the bar, close to the dorsal surface of the postorbital:**  *Clark (1994, ch. 27);* *Young & Andrade (2009, ch. 114); Andrade et al. (2011, ch. 202); Young et al. (2011, ch. 114); Young et al. (2013a, ch. 70); Young et al. (2012, ch. 83); Young (2014, ch. 85); Young et al. (2016, ds 2, ch. 102); Ristevski et al. (2018, ds 2, ch. 138); Smith et al. (in review, ds 1, ch. 142); Ősi et al. (in review, ds 1, ch. 155).*  *Note that scoring of state (0) can be highly influenced by preservation.*  0. absent  1. present |
| 161 | **Postorbital bar, morphology of postorbital-jugal contact:**  *Wilkinson et al. (2008, ch. 35); Young & Andrade (2009, ch. 35); Young et al. (2011, ch. 35); Young et al. (2013a, ch. 71); Young et al. (2012, ch. 84); Young (2014, ch. 86); Young et al. (2016, ds 2, ch. 103); Ristevski et al. (2018, ds 2, ch. 139); Smith et al. (in review, ds 1, ch. 143) Ősi et al. (in review, ds 1, ch. 156).*  0. postorbital medial to jugal  1. postorbital lateral to jugal |
| 162 | **Postorbital bar, structure:**  *Clark (1994, ch. 26 modified); Wilkinson et al. (2008, ch. 36 modified); Young & Andrade (2009, ch. 36 modified); Young et al. (2011, ch. 36 modified); Young et al. (2013a, ch. 72 modified); Young et al. (2012, ch. 85 modified); Young (2014, ch. 87 modified); Young et al. (2016, ds 2, ch. 104 modified); Ristevski et al. (2018, ds 2, ch. 140); Smith et al. (in review, ds 1, ch. 144); Ősi et al. (in review, ds 1, ch. 157).*  *State (1) occurs in Metasuchia.*  *State (2) describes the flattened morphology of tethysuchians.*  0. dermal bar that is either not columnal or transversely flattened  1. subdermal bar that is distinctly columnar and cylindrical or oval-shaped  2. subdermal bar that is distinctly columnar and transversely flattened |
| 163 | **Postorbital bar, composition of lateral surface:**  *Gasparini et al. (2006, ch. 244);* *Andrade et al. (2011, ch. 199); Ristevski et al. (2018, ds 2, ch. 141); Smith et al. (in review, ds 1, ch. 145); Ősi et al. (in review, ds 1, ch. 158).*  *State (1) is putative apomorphy of Thalattosuchia*  0. lateral surface formed by the postorbital and jugal  1. lateral surface formed by solely by the postorbital, with the jugal only exposed on the medial face of the bar |
| 164 | **Quadratojugal-postorbital, contact:**  *Ortega et al. (2000, ch. 49); Nesbitt (2011, ch. 64); Young et al. (2016, ds 2, ch. 105); Ristevski et al. (2018, ds 2, ch. 142); Smith et al. (in review, ds 1, ch. 146); Ősi et al. (in review, ds 1, ch. 159).*  *State (1) is a putative apomorphy of Crocodyliformes.*  0. absent  1. present |
| 165 | **Infratemporal fenestra (= laterotemporal fenestra), in lateral view:**  *Young (2006, ch. 12); Wilkinson et al. (2008, ch. 32); Young & Andrade (2009, ch. 32); Young et al. (2011, ch. 32); Young et al. (2013a, ch. 73); Young et al. (2012, ch. 86); Young (2014, ch. 88); Young et al. (2016, ds 2, ch. 106); Ristevski et al. (2018, ds 2, ch. 143); Smith et al. (in review, ds 1, ch. 147); Ősi et al. (in review, ds 1, ch. 160).*  0. considerably longer in length than the orbit (greater than 25%)  1. equal/subequal in length than the orbit (± 10%)  2. shorter in length than the orbit (less than 25%) |
| 166 | **Quadratojugal, spine (= spina quadratojugalis):**  *Brochu (1999, ch. 114); Young & Andrade (2009, ch. 133); Young et al. (2011, ch. 133); Young et al. (2013a, ch. 74); Andrade et al. (2011, ch. 167 + 170). Young et al. (2012, ch. 87); Young (2014, ch. 89); Young et al. (2016, ds 2, ch. 107); Ristevski et al. (2018, ds 2, ch. 144); Smith et al. (in review, ds 1, ch. 148); Ősi et al. (in review, ds 1, ch. 161).*  0. absent  1. either small or low crest  2. prominent |

**Palate and perichoanal structures** (Ch. 167 – 188; 4.846% of characters)

*[palate contribution of the dermatocranium facial series (= os præmaxillare and os maxillare), and dermatocranium palatal series (= ossa palatina, ossa pterygoidea, ossa ectopterygoidea and ossa vomeria)]*

| # | Description |
| --- | --- |
| 167 | **Premaxillae, presence of a subelliptic naso-oral fossa (= incisive foramen, = fossa premaxillaris) at medial contact of ventral rami:**  *Brochu (1999, ch. 124 part);* *Andrade et al. (2011, ch. 66); Young et al. (2012, ch. 89 modified); Young (2014, ch. 91 part); Young et al. (2016, ds 2, ch. 109 modified); Ristevski et al. (2018, ds 2, ch. 145); Smith et al. (in review, ds 1, ch. 149); Ősi et al. (in review, ds 1, ch. 162).*  *When the palate does not close completely, the passage will involve both premaxilla and maxilla, assuming a diamond-shaped profile, with edges straight to irregular, but never rounded and smooth. When the palate is incompletely closed, it is most likely that the vomer is also exposed at the opening; however, the vomer may not be preserved; or may be covered by sediment and not evident. The use of 'sub-elliptic' allows that simple openings on the palatal surface, considered as non-homologous to the naso-oral fossa, to be scored as (0).*  0. absent, premaxillae fully in contact medially along the palate  1. present as a discrete fossa or foramen, less than half the greatest width of premaxillae  2. large, more than half the greatest width of premaxillae |
| 168 | **Premaxillae, shape of naso-oral fenestra (= incisive foramen): (*)**  *Young et al. (2016, ds 2, ch. 7 modified); Ristevski et al. (2018, ds 2, ch. 146); Smith et al. (in review, ds 1, ch. 150); Ősi et al. (in review, ds 1, ch. 163).*  *In Metriorhynchidae, state (1) occurs in* Torvoneustes, *Mr Passmore’s specimen* + ‘M.’ hastifer.  *This character is not applicable for taxa that lack the naso-oral fenestra.*  0. subcircular or longer than wide (but not an elongate oval)  1. elongate anteroposterior oval-shape (can be as long or longer than the premaxillary alveoli, but not as mediolaterally broad) |
| 169 | **Suborbital fenestrae, presence and size: (ORDERED)**  *Andrade et al. (2011, ch. 206); Ristevski et al. (2018, ds 1, ch. 206); Smith et al. (in review, ds 2, ch. 206); Ősi et al. (in review, ds 1, ch. 164).*  0. absent  1. present, much smaller than orbits  2. present, subequal or larger than orbits |
| 170 | **Suborbital fenestrae, shape of anterior border: (*)**  *Andrade & Bertini (2008, ch. 86);**Andrade et al. (2011, ch. 207); Ristevski et al. (2018, ds 1, ch. 207); Smith et al. (in review, ds 2, ch. 207); Ősi et al. (in review, ds 1, ch. 165).*  *The original scoring in Andrade & Bertini (2008) for* Malawisuchus *and* Candidodon *was state (1), but this could be due to taphonomic deformation, therefore both taxa should be scored as (?) until a detailed description is provided for each taxon.*  *Nonetheless, state (1) is present in Thalattosuchia.*  *This character is not applicable for taxa that lack suborbital fenestrae.*  0. rounded, smooth  1. in sharp angle, forming a notch, fissure-like |
| 171 | **Maxilla, palatal processes: (ORDERED)**  *Nesbitt (2011, ch. 32); Ristevski et al. (2018, ds 2, ch. 147); Smith et al. (in review, ds 1, ch. 151); Ősi et al. (in review, ds 1, ch. 166).*  *Character helps to quantify the development of the secondary palate.*  *State (2) occurs in crocodylomorphs.*  0. do not meet at the midline  1. meet at the midline  2. meet at the midline and expand anteriorly and posteriorly |
| 172 | **Maxilla, posterior margin of palatal processes contact with the anterior margin of palatine anterior processes:**  *Young et al. (2012, ch. 90 modified); Young (2014, ch. 92 modified); Young et al. (2016, ds 2, ch. 110 modified); Ristevski et al. (2018, ds 2, ch. 148); Smith et al. (in review, ds 1, ch. 152); Ősi et al. (in review, ds 1, ch. 167).*  *Character helps to quantify the development of the secondary palate.*  *State (1) occurs in Mesoeucrocodylia.*  *Note, for* Calsoyasuchus *we interpret the ‘primary choanae’ as maxillo-palatine fenestrae.*  0. the maxilla-palatine contact only along a margin medial to the alveolar row  1. the maxilla posterior palatal margin has an extensive contact with the palatine anterior palatal margin. This results in either the vomer being excluded from the palatal surface, or if maxillo-palatine fenestrae are present, the vomer is visible within. The maxillo-palatine contact forms a continuous surface as the two elements contact one another, or when maxillo-palatine fenestrae are present, the anterior-most region of the contact is interrupted. |
| 173 | **Palate canals, presence: (*)**  *Andrade et al. (2011, ch. 220); Ristevski et al. (2018, ds 2, ch. 149); Smith et al. (in review, ds 1, ch. 153); Ősi et al. (in review, ds 1, ch. 168).*  *State (1) is a putative apomorphy of Thalattosuchia.*  *This character is not applicable for taxa that lack maxillary and palatine palatal processes which meet along the skull midline.*  *Palate canals are a paired, parallel, elongated, tubular ducts connecting the internal nasal cavity to the oral cavity, through the palatines. The orientation is almost coincident with the horizontal plane and longitudinal axis, with very little deviation (0-5 degrees). The internal openings are located anterior to the internal end of the nasopharyngeal duct. The external openings are located at the anterior end of palatines and, because of its sub-horizontal orientation, they progress as paired shallow (but well-defined) gutter-like grooves through the palatine laminae of the maxillae, at least to mid-rostrum. In teleosauroids (the Chinese teleosauroid,* Steneosaurus leedsi, S. edwardsi, *specimens attributed to* Steneosaurus latifrons*) and basal metriorhynchoids (*Pelagosaurus typus *and* Eoneustes gaudryi*)* *these passages are located next to the medial line of the palate, very close to each other, while in Metriorhynchidae* *the grooves diverge anteriorly (e.g. see Andrews, 1913; Young et al. 2013). This anterior divergence is also seen in some well preserved teleosauroids (MTY pers. obs). It is unclear if these canals constitute passages for nerves, vessels, or gland ducts.*  *In specimens which have experienced dorsoventral compression, and/or are highly broken, these canals can be very hard to discern.*  0. absent  1. present |
| 174 | **Palatine, anterior extent of the palatine relative to the maxillary tooth row:**  *Young (2014, ch. 93); Young et al. (2016, ds 2, ch. 111); Ristevski et al. (2018, ds 2, ch. 150); Smith et al. (in review, ds 1, ch. 154); Ősi et al. (in review, ds 1, ch. 169).*  *State (5) is a putative autapomorphy of* Plesiosuchus manselii.  0. palatine anterior margin terminates level to 20th maxillary alveoli, or more distal alveoli  1. palatine anterior margin terminates level to 15th to 19th maxillary alveoli  2. palatine anterior margin terminates level to 11th to 14th maxillary alveoli  3. palatine anterior margin terminates level to 8th to 10th maxillary alveoli  4. palatine anterior margin terminates level to 5th to 7th maxillary alveoli  5. palatine anterior margin terminates level to 4th maxillary alveoli, or more anterior alveoli |
| 175 | **Palatine, anterior margin has a mid-line anterior process:**  *Wilkinson et al. (2008, ch. 6 part); Young & Andrade (2009, ch. 6 part); Young et al. (2011, ch. 6 part); Young et al. (2013a, ch. 76 part); Young et al. (2012, ch. 91); Young (2014, ch. 94); Young et al. (2016, ds 2, ch. 112); Ristevski et al. (2018, ds 2, ch. 151); Smith et al. (in review, ds 1, ch. 155); Ősi et al. (in review, ds 1, ch. 170).*  0. present  1. absent |
| 176 | **Palatine, mid-line anterior process shape, in palatal view: (*)**  *Wilkinson et al. (2008, ch. 6 part); Young & Andrade (2009, ch. 6 part); Young et al. (2011, ch. 6 part); Young et al. (2013a, ch. 76 part); Young et al. (2012, ch. 92); Young (2014, ch. 95); Young et al. (2016, ds 2, ch. 113); Ristevski et al. (2018, ds 2, ch. 152); Smith et al. (in review, ds 1, ch. 156); Ősi et al. (in review, ds 1, ch. 171).*  *This character is not applicable for taxa that lack mid-line palatine palatal processes.*  0. lateral margins of the mid-line anterior process converge: anteriorly orientated “V”-shape  1. lateral margins of the mid-line anterior process largely parallel: anteriorly orientated “U”-shape |
| 177 | **Palatine, anterior margin has two non-midline anterior processes:**  *Wilkinson et al. (2008, ch. 6 part); Young & Andrade (2009, ch. 6 part); Young et al. (2011, ch. 6 part); Young et al. (2013a, ch. 76 part); Young et al. (2012, ch. 93); Young (2014, ch. 96); Young et al. (2016, ds 2, ch. 114); Ristevski et al. (2018, ds 2, ch. 153); Smith et al. (in review, ds 1, ch. 157); Ősi et al. (in review, ds 1, ch. 172).*  *In Thalattosuchia, state (1) is a putative apomorphy of Metriorhynchinae.*  *In* Montealtosuchus *and* Hamadasuchus *the mid-line anterior process has a concave anterior margin, creating two “non-midline” processes.*  0. absent  1. present |
| 178 | **Palatine, at the suborbital fenestrae the palatine anterior margin curves anterolaterally towards it, creating two “small processes” projecting laterally:**  *Young & Andrade (2009, ch. 161); Young et al. (2011, ch. 161); Young et al. (2013a, ch. 77); Young et al. (2012, ch. 94); Young (2014, ch. 97); Young et al. (2016, ds 2, ch. 115); Ristevski et al. (2018, ds 2, ch. 154); Smith et al. (in review, ds 1, ch. 158); Ősi et al. (in review, ds 1, ch. 173).*  *This morphology is variably observed in derived neosuchians and eusuchians.*  0. absent  1. present |
| 179 | **Palate, presence of palatal shelves of palatines, and their relation with the narial passage: (ORDERED)**  *Clark (1994, ch. 37 part);* *Wilkinson et al. (2008, ch. 8 part); Young & Andrade (2009, ch. 8 part); Andrade et al. (2011, ch. 212); Young et al. (2011, ch. 8 part); Young et al. (2013a, ch. 78 part); Young et al. (2012, ch. 95 part); Pol et al. (2013, ch. 67 part); Young (2014, ch. 98 part); Young et al. (2016, ds 2, ch. 116 part); Leardi et al. (2017, ch. 67 part); Ristevski et al. (2018, ds 1, ch. 212; ds 2, ch. 155 part); Smith et al. (in review, ds 1 ch. 159 part; ds 2, ch. 212); Ősi et al. (in review, ds 1, ch. 174).*  *Character helps to quantify the development of the secondary palate.*  *State (2) occurs in Mesoeucrocodylia, and in some more basal taxa.*  *Note that in state (2) the palatal laminae may not be in contact for taxa with extensive maxillopalatine fenestrae and elongate choanae (e.g.* Eutretauranosuchus*).*  0. palatal shelves of palatine absent, narial passage only bounded dorsally, by the pterygoid  1. narial passage at least partially bounded by palatal shelves of the palatine, laterally, creating the choanal grove  2. narial passage at least mostly bounded by palatal shelves of the palatine, laterally and ventrally, forming the nasopharyngeal duct |
| 180 | **Palatine, presence of a posterior extension to the choanae:**  *Jouve et al. (2005b, ch. 4); Jouve et al. (2008, ch. 4); Hastings et al. (2010, ch. 61); Ristevski et al. (2018, ds 2, ch. 156); Smith et al. (in review, ds 1, ch. 160); Ősi et al. (in review, ds 1, ch. 175).*  0. do not contact or only contact along the anterior margin  1. contact along the anterior and medial margins |
| 181 | **Palatine-pterygoid suture, lateral protrusions by palatine into the pterygoids:**  *Young & Andrade (2009, ch. 132); Young et al. (2011, ch. 132); Young et al. (2013a, ch. 80); Young et al. (2012, ch. 97); Young (2014, ch. 100); Young et al. (2016, ds 2, ch. 118); Ristevski et al. (2018, ds 2, ch. 157); Smith et al. (in review, ds 1, ch. 161); Ősi et al. (in review, ds 1, ch. 176).*  0. absent  1. present |
| 182 | **Ectopterygoid, presence of broad contact with palatine ramus of maxilla:**  *Ristevski et al. (2018, ds 2, ch. 158); Smith et al. (in review, ds 1, ch. 162); Ősi et al. (in review, ds 1, ch. 177).*  *Character based on Brochu (1997, ch. 91 modified); Andrade et al. (2011, ch. 253).*  *Basal forms within Sphenosuchia will show no (or very limited) contact between ectopterygoid and maxilla (0). As* Fruitachampsa *has a jugal-ectopterygoid contact (Clark, 2011), here we find this character to be a putative apomorphy of Mesoeucrocodylia +*Hsisosuchus*, rather than Crocodyliformes as in Andrade et al. (2011). Note,* Hsisosuchus *is not in our matrix, but scores as (1) in Andrade et al. (2011)*  *State (1) is putative apomorphy of Mesoeucrocodylia +* Hsisosuchus *(reversals in: French* Pholidosaurus*, and* Zoneait *+ Metriorhynchidae – the ectopterygoid solely contacts the jugal).*  *Note that in metriorhynchids the ectopterygoid is rarely preserved, and thus hard to score. It can be scored for* Metriorhynchus superciliosus *as it has what looks like the jugal-ectopterygoid articulation in NHMUK PV R 6860. However, the ectopterygoids are complete and in articulation in both* Zoneait *and* Maledictosuchus*.*  0. absent, ectopterygoid does not contact maxilla, or barely contacts its caudal end, medial to jugal  1. present |
| 183 | **Ectopterygoid, morphology of the distal ramus: (*)**  *Andrade et al. (2011, ch. 256); Ristevski et al. (2018, ds 1, ch. 256); Smith et al. (in review, ds 2, ch. 256); Ősi et al. (in review, ds 1, ch. 178).*  *Based on description by Pol & Apesteguia (2005: p. 8), where the subcylindrical profile of the distal ramus (1) was noted in* Araripesuchus buitreraensis*.*  *The condition is shared at least by other* Araripesuchus*,* Montealtosuchus *and a few other basal notosuchians.*  *This character is not applicable for taxa in which the ectopterygoid does not extend over the pterygoid wing.*  0. laminar, extending as a flattened sheet over the pterygoid wing  1. robust, extending as a rod over most of the pterygoid wing, with subcircular cross-section through most of its length |
| 184 | **Pterygoid flange, orientation (in palatal view):**  *Young et al. (2011, ch. 186); Young et al. (2013a, ch. 81); Young et al. (2012, ch. 98); Young (2014, ch. 101); Young et al. (2016, ds 2, ch. 119); Ristevski et al. (2018, ds 2, ch. 159); Smith et al. (in review, ds 1, ch. 163); Ősi et al. (in review, ds 1, ch. 179).*  0. horizontal  1. largely horizontal, but with a distinct posterolateral orientation  2. strongly orientated posteriorly |
| 185 | **Choanae, participation of pterygoid in the choanal border: (*)**  *Clark (1994, ch. 43 modified); Brochu (1999, ch. 71 modified); Jouve et al. (2005, ch. 4 modified); Turner & Buckley (2008, ch. 43 modified);**Young & Andrade (2009, ch. 131 + 139 modified); Andrade et al. (2011, ch. 242); Young et al. (2011, ch. 131 + 139 modified); Young et al. (2013a, ch. 79 + 82 modified); Young et al. (2012, ch. 96 + 99 modified); Young (2014 ch. 99 + 102); Young et al. (2016, ds 2, ch. 117 + 120 modified); Ristevski et al. (2018, ds 2, ch. 160); Smith et al. (in review, ds 1, ch. 164); Ősi et al. (in review, ds 1, ch. 180).*  *Note that the palatines may be excluded from the choanal border either in states (2) and (3), but the eusuchian condition is only achieved in state (3). State (2) corresponds directly to state (1) of Jouve et al. (2005, ch. 4), apomorphic for* Elosuchus, Terminonaris, Pholidosaurus purbeckensis *+ dyrosaurids.*  *Note that we do not consider* Koumpiodontosuchus *or* Isisfordia *to have the eusuchian condition. Our interpretation for* Isisfordia *follows Turner & Pritchard (2015), and* Koumpiodontosuchus *has a similar morphology (MTY pers. obs.).*  *This character is not applicable for taxa that lack the development of the secondary palate.*  0. pterygoid only bounds the posterior border of the choanae  1. pterygoid forms at least the posterior and lateral choanal borders  2. anterolateral rami of pterygoid embrace most of the choanae, but do not meet medially, at the anterior choanal border (either by the presence of palatine or ventral exposure and expansion of interchoanal septum)  3. anterolateral rami of pterygoid completely embrace the choanae, meeting medially at its anterior border (eusuchian choanae) |
| 186 | **Pterygoids, fusion posterior to choanae:**  *Clark (1994, ch. 41);* *Andrade et al. (2011, ch. 258); Ristevski et al. (2018, ds 2, ch. 161); Smith et al. (in review, ds 1, ch. 165); Ősi et al. (in review, ds 1, ch. 181).*  *State (1) is putative apomorphy of* Zosuchus *+ Mesoeucrocodylia.*  0. not fused  1. fused |
| 187 | **Choanal opening, in palatal view:**  *Wilkinson et al. (2008, ch. 9 part); Young & Andrade (2009, ch. 9 part); Young et al. (2011, ch. 187); Young et al. (2013a, ch. 83); Young et al. (2012, ch. 100); Young (2014, ch. 103); Young et al. (2016, ch. 121); Ristevski et al. (2018, ds 2, ch. 162); Smith et al. (in review, ds 1, ch. 166); Ősi et al. (in review, ds 1, ch. 182).*  *State (1) is observed in extant species.*  0. choanal opening orientated posteriorly, enclosed ventrally by the palatine and by either the pterygoid dorsally or the maxilla  1. choana opens into palate through a deep midline depression (choanal groove) |
| 188 | **Choana, anterior margin shape:**  *Wilkinson et al. (2008, ch. 9 part); Young & Andrade (2009, ch. 9 part); Young et al. (2011, ch. 9); Young et al. (2013a, ch. 84); Young et al. (2012, ch. 101); Young (2014, ch. 104); Young et al. (2016, ds 2, ch. 122); Ristevski et al. (2018, ds 2, ch. 163); Smith et al. (in review, ds 1, ch. 167); Ősi et al. (in review, ds 1, ch. 183).*  0. semicircular or elliptical  1. ‘V’-shaped with its base directed anteriorly  2. broad ‘U’-shaped with its base directed anteriorly  3. ‘W’-shaped with its base directed anteriorly |

**Occiptal** (Ch. 189 – 203; 3.304% of characters)

*[Partial chondrocranium = os supraoccipitale, ossa exoccipitalia + ossa opisthotica (= os otoccipitale)]*

| # | Description |
| --- | --- |
| 189 | **Occipital tuberosities:**  *Jouve (2005, ch. 1 modified), Jouve et al. (2005b, ch. 3 modified), Jouve et al. (2008, ch. 3 modified), Hastings et al. (2010, ch. 53 modified); Young et al. (2011, ch. 188); Young et al. (2013a, ch. 85); Young et al. (2012, ch. 102 modified); Young (2014, ch. 105 modified); Young et al. (2016, ds 2, ch. 123 modified); Ristevski et al. (2018, ds 2, ch. 164); Smith et al. (in review, ds 1, ch. 168); Ősi et al. (in review, ds 1, ch. 184).*  *State (1) occurs in teleosauroids, basal dyrosaurids and in the pholidosaurids* Sarcosuchus *and* Chalawan.  *State (2) occurs in most dyrosaurids and the teleosauroid* Steneosaurus heberti.  0. absent  1. small and reduced  2. large and well-developed |
| 190 | **Supraoccipital, presence:**  *Leardi et al. (2017, ch. 97); Ősi et al. (in review, ds 1, ch. 185).*  *State (1) occurs in Crocodylomorpha.*  0. fused with the exoccipital  1. present as a separate ossification |
| 191 | **Exoccipitals, presence of medial contact between both elements:**  *Clark (1994, ch. 62); Ortega et al. (2000, ch. 63);* *Gower (2002, ch. 19 modified); Andrade et al. (2011, ch. 270); Nesbitt (2011, ch. 126); Young et al. (2013a, ch. 86); Young et al. (2012, ch. 103); Young (2014, ch. 106); Tennant et al. (2016, ch. 198); Young et al. (2016, ds 2, ch. 124); Ristevski et al. (2018, ds 2, ch. 166); Smith et al. (in review, ds 1, ch. 170); Ősi et al. (in review, ds 1, ch. 187).*  *Can also be defined as the participation of supraoccipital in the foramen magnum.*  0. do not meet in midline  1. meet on the midline, dorsal to the basioccipital, excluding the supraoccipital from the foramen magnum |
| 192 | **Paroccipital processes of the opisthotic, orientation in occipital view:**  *Wilkinson et al. (2008, ch. 7); Young & Andrade (2009, ch. 7); Young et al. (2011, ch. 7); Young et al. (2013a, ch. 87); Young et al. (2012, ch. 104); Young (2014, ch. 107); Young et al. (2016, ds 2, ch. 125); Ristevski et al. (2018, ds 2, ch. 167); Smith et al. (in review, ds 1, ch. 171); Ősi et al. (in review, ds 1, ch. 188).*  *State (1) is a putative apomorphy of Rhacheosaurini.*  *State (2) is a putative apomorphy of Geosaurinae.*  *State (3) is a putative apomorphy of Dyrosauridae +* Pholidosaurus purbeckensis, *and also for* 'Dakosaurus' lissocephalus  0. horizontal  1. dorsolaterally orientated, at a 45 degree angle  2. ventral-edge horizontal, then terminal third sharply inclined dorsolaterally at a 45 degree angle  3. ventrally arched |
| 193 | **Paroccipital processes of the opisthotic, large ventrolateral region (i.e. the distal lower border is convex and bulges ventrally):**  *Young & Andrade (2009, ch. 116); Young et al. (2011, ch. 116); Young et al. (2013a, ch. 88); Young et al. (2012, ch. 105); Young (2014, ch. 108); Young et al. (2016, ds 2, ch. 126); Ristevski et al. (2018, ds 2, ch. 168); Smith et al. (in review, ds 1, ch. 172); Ősi et al. (in review, ds 1, ch. 189).*  *State (1) occurs in Crocodyliformes.*  0. present  1. absent |
| 194 | **Paroccipital process, overlap by the squamosal:**  *Young & Andrade (2009, ch. 119); Young et al. (2011, ch. 119); Young et al. (2013a, ch. 89); Young et al. (2012, ch. 106); Young (2014, ch. 109); Young et al. (2016, ds 2, ch. 127); Ristevski et al. (2018, ds 2, ch. 169); Smith et al. (in review, ds 1, ch. 173); Ősi et al. (in review, ds 1, ch. 190).*  0. small: the squamosal does not extend more posteriorly than the paroccipital process  1. large: it extends further posteriorly than the paroccipital process |
| 195 | **Foramen for cranial nerve XII (hypoglossal), position on occipit:**  *Wilkinson et al. (2008, ch. 10); Young & Andrade (2009, ch. 10); Young et al. (2011, ch. 10); Young et al. (2013a, ch. 90); Young et al. (2012, ch. 107); Young (2014, ch. 110); Young et al. (2016, ds 2, ch. 129); Ristevski et al. (2018, ds 2, ch. 170); Smith et al. (in review, ds 1, ch. 174); Ősi et al. (in review, ds 1, ch. 191).*  0. above the occipital condyle in line with the foramen magnum  1. below the foramen magnum |
| 196 | **Foramen for cranial nerve XII (hypoglossal), sits in the dorsomedial corner of ‘occipital fossae’ – concave depressions on the exoccipital on either side of the skull midline:**  *Ristevski et al. (2018, ds 2, ch. 171); Smith et al. (in review, ds 1, ch. 175); Ősi et al. (in review, ds 1, ch. 192).*  *State (1) occurs in* Torvoneustes.  0. absent  1. present |
| 197 | **Foramen for the internal carotid artery, external margin of the foramen is raised relative to the posterior face of the basioccipital, forming a sub-rectangular shape:**  *Ristevski et al. (2018, ds 2, ch. 172); Smith et al. (in review, ds 1, ch. 176); Ősi et al. (in review, ds 1, ch. 193).*  *State (1) occurs in* Torvoneustes.  0. no  1. yes |
| 198 | **Foramen for the internal carotid artery, size:**  *Wilkinson et al. (2008, ch. 11); Young & Andrade (2009, ch. 11); Young et al. (2011, ch. 11); Young et al. (2013a, ch. 91); Young et al. (2012, ch. 108); Young (2014, ch. 111); Young et al. (2016, ds 2, ch. 130); Ristevski et al. (2018, ds 2, ch. 173); Smith et al. (in review, ds 1, ch. 177); Ősi et al. (in review, ds 1, ch. 194).*  *State (1) is a putative apomorphy of* Pelagosaurus *+ Metriorhynchidae.*  0. similar in size to the openings for cranial nerves IX–XI  1. extremely enlarged |
| 199 | **Exoccipital, presence of descending flange ventral to subcapsular process:**  *Clark (1994, ch. 58);* *Andrade et al. (2011, ch. 273); Ristevski et al. (2018, ds 2, ch. 174); Smith et al. (in review, ds 1, ch. 178); Ősi et al. (in review, ds 1, ch. 195).*  *State (1) is putative apomorphy of protosuchids, but also present at least in* Araripesuchus tsangatsangana*.*  0. absent  1. present, laterally concave |
| 200 | **Exoccipital, extent of contact with the quadrate:**  *Clark (1994, ch. 48 modified + 51);* *Andrade et al. (2011, ch. 274); Ristevski et al. (2018, ds 2, ch. 175); Smith et al. (in review, ds 1, ch. 179); Ősi et al. (in review, ds 1, ch. 196).*  *Andrade et al. (2011) merged characters 48 and 51 of Clark (1994), into one ordered series, as both refer to the contact between exoccipitals and quadrate.*  *Following the present format, state (1) is a putative apomorphy of Gobiosuchidae + Mesoeucrocodylia.*  0. absent or narrow  1. broad contact present, stabilising the quadrate |
| 201 | **Exoccipital, presence of ventrolateral contact with the ventromedial part of quadrate:**  *Clark (1994, ch. 51 modified);* *Andrade et al. (2011, ch. 275); Ristevski et al. (2018, ds 1, ch. 275); Smith et al. (in review, ds 2, ch. 275); Ősi et al. (in review, ds 1, ch. 197).*  *Focus of character (51) modified from quadrate to exoccipital, to make evident its relation with character 48 (original numbers of Clark, 1994). Note that both characters may be fused into one ordered series, as they refer to the contact between both elements.*  *Following the present format, (1) is putative apomorphy of* Junngarsuchus *+ Crocodyliformes.*  0. absent, quadrate does not contact exoccipital  1. present, exoccipital and quadrate enclosing carotid artery and forming passage for cranial nerves IX-XI |
| 202 | **Exoccipital, participation in the occipital condyle:**  *Jouve (2004, ch. 96 modified); Jouve et al. (2005b, ch. 5 modified); Jouve et al. (2006, ch. 104 modified); Jouve et al. (2008, ch. 5 modified); Hastings et al. (2010, ch. 52 modified); Ristevski et al. (2018, ds 2, ch. 176); Smith et al. (in review, ds 1, ch. 180); Ősi et al. (in review, ds 1, ch. 198).*  *This scores the large contribution of the otocciptials to the occipital condyle seen in dyrosaurids, where the otoccipitals broadly contact the lateral margins of the condyle.*  0. slight to moderate  1. large, such that only a thin strip of the basioccipital is visible between the exoccipitals on the dorsal surface of the occipital condyle |
| 203 | **Occipital surface ventral to occipital condyle:**  *Young & Andrade (2009, ch. 143); Young et al. (2011, ch. 143); Young et al. (2013a, ch. 92); Young et al. (2012, ch. 109); Young (2014, ch. 112); Young et al. (2016, ds 2, ch. 131); Ristevski et al. (2018, ds 2, ch. 177); Smith et al. (in review, ds 1, ch. 181); Ősi et al. (in review, ds 1, ch. 199).*  *State (1) is a putative apomorphy of Crocodylia.*  0. slopes anteroventrally  1. sub-parallel or parallel to the transverse plane |

**Braincase, basicranium and suspensorium** (Ch. 204 – 228; 5.727% of characters)

*[Partial chondrocranium (= ossa laterosphenoidea, ossa prootica, os basioccipitale, os basisphenoideum); partial splanchnocranium (= ossa quadrata); pneumatic foramina; cranioquadrate canal]*

| # | Description |
| --- | --- |
| 204 | **Trigeminal fossa (=fossa for cranial nerve V), development on quadrate and laterosphenoid:**  *Young et al. (2013a, ch. 93); Young et al. (2012, ch. 110); Young (2014, ch. 113); Young et al. (2016, ds 2, ch. 132); Ristevski et al. (2018, ds 2, ch. 178); Smith et al. (in review, ds 1, ch. 182); Ősi et al. (in review, ds 1, ch. 200).*  *Character based on the discovery by Fernández et al. (2011)*.  *State (1) is a putative apomorphy of Metriorhynchidae.*  0. developed anteriorly and posteriorly to the trigeminal fenestra (i.e. fossa present on both laterosphenoid and quadrate)  1. fossa is mainly developed posteriorly to the fenestra (i.e. fossa present on quadrate) |
| 205 | **Laterosphenoids, sutures with parietal:**  *Hastings et al. (2010, ch. 63 modified); Ristevski et al. (2018, ds 2, ch. 179); Smith et al. (in review, ds 1, ch. 183); Ősi et al. (in review, ds 1, ch. 201).*  0. parallel to the skull table  1. descends posteriorly, relative to the skull table |
| 206 | **Laterosphenoids, fossae for the *m. pseudotemporalis superficialis*:**  *Young et al. (2013a, ch. 94 modified); Young et al. (2012, ch. 111 modified); Young (2014, ch. 114 modified); Young et al. (2016, ds 2, ch. 133 modified); Ristevski et al. (2018, ds 2, ch. 180); Smith et al. (in review, ds 1, ch. 184); Ősi et al. (in review, ds 1, ch. 202).*  *Character based upon data from Holliday & Witmer (2009) and Fernández et al. (2011).*  *State (1) is a putative apomorphy of Metasuchia.*  0. presence of a *pseudotemporalis* fossa on the dorsal surface of the laterosphenoid, and/or continuing on to the frontal  1. either an absence of the pseudotemporalis fossa on the dorsal surface of the laterosphenoid (i.e. only the *m. adductor mandibulae externus profundus* is within the supratemporal fenestra), or scorable by the presence of the fossa on the posteroventral surface of the laterosphenoid (the “subfenestral position”) |
| 207 | **Parasphenoid ridge/rostrum (?), in palatal view:**  *Wilkinson et al. (2008, ch. 4); Young & Andrade (2009, ch. 4); Young et al. (2011, ch. 4); Young et al. (2013a, ch. 95); Young et al. (2012, ch. 112); Young (2014, ch. 115); Young et al. (2016, ds 2, ch. 134); Ristevski et al. (2018, ds 2, ch. 181); Smith et al. (in review, ds 1, ch. 185); Ősi et al. (in review, ds 1, ch. 203).*  *The homology of this ridge is unknown. Andrews (1913) considered the midline pterygoid ridge to be the parasphenoid. However, the pterygoids are poorly known for metriorhynchids, and we cannot discount this as a purely pterygoid structure. Until this structure has undergone CT scanning we will provisionally use the term parasphenoid.*  0. not visible  1. forms a midline ridge along the pterygoids |
| 208 | **Basisphenoid, paired ridges located medially on the ventral surface:**  *Young & Andrade (2009, ch. 83); Young et al. (2011, ch. 83); Young et al. (2013a, ch. 96); Young et al. (2012, ch. 113); Young (2014, ch. 116); Young et al. (2016, ds 2, ch. 135); Ristevski et al. (2018, ds 2, ch. 182); Smith et al. (in review, ds 1, ch. 186); Ősi et al. (in review, ds 1, ch. 204).*  *State (1) occurs in Teleosauroidea.*  0. absent  1. present |
| 209 | **Basisphenoid, ventral exposure in adults and young individuals, but not immature or hatchlings: (ORDERED)**  *Clark (1994, ch. 55 revised+ 56 revised); Ortega et al. (2000, ch. 68 modified); Young & Andrade (2009, ch. 87 modified); Andrade et al. (2011, ch. 286 modified); Young et al. (2011, ch. 87 modified); Young et al. (2013a, ch. 97 modified); Young et al. (2012, ch. 114 modified); Young (2014, ch. 117 modified); Young et al. (2016, ds 2, ch. 136 modified); Ristevski et al. (2018, ds 2, ch. 183); Smith et al. (in review, ds 1, ch. 187); Ősi et al. (in review, ds 1, ch. 205).*  *Original characters by Clark (1994, ch. 55-56) actually reflect the size of basisphenoid and here were combined into one character by Andrade et al. (2011). Note disagreement in the scorings from previous works, e.g., Clark (1994) considered thalattosuchians as (0) and Turner & Buckley (2008) considers them as (1); Turner & Buckley (2008) considers* Mahajangasuchus *as (2), whereas here it is considered as (1). Most authors consider "Sphenosuchians" as (1), but the basisphenoid is well exposed at least in* Gracilisuchus*,* Sphenosuchus *and possibly in* Pseudhesperosuchus *(see Bonaparte, 1971; Romer, 1972; Walker, 1990). Further scorings by Turner & Buckley (2008).*  *Note Ristevsski et al. (2018, ds 2) re-ordered the character from Andrade et al. (2011). State (2) is now (0), and state (0) is now (2). State (1) is unaffected.*  0. ample surface exposed ventrally, basisphenoid at least as long as the basioccipital, or longer  1. well-exposed, although basisphenoid surface clearly smaller than basioccipital surface  2. extremely reduced surface, exposed as a transversal slit, almost obliterated ventrally by the basioccipital and the pterygoids |
| 210 | **Basisphenoid, exposure anterior to the quadrates in palatal view:**  *Wilkinson et al. (2008, ch. 5 modified); Young & Andrade (2009, ch. 5 modified); Young et al. (2011, ch. 5 modified); Young et al. (2013a, ch. 98); Young et al. (2012, ch. 115); Young (2014, ch. 118); Young et al. (2016, ds 2, ch. 137); Ristevski et al. (2018, ds 2, ch. 184); Smith et al. (in review, ds 1, ch. 188); Ősi et al. (in review, ds 1, ch. 206).*  *State (1) is a putative apomorphy of a teleosauroid subclade. This character state is caused by the posterior expansion of the pterygoid’s posterior margin, so that the anterior portion of the quadrates is obscured, as are the lateral margins of the basisphenoid. However, there is a distinct basisphenoid ‘rostrum’ that in some taxa continue to bifurcate the ptergoids anteriorly. This morphology is not observed in* Teleosaurus cadomensis*,* *the skull referred to* Peipehsuchus teleorhinus, Steneosaurus brevior, Pelagosaurus typus *or Metriorhynchidae.*  0. basisphenoid terminates approximately level to the anterior extent of the quadrates  1. basisphenoid ‘rostrum’/cultriform process exposed along the palatal surface anterior to the quadrates, continuing to bifurcate the pterygoids |
| 211 | **Basisphenoid rostrum (= cultriform process):**  *Jouve (2005, ch. 2), Jouve et al. (2005b, ch. 7), Jouve et al. (2008, ch. 7), Hastings et al. (2010, ch. 54); Ristevski et al. (2018, ds 2, ch. 185); Smith et al. (in review, ds 1, ch. 189); Ősi et al. (in review, ds 1, ch. 207).*  *State (1) is observed in some derived dyrosaurids. This character is not homologous with the anterior projection of the basisphenoid oberserved in teleosauroids. Here, the basisphenoid projects anteriorly between the pterygoids and laterosphenoids, rather than bifurcating the former.*  0. short  1. extremely long anteriorly |
| 212 | **Basisphenoid, exposure ventral to the basioccipital at maturity in occipital aspect:**  *Young & Andrade (2009, ch. 144); Young et al. (2011, ch. 144); Young et al. (2013a, ch. 99); Young et al. (2012, ch. 116); Young (2014, ch. 119); Young et al. (2016, ds 2, ch. 138); Ristevski et al. (2018, ds 2, ch. 186); Smith et al. (in review, ds 1, ch. 190); Ősi et al. (in review, ds 1, ch. 208).*  *State (1) is a putative apomorphy of Eusuchia.*  0. absent, pterygoid dorsoventrally short ventral to medial pharyngeal opening (= “medial Eustachain foramen”)  1. present, pterygoid dorsoventrally tall ventral to medial pharyngeal opening |
| 213 | **Basisphenoid, development of basipterygoid processes:**  *Clark (1994, ch. 54 revised);* *Andrade et al. (2011, ch. 289 modified); Ristevski et al. (2018, ds 2, ch. 187); Smith et al. (in review, ds 1, ch. 191); Ősi et al. (in review, ds 1, ch. 209).*  *State (1) occurs in* *Crocodyliformes.*  0. prominent, forming a movable joint with pterygoid, and with basisphenoid joint suturally closed  1. small or absent |
| 214 | **Basioccipital, single wide rugosity oriented anteroposteriorly along the midline of the ventral surface of the occipital condyle:**  *Hastings et al. (2010, ch. 55 modified); Ristevski et al. (2018, ds 2, ch. 188); Smith et al. (in review, ds 1, ch. 192); Ősi et al. (in review, ds 1, ch. 210).*  0. absent  1. present |
| 215 | **Basioccipial, presence of tuberosities (= basal tubera):**  *Clark (1994, ch. 57); Lauprasert et al. (2007, ch. 46);* *Young & Andrade (2009, ch. 151); Andrade et al. (2011, ch. 288); Young et al. (2011, ch. 151); Young et al. (2013a, ch. 100); Young et al. (2012, ch. 117); Young (2014, ch. 120); Young et al. (2016, ds 2, ch. 139); Ristevski et al. (2018, ds 2, ch. 189); Smith et al. (in review, ds 1, ch. 193); Ősi et al. (in review, ds 1, ch. 211).*  *State (1) occurs in longirostrine taxa.*  0. reduced  1. large and pendulous |
| 216 | **Basioccipital tuberosities, in ventral view:**  *Hastings et al. (2010, ch. 56 modified); Ristevski et al. (2018, ds 2, ch. 190); Smith et al. (in review, ds 1, ch. 194); Ősi et al. (in review, ds 1, ch. 212).*  0. oblong-shaped  1. 'V'-shaped or tear-drop shaped |
| 217 | **Paired grooves along ventral surface, extending from base of the occipital condyle to the basioccipital tuberosities:**  *Hastings et al. (2010, ch. 57 modified); Ristevski et al. (2018, ds 2, ch. 191); Smith et al. (in review, ds 1, ch. 195); Ősi et al. (in review, ds 1, ch. 213).*  0. absent  1. present |
| 218 | **Ventral part of the basioccipital:**  *Jouve et al. (2005b, ch. 13), Jouve et al. (2008, ch. 13), Hastings et al. (2010, ch. 59); Ristevski et al. (2018, ds 2, ch. 192); Smith et al. (in review, ds 1, ch. 196); Ősi et al. (in review, ds 1, ch. 214).*  0. vertical, largely visible in occipital view  1. strongly inclined, weakly visible in occipital view |
| 219 | **Quadrate, prominent crest on dorsal surface of distal quadrate extending proximally to lateral extent of quadrate–exoccipital contact:**  *Young & Andrade (2009, ch. 101); Young et al. (2011, ch. 101); Young et al. (2013a, ch. 101); Young et al. (2012, ch. 118); Young (2014, ch. 121); Young et al. (2016, ds 2, ch. 140); Ristevski et al. (2018, ds 2, ch. 193); Smith et al. (in review, ds 1, ch. 197); Ősi et al. (in review, ds 1, ch. 215).*  *State (1) occurs in Metasuchia (with reversals, such as in Crocodylia).*  0. absent  1. present |
| 220 | **Quadrate, contact with the proötics:**  *Clark et al. (2000, ch. 14); Clark & Sues (2002, ch. 15); Sues et al. (2003, ch. 15); Clark et al. (2004, ch. 15); Nesbitt (2011, ch. 76); Pol et al. (2013, ch. 15); Young et al. (2016, ds 2, ch. 141); Leardi et al. (2017, ch. 15); Ristevski et al. (in review, ds 2, ch. 194); Smith et al. (2018, ds 1, ch. 198); Ősi et al. (in review, ds 1, ch. 216).*  *State (1) is a putative apomorphy of Crocodylomorpha.*  0. does not contact the proötic  1. contacts the proötic |
| 221 | **Quadrate, articulation of dorsal head contact:**  *Clark (1994, ch. 47);* *Young & Andrade (2009, ch. 102 modified); Andrade et al. (2011, ch. 298); Young et al. (2011, ch. 102 modified); Young et al. (2013a, ch. 102 modified); Young et al. (2012, ch. 119 modified); Young (2014, ch. 122 modified); Young et al. (2016, ds 2, ch. 142); Ristevski et al. (2018, ds 2, ch. 195); Smith et al. (in review, ds 1, ch. 199); Ősi et al. (in review, ds 1, ch. 217).*  *State (1) is a putative apomorphy of* Junggarsuchus *+ Crocodyliformes.*  0. squamosal and exoccipital/opisthotic/otoccipital (can have medial contact with proötics and laterosphenoids)  1. proötic and laterosphenoid |
| 222 | **Quadrate, posterior margin:**  *Nesbitt (2011, ch. 77); Young et al. (2016, ds 2, ch. 143); Ristevski et al. (2018, ds 2, ch. 196); Smith et al. (in review, ds 1, ch. 200); Ősi et al. (in review, ds 1, ch. 218).*  *State (1) is a putative apomorphy of Metasuchia – note that the ventral/anteroventral margins of the distal ends of the paroccipital processes have a strong sutural contact with the quadrates.*  0. does not have a sutural contact with the paroccipital process of the opisthotic, or the anterior margin of the paroccipital process has a simple contact with the posterior margin of the quadrate  1. has a robust sutural contact with the paroccipital process of the opisthotic |
| 223 | **Quadrate, anteroventral process suturing to the braincase:**  *Young et al. (2013a, ch. 103 modified); Young et al. (2012, ch. 120 modified); Young (2014, ch. 123 modified); Young et al. (2016, ds 2, ch. 144 modified); Ristevski et al. (2018, ds 2, ch. 197); Smith et al. (in review, ds 1, ch. 201); Ősi et al. (in review, ds 1, ch. 219).*  *The scores for the contact of the anteroventral process (referred to as the ‘orbital’ and ‘pterygoid’ processes by different authors).*  *State (2) represents the ‘quadrate incompletely sutured to the braincase’ statement in* *Holliday & Witmer (2009), Jouve (2009) and Fernández et al. (2011)*.  *The current version of this character aims to quantify two trends: 1) the contact between the quadrate and the laterosphenoid (as part of the stabilisation of the crocodylomorph skull), and 2) the thalattosuchian modification of this trend. In Thalattosuchia, it appears as though the anteromedial region of this process no longer articulates with the lateral surface of the neurocranium, but it is still elongated enough to have, and seems to sit lateral to the laterosphenoid. Perhaps suggesting a soft-tissue contact.*  *State (1) occurs in Crocodyliformes.*  *State (2) occurs in Thalattosuchia.*  0. this process contacts the pterygoid, but little to no contact with the neurocranium  1. this process has extensive contact with the laterosphenoid, basisphenoid and pterygoid (i.e. stabilises the splanchnocranium with the palate and neurocranium)  2. this process is free of bony attachment along its anteromedial surface, but ventrally contacts the pterygoid. Process likely has a posteromedial contact with the basisphenoid, but is free of contact with the laterosphenoid |
| 224 | **Quadrate, distal articular surface separated into two condyles:**  *Young (2014, ch. 126); Young et al. (2016, ds 2, ch. 147); Ristevski et al. (2018, ds 2, ch. 200); Smith et al. (in review, ds 1, ch. 204); Ősi et al. (in review, ds 1, ch. 222).*  *State (1) is a putative apomorphy of Plesiosuchina.*  *Character can be scored if the articular is preserved, and no ridge that supports the intercondylar sulcus is present.*  0. yes  1. no |
| 225 | **Quadrate-quadratojugal, quadratojugal contributes to the upper jaw joint along with the quadrate (i.e. helps to form the lateral hemicondyle):**  *Jouve et al. (2005b, ch. 19 modified); Jouve et al. (2008, ch. 19modified); Hastings et al. (2010, ch. 60 modified); Ristevski et al. (2018, ds 2, ch. 201); Smith et al. (in review, ds 1, ch. 205); Ősi et al. (in review, ds 1, ch. 223).*  0. lateral hemicondyle soley formed by the quadrate  1. lateral hemicondyle has a quadratojugal contribution |
| 226 | **Fossa for the tympanic membrane, anterior extension:**  *Ristevski et al. (2018, ds 2, ch. 202); Smith et al. (in review, ds 1, ch. 206); Ősi et al. (in review, ds 1, ch. 224).*  *State (1) occurs in Notosuchia and Sebecia.*  *State (2) occurs in Neosuchia.*  0. limited to the squamosal  1. reaches the posterior margin of the postorbital  2. broadly exposed on the postorbital (covering the anterolateral margin)  3. crosses the postorbital and reaches the orbit |
| 227 | **Cranioquadrate canal, contact between the quadrate and exoccipital around the opening: (ORDERED)**  *Clark (1994, ch. 49 modified);* *Andrade et al. (2011, ch. 306 modified + ch. 308 modified); Ristevski et al. (2018, ds 2, ch. 203); Smith et al. (in review, ds 1, ch. 207); Ősi et al. (in review, ds 1, ch. 225).*  *Cranioquadrate canal (=quadratosquamosootoccipitalis, in Salisbury et al., 1999; or =quadratosquamosoexoccipitalis, in Delfino et al., 2008).*  *State (1) occurs in Hallopodidae* (*e.g.* Almadasuchus) *and Mesoeucrocodylia.*  *In derived forms the squamosal will also help enclose the cranioquadrate canal.*  *Contact between quadrate and exoccipital is extensive (2) in all crown crocodylians, but in all stem metasuchians this contact is feeble (1).*  0. absent (and the quadrate and exoccipital do not meet to enclose the cranioquadrate canal)  1. lateral contact between the quadrate and exoccipital is feeble, but these bones do meet to enclose the cranioquadrate canal  2. lateral contact between the quadrate and exoccipital is broad, and these bones do meet to enclose the cranioquadrate canal |
| 228 | **Cranioquadrate canal, bones enclosing:**  *Ristevski et al. (2018, ds 2, ch. 204); Smith et al. (in review, ds 1, ch. 208); Ősi et al. (in review, ds 1, ch. 226).*  *Scores for a similar morphology as Andrade et al. (2011, ch. 307), but with distinct differences.*  *Cranioquadrate canal does not imply in the presence of a passage, and therefore may be opened laterally. The canal is only considered absent (0) in basal crocodylomorphs and basal crocodyliformes.*  *Note at present state (0) here correlates with the state (0) in character quantifying the contact between the quadrate and exoccipital around the cranioquadrate canal. However, here a taxon with an enclosed cranioquadrate canal which does not have a squamosal participation would be scored as (0).*  *State (1) occurs in Thalattosuchia.*  *State (2) is common among goniopholidids and pholidosaurids.*  *State (3) occurs in Metasuchia, but with some losses (especially in Neosuchia).*  0. quadrate, squamosal and exoccipital do not enclose the cranioquadrate canal along its length  1. squamosal laterally encloses the cranioquadrate canal, the quadrate ventrally, and the exoccipital posteriorly, medially and partly ventrally encloses the canal. This results in the canal opening laterally and/or posterolaterally  2. quadrate and squamosal do not laterally enclose the cranioquadrate canal, and it is laterally exposed but still exits on the occipital surface. *This looks to be a modification of state (3), where there is no ossified lateral enclosure, resulting in the ‘open morphotype’*.  3. quadrate and squamosal laterally enclose the cranioquadrate canal, and the exoccipital helps enclose it dorsally. This results in the canal opening on the occipital surface |
| 229 | **Cranioquadrate canal, presence of a squamosal descending process separating the cranioquadrate canal from the external auditory meatus:**  *Ristevski et al. (2018, ds 2, ch. 205); Smith et al. (in review, ds 1, ch. 209); Ősi et al. (in review, ds 1, ch. 227).*  *State (1) occurs in thalattosuchians. Note that the* Teleosaurus cadomensis *specimen figured by Jouve (2009) had a broken squamosal descending lamina, and that the skull had been acid prepared. Here it is scored as (1).* Pelagosaurus typus *is also scored as (1), as the skull NHMUK PV OR 32599 is also acid prepared and many of the thin laminae are broken.*  0. absent, no clear separation of these structures  1. present, the cranioquadrate canal and the external auditory meatus are distinct openings, sharing a common wall (squamosal descending process) |

**Mandibular geometry** (Ch. 230 – 237; 1.762% of characters)

| # | Description |
| --- | --- |
| 230 | **Mandible geometry, relative positions of the dentary tooth-row and coronid process, and development of dorsal curvature of the posterior-end of the mandible:**  *Young et al. (2011, ch. 167); Young et al. (2013a, ch. 109); Young et al. (2012, ch. 127); Young (2014, ch. 131); Young et al. (2016, ds 2, ch. 153); Ristevski et al. (2018, ds 2, ch. 207); Smith et al. (in review, ds 1, ch. 211); Ősi et al. (in review, ds 1, ch. 229).*  *State (1) is a putative apomorphy of Metriorhynchidae.*  *Quantifies the incipient increase of gape at the base of Metriorhynchidae.*  0. gentle curvature in the dorsal margin of the mandible, from the coronoid process to the end of the tooth-row  1. strong curvature, raising the coronoid process considerably above the tooth-row |
| 231 | **Mandible geometry, relative positions of coronoid process, retroarticular process and glenoid fossa:**  *Young et al. (2011, ch. 168); Young et al. (2013a, ch. 110); Young et al. (2012, ch. 128); Young (2014, ch. 132); Young et al. (2016, ds 2, ch. 154); Ristevski et al. (2018, ds 2, ch. 208); Smith et al. (in review, ds 1, ch. 212); Ősi et al. (in review, ds 1, ch. 230).*  *State (1) is a putative apomorphy of Geosaurini.*  *This character quantifies the greater increase in gape associated with macrophagous geosaurines.*  0. coronoid process level to both the retroarticular process and glenoid fossa  1. coronoid process ventral to both the retroarticular process and glenoid fossa |
| 232 | **Mandibular rami, presence of a sharp dorsal inclination:**  *Ristevski et al. (2018, ds 2, ch. 209); Smith et al. (in review, ds 1, ch. 213); Ősi et al. (in review, ds 1, ch. 231).*  *State (1) is a putative apomorphy of Plesiosuchina.*  0. absent  1. present - immediately posterior to the mandibular symphysis the mandible sharply rises dorsally such that the ventral margin of the dentary (along with angular) is dorsally deflected (resulting in a distinct 'kink' along the mandibular ventral margin) |
| 233 | **Mandible, orientation of hemimandibles at their medial contact:**  *Andrade et al. (2011, ch. 320); Ristevski et al. (2018, ds 1, ch. 320); Smith et al. (in review, ds 2, ch. 320); Ősi et al. (in review, ds 1, ch. 232).*  0. evidently acute angle, hemimandibles meet at approximately 45 degrees of each other, or less  1. broad angle, hemimandibles meet at approximately 70 degrees of each other, or more |
| 234 | **Mandible, morphology of distal rami in dorsal/ventral views:**  *Andrade et al. (2011, ch. 321); Ristevski et al. (2018, ds 2, ch. 210); Smith et al. (in review, ds 1, ch. 214); Ősi et al. (in review, ds 1, ch. 233).*  *Note that the broad-Y shape in (1) is not the result of elongation of the symphysis (which is present, but not exclusively in these forms), but by the arched distal rami, meeting at mid-mandible.*  *State (1) is putative apomorphy of Notosuchidae + Sphagesauridae.*  0. distal rami mostly straight or poorly curved  1. distal rami strongly curved medially at mid-mandible, giving the mandible a broad-Y shape |
| 235 | **Mandible, ventral border at angular, in lateral view: (ORDERED)**  *Andrade et al. (2011, ch. 322); Ristevski et al. (2018, ds 1, ch. 322); Smith et al. (in review, ds 2, ch. 322); Ősi et al. (in review, ds 1, ch. 234).*  *This character, created by Andrade et al. (2011), is potentially co-dependent with Pol et al. (2012, ch. 280), which is not included here (see also Turner & Buckley 2008, ch. 280)*  *State (0) is based on descriptions by Woodward (1896), Price (1945) and Andrade & Bertini (2008b). State (2) is originally based on descriptions by Hooley (1907), Schwarz (2002) and Ősi et al. (2007).*  0. angular straight and mostly horizontal, or poorly curved, from the anterior to the posterior end  1. angular evidently (but gently) curved  2. angular abruptly curved, always below glenoid fossa, with mid-posterior sections of angular sub-vertical, facing posteriorly |
| 236 | **Mandible, morphology of ventral margin, in lateral view:**  *Andrade et al. (2011, ch. 323); Ristevski et al. (2018, ds 1, ch. 323); Smith et al. (in review, ds 2, ch. 323); Ősi et al. (in review, ds 1, ch. 235).*  *The triple contact between dentary, angular and surangular can be taken as reference, if mandibular fenestra is absent*  0. mandible is curved ventrally, with maximum curvature at anterior section of angular, below the mandibular fenestra (when present), or not curved at all  1. mandible is curved posteroventrally, with maximum curvature at posterior section of angular, below (or almost below) the mandibular glenoid fossa, usually posterior to mandibular fenestra (when present) |
| 237 | **Mandible, dorsal border at dentary-surangular contact, in lateral view:**  *Clark (1994, ch. 74); Sereno et al. (2003, ch. 41);* *Andrade et al. (2011, ch. 324); Ristevski et al. (2018, ds 1, ch. 324); Smith et al. (in review, ds 2, ch. 324); Ősi et al. (in review, ds 1, ch. 236).*  *State (2) is putative apomorphy of Notosuchidae + Sphagesauridae + Comahuesuchidae.*  0. mostly straight  1. gently arched dorsally  2. strongly arched dorsally |

**Mandible** (Ch. 238 – 269; 7.048% of characters)

*[Dermatocranium mandibular series (=* *ossa dentalia, ossa splenialia, ossa angularia, ossa supraangularia, ossa præarticularia, ossa coronoidea); and the mandibular contribution of the splanchnocranium (= ossa articularia and cartilagines meckeli)]*

| # | Description |
| --- | --- |
| 238 | **Anterior mandible (dentary), dorsal margin of the anterior portion compared to the dorsal margin of the posterior portion:**  *Nesbitt (2011, ch. 154); Young et al. (2012, ch. 129); Young (2014, ch. 133); Young et al. (2016, ds 2, ch. 155); Ristevski et al. (2018, ds 2, ch. 211); Smith et al. (in review, ds 1, ch. 215); Ősi et al. (in review, ds 1, ch. 237).*  0. horizontal (in the same plane)  1. ventrally deflected  2. dorsally expanded |
| 239 | **Anterior mandible (dentary), in dorsal or ventral view:**  *Young et al. (2011, ch. 181 modified); Young et al. (2013a, ch. 111 modified); Young et al. (2012, ch. 130 modified); Young (2014, ch. 135 modified); Young et al. (2016, ds 2, ch. 156 modified); Ristevski et al. (2018, ds 2, ch. 212); Smith et al. (in review, ds 1, ch. 216); Ősi et al. (in review, ds 1, ch. 238).*  *Note, Ristevski et al. (2018, ds 2) added two new character states. These where added to determine whether the ‘spatulate’ anterior dentary morphotypes would homologous.*  *State (1) occurs in most pholidosaurids, and in some dyrosaurids and eusuchians.*  *State (2) is a putative apomorphy of Teleosauroidea.*  *State (3) is a putative apomorphy of* Sarcosuchus *and* Chalawan.  0. outer margin converging towards tip or parallel  1. distinct spatulate shape, with the maximum transverse width at the D2 alveoli  2. distinct spatulate shape, with the maximum transverse width at the D3-D4 couplet  3. distinct spatulate shape, with the maximum transverse width at the D4 alevoli |
| 240 | **Anterior mandible (dentary), in dorsal or ventral view:**  *Young et al. (2016, ds 2, ch. 157 + 158); Ristevski et al. (2018, ds 2, ch. 213); Smith et al. (in review, ds 1, ch. 217); Ősi et al. (in review, ds 1, ch. 239).*  *State (1) occurs in basal dyrosaurids and tomistomine crocodyloids*.  *State (2) occurs in* Hamadasuchus, *Peirosauridae and* Baurusuchus.  *States (1) and (2) differ in that the ‘trowel’-shape has a shorter, broader and deeper symphyseal region; the anteriorly tapering maximal anterior width is more pronounced, and the width at the posterior symphyseal region is greater than the maximal anterior width.*  0. non-'gladius', or ‘trowel’-shaped  1. 'gladius'-shaped - i.e. a long symphyseal region with the anterior maximal width near the D3–D5 region, with the dentaries tapering anteriorly. Immediately posterior to the maximal width, the dentaries begin to narrow until they reach a minimal width, and begin expanding again. At the end of the symphyseal region the breadth is now wider than the anterior maximal width  2. 'trowel'-shaped - i.e. a moderate to short symphyseal region with the anterior maximal width near the D3–D5 region, with the dentaries tapering strongly anteriorly. Immediately posterior to the maximal width the dentaries begin to narrow until they reach a minimal width, and begin expanding again. At the end of the symphyseal region the breadth is either narrower or subequal to the anterior maximal width |
| 241 | **Mandibular symphysis, length:**  *Young (2006, ch. 20 modified); Wilkinson et al. (2008, ch. 43 modified); Young & Andrade (2009, ch. 43 modified); Young et al. (2011, ch. 43 modified); Young et al. (2013a, ch. 112 modified); Young et al. (2012, ch. 132); Young (2014, ch. 136); Young et al. (2016, ds 2, ch. 159); Ristevski et al. (2018, ds 2, ch. 214); Smith et al. (in review, ds 1, ch. 218); Ősi et al. (in review, ds 1, ch. 240).*  0. symphysis less than a third of mandible length (lower than 0.3)  1. symphysis less than half and more than a third of mandible length (between 0.3 and 0.45)  2. symphysis under half of mandible length (between 0.45 and 0.5)  3. symphysis greater than half of mandible length (more than 0.5) |
| 242 | **Mandibular symphysis, depth:**  *Young (2006, ch. 21); Wilkinson et al. (2008, ch. 44); Young & Andrade (2009, ch. 44); Young et al. (2011, ch. 44); Young et al. (2013a, ch. 113); Young et al. (2012, ch. 133); Young (2014, ch. 137); Young et al. (2016, ds 2, ch. 160); Ristevski et al. (2018, ds 2, ch. 215); Smith et al. (in review, ds 1, ch. 219); Ősi et al. (in review, ds 1, ch. 241).*  0. deep (9% or more of mandible length)  1. moderate (6.5–8% of mandible length)  2. narrow (4.5–6% of mandible length)  3. very narrow (4% or less of mandible length) |
| 243 | **External mandibular fenestra, presence:**  *Clark (1994, ch. 75 modified); Ortega et al. (2000, ch. 80 revised);* *Young (2006, ch. 22 part); Wilkinson et al. (2008, ch. 45 part); Young & Andrade (2009, ch. 45 part); Andrade et al. (2011, ch. 312); Young et al. (2011, ch. 45 part); Young et al. (2013a, ch. 114 part); Young et al. (2012, ch. 134 part); Young (2014, ch. 138 part); Young et al. (2016, ds 2, ch. 161 part); Ristevski et al. (2018, ds 1, ch. 312); Smith et al. (in review, ds 2, ch. 312); Ősi et al. (in review, ds 1, ch. 242).*  *State (0) occurs in Gobiosuchidae, Hylaeochampsidae, Bernissartiidae, Paralligatoridae and Metriorhynchidae. Also in derived goniopholidids (e.g.* Anteophthalmosuchus *and* Goniopholis *sensu stricto – Andrade et al., 2011), derived pholidosaurids (*Oceanosuchus *and* Terminonaris browni*), and within Dyrosauridae (*Sabinosuchus*).*  0. absent  1. present as a diminutive passage  2. present as an evident fenestra |
| 244 | **External mandibular fenestra, shape: (*)**  *Andrade et al. (2011, ch. 315); Ristevski et al. (2018, ds 1, ch. 315); Smith et al. (in review, ds 2, ch. 315); Ősi et al. (in review, ds 1, ch. 243).*  *This character is not applicable for taxa that lack external mandibular fenestrae.*  0. subcircular to poorly elliptic  1. highly elliptic, anteroposterior axis much longer than dorso-ventral axis, three time or more, but both ends rounded  2. slit-like, proportionally very long and both ends acute  3. broad teardrop-like  4. narrow teardrop-like  5. triangle |
| 245 | **External mandibular fenestra, morphology of anterior margin: (*)**  *Andrade et al. (2011, ch. 316); Ristevski et al. (2018, ds 1, ch. 316); Smith et al. (in review, ds 2, ch. 316); Ősi et al. (in review, ds 1, ch. 244).*  *State (1) is present in peirosaurids,* Araripesuchus *and closely related taxa.*  *Note that* Baurusuchus *was reconstructed as (1), but is actually (0).*  *This character is not applicable for taxa that lack external mandibular fenestrae.*  0. curved, with a broad arched margin anteriorly  1. anterodorsal and anteroventral margins poorly arched, meeting at an acute angle anteriorly, anterior end is wedge-like |
| 246 | **Surangular foramen, presence:**  *Clark et al. (2004 modified); Nesbitt (2007 modified); Nesbitt (2011, ch. 163); Ősi et al. (in review, ds 1, ch. 245).*  *State (1) is a putative apomorphy of* Junggarsuchus.  *The foramen is located posterior to the external mandibular fenestra, and is surrounded by the surangular.*  0. present and small  1. present and large  2. absent |
| 247 | **Dentary, ventral margin strongly curved:**  *Young et al. (2016, ds 2, ch. 162 + 163); Ristevski et al. (2018, ds 2, ch. 217); Smith et al. (in review, ds 1, ch. 221); Ősi et al. (in review, ds 1, ch. 246).*  *State (1) occurs in* Junggarsuchus, Dakosaurus, Baurusuchus, *and in 'trematochampsids' and peirosaurids.*  *State (2) occurs in* Pachycheilosuchus *+* Pietraroiasuchus*.*  0. no  1. yes, ventral margin is distinctly curved (convex). It rises sharply dorsally towards the anterior tip (this curvature occurs along the anterior ventral margin of the dentary)  2. yes, ventral margin is curved (concave). It rises dorsally towards the anterior tip (this curvature occurs along the anterior ventral margin of the dentary, from a dorsoventrally deepened region of the dentary, immediately anterior to the dentary-splenial suture) |
| 248 | **Dentary foramina, lateral and dorsal surface of the anterior (symphyseal) region of the dentary:**  *Young et al. (2016, ds 2, ch. 164); Ristevski et al. (2018, ds 2, ch. 218); Smith et al. (in review, ds 1, ch. 222); Ősi et al. (in review, ds 1, ch. 247).*  *State (1) is a putative apomorphy of* Dakosaurus*.*  0. foramina either small or variable in size. Number is variable.  1. has numerous small to medium-sized foramina |
| 249 | **Surangulodentary groove, morphology:**  *Young (2006, ch. 23 modified); Wilkinson et al. (2008, ch. 46 part); Young & Andrade (2009, ch. 46 part); Young et al. (2011, ch. 46 part); Young et al. (2013a, ch. 115 part); Young et al. (2012, ch. 135); Young (2014, ch.139); Young et al. (2016, ds 2, ch. 166); Ristevski et al. (2018, ds 2, ch. 220); Smith et al. (in review, ds 1, ch. 224); Ősi et al. (in review, ds 1, ch. 248).*  *Note taphonomic or preservational damage can obscure state (1).*  *State (2) is a putative apomorphy of the clade Geosaurini. Previously it was considered an apomorphy of* Dakosaurus*; however, the type specimens for the genera* Dakosaurus, Plesiosuchus *and* Geosaurus *share this morphology. The deep groove is also observed in the holotype of* Torvoneustes coryphaeus*, and large specimens of* Tyrannoneustes lythrodectikos*.*  0. absent  1. present as a subtle, shallow groove  2. deeply excavated |
| 250 | **Surangulodentary groove, relative length on both elements: (*)**  *Young et al. (2013a, ch. 115 part); Young et al. (2012, ch. 136); Young (2014, ch. 140); Young et al. (2016, ds 2, ch. 167); Ristevski et al. (2018, ds 2, ch. 221); Smith et al. (in review, ds 1, ch. 225); Ősi et al. (in review, ds 1, ch. 249).*  *This character is not applicable for taxa that lack the surangulodentary groove.*  0. groove is longer on the dentary than on the surangular  1. groove is as long on the dentary as on the surangular |
| 251 | **Surangulodentary groove, large foramen present at the dentary terminus: (*)**  *Wilkinson et al. (2008, ch. 46 part); Young & Andrade (2009, ch. 46 part); Young et al. (2011, ch. 190); Young et al. (2013a, ch. 116); Young et al. (2012, ch. 137); Young (2014, ch. 141); Young et al. (2016, ds 2, ch. 168); Ristevski et al. (2018, ds 2, ch. 222); Smith et al. (in review, ds 1, ch. 226); Ősi et al. (in review, ds 1, ch. 250).*  *This character is not applicable for taxa that lack the surangulodentary groove.*  *State (1) is a putative apomorphy of* Dakosaurus.  0. absent  1. present |
| 252 | **Mandibular grooves, morphology along the dentary in lateral view: (*)**  *Smith et al. (in review, ds 1, ch. 227); Ősi et al. (in review, ds 1, ch. 251).*  *This character is not applicable for taxa that lack the surangulodentary groove.*  *State (1) occurs in basal metriorhynchoids.*  0. the surangulodentary and angulodentary grooves are either poorly developed, not elongate, converge towards one another (i.e. they are not parallel, and close to one another ventral to the dentary rami tooth row  1. the surangulodentary and angulodentary grooves are parallel and positioned close to one another ventral to the dentary rami tooth row |
| 253 | **Splenial, involvement in mandibular symphysis:**  *Young (2006, ch. 25 modified); Wilkinson et al. (2008, ch. 49 modified); Young & Andrade (2009, ch. 49 modified); Young et al. (2011, ch. 49 modified); Young et al. (2013a, ch. 117 modified); Young et al. (2012, ch. 138 modified); Young (2014, ch. 142); Young et al. (2016, ds 2, ch. 169 modified); Ristevski et al. (2018, ds 2, ch. 223); Smith et al. (in review, ds 1, ch. 228); Ősi et al. (in review, ds 1, ch. 252).*  0. slight (less than 10% of symphysis length)  1. extensive (greater than, or equal to, 15% of symphysis length)  2. not involved |
| 254 | **Angular, in lateral view, extension of the anterior lateral ramus:**  *Young (2006, ch. 24 part); Wilkinson et al. (2008, ch. 47 part); Young & Andrade (2009, ch. 47 part); Young et al. (2011, ch. 47 part); Young et al. (2013a, ch. 118 part); Young et al. (2012, ch. 139); Young (2014, ch. 143); Young et al. (2016, ds 2, ch. 170); Ristevski et al. (2018, ds 2, ch. 224); Smith et al. (in review, ds 1, ch. 229); Ősi et al. (in review, ds 1, ch. 253).*  0. short, does not extend beyond the orbits  1. long, does extend anteriorly beyond the orbits |
| 255 | **Angular, in lateral view, posterodorsal extension:**  *Jouve et al. (2008, ch. 39 modified); Hastings et al. (2010, ch. 79 modified); Ristevski et al. (2018, ds 2, ch. 225); Smith et al. (in review, ds 1, ch. 230); Ősi et al. (in review, ds 1, ch. 254).*  0. reaches the retroarticular process  1. does not reach the retroarticular process |
| 256 | **Surangular, in lateral view, extension of the anterior lateral ramus:**  *Young (2006, ch. 24 part); Wilkinson et al. (2008, ch. 47 part); Young & Andrade (2009, ch. 47 part); Andrade et al. (2011, ch. 346 modified); Young et al. (2011, ch. 47 part); Young et al. (2013a, ch. 118 part); Young et al. (2012, ch. 140); Young (2014, ch. 144); Young et al. (2016, ds 2, ch. 171); Ristevski et al. (2018, ds 2, ch. 226); Smith et al. (in review, ds 1, ch. 231); Ősi et al. (in review, ds 1, ch. 255).*  0. short, does not extend anteriorly beyond the orbit  1. long, extends anteriorly beyond the orbit |
| 257 | **Surangular, along the dorsal margin of the mandible:**  *Wilkinson et al. (2008, ch. 48); Young & Andrade (2009, ch. 48); Young et al. (2011, ch. 48); Young et al. (2013a, ch. 119); Young et al. (2012, ch. 141); Young (2014, ch. 145); Young et al. (2016, ds 2, ch. 172); Ristevski et al. (2018, ds 2, ch. 227); Smith et al. (in review, ds 1, ch. 232); Ősi et al. (in review, ds 1, ch. 256).*  *This character does not always covary with the previous character, as in non-Rhacheosaurini metriorhynchines the dentary extensively overlaps the surangular (particularly in lateral view), obscuring its anterior development. The full extent of the surangular anterior development can only be determined by examining the dorsal margin in those taxa (e.g.,* Metriorhynchus superciliosus*).*  0. does not extend anteriorly beyond the orbit  1. does extend anteriorly beyond the orbit |
| 258 | **Surangular, presence of a distinct coronoid process:**  *Young & Andrade (2009, ch. 155); Young et al. (2011, ch. 155); Young et al. (2013a, ch. 120); Young et al. (2012, ch. 142); Young (2014, ch. 146); Young et al. (2016, ds 2, ch. 173); Ristevski et al. (2018, ds 2, ch. 228); Smith et al. (in review, ds 1, ch. 233); Ősi et al. (in review, ds 1, ch. 257).*  *In Crocodyliformes, state (1) occurs in Thalattosuchia and* Iharkutosuchus*.*  *In Thalattosuchia it appears as though all taxa have a coronoid process. In teleosauroids the coronoid process is medially orientated and is not visible in lateral view, unlike in* Pelagosaurus + *Metriorhynchidae.*  0. absent  1. present |
| 259 | **Surangular, presence of extension to the retroarticular process:**  *Norell (1988, ch. 42 modified); Brochu (1999, ch. 51 revised); Young & Andrade (2009, ch. 103); Andrade et al. (2011, ch. 350); Young et al. (2011, ch. 103); Young et al. (2013a, ch. 121); Young et al. (2012, ch. 143 modified); Young (2014, ch. 147); Young et al. (2016, ds 2, ch. 174 modified); Ristevski et al. (2018, ds 2, ch. 229); Smith et al. (in review, ds 1, ch. 234); Ősi et al. (in review, ds 1, ch. 258).*  0. absent, pinched off anterior to tip of retroarticular process, or surangular excluded from process  1. present, extends to posterior end of retroarticular process |
| 260 | **Prearticulars, presence:**  *Clark (1994, ch. 72 revised); Sereno et al. (2003, ch. 39);* *Young & Andrade (2009, ch. 89); Andrade et al. (2011, ch. 354); Young et al. (2011, ch. 89); Young et al. (2013a, ch. 122); Young et al. (2012, ch. 144); Young (2014, ch. 148); Young et al. (2016, ds 2, ch. 175); Ristevski et al. (2018, ds 2, ch. 230); Smith et al. (in review, ds 1, ch. 235); Ősi et al. (in review, ds 1, ch. 259).*  *Note, here we follow Andrade et al. (2011) in scoring* Pholidosaurus schaumburgensis *and* Sarcosuchus imperator *as lacking prearticulars (as MTY also could not find these elements in first-hand observations). As such they are scored as (?).*  *It is not possible to verify the potential prearticular in* Oceanosuchus *(Hua et al., 2007, Fig. 4U) as too much of the angular is not preserved. Thus, this OTU is scored as (?).*  *State (1) occurs in Metasuchia.*  0. present  1. absent |
| 261 | **Coronoids:**  *Jouve et al. (2005b, ch. 6 modified); Jouve et al. (2008, ch. 6 modified); Young & Andrade (2009, ch. 157 part); Hastings et al. (2010, ch. 77 modified); Young et al. (2011, ch. 157 part); Young et al. (2013a, ch. 124 part); Young et al. (2012, ch. 146 part); Young (2014, ch. 150 part); Young et al. (2016, ds 2, ch. 177 part); Ristevski et al. (2018, ds 2, ch. 231); Smith et al. (in review, ds 1, ch. 236).*  *This character is an amalgam of those in Hastings et al. (2010, ch. 77) and Young et al. (2016, ch. 177); Ősi et al. (in review, ds 1, ch. 260).*  *State (1) occurs in derived Rhacheosaurini metriorhynchids.*  *Dyrosaurids have state (2). However, to evaluate the presence of the coronoids requires well preserved specimens.*  0. present, but not exposed on the external (= lateral) surface of the mandible  1. present, and exposed on the external surface of the mandible  2. absent |
| 262 | **Coronoid, anterior development along the dorsal margin:**  *Wilkinson et al. (2008, ch. 51 modified); Young & Andrade (2009, ch. 51 modified); Young et al. (2011, ch. 51 modified); Young et al. (2013a, ch. 123 modified); Young et al. (2012, ch. 145 modified); Young (2014, ch. 149); Young et al. (2016, ds 2, ch. 176 modified); Ristevski et al. (2018, ds 2, ch. 232); Smith et al. (in review, ds 1, ch. 237); Ősi et al. (in review, ds 1, ch. 261).*  0. does not project as far as the dentary tooth row, or coronoid absent  1. projects further anteriorly than the posterior-most alveoli |
| 263 | **Articular, glenoid fossa orientation:**  *Young & Andrade (2009, ch. 154); Young et al. (2011, ch. 154); Young et al. (2013a, ch. 125); Young et al. (2012, ch. 147); Young (2014, ch. 151); Young et al. (2016, ds 2, ch. 178); Ristevski et al. (2018, ds 2, ch. 233); Smith et al. (in review, ds 1, ch. 238); Ősi et al. (in review, ds 1, ch. 262).*  0. anterodorsally  1. dorsally |
| 264 | **Retroarticular process, development:**  *Clark (1994, ch. 71 part);* *Andrade et al. (2011, ch. 358); Ristevski et al. (2018, ds 2, ch. 234); Smith et al. (in review, ds 1, ch. 239); Ősi et al. (in review, ds 1, ch. 263).*  *For practical purposes, a retroarticular process is here considered as (1) when its orientation can be established.*  *State (1) occurs in Mesoeucrocodylia.*  0. absent or poorly developed  1. present and evidently projecting posterior to glenoid fossa |
| 265 | **Retroarticular process, length of the attachment surface for the adductor muscles relative to its width: (*) (ORDERED)**  *Jouve et al. (2005, ch. 1 modified);**Jouve et al. (2008, ch. 1 modified), Andrade et al. (2011, ch. 359); Hastings et al. (2010, ch. 75 modified); Ristevski et al. (2018, ds 2, ch. 235); Smith et al. (in review, ds 1, ch. 240); Ősi et al. (in review, ds 1, ch. 264).*  *State (2) is a putative apomorphy of Dyrosauridae. Note, that in dyrosaurids the retroarticular processes also have a strong posterodorsal curvature.*  *This character is not applicable for taxa that lack retroarticular processes.*  0. short, subequal  1. moderately elongated, evidently longer than wide  2. extremely elongate, more than twice its width |
| 266 | **Retroarticular process, morphology of the surface for the attachment of adductor muscles: (*)**  *Wilkinson et al. (2008, ch. 50 modified); Young & Andrade (2009, ch. 50 modified); Andrade et al. (2011, ch. 363); Young et al. (2011, ch. 50 modified); Young et al. (2013a, ch. 126 modified); Young et al. (2012, ch. 148 modified); Young (2014, ch. 152 modified); Young et al. (2016, ds 2, ch. 179 modified); Ristevski et al. (2018, ds 2, ch. 236); Smith et al. (in review, ds 1, ch. 241); Ősi et al. (in review, ds 1, ch. 265).*  *This character is not applicable for taxa that lack retroarticular processes.*  0. trianglular  1. ellipsoid, rectangular or spoon-shaped  2. shovel-shaped (or paddle-shaped) |
| 267 | **Retroarticular process, width: (*)**  *Young & Andrade (2009, ch. 152); Young et al. (2011, ch. 152); Young et al. (2013a, ch. 127); Young et al. (2012, ch. 149); Young (2014, ch. 153); Young et al. (2016, ds 2, ch. 180); Ristevski et al. (2018, ds 2, ch. 237); Smith et al. (in review, ds 1, ch. 242); Ősi et al. (in review, ds 1, ch. 266).*  *This character is not applicable for taxa that lack retroarticular processes.*  0. narrower than the glenoid fossa  1. wider than the glenoid fossa (projecting medially past the glenoid fossa) |
| 268 | **Retroarticular process, length: (*)**  *Young & Andrade (2009, ch. 153); Young et al. (2011, ch. 153); Young et al. (2013a, ch. 128); Young et al. (2012, ch. 150); Young (2014, ch. 154); Young et al. (2016, ds 2, ch. 181); Ristevski et al. (2018, ds 2, ch. 238); Smith et al. (in review, ds 1, ch. 243); Ősi et al. (in review, ds 1, ch. 267).*  *This character is not applicable for taxa that lack retroarticular processes.*  0. long (longer than wide, and longer than the glenoid fossa width)  1. short (wider than long, and shorter than the glenoid fossa width) |
| 269 | **Retroarticular process, position of the posteromedial wing: (*)**  *Jouve et al. (2005b, ch. 2); Jouve et al. (2008, ch. 2); Hastings et al. (2010, ch. 76); Andrade et al. (2011, ch. 365); Ristevski et al. (2018, ds 2, ch. 239); Smith et al. (in review, ds 1, ch. 244); Ősi et al. (in review, ds 1, ch. 268).*  *State (1) is a putative apomorphy of Dyrosauridae.*  *This character is not applicable for taxa that lack retroarticular processes.*  0. posteromedial wing dorsally situated, or at mid height on the retroarticular process  1. posteromedial wing ventrally situated on the retroarticular process |

**Dentition and alveolar morphologies** (Ch. 270 – 334; 14.317% of characters)

*[Note abbreviations used in this section: P = premaxilla, M = maxilla, D = dentary. Thus, D1 would refer to the first dentary alveolus, while M4 would be the fourth maxillary alveolus, etc. Tooth count numbering starts from the anterior-most alveolus.]*

| # | Description |
| --- | --- |
| 270 | **Tooth row, premaxillary alveoli and posterior maxillary alveoli:**  *Young & Andrade (2009, ch. 129); Young et al. (2011, ch. 129); Young et al. (2013a, ch. 3); Young et al. (2012, ch. 5); Young (2014, ch. 5); Young et al. (2016, ds 2, ch. 6), Ristevski et al. (2018, ds 2, ch. 13); Smith et al. (in review, ds 1, ch. 14); Ősi et al. (in review, ds 1, ch. 269).*  *State (1) is a putative apomorphy of Metriorhynchidae.*  *Note that the ventral offset scored by this character is formed by the dorsoventral expansion of the orbits, this results in the ventroposterior curvature of the posterior maxillae (and thus the concave maxillary tooth row).*  0. upper tooth row largely in the same plane (excludes maxillary deflections)  1. posterior maxillary alveoli ventral to all other alveoli (caused by the ventroposterior curvature of the posterior maxillae) |
| 271 | **Premaxilla, alveolar count:**  *Young (2006, ch. 26 modified); Wilkinson et al. (2008, ch. 52 modified); Young & Andrade (2009, ch. 52 modified); Nesbitt (2011, ch. 6 modified); Young et al. (2011, ch. 52 modified); Young et al. (2013a, ch. 129 modified); Young et al. (2012, ch. 151 modified); Young (2014, ch. 155 modified); Young et al. (2016, ds 2, ch. 182 modified); Ristevski et al. (2018, ds 2, ch. 240 modified); Smith et al. (in review, ds 1, ch. 245 modified); Ősi et al. (in review, ds 1, ch. 270).*  *State (0) occurs in* Anatosuchus.  0. six or more alveoli  1. five alveoli  2. four alveoli  3. three or fewer alveoli |
| 272 | **Maxilla, alveolar count:**  *Young (2006, ch. 27 modified); Wilkinson et al. (2008, ch. 53 modified); Young & Andrade (2009, ch. 53 modified); Young et al. (2011, ch. 53 modified); Young et al. (2013a, ch. 130 modified); Young et al. (2012, ch. 152 modified); Young (2014, ch. 156); Young et al. (2016, ds 2, ch. 183); Ristevski et al. (2018, ds 2, ch. 241); Smith et al. (in review, ds 1, ch. 246); Ősi et al. (in review, ds 1, ch. 271).*  0. 11 or fewer alveoli  1. 12–16 alveoli  2. 17–20 alveoli  3. 21–28 alveoli  4. 29 or more alveoli |
| 273 | **Maxilla, end of the alveolar row:**  *Ristevski et al. (2018, ds 2, ch. 242); Smith et al. (in review, ds 1, ch. 247); Ősi et al. (in review, ds 1, ch. 272).*  *State (0) occurs in Dyrosauridae.*  *State (2) occurs in the metriorhynchid subclade* Tyrannoneustes lythrodectikos, Purranisaurus, Torvoneustes, 'Metriorhynchus' hastifer *+* *Mr. Passmore's specimen. It also occurs in Baurusuchidae,* Stolokrosuchus *and* Kaprosuchus *+* Mahajangasuchus*.*  0. maxillary tooth row terminates posterior to the posterior margin of the orbit, but does not extend beyond the anteroposterior mid-length of the supratemporal fenestrae  1. maxillary tooth row terminates level to, or posterior to, the anterior margin of the orbit  2. maxillary tooth row terminates prior to the anterior margin of the orbit |
| 274 | **Premaxilla, P1 and P2 alveoli are lateral to one another: (NEW)**  *State (1) occurs in the teleosauroids* Aeolodon priscus*,* Bathysuchus megarhinus*, and* Mycterosuchus nasutus. *State (1) also occurs in Pholidosauridae.*  *State (2) occurs in* Elosuchus.  0. no, P2 posterolateral  1. yes, both alveoli are in the same transverse plane  2. the P2 alveolus is anterolateral to the P1 alveolus |
| 275 | **Premaxilla, shape of the anterior margin between the P2-P3 alveoli: (NEW)**  *State (1) occurs in* Aeolodon priscus*,* Bathysuchus megarhinus*, and* Mycterosuchus nasutus.  *Note that this morphology does not occur in Pholidosauridae, which have a semi-circular shaped premaxilla in dorsal view. Thus, how the P1-P2 alveoli are in the same transverse plane, and its structural implications for premaxillary shape differ between teleosauroids and pholidosaurids.*  0. premaxilla lateral margins are clearly curved, with the P3 alveoli being either: in-line, posteromedial or posterolateral to the P2 alveoli  1. premaxilla lateral margins subrectangular, with the P3 alveoli being clearly lateral to the P2 alveoli (i.e. not part of a curving tooth-row) |
| 276 | **Third premaxillary alveoli, relative size when more than three premaxillary alveoli are present: (*)**  *Hastings et al. (2010, ch. 16 modified); Ristevski et al. (2018, ds 2, ch. 243); Smith et al. (in review, ds 1, ch. 248); Ősi et al. (in review, ds 1, ch. 273).This character is not applicable for taxa that have fewer than four premaxillary alveoli.*  0. not enlarged relative to both the second and fourth premaxillary alveoli  1. third alveoli are enlarged relative to both adjacent alveoli |
| 277 | **Premaxilla, tooth row: (ORDERED)**  *Sereno et al. (2001, ch. 69 modified); Turner & Buckley (2008, ch. 240 modified);* *Andrade et al. (2011, ch. 390 modified); Young et al. (2016, ds 2, ch. 12 modified), Ristevski et al. (2018, ds 2, ch. 25); Smith et al. (in review, ds 1, ch. 26); Ősi et al. (in review, ds 1, ch. 274).*  *State* *(2)* *occurs in the pholidosaurids* Chalawan, Sarcosuchus, Pholidosaurus schaumburgensis *(based on the German natural mould specimens)* *and* Meridiosaurus. *The morphology in* Elosuchus *and the French* Pholidosaurus *approaches this condition, however the P5 is directed posteriorly and the premaxilla has definitive lateral margins rather than a curved anterolateral curve (however, this could be due to the enlargement of the P3 alveoli). Here, we have created a new character state (1) to accommodate this morphology.*  *State (3) is a modification seen in* Terminonaris *and* Oceanosuchus*.*  0. alveoli along the anterior and lateral margins  1. in a slight semi-circle, (similar to state 2), but the P5 alveolar are directly posteriorly, and the premaxilla still has definitive lateral margins rather than a true anterolateral curve  2. in a slight semi-circle, resulting in the premaxillary alveoli being restricted to the anterior and anterolateral margins  3. the premaxillary tooth row is restricted to an even tighter curve, resulting in the P5 alveoli being lateral to the P4 alveoli and being somewhat laterally oriented (compared to the other four alveoli). The tighter curve means the normally very transversely wide premaxilla of pholidosaurids is now much less wide (with the maximal width at the P5) |
| 278 | **Number of teeth partially supported by both the premaxilla and maxilla:**  *Young & Andrade (2009, ch. 162); Young et al. (2011, ch. 162); Young et al. (2013a, ch. 131); Young et al. (2012, ch. 153); Young (2014, ch. 157); Young et al. (2016, ds 2, ch. 184); Ristevski et al. (2018, ds 2, ch. 244); Smith et al. (in review, ds 1, ch. 249); Ősi et al. (in review, ds 1, ch. 275).*  *State (1) occurs in* Mariliasuchus *and* Notosuchus.  0. none  1. one |
| 279 | **Presence of a premaxillary lamina extending posteriorly along the palatal surface that overlaps the anterior margin of the first maxillary alveoli:**  *Ristevski et al. (2018, ds 2, ch. 245); Smith et al. (in review, ds 1, ch. 250); Ősi et al. (in review, ds 1, ch. 276).*  *State (1) occurs in* Tyrannoneustes lythrodectikos, Torvoneustes, ‘Metriorhynchus’ hastifer *and* *Mr. Passmore’s specimen.*  0. absent  1. present |
| 280 | **Anterior margin of maxillary alveolus one:**  *Ristevski et al. (2018, ds 2, ch. 246); Smith et al. (in review, ds 1, ch. 251); Ősi et al. (in review, ds 1, ch. 277).*  *State (1) occurs in* Metriorhynchus superciliosus *and* M. geoffroyii.  0. lacks an interdigitating suture with the premaxilla  1. has an interdigitating suture with the premaxilla, restricted to the anterior margin of the first maxillary alveolus |
| 281 | **Dentary, alveolar count:**  *Young (2006, ch. 28 modified); Wilkinson et al. (2008, ch. 54 modified); Young & Andrade (2009, ch. 54 modified); Young et al. (2011, ch. 54 modified); Young et al. (2013a, ch. 132 modified); Young et al. (2012, ch. 154); Young (2014, ch. 158); Young et al. (2016, ds 2, ch. 185); Ristevski et al. (2018, ds 2, ch. 247); Smith et al. (in review, ds 1, ch. 252); Ősi et al. (in review, ds 1, ch. 278).*  *This character does not covary with the maxillary alveolar count character, as some taxa (e.g.* ‘Metriorhynchus’ casamiquelai*) have more teeth in the dentary than in the maxilla.*  0. 30 or more alveoli per rami  1. 20–29 alveoli  2. 19–15 alveoli  3. 14 or fewer alveoli |
| 282 | **Maxillary anterior alveoli shape:**  *Young et al. (2016, ds 2, ch. 186); Ristevski et al. (2018, ds 2, ch. 248); Smith et al. (in review, ds 1, ch. 253); Ősi et al. (in review, ds 1, ch. 279).*  *In Thalattosuchia, state (1) is a putative apomorphy of the clade* ‘Metriorhynchus’ hastifer *and* *Mr. Passmore's specimen.*  *Note that shearing or crushing of the snout can make this character hard to discern.*  0. sub-circular  1. sub-oval, being wider transversely than anteroposteriorly |
| 283 | **Maxillary interalveolar spaces, relative size:**  *Young (2014, ch. 159); Young et al. (2016, ds 2, ch. 187); Ristevski et al. (2018, ds 2, ch. 249); Smith et al. (in review, ds 1, ch. 254); Ősi et al. (in review, ds 1, ch. 280).*  *State (1) is a putative apomorphy of* Dakosaurus + Plesiosuchus *sub-clade and* Gracilineustes leedsi*.*  *This character correlates with the dentary interalveolar space character for the metriorhynchids* Gracilineustes leedsi and *the* Dakosaurus + Plesiosuchus *sub-clade*; *however, the maxillary interalveolar spacing does not correlate with the dentary character for the teleosauroid* Machimosaurus hugii*.*  *State (1) does not occur in* Torvoneustes carpenteri, ‘Metriorhynchus’ hastifer *and Mr. Passmore’s specimen as some interalveolar spaces are large, over half the length of the adjacent alveoli and they do not alway share the same alveolar lamina. They appear to evolve an analogous, but slightly different morphology, which has not yet been scored.*  *State (1) also occurs in* Iharkutosuchus makadii*.*  0. Interalveolar spaces are variable in size, some are similar in length to the adjacent alveoli, while others are approximately half the length of the immediately adjacent alveoli (especially towards the end of the maxillary tooth row)  1. Interalveolar spaces are/almost completely uniformly narrow, being approximately one quarter the length of the adjacent alveoli (or even smaller). The adjacent alveoli share the same alveolar lamina. |
| 284 | **Dentary tooth-row, distinctly sigmoidal:**  *Young et al. (2016, ds 2, ch. 165); Ristevski et al. (2018, ds 2, ch. 219); Smith et al. (in review, ds 1, ch. 223); Ősi et al. (in review, ds 1, ch. 281).*  *State (1) occurs in Hylaeochampsidae.*  0. no  1. yes, with the anterior alveoli orientated slightly anterolaterally and the posterior alveoli orientated posteromedially, between these two orientations the mid-region alveoli become dorsally orientated |
| 285 | **Dentary alveoli one, orientation:**  *Young et al. (2016, ds 2, ch. 188); Ristevski et al. (2018, ds 2, ch. 250); Smith et al. (in review, ds 1, ch. 255); Ősi et al. (in review, ds 1, ch. 282).*  *State (1) occurs in Tethysuchia (e.g. dyrosaurids,* Sarcosuchus, Chalawan) *and* Hamadasuchus.  *State (2) occurs in the* Pachycheilosuchus *+* Pietraroiasuchus *clade,* Iharkutosuchus makadii, Dakosaurus *and* Maledictosuchus riclaensis.  *This morphology differs from the procumbency of the first dentary alveolus seen in* Cricosaurus aracuanensis*, as they are also partially laterally orientated.*  0. dorsally orientated  1. mainly dorsally orientated, but with a slight anterior orientation  2. strongly anteriorly orientated (procumbent), resulting in the first dentary tooth being directed anteriorly from the mouth, along anteroposterior axis of the skull |
| 286 | **Dentary interalveolar spaces, relative size:**  *Young (2014, ch. 160); Young et al. (2012, ch. 131 modified); Young et al. (2016, ds 2, ch. 189); Ristevski et al. (2018, ds 2, ch. 251); Smith et al. (in review, ds 1, ch. 256); Ősi et al. (in review, ds 1, ch. 283).*  *State (1) occurs in the thalattosuchians* Dakosaurus + Plesiosuchus *sub-clade,* Gracilineustes leedsi *and* Machimosaurus hugii. *It also occurs in* Iharkutosuchus makadii*.*  *This character correlates with the maxillary interalveolar space character for the metriorhynchids* Gracilineustes leedsi *and the* Dakosaurus + Plesiosuchus *sub-clade, and for the hylaeochampsid* Iharkutosuchus makadii*, but does not for the teleosauroid* Machimosaurus hugii.  0. interalveolar spaces are variable in size, some are similar in length to the adjacent alveoli, while others are approximately half the length of the immediately adjacent alveoli  1. interalveolar spaces are/almost completely uniformly narrow, being approximately one quarter the length of the immediately adjacent alveoli (or even smaller) |
| 287 | **Dentary alveoli, diastema between the first and second alveoli:**  *Young et al. (2016, ds 2, ch. 190); Ristevski et al. (2018, ds 2, ch. 252); Smith et al. (in review, ds 1, ch. 257) Ősi et al. (in review, ds 1, ch. 284).*  *State (1) is a putative apomorphy of* Dakosaurus maximus*.*  0. absent  1. present |
| 288 | **Dentary alveoli 1–2, confluence:**  *Andrade et al. (2011, ch. 402); Young et al. (2016, ds 2, ch. 191); Ristevski et al. (2018, ds 2, ch. 253); Smith et al. (in review, ds 1, ch. 258); Ősi et al. (in review, ds 1, ch. 285).*  *State (1) is a putative apomorphy of Goniopholis.*  0. well-separated, usually as much distant from each other as from other dentary teeth  1. alveoli 1–2 confluent, separated by a thin alveolar wall, and clearly apart from neighbouring alveoli |
| 289 | **D2 alveoli, size relative to D1 alveoli:**  *Hastings et al. (2010, ch. 64 modified); Young et al. (2016, ds 2, ch. 192); Ristevski et al. (2018, ds 2, ch. 254); Smith et al. (in review, ds 1, ch. 259); Ősi et al. (in review, ds 1, ch. 286).*  0. similar in size  1. reduced in size relative to both adjacent alveoli |
| 290 | **D3 alveoli, position:**  *Hastings et al. (2010, ch. 66 modified); Ristevski et al. (2018, ds 2, ch. 255); Smith et al. (in review, ds 1, ch. 260); Ősi et al. (in review, ds 1, ch. 287).*  0. interalveolar space between D2 and D3 is approximately equal to that between D3 and D4  1. closer to the D4 alveoli |
| 291 | **Interalveolar space between the D2 and D3 alveoli relative to that of the D1 and D2 alveoli:**  *Hastings et al. (2010, ch. 65 modified); Young et al. (2016, ds 2, ch. 193); Ristevski et al. (2018, ds 2, ch. 256); Smith et al. (in review, ds 1, ch. 261); Ősi et al. (in review, ds 1, ch. 288).*  0. approximately equal in proportion  1. the D2–D3 interalveolar space is longer than the interalveolar space between the D1 and D2 |
| 292 | **D4 alveolar wall:**  *Hastings et al. (2010, ch. 68 modified); Young et al. (2016, ds 2, ch. 194); Ristevski et al. (2018, ds 2, ch. 257); Smith et al. (in review, ds 1, ch. 262); Ősi et al. (in review, ds 1, ch. 289).*  0. level with the adjacent alveoli  1. raised relative to the adjacent alveoli |
| 293 | **Dentary alveoli, diastema present between the fourth and fifth alveoli:**  *Young (2014, ch. 161); Young et al. (2016, ds 2, ch. 195); Ristevski et al. (2018, ds 2, ch. 258); Smith et al. (in review, ds 1, ch. 263); Ősi et al. (in review, ds 1, ch. 290).*  *State (1) is a putative apomorphy of Thalattosuchia and* Sarcosuchus.  *Within Thalattosuchia: state (0) is a putative apomorphy of the* Dakosaurus + Plesiosuchus *sub-clade.*  *Note that while the very small dentary interalveolar spaces are putative apomorphies of* Dakosaurus, Plesiosuchus *and* Gracilineustes leedsi*, the D4–D5 diastema is still present in* Gracilineustes leedsi.  0. absent  1. present |
| 294 | **D7 alveoli, size:**  *Jouve (2004, ch. 153 modified); Jouve (2005, ch. 3 modified); Jouve et al. (2005b, ch. 8 modified); Jouve et al. (2006, ch. 164 modified); Jouve et al. (2008, ch. 8 modified); Hastings et al. (2010, ch. 73 modified); Young et al. (2016, ds 2, ch. 196 modified); Ristevski et al. (2018, ds 2, ch. 259); Smith et al. (in review, ds 1, ch. 264); Ősi et al. (in review, ds 1, ch. 291).*  *State (1) occurs in Dyrosauridae.*  0. comparable in size to the adjacent alveoli  1. reduced in size compared to the adjacent alveoli |
| 295 | **D7 alveoli, position:**  *Jouve (2004, ch. 153 modified); Jouve (2005a, ch. 3 modified); Jouve et al. (2005b, ch. 8 modified); Jouve et al. (2006, ch. 164 modified); Jouve et al. (2008, ch. 8 modified); Hastings et al. (2010, ch. 73 modified); Young et al. (2016, ds 2, ch. 197 modified); Ristevski et al. (2018, ds 2, ch. 260); Smith et al. (in review, ds 1, ch. 265); Ősi et al. (in review, ds 1, ch. 292).*  *State (1) occurs in Dyrosauridae.*  0. comparable in size to the adjacent alveoli  1. close in position to the eighth alveoli |
| 296 | **Dentary alveoli, number of alveoli adjacent to the mandibular symphysis:**  *Young (2014, ch. 162); Young et al. (2016, ds 2, ch. 198); Ristevski et al. (2018, ds 2, ch. 261); Smith et al. (in review, ds 1, ch. 266); Ősi et al. (in review, ds 1, ch. 293).*  *Within Thalattosuchia: state (3) is a putative apomorphy of* Dakosaurus.  0. 15 or more  1. 10 to 14  2. 7 to 9  3. 4 to 6  4. Fewer than 4 |
| 297 | **Premaxilla-anterior maxillary tooth crown apicobasal length to basal width ratio:**  *Young et al. (2012, ch. 155); Young (2014, ch. 163); Young et al. (2016, ds 2, ch. 199); Ristevski et al. (2018, ds 2, ch. 262); Smith et al. (in review, ds 1, ch. 267); Ősi et al. (in review, ds 1, ch. 294).*  0. 3 or greater  1. 2.5 or less |
| 298 | **Anterior maxilla, crown size:**  *Wilkinson et al. (2008, ch. 56); Young & Andrade (2009, ch. 56); Young et al. (2011, ch. 56); Young et al. (2013a, ch. 133); Young et al. (2012, ch. 156); Young (2014, ch. 164); Young et al. (2016, ds 2, ch. 200); Ristevski et al. (2018, ds 2, ch. 263); Smith et al. (in review, ds 1, ch. 268); Ősi et al. (in review, ds 1, ch. 295).*  *It is currently unknown if this character correlates with the character quantifying mandibular symphysis depth across Crocodylomorpha. However, in Geosaurinae this is not the case, as shown by Young et al. (2013), the symphysis is deeper in* ‘Metriorhynchus’ brachyrhynchus *than* Tyrannoneustes lythrodectikos*, but the latter has tooth crowns with a greater apicobasal length. Moreover, the symphyseal depth of* Dakosaurus maximus *and* Plesiosuchus manselii *noticeably differ, but both taxa have tooth crowns similar in apicobasal length (Young et al., 2012).*  *Anterior maxilla = tooth crowns of the anterior half of the maxillary tooth row.*  0. crowns not enlarged (typically less than 3cm in apicobasal length)  1. moderately enlarged (between 3 and 4 cm in apicobasal length)  2. enlarged (apicobasal length 5 cm or greater) |
| 299 | **Anterior maxilla, mediolateral compression/crown cross section:**  *Young (2006, ch. 30); Wilkinson et al. (2008, ch. 57); Young & Andrade (2009, ch. 57); Young et al. (2011, ch. 57); Young et al. (2013a, ch. 134 modified); Young et al. (2012, ch. 157); Young (2014, ch. 165); Young et al. (2016, ds 2, ch. 201); Ristevski et al. (2018, ds 2, ch. 264); Smith et al. (in review, ds 1, ch. 269); Ősi et al. (in review, ds 1, ch. 296).*  0. no mediolateral compression  1. weak mediolateral compression (crown midpoint labiolingual width 60–90% distal-medial width)  2. strong mediolateral compression (crown midpoint labiolingual width less than 60% distal-medial width) |
| 300 | **Anterior maxilla, constriction at base of crowns:**  *Young (2006, ch. 32); Wilkinson et al. (2008, ch. 59); Young & Andrade (2009, ch. 59); Young et al. (2011, ch. 59); Young et al. (2013a, ch. 135); Young et al. (2012, ch. 159); Young (2014, ch. 167); Young et al. (2016, ch. 203); Ristevski et al. (2018, ds 2, ch. 266); Smith et al. (in review, ds 1, ch. 271); Ősi et al. (in review, ds 1, ch. 297).*  0. absent  1. present |
| 301 | **Maxillary teeth, orientation of the anterior to mid-snout crowns:**  *Young & Andrade (2009, ch. 123); Young et al. (2011, ch. 123); Young et al. (2013a, ch. 136); Young et al. (2012, ch. 160); Young (2014, ch. 168); Young et al. (2016, ds 2, ch. 204); Ristevski et al. (2018, ds 2, ch. 267); Smith et al. (in review, ds 1, ch. 272); Ősi et al. (in review, ds 1, ch. 298).*  0. not procumbent  1. procumbent |
| 302 | **Posterior maxilla, presence of enamel bands:**  *Gasparini et al. (2006, ch. 242);* *Andrade et al. (2011, ch. 418); Young et al. (2011, ch. 167); Young et al. (2013a, ch. 137); Young et al. (2012, ch. 161); Young (2014, ch. 169); Young et al. (2016, ds 2, ch. 205); Ristevski et al. (2018, ds 2, ch. 268); Smith et al. (in review, ds 1, ch. 273); Ősi et al. (in review, ds 1, ch. 299).*  *‘Enamel bands’ follow the definition by Brusatte et al. (2007).*  *Posterior maxilla = tooth crowns in the posterior half of the maxillary tooth row.*  *State (1) occurs in* Dakosaurus *and* Geosaurus*.*  0. absent  1. present |
| 303 | **Anterior maxilla, tooth crown tip:**  *Young et al. (2011, ch. 183); Young et al. (2013a, ch. 138); Young et al. (2012, ch. 162); Young (2014, ch. 170); Young et al. (2016, ds 2, ch. 206); Ristevski et al. (2018, ds 2, ch. 269); Smith et al. (in review, ds 1, ch. 274); Ősi et al. (in review, ds 1, ch. 300).*  0. sharp or worn apex  1. blunt and rounded at the tips |
| 304 | **Dentary tooth opposite to premaxilla-maxilla contact, isometry:**  *based on Clark (1994, ch. 80); Wilkinson et al. (2008, ch. 60); Young & Andrade (2009, ch. 60); Andrade et al. (2011, ch. 408); Young et al. (2011, ch. 60); Young et al. (2013a, ch. 139); Young et al. (2012, ch. 163); Young (2014, ch. 171); Young et al. (2016, ds 2, ch. 207); Ristevski et al. (2018, ds 1, ch. 408); Smith et al. (in review, ds 2, ch. 408); Ősi et al. (in review, ds 1, ch. 301).*  *Alveolar size may be used as a reasonable proxy for crown size, when teeth are not preserved.*  0. subequal to other neighbouring teeth  1. tooth is at least evidently enlarged, anisometric relative to other neighbouring teeth |
| 305 | **Dentary tooth opposite to premaxilla-maxilla contact, length:**  *Clark (1994, ch. 80); Sereno et al. (2003, ch. 54); Andrade & Bertini (2008a, ch. 142);* *Andrade et al. (2011, ch. 409); Ristevski et al. (2018, ds 1, ch. 409); Smith et al. (in review, ds 2, ch. 409); Ősi et al. (in review, ds 1, ch. 302).*  *Alveolar size may be used as a reasonable proxy for crown size, when teeth are not preserved.*  0. small to medium sized, but length is no more than twice the length of other neighbouring teeth  1. hypertrophied, at least twice longer than neighboring teeth |
| 306 | **Dentary tooth opposite to premaxillary-maxillary suture, occlusion:**  *Norell (1988, ch. 29); Brochu (1999, ch. 77 modified);* *Andrade et al. (2011, ch. 410); Ristevski et al. (2018, ds 1, ch. 410); Smith et al. (in review, ds 2, ch. 410); Ősi et al. (in review, ds 1, ch. 303).*  *The series cannot be ordered, as a transition between states (0) - (2) is possible without intermediate steps.*  0. occludes either in notch at premaxilla and maxilla early in ontogeny, or lateral to premaxilla-maxilla suture, when the notch is absent or poorly defined  1. occludes in a pit between premaxilla and maxilla; no notch early in ontogeny  2. occludes medial to premaxilla-maxilla suture, but not in a pit or a notch |
| 307 | **Dentary tooth occluding against premaxillary-maxillary suture:**  *based on Norell (1988, ch. 29) and Clark (1994, ch. 80) and Brochu (1999, ch. 77); Andrade et al. (2011, ch. 411); Ristevski et al. (2018, ds 1, ch. 411); Smith et al. (in review, ds 2, ch. 411); Ősi et al. (in review, ds 1, ch. 304).*  *The tooth occluding to the premaxillomaxillary suture is usually seen as the fourth dentary tooth, but in Crocodylomorpha this may be another tooth due to the loss of anterior teeth or other morphological adaptation. The tooth is not necessarily enlarged, and may be isometric to neighbouring teeth.*  *State (0) is putative apomorphy of* Mahajangasuchus, *Sphagesauridae, and Teleosauroidea.*  *State (2) is putative apomorphy of* Sarcosuchus*.*  *Note that in teleosauroids, the D3 tooth contacts the premaxilla-maxilla suture, not the D4 tooth, due to the orientation of the D3-D4 couplet.*  0. third, or anterior  1. fourth  2. fifth, or posterior |
| 308 | **Dentition, relation between tooth rows on both sides of the skull:**  *Novas et al. (2009);* *Andrade et al. (2011, ch. 367); Ristevski et al. (2018, ds 1, ch. 367); Smith et al. (in review, ds 2, ch. 367); Ősi et al. (in review, ds 1, ch. 305).*  *State (1) is putative autapomorphy of* Yacarerani*, where maxillary tooth rows converge at mid-palate, the same occurring with the dentition in the mandible. As a consequence, anterior teeth (pairs 1-4) both in the upper and lower dentition constitute functionally distinct sets, one anterior and one posterior. Teeth at the posterior set (mid-dentition) are located close to the median line of the skull, with first tooth at least almost in contact with its complementary tooth.*  0. forming one continuous set of teeth, both in the cranium and mandible  1. forming two distinct sets, tooth rows at posterior set convergent rostrally and almost in touch each other, at mid-palate and mandible |
| 309 | **Posterior maxillary teeth, transverse section:**  *Buckley et al. (2000, ch. 116 modified); Ortega et al. (2000, ch. 104 modified); as in Andrade & Bertini (2008, ch. 135); Andrade et al. (2011, ch. 368); Ristevski et al. (2018, ds 1, ch. 368); Smith et al. (in review, ds 2, ch. 368); Ősi et al. (in review, ds 1, ch. 306).*  0. evident lateral compression affecting both edges of the crown, making both edges evident regardless of the presence/absence of carinae/keel  1. transverse section circular to subcircular, without significant lateral compression  2. transverse section ‘teardrop-like’ (= triangular), with asymmetric lateral compression occurring on the distal margin only |
| 310 | **Mid to posterior mandibular teeth, transverse section:**  *Buckley et al. (2000, ch. 116 modified); Ortega et al. (2000, ch. 104 modified); as in Andrade & Bertini (2008, ch. 146);* *Andrade et al. (2011, ch. 369); Ristevski et al. (2018, ds 1, ch. 369); Smith et al. (in review, ds 2, ch. 369); Ősi et al. (in review, ds 1, ch. 307).*  0. evident lateral compression affecting the entire crown, making evident both mesial and distal edges, regardless of the presence/absence of carinae/keel  1. transverse section circular to subcircular, without significant lateral compression  2. transverse section ‘teardrop-like’ (= triangular), with asymmetric lateral compression occurring on the mesial margin only |
| 311 | **Dentition, presence of apicobasal facets on the labial sufrace:**  *Young & Andrade (2009, ch. 130); Andrade et al. (2011, ch. 370); Young et al. (2011, ch. 130); Young et al. (2013a, ch. 140); Young et al. (2012, ch. 164); Young (2014, ch. 172); Young et al. (2016, ds 2, ch. 208); Ristevski et al. (2018, ds 2, ch. 271); Smith et al. (in review, ds 1, ch. 276); Ősi et al. (in review, ds 1, ch. 308).*  *State (1) is a putative apomorphy of* Geosaurus giganteus, G. grandis + Ieldraan melkshamensis.  0. absent, either lacking facets, or facetted into 4–5 indistinct planes  1. present, most crowns have the labial surface distinctly facetted into three planes (one large medial one, and two smaller planes either side) |
| 312 | **Dentition, presence of laminar teeth:**  *Andrade et al. (2011, ch. 371); Young et al. (2011, ch. 170); Young et al. (2013a, ch. 141); Young et al. (2012, ch. 165); Young (2014, ch. 173); Young et al. (2016, ds 2, ch. 209); Ristevski et al. (2018, ds 2, ch. 272); Smith et al. (in review, ds 1, ch. 277); Ősi et al. (in review, ds 1, ch. 309).*  *State (1) is a putative apomorphy of Geosaurina (*Geosaurus + Ieldraan*)*.  *For practical purposes, 'laminar tooth' are here considered as teeth with cross-section highly elliptical at the base of crown, with mesial-distal axis approximately twice the labial-lingual axis, or greater.*  0. absent  1. present, laminar teeth dominate dentition |
| 313 | **Dentition, presence of spatulated teeth:**  *Buckley et al. (2000, ch. 116 modified);* *Andrade et al. (2011, ch. 372); Ristevski et al. (2018, ds 1, ch. 372); Smith et al. (in review, ds 2, ch. 372); Ősi et al. (in review, ds 1, ch. 310).*  *The spatulated morphology refers to the morphology of the crown, not simply its compression, number of cusps or presence of cingula. Therefore, it is considered as a different character, and treated separately. However, all spatulated teeth are considered as laterally compressed.*  *State (1) occurs in* Candidodon, Malawisuchus *and* Uruguaysuchus*.*  0. absent  1. present |
| 314 | **Dentition, presence of tribodont teeth in both the posterior maxillae and dentaries:**  *Ristevski et al. (2018, ds 2, ch. 274); Smith et al. (in review, ds 1, ch. 279); Ősi et al. (in review, ds 1, ch. 311).*  *State (1) occurs in Bernissartiidae and in some alligatoroids*.  *For practical purposes, ‘tribodont teeth’ are here considered as teeth that are ‘low crowned’, bulbous, mesiodistally compressed, single cusped, and lack carinae.*  0. absent  1. present |
| 315 | **Mid to posterior dentition, presence of pebbled ornamentation on tooth crown surface:**  *Andrade et al. (2011, ch. 374); Ristevski et al. (2018, ds 1, ch. 374); Smith et al. (in review, ds 2, ch. 374); Ősi et al. (in review, ds 1, ch. 312).*  *State (1) is putative apomorphy of Sphagesauridae.*  0. absent  1. present, enamel ornamented with a peebled pattern |
| 316 | **Mid to posterior dentition, presence of accessory ridges on labial-lingual surfaces of crown:**  *Andrade et al. (2011, ch. 376); Ristevski et al. (2018, ds 1, ch. 376); Smith et al. (in review, ds 2, ch. 376); Ősi et al. (in review, ds 1, ch. 313).*  *The ridges present in* Notosuchus *and sphagesaurids do involve enamel and dentine, therefore should not be considered as superficial ornamentation.*  *State (1) occurs in* Notosuchus *and in derived sphagesaurids (i.e. not* Adamantinasuchus *and* Yacareni*).*  0. absent  1. present, apicobasal, evident and well-spaced, formed by enamel and dentine |
| 317 | **Mid to posterior dentition, number of cusps per tooth:**  *Gomani (1997, ch. 46 modified); Buckley et al. (2000, ch. 113 modified); Pol (2003, ch. 162 modified); Turner & Buckley (2008, ch. 188 modified);* *Andrade et al. (2011, ch. 377); Ristevski et al. (2018, ds 1, ch. 377); Smith et al. (in review, ds 2, ch. 377); Ősi et al. (in review, ds 1, ch. 314).*  *This character was modified by Andrade et al. (2011), and here only the main crown is evaluated, not the presence of accessory cusps in cingula. This is considered as a separate character. However, note that states (2) and (3) sample teeth where primary and secondary rows of cusps are present, while in states (0) and (1) there is only one row.*  *State (1) occurs in* Malawisuchus*.*  *State (2) occurs in* Iharkutosuchus*.*  *State (3) occurs in* Edentosuchus *and* Kayentasuchus, *not sampled in this analysis.*  0. each crown has single apical cusp, regardless of presence of accessory cusps in cingula  1. each crown has one main cusp aligned with smaller cusps, arranged in a single row  2. several cusps, unequal in size, arranged in more than one row  3. multiple small cusps, subequal in size, along edges of occlusal surface |
| 318 | **Tooth wear, macroscopic wear along the carinae/mesiodistal margins:**  *Young et al. (2016, ds 2, ch. 211); Ristevski et al. (2018, ds 2, ch. 275); Smith et al. (in review, ds 1, ch. 280); Ősi et al. (in review, ds 1, ch. 315).*  *State (1) is a putative apomorphy of* Dakosaurus *+ Mr Leeds Dakosaur.*  0. absent  1. present |
| 319 | **Anterior–middle dentition, tooth crown curvature:**  *Young (2006, ch. 31); Wilkinson et al. (2008, ch. 58); Young & Andrade (2009, ch. 58); Young et al. (2011, ch. 58); Young et al. (2013a, ch. 142); Young et al. (2012, ch. 166); Young (2014, ch. 174); Young et al. (2016, ds 2, ch. 212); Ristevski et al. (2018, ds 2, ch. 276); Smith et al. (in review, ds 1, ch. 281); Ősi et al. (in review, ds 1, ch. 316).*  0. none, crown apical/subapical (between 91 – 89 degrees)  1. weakly recurved (between 88 – 82 degrees)  2. strongly recurved (less than 80 degrees) |
| 320 | **Carinae, presence of keel at the edge of tooth crown:**  *Young (2006, ch. 29 part modified); Wilkinson et al. (2008, ch. 55 part modified); Young & Andrade (2009, ch. 55 part modified); Andrade et al. (2011, ch. 378); Young et al. (2011, ch. 55 part modified); Young et al. (2013a, ch. 143 part modified); Young et al. (2012, ch. 167 modified); Young (2014, ch. 175); Young et al. (2016, ds 2, ch. 213 modified); Ristevski et al. (2018, ds 2, ch. 277); Smith et al. (in review, ds 1, ch. 282); Ősi et al. (in review, ds 1, ch. 317).*  *Currently, no data suggests differential presence of keels in antero-posterior or upper-lower dentition, therefore a single character is used. Mesial-distal keels may occur independently from denticles in the mesial and distal carinae; denticulated carinae may or may not have keel on denticles.*  0. absent (i.e. lacks keeled carinae)  1. present (i.e. carinated sensu stricto, created by a smooth keel [raised ridge] on the crown edges, typically on the mesial and distal margins) |
| 321 | **Carinae, presence of ‘carinal flanges’: (*)**  *Ristevski et al. (2018, ds 2, ch. 278); Smith et al. (in review, ds 1, ch. 283); Ősi et al. (in review, ds 1, ch. 318).*  *State (1) occurs in* Plesiosuchus*,* Suchodus *and Mr Leeds Dakosaur*.  *State (2) occurs in* Dakosaurus.  *This character is not applicable for taxa that lack carinae on all tooth crowns.*  0. absent - the external surfaces of the tooth crowns are still convex/straight when they approach the carinae  1. poorly-developed - the external surface of the tooth crown becomes concave immediately adjacent to the carinae. However, they are unequally expressed on the labial and lingual surfaces, and are rarely expressed along the entire carina  2. well-developed -the external surface of the tooth crown becomes concave immediately adjacent to the carinae. They are present on both the labial and lingual surfaces, being most noticeably developed at the mid-crown and apex |
| 322 | **Carinae, height of the keel in the apical region:**  *Ristevski et al. (2018, ds 2, ch. 279); Smith et al. (in review, ds 1, ch. 284); Ősi et al. (in review, ds 1, ch. 319).*  *State (1) occurs in* Torvoneustes.  0. keel is either absent, or not greatly enlarged  1. keel is greatly enlarged in height |
| 323 | **Carinae, presence of false zipdont serrations at crown edges: (*)**  *Young et al. (2011, ch. 172 part); Young et al. (2013a, ch. 144 part); Young et al. (2012, ch. 168 part); Young (2014, ch. 176 part); Young et al. (2016, ds 2, ch. 214 part); Ristevski et al. (2018, ds 2, ch. 280); Smith et al. (in review, ds 1, ch. 285); Ősi et al. (in review, ds 1, ch. 320).*  *This character is not applicable for taxa that lack carinae on all tooth crowns.*  *False ziphodonty (= conspicuous superficial enamel ornamentation contacting the keel) herein follows the definition described in Prasad & de Lapparent de Broin (2002).*  *State (1) occurs in* Theriosuchus pusillus*.*  *State (2) occurs in* Goniopholis, Anteophthalmosuchus, Torvoneustes*, and Machimosaurini.*  0. absent across the dentition  1. present, but restricted to the tooth crowns in the posterior end of the tooth row  2. present across the dentition |
| 324 | **Carinae, presence and development of true denticles at crown edges: (*)**  *Young (2006, ch. 29 part); Wilkinson et al. (2008, ch. 55 part); Young & Andrade (2009, ch. 53 part); Young et al. (2011, ch. 53 part); Young et al. (2012, ch. 169); Young (2014, ch. 177); Young et al. (2016, ds 2, ch. 215); Ristevski et al. (2018, ds 2, ch. 281); Smith et al. (in review, ds 1, ch. 286); Ősi et al. (in review, ds 1, ch. 321).*  *In Thalattosuchia, basal geosaurines are scored as state (1).*  *Derived genera within Geosaurini are scored as state (2).*  *This character is not applicable for taxa that lack carinae on all tooth crowns.*  *True ziphodonty herein follows the definition described in Prasad & de Lapparent de Broin (2002).*  0. absent  1. incipient denticles that are poorly defined (hard to discern, in some cases even under Scanning Electron Microscopy). Typically, they either alter the height of the carinal keel very little or not at all (definition described in Young *et al*., 2013)  2. well-defined denticles (can be discerned with or without optical aids) |
| 325 | **Carinae (mid-posterior dentition), presence and morphology of denticles at crown edges:**  *Buckley et al. (2000, ch. 104 modified); Sereno et al. (2003, ch. 53 modified); Andrade & Bertini (2008a, ch. 132 revised);* *Andrade et al. (2011, ch. 379 modified – character states re-ordered); Young et al. (2011, ch. 172 modified); Young et al. (2013a, ch. 145 modified); Young et al. (2012, ch. 170 modified); Young (2014, ch. 178 modified); Young et al. (2016, ds 2. ch. 216 modified – new character state added); Ristevski et al. (2018, ds 2, ch. 282); Smith et al. (in review, ds 1, ch. 287); Ősi et al. (in review, ds 1, ch. 322).*  *State (1) is putative apomorphy of Notosuchidae + Sphagesauridae.*  *In Thalattosuchia, basal geosaurines score as state (2).*  *Derived genera within Geosaurini score as state (3).*  *Note that this character and the character describing the presence of true denticles appear to correlate. However, the two morphologies are not the same, and it is possible that taxa can score differently for these two characters (i.e., the ziphomorphy condition – see Andrade & Bertini, 2008a).*  *Moreover, in Metriorhynchidae the development of the denticles, and whether they form a contiguous row along the carina is highly variable. Some taxa have contiguous and well-defined denticles (e.g.* Dakosaurus*,* Plesiosuchus*,* Geosaurus*) while some taxa have contiguous but incipient denticles (*Torvoneustes*), others non-contiguous incipient denticles (*Tyrannoneustes*,* ‘M.’ brachyrhynchus*).*  0. carinae and/or denticles are absent (non-ziphodont), or homogenous carina where serrations may appear as the result of superficial enamel ornamentation (false ziphodont)  1. heterogeneous carina, tubercle-like true denticles that do not form a series (ziphomorph)  2. heterogeneous carina, cuneiform or ripple-like true denticles form short rows of 2–10 denticles and do not proceed contiguously along the entire carina (incipient ziphodont)  3. homogeneous carina, cuneiform or ripple-like true true denticles form a contiguous, or near contiguous, series along the entire carina (ziphodont) |
| 326 | **Carinae, true denticle shape when observed in lingual or labial view: (*)**  *Young et al. (2012, ch. 171); Young (2014, ch. 179); Young et al. (2016, ds 2, ch. 217); Ristevski et al. (2018, ds 2, ch. 283); Smith et al. (in review, ds 1, ch. 288); Ősi et al. (in review, ds 1, ch. 323).*  *In Thalattosuchia,* Plesiosuchus *and* Suchodus are *scored as state (0).*  *This character is not applicable for taxa that lack carinae on all tooth crowns, and for those that lack denticles.*  0. “chisel”-shaped or rectangular  1. rounded |
| 327 | **Carinae, denticle distribution across the dentition:**  *Young et al. (2012, ch. 172); Young (2014, ch. 180); Young et al. (2016, ds 2, ch. 218); Ristevski et al. (2018, ds 2, ch. 284); Smith et al. (in review, ds 1, ch. 289); Ősi et al. (in review, ds 1, ch. 324).*  *In Thalattosuchia, state (2) occurs in* Dakosaurus*.*  *At present no taxon is known to combine the microziphodont and macroziphodont conditions. However, it is entirely possible that such a taxon could occur. As such, state (3) was created.*  *In Thalattosuchia,* Dakosaurus *scores as (2), while* ‘Metriorhynchus’ brachyrhynchus, Tyrannoneustes lythrodectikos*,* Torvoneustes*,* Geosaurus *and* Plesiosuchus *score as (1).*  *Note that this character appears to correlate with the characters describing the incipient/well-developed denticles) and homogeneous/heterogenous carinae. However, these morphologies are not the same, and it is possible that taxa can score differently for these three characters.*  *In Metriorhynchidae the development of the macroscopic denticles is a putative apomorphy of* Dakosaurus*, giving this genus macroscopic, well-defined contiguous denticles. In contrast,* Plesiosuchus *and* Geosaurus *have microscopic, well-defined contiguous denticles;* Torvoneustes *has microscopic, incipient contiguous denticles; while* Tyrannoneustes *and* ‘M.’ brachyrhynchus *have microscopic, incipient, non-contiguous denticles.*  *Thus, these three characters are describing a different aspect of denticle development and arrangement.*  0. all or most teeth lack denticles  1. all teeth are microziphodont (*sensu* Andrade *et al*., 2010)  2. all teeth are macroziphodont (*sensu* Andrade *et al*., 2010)  3. teeth show variation in denticle size (with both microziphodonty and macroziphodonty) |
| 328 | **Carinae (maxillae), distribution of denticles at crown edges:**  *based on Price (1950) and Pol (2003); Andrade & Bertini (2008a, ch. 132 modified);* *Andrade et al. (2011, ch. 380); Ristevski et al. (2018, ds 1, ch. 380); Smith et al. (in review, ds 2, ch. 380); Ősi et al. (in review, ds 1, ch. 325).*  *This character samples presence of true denticles only, not all serrated carinae or ziphomorph denticles.*  *State (1) is putative apomorphy of Notosuchidae + Sphagesauridae (but note that* Adamantinasuchus *and* Mariliasuchu*s do not share the character).*  0. mesial and distal crown edges with the same morphology, either with or without true denticles  1. mesial carina absent and distal carina present |
| 329 | **Carinae (mid-posterior mandible), distribution of denticles at crown edges:**  *Andrade & Bertini (2008a, ch. 132 modified);* *Andrade et al. (2011, ch. 381); Ristevski et al. (2018, ds 1, ch. 381); Smith et al. (in review, ds 2, ch. 381); Ősi et al. (in review, ds 1, ch. 326).*  *State (1) is putative apomorphy of Sphagesaurus, but unknown in* Armadillosuchus*.*  0. mesial and distal crown edges with the same morphology, either with or without true denticles  1. mesial carina present and distal carina absent, with mid-posterior teeth ocluding as opposing blades |
| 330 | **Occlusion, relation between maxillary and dentary series:**  *Young et al. (2011, ch. 173); Young et al. (2013a, ch. 146); Young et al. (2012, ch. 173); Young (2014, ch. 181); Young et al. (2016, ds 2, ch. 219); Ristevski et al. (2018, ds 2, ch. 285); Smith et al. (in review, ds 1, ch. 290); Ősi et al. (in review, ds 1, ch. 327).*  0. in-line or interlocked  1. maxillary dentition overbites dentary dentition |
| 331 | **Morphology of enamel surface ornamentation, apicobasal ridges:**  *Young et al. (2011, ch. 174); Young et al. (2013a, ch. 147); Young et al. (2012, ch. 174 modified); Young (2014, ch. 182 modified); Young et al. (2016, ds 2, ch. 220 modified); Ristevski et al. (2018, ds 2, ch. 286 modified); Smith et al. (in review, ds 1, ch. 291 modified); Ősi et al. (in review, ds 1, ch. 328).*  *In Thalattosuchia,* Geosaurus*,* Dakosaurus, Rhacheosaurus *and* Cricosaurus *score as state (0).*  *State (1) occurs in* Ieldraan melkshamensis*.*  *State (2) occurs in* Tyrannoneustes*.*  *State (3) occurs in Mr Leeds dakosaur,* Suchodus, Plesiosuchus manselii*.*  *State (4) is the standard, ridged crocodylomorph morphotype.*  *State (5) occurs in Mr Passmore’s specimen.*  0. enamel ornamentation absent macroscopically (although under SEM microscopic ripples may be present)  1. enamel ornamentation present macroscopically, but largely looks like an enlarged version of the ‘rippled’ morphology seen under the SEM in ‘smooth specimens’. There may also be the occasional poorly defined apicobasal ridge  2. enamel ornamentation largely inconspicuous, being composed of short, well-spaced, well-defined apicobasally aligned ridges on at least the basal half of the crown  3. enamel ornamentation composed of numerous apicobasally aligned ridges that are of low-relief (can only be properly viewed with visual aids), set close to each other, but become shorter and well-spaced towards the carinae  4. enamel ornamentation composed of well-defined apicobasally aligned ridges that are conspicuous and are elongate; being continuous, or having long discontinuous ridges  5. noticeable disparity between the labial and lingual surfaces: lingual surface changes from the standard apicobasal ridge morphology basally, to having shorter ridges which create almost reticulating pattern in the mid-crown region on the lingual surface; on the labial surface, basally the crown is largely smooth, and nearer the mid-crown and up towards the apex the crown is ornamented with numerous short ridges that similarly can make a reticulating pattern |
| 332 | **Morphology of apical enamel surface ornamentation, macroscopic anastomosed pattern:**  *Young et al. (2012, ch. 175); Young (2014, ch. 183); Young et al. (2016, ds 2, ch. 221); Ristevski et al. (2018, ds 2, ch. 287); Smith et al. (in review, ds 1, ch. 292); Ősi et al. (in review, ds 1, ch. 329).*  *State (1) occurs in Machimosaurini* (*e.g.* Machimosaurus, Lemmysuchus), Torvoneustes, *and Goniopholididae (e.g.* Anteophthalmosuchus *and* Goniopholis*).*  0. absent  1. present and strongly developed, but only in the apical region of the crown |
| 333 | **Maxillary teeth, occurrence of bilateral paramesial rotation: (ORDERED)**  *Pol (2003, ch. 137 modified); Andrade & Bertini (2008a, ch. 133);* *Andrade et al. (2011, ch. 414); Ristevski et al. (2018, ds 1, ch. 414); Smith et al. (in review, ds 2, ch. 414); Ősi et al. (in review, ds 1, ch. 330).*  *State (1) occurs in* Mariliasuchus *and* Notosuchus*.*  *State (2) is a putative apomorphy of Sphagesauridae.*  0. absent  1. bilateral paramesial rotation up to 30 degrees from the original plane  2. bilateral paramesial rotation clearly over 30 degrees from the original plane |
| 334 | **Middle and posterior mandibular teeth, occurrence of bilateral paramesial rotation:**  *Andrade & Bertini (2008a, ch. 144);* *Andrade et al. (2011, ch. 415); Ristevski et al. (2018, ds 1, ch. 415); Smith et al. (in review, ds 2, ch. 415); Ősi et al. (in review, ds 1, ch. 331).*  *State (2) is a putative apomorphy of Sphagesauridae.*  0. not oblique or slightly altered  1. oblique (more than 30 degrees). |
| 335 | **Middle and posterior teeth, presence of cingula with accessory cusps:**  *Andrade & Bertini (2008a, ch. 149 modified);* *Andrade et al. (2011, ch. 417); Ristevski et al. (2018, ds 1, ch. 417); Smith et al. (in review, ds 2, ch. 417); Ősi et al. (in review, ds 1, ch. 332).*  *State (1) occurs in* Candidodon *and* Malawisuchus*.*  0. absent  1. present, cingulum bearing a series small of cusps, set labial/lingual to the main body of crown |
| 336 | **Morphology of enamel surface ornamentation, ‘pseudodenticles’:**  *Ristevski et al. (2018, ds 2, ch. 288); Smith et al. (in review, ds 1, ch. 293); Ősi et al. (in review, ds 1, ch. 333).*  *State (1) occurs in* Machimosaurus hugii *and* M. rex*. The ‘pseudodenticles’ are denticle-like structures that occur on the enamel ridges, but not on the carinae.*  0. absent  1. present |

**Axial post-cranial skeleton** (Ch. 335 – 365; 6.828% of characters)

[*Vertebrae (= cervicale, thoracicae, lumbales, sacrales and caudal), costae (= cervicales, thoracicae, sacrales and arcus hæmales)*]

| # | Description |
| --- | --- |
| 337 | **Atlas, hypocentrum length:**  *Young & Andrade (2009, ch. 122); Young et al. (2011, ch. 122); Young et al. (2013a, ch. 148); Young et al. (2012, ch. 176); Young (2014, ch. 184); Young et al. (2016, ds 2, ch. 222); Ristevski et al. (2018, ds 2, ch. 289); Smith et al. (in review, ds 1, ch. 294); Ősi et al. (in review, ds 1, ch. 334).*  0. long: greater than 15% of odontoid process length  1. short: subequal to odontoid process length (± 5%) |
| 338 | **Axis, neural arch diapophysis:**  *Young & Andrade (2009, ch. 104); Young et al. (2011, ch. 104); Young et al. (2013a, ch. 149); Young et al. (2012, ch. 177); Young (2014, ch. 185); Young et al. (2016, ds 2, ch. 223); Ristevski et al. (2018, ds 2, ch. 290); Smith et al. (in review, ds 1, ch. 295); Ősi et al. (in review, ds 1, ch. 335).*  0. absent  1. present |
| 339 | **Presacral vertebrae number:**  *Young & Andrade (2009, ch. 156); Young et al. (2011, ch. 156); Young et al. (2013a, ch. 150); Young et al. (2012, ch. 178); Young (2014, ch. 186); Young et al. (2016, ds 2, ch. 224); Ristevski et al. (2018, ds 2, ch. 291); Smith et al. (in review, ds 1, ch. 296); Ősi et al. (in review, ds 1, ch. 336).*  0. 24  1. 25 |
| 340 | **Number of cervico-dorsal vertebrae where the parapophyses are borne on the centrum (‘cervical vertebrae’), including the atlas-axis:**  *Young (2006, ch. 35 modified); Wilkinson et al. (2008, ch. 63 modified); Young & Andrade (2009, ch. 63 modified); Young et al. (2011, ch. 63 modified); Young et al. (2013a, ch. 151); Young et al. (2012, ch. 179); Young (2014, ch. 187); Young et al. (2016, ds 2, ch. 225); Ristevski et al. (2018, ds 2, ch. 292); Smith et al. (in review, ds 1, ch. 297); Ősi et al. (in review, ds 1, ch. 337).*  0. 9 or 10  1. 8  2. 7 |
| 341 | **Cervical vertebrae, hypapophyses:**  *Ristevski et al. (2018, ds 2, ch. 293 modified); Smith et al. (in review, ds 1, ch. 298 modfied); Ősi et al. (in review, ds 1, ch. 338).*  *This character scores the presence of distinct hypapophyses on the ventral surface of the cervical centra.*  *State (1) is a putative apomorphy of Thalattosuchia.*  0. present  1. reduced, distinct ventral processes are absent, but a reduced anteroposterior keel is still present |
| 342 | **Cervical vertebrae, shape:**  *Clark (1994, ch. 92 modified); Young & Andrade (2009, ch. 145 modified); Young et al. (2011, ch. 145 modified); Young et al. (2013a, ch. 152 modified); Young et al. (2012, ch. 180 modified); Young (2014, ch. 188 modified); Young et al. (2016, ds 2, ch. 226); Ristevski et al. (2018, ds 2, ch. 294); Smith et al. (in review, ds 1, ch. 299); Ősi et al. (in review, ds 1, ch. 339).*  *Designed to test the homology of repeated procoely evolution in Crocodylomorpha.*  *State (2) is occurs in Eusuchia.*  0. amphicoelous or amphyplatian  1. weakly procoelous (i.e. the *Isisfordia* and *Junggarsuchus* morphotype – posterior condyle is poorly developed, with the rim of the posterior face of the centrum still distinct from the convexity of the condyle)  2. strongly procoelous (i.e. the eusuchian morphotype – well-developed posterior condyle, which is formed by the entire posterior face of the centrum) |
| 343 | **Posterior cervical vertebrae, centrum length vs centrum width:**  *Young (2006, ch. 34); Wilkinson et al. (2008, ch. 62); Young & Andrade (2009, ch. 62); Young et al. (2011, ch. 62); Young et al. (2013a, ch. 153); Young et al. (2012, ch. 181); Young (2014, ch. 189); Young et al. (2016, ds 2, ch. 227); Ristevski et al. (2018, ds 2, ch. 295); Smith et al. (in review, ds 1, ch. 300); Ősi et al. (in review, ds 1, ch. 340).*  *State (1) occurs in Geosaurinae.*  *State (2) is a putative apomorphy of Metriorhynchidae.*  0. long (centrum length more than 1.5 times the centrum width)  1. moderate (centrum length to width subequal, ± 5%)  2. short (centrum length less than 95% of the centrum width) |
| 344 | **Middle cervical vertebrae, neural spine height relative to centrum height:**  *Young et al. (2012, ch. 182); Young (2014, ch. 190); Young et al. (2016, ds 2, ch. 228); Ristevski et al. (2018, ds 2, ch. 296); Smith et al. (in review, ds 1, ch. 301); Ősi et al. (in review, ds 1, ch. 341).*  *Currently, there is not the information needed to score for most crocodylomorphs. Within Thalattosuchia* Steneosaurus edwardsi *is (0),* St. leedsi *is (1), and metriorhynchids are state (2).*  0. neural spine height is greater than centrum height  1. neural spine and centrum heights are approximately equal  2. neural spine height is less than centrum height |
| 345 | **Number of cervico-dorsal vertebrae where the parapophyses are borne partially, or solely, on the neural arch (‘thoracic vertebrae’):**  *Young et al. (2011, ch. 175); Young et al. (2013a, ch. 154); Young et al. (2012, ch. 183); Young (2014, ch. 191); Young et al. (2016, ds 2, ch. 229); Ristevski et al. (2018, ds 2. ch. 297); Smith et al. (in review, ds 1, ch. 302); Ősi et al. (in review, ds 1, ch. 342).*  *This character, (along with the character categorising lumbar vertebrae) was formulated to help understand the regionalisation of the presacral column. Currently, there is not the information needed to score for most crocodylomorphs.*  0. 12  1. 13  2. 14  3. 15 |
| 346 | **Number of cervico-dorsal vertebrae posterior to the “thoracic vertebrae” and anterior to the sacral vertebrae where the parapophyses are no longer borne on the neural arch (‘lumbar vertebrae’):**  *Young et al. (2011, ch. 176); Young et al. (2013a, ch. 155); Young et al. (2012, ch. 184); Young (2014, ch. 192); Young et al. (2016, ds 2, ch. 230); Ristevski et al. (2018, ds 2, ch. 298); Smith et al. (in review, ds 1, ch. 303); Ősi et al. (in review, ds 1, ch. 343).*  *This character, (along with the character categorising thoracic vertebrae) was formulated to help understand the regionalisation of the presacral column. Currently, there is not the needed information to score for most crocodylomorphs.*  0. 2  1. 3  2. 4 |
| 347 | **Thoracic and lumbar vertebrae, shape:**  *Clark (1994, ch. 93 modified); Young & Andrade (2009, ch. 146 modified); Young et al. (2011, ch. 146 modified); Young et al. (2013a, ch. 156 modified); Young et al. (2012, ch. 185 modified); Young (2014, ch. 193 modified); Young et al. (2016, ds 2, ch. 231); Ristevski et al. (2018, ds 2, ch. 299); Smith et al. (in review, ds 1, ch. 304); Ősi et al. (in review, ds 1, ch. 344).*  *State (2) is a putative apomorphy of Eusuchia.*  0. amphicoelous or amphyplatian  1. weakly procoelous (i.e. the *Isisfordia* and *Junggarsuchus* morphotype – posterior condyle is poorly developed, with the rim of the posterior face of the centrum still distinct from the convexity of the condyle)  2. strongly procoelous (i.e. the eusuchian morphotype – well-developed posterior condyle, which is formed by the entire posterior face of the centrum) |
| 348 | **Thoracic vertebrae, shallow fossa on the anterior margin of the diapophysis immediately lateral to the parapophysis:**  *Young & Andrade (2009, ch. 165); Young et al. (2011, ch. 165); Young et al. (2013a, ch. 157); Young et al. (2012, ch. 186 modified); Young (2014, ch. 194); Young et al. (2016, ds 2, ch. 232); Ristevski et al. (2018, ds 2, ch. 300); Smith et al. (in review, ds 1, ch. 305); Ősi et al. (in review, ds 1, ch. 345).*  *State (1) is a putative apomorphy of Metriorhynchidae, best observed in thoracic vertebrae mid-to-late in the series.*  0. present  1. absent |
| 349 | **Thoracic vertebrae, orientation of parapophysis:**  *Young & Andrade (2009, ch. 166); Young et al. (2011, ch. 166); Young et al. (2013a, ch. 158); Young et al. (2012, ch. 187); Young (2014, ch. 195); Young et al. (2016, ds 2, ch. 233); Ristevski et al. (2018, ds 2, ch. 301); Smith et al. (in review, ds 1, ch. 306); Ősi et al. (in review, ds 1, ch. 346).*  *State (1) is a putative apomorphy of Metriorhynchidae.*  0. posteriorly or horizontally  1. anteriorly |
| 350 | **Anterior thoracic vertebrae, parapophysis in relation to the diapophysis:**  *Young et al. (2012, ch. 188); Young (2014, ch. 196); Young et al. (2016, ds 2, ch. 234); Ristevski et al. (2018, ds 2, ch. 302); Smith et al. (in review, ds 1, ch. 307); Ősi et al. (in review, ds 1, ch. 347).*  *Currently, there is not the information needed to score for most crocodylomorphs.*  *Within Thalattosuchia,* Steneosaurus edwardsi *and* St. leedsi *are state (0), and metriorhynchids score as state (1).*  0. parapophysis ventral to, or level with, diapophysis (when observed in lateral view)  1. parapophysis dorsal to diapophysis (when observed in lateral view) |
| 351 | **Anterior thoracic vertebrae, neural spine height relative to centrum height:**  *Young et al. (2012, ch. 189); Young (2014, ch. 197); Young et al. (2016, ds 2, ch. 235); Ristevski et al. (2018, ds 2, ch. 303); Smith et al. (in review, ds 1, ch. 308); Ősi et al. (in review, ds 1, ch. 348).*  *Currently, there is not the needed information to score for most crocodylomorphs.*  *Within Thalattosuchia,* Machimosaurus mosae *and* Steneosaurus edwardsi *are state (0), and* St. leedsi *and metriorhynchids score as state (1).*  0. neural spine and centrum heights are approximately equal  1. neural spine height is less than centrum height |
| 352 | **Sacral vertebra, number (= sacralisation of the first caudal vertebra):**  *Buscalioni & Sanz (1988, ch. 44 modified); Pol & Apesteguia (2005, ch. 115 modified);* *Andrade et al. (2011, ch. 432); Ristevski et al. (2018, ds 2, ch. 304); Smith et al. (in review, ds 1, ch. 309); Ősi et al. (in review, ds 1, ch. 349).*  *The number of sacral vertebrae can be increased by the addition of last dorsal/lumbar or the first caudal, which constitute two divergent conditions, both leading to the total number of three sacral vertebrae (R. M. Santucci, pers. comm. 2004). Andrade et al. (2011) modified this character from the original to reflect this problem, although only the latter condition (addition of first caudal) has been reported so far (see for example, description in Pol 2005: p. 7-8). Note that the fusion of sacrals observed in* Alligatorellus *and* Montsecosuchus *(1st+2nd sacrals) is not homologous to the one reported by Pol (2005) for* Notosuchus *(2nd sacral+1st caudal).*  *This character scores for a similar character as: Nesbitt (2011, ch. 207); Young et al. (2013a, ch. 159); Young et al. (2012, ch. 190); Young (2014, ch. 198); Young et al. (2016, ds 2, ch. 236). However, those characters referred to an “insertion” of a sacral vertebra between the first and second primordial sacral vertebrae.*  *This character scores for the “third” sacral found in certain taxa (e.g.* Machimosaurus, Notosuchus, Mariliasuchus *and* Baurusuchus*).*  *Within Thalattosuchia, evidence for three sacral vertebrae is found in Machimosaurini (*Lemmysuchus *and* Machimosaurus*).*  0. two  1. three, with the third being the first caudal vertebra |
| 353 | **Sacral vertebrae, shape of centra posterior face:**  *Young (2014, ch. 199); Young et al. (2016, ds 2, ch. 237); Ristevski et al. (2018, ds 2, ch. 305); Smith et al. (in review, ds 1, ch. 310); Ősi et al. (in review, ds 1, ch. 350).*  *State (1) is a putative apomorphy of* Pelagosaurus *+ Metriorhynchidae.*  *Note that this character has a wider distribution than Young (2014) and Young et al. (2016, ds 2) thought (i.e. not restricted to Geosaurini).*  0. circular to sub-circular, with- or without an equatorial bulge  1. distinctly oval, transverse width noticeably greater than dorsoventral height |
| 354 | **Caudal vertebra, shape of caudal vertebra 1:**  *Ristevski et al. (2018, ds 2, ch. 306); Smith et al. (in review, ds 1, ch. 311); Ősi et al. (in review, ds 1, ch. 351).*  *Character based on Clark (1994, ch. 94).*  *State (1) occurs in* Theriosuchus, *bernissartids and eusuchians.*  0. amphicoelous or amphyplatian  1. biconvex  2. procoelous |
| 355 | **Caudal vertebra, shape of the caudal vertebrae posterior to the first caudal:**  *Ristevski et al. (2018, ds 2, ch. 307); Smith et al. (in review, ds 1, ch. 312); Ősi et al. (in review, ds 1, ch. 352).*  *Character based on Clark (1994, ch. 94).*  0. all are amphicoelous or amphyplatian  1. mixture of semi-procoelous, amphicoelous or amphyplatian  2. all are procoelous |
| 356 | **Caudal vertebrae, number:**  *Young (2006, ch. 36 modified); Wilkinson et al. (2008, ch. 64); Young & Andrade (2009, ch. 64); Young et al. (2011, ch. 64); Young et al. (2013a, ch. 160); Young et al. (2012, ch. 191); Young (2014, ch. 200); Young et al. (2016, ds 2, ch. 239); Ristevski et al. (2018, ds 2, ch. 308); Smith et al. (in review, ds 1, ch. 313); Ősi et al. (in review, ds 1, ch. 353).*  0. less than 46  1. 50 or more |
| 357 | **Caudal vertebrae, relative height of neural spine:**  *Andrade et al. (2011, ch. 435); Ristevski et al. (2018, ds 2, ch. 309); Smith et al. (in review, ds 1, ch. 314); Ősi et al. (in review, ds 1, ch. 354).*  *State (1) occurs in Dyrosauridae.*  0. larger spines are up to 2.5 times the height of vertebral body  1. spines are typically 2.5–4 times the height of vertebral body |
| 358 | **Caudal vertebrae, abrupt change in centrum shape at the distal end:**  *Andrade et al. (2011, ch. 436 part); Young et al. (2013a, ch. 161 part); Young et al. (2012, ch. 192 part); Young (2014, ch. 201 part); Young et al. (2016, ds 2, ch. 240 part); Ristevski et al. (2018, ds 2, ch. 310 part); Smith et al. (in review, ds 1, ch. 315 part); Ősi et al. (in review, ds 1, ch. 355).*  *State (1) is a putative apomorphy of Metriorhynchidae, but also occurs in* Magyarosuchus fitosi*. This suggests this character may have a wider distribution in Metriorhynchoidea.*  *This character is an osteological correlate relating to the increase in distal tail lateral surface area. In taxa with a tail fin, this shape change is seen in both ‘flexural’, and post-flexural caudal vertebrae.*  *All the characters relating to the tail fin morphological complex are present in known metriorhynchids; however, in plesiosaurians the presence of these characters is variable between taxa, with no taxon having all the character states (Smith, 2013). Moreover, ichthyosaurs also show a gradual evolution of the tail bend and fin (see Motani, 2005). It is likely the morphological adaptations for a tail fin evolved in a mosaic manner in basal metriorhynchoids as well.*  *This character helps score the modification of the distal caudal vertebrae into a hypocercal tail.*  0. centra retain a sub-circular to sub-oval cross-section the same as, or similar to, that seen in proximal caudal vertebrae (i.e. the caudal vertebrae are isomorphic or poorly heteromorphic)  1. abrupt change in centrum shape, with strong mediolateral compression (distal vertebrae are clearly heteromorphic) |
| 359 | **Caudal vertebrae, shift in neural spine inclination near distal end:**  *Andrade et al. (2011, ch. 436 part); Young et al. (2013a, ch. 161 part); Young et al. (2012, ch. 192 part); Young (2014, ch. 201 part); Young et al. (2016, ds 2, ch. 240 part); Ristevski et al. (2018, ds 2, ch. 310 part); Smith et al. (in review, ds 1, ch. 315 part); Ősi et al. (in review, ds 1, ch. 356).*  *State (1) is a putative apomorphy of Metriorhynchidae.*  *The neural spines of the distal caudal vertebrae are unknown in* Magyarosuchus fitosi*.*  *This character is an osteological correlate for a soft tissue structure along the dorsal margin of the distal tail, as the thickening and re-orientation of the neural spines support this structure. However, this structure need not be very large (i.e. a true upper lobe of a hypocercal tail).*  *All the characters relating to the tail fin morphological complex are present in known metriorhynchids; however, in plesiosaurians the presence of these characters is variable between taxa, with no taxon having all the character states (Smith, 2013). Moreover, ichthyosaurs also show a gradual evolution of the tail bend and fin (see Motani, 2005). It is likely the morphological adaptations for a tail fin evolved in a mosaic manner in basal metriorhynchoids as well.*  *This character helps score the modification of the distal caudal vertebrae into a hypocercal tail.*  0. no, distal caudal vertebral neural spines do not have a shift in orientation (being sub-vertical and/or posteriorly inclinded)  1. yes, there is a distinct region of the distal caudal vertebrae which have a shift in neural spine orientation, changing from: a posterior inclination, to being sub-vertical, to having an anterior inclination |
| 360 | **Caudal vertebrae, ventral deflection of the distal end: (ORDERED)**  *Young (2006, ch. 33 part); Wilkinson et al. (2008, ch. 61 part); Young & Andrade (2009, ch. 61 part); Young et al. (2011, ch. 61 part); Ősi et al. (in review, ds 1, ch. 357).*  *State (2) is a putative apomorphy of Metriorhynchidae.*  *The presence of a ventral deflection of the distal caudal vertebrae is unknown in* Magyarosuchus fitosi*.*  *This character helps define the lower lobe of a hypocercal tail. Note that in ichthyosaurs (Motani, 2005), the presence of a ventral deflection does not always mean there would have been a true upper lobe.*  *All the characters relating to the tail fin morphological complex are present in known metriorhynchids; however, in plesiosaurians the presence of these characters is variable between taxa, with no taxon having all the character states (Smith, 2013). Moreover, ichthyosaurs also show a gradual evolution of the tail bend and fin (see Motani, 2005). It is likely the morphological adaptations for a tail fin evolved in a mosaic manner in basal metriorhynchoids as well.*  *This character helps score the modification of the distal caudal vertebrae into a hypocercal tail.*  *Note, most preserved metriorhynchid tails give an exaggerated angle, either due to how the vertebrae have been arranged (in disarticulated specimens) or the vertebrae are not fully in in vivo condition (for specimens preserved in limestone). The in vivo condition is shown by retaining the curvature of the post-flexural caudal vertebrae.*  *Note that juvenile specimens cannot be used to score this character (e.g.* Rhacheosaurus gracilis*) as there may be an ontogenetic increase in the angle, such as in ichthyosaurs.*  0. absent  1. present, tail bend angle is less than 10 degrees  2. present, tail bend angle is between 10-40 degrees  3. present, tail bend angle is greater than 40 degrees |
| 361 | **Caudal vertebrae, number of vertebrae involved in the tail deflection:**  *Ősi et al. (in review, ds 1, ch. 358).*  *State (2) is a putative apomorphy of Metriorhynchidae.*  *The presence of a ventral deflection of the distal caudal vertebrae is unknown in* Magyarosuchus fitosi*.*  *This character helps define the lower lobe of a hypocercal tail. Note that in ichthyosaurs (Motani, 2005) the abruptness of the caudal series deflection varies between basal and derived clades, and in mosasaurids the tail bend is spread out across multiple vertebrae, similarly to basal ichthyosaurs (Lindgren et al. 2008, 2010). Therefore, a multi-state was created here to accommodate potential basal metriorhynchoids with a tail bend spread across a high number of caudal vertebrae.*  *This character helps score the modification of the distal caudal vertebrae into a hypocercal tail.*  *Note that juvenile specimens cannot be used to score this character (e.g.* Rhacheosaurus gracilis*) as there may be an ontogenetic increase in the angle, such as in ichthyosaurs.*  0. no ventral deflection of the distal caudal series  1. deflection is large, occurring over 15 to 30 vertebrae  2. deflection is abrupt, occurring over 5 to 10 vertebrae |
| 362 | **Axis rib:**  *Young et al. (2012, ch. 193); Young (2014, ch. 202); Young et al. (2016, ds 2, ch. 241); Ristevski et al. (2018, ds 2, ch. 311); Smith et al. (in review, ds 1, ch. 316); Ősi et al. (in review, ds 1, ch. 359).*  *State (1) is a putative apomorphy of* Pelagosaurus *+ Metriorhynchidae.*  *Callovian teleosauroids have a distinct ‘bump’ or ‘process’ where a second articular head would be (see Andrews, 1913). However, in no specimen is there a second articular head preserved.*  0. holocephalous (rib elongate, with one articular head)  1. dichocephalous (rib triradiate, with two articular heads) |
| 363 | **Axis rib, tuberculum:**  *Young & Andrade (2009, ch. 149); Young et al. (2011, ch. 149); Young et al. (2013a, ch. 162); Young et al. (2012, ch. 194); Young (2014, ch. 203); Young et al. (2016, ds 2, ch. 242); Ristevski et al. (2018, ds 2, ch. 312); Smith et al. (in review, ds 1, ch. 317); Ősi et al. (in review, ds 1, ch. 360).*  0. wide with broad dorsal tip  1. narrow with acute dorsal tip |
| 364 | **Atlantal ribs, presence of very thin medial laminae at anterior end:**  *Brochu (1999, ch. 16);* *Andrade et al. (2011, ch. 437); Ristevski et al. (2018, ds 1, ch. 437); Smith et al. (in review, ds 2, ch. 437); Ősi et al. (in review, ds 1, ch. 361).*  *State (1) is a putative apomorphy of Caimaninae.*  0. absent  1. present |
| 365 | **Sacral vertebrae, relative position of lateral end of the transverse processes (= sacral ribs): (ORDERED)**  *Young (2006, ch. 53 + 54); Wilkinson et al. (2008, ch. 81 + 82); Young & Andrade (2009, ch. 81 + 82); Andrade et al. (2011, ch. 433 + 434); Young et al. (2011, ch. 81 + 82); Young et al. (2013a, ch. 163 + 164); Young et al. (2012, ch. 195 + 196); Young (2014, ch. 204 + 205); Young et al. (2016, ds 2, ch. 243 + 244); Ristevski et al. (2018, ds 2, ch. 313); Smith et al. (in review, ds 2, ch. 318); Ősi et al. (in review, ds 1, ch. 362).*  *In Thalattosuchia the first sacral (as often the second) has its transverse processes at least poorly arched ventrally (see Andrews, 1913). In* Pelagosaurus *and metriorhynchids the transverse processes are strongly arched ventrally projecting the head for head contact with the ilium below the level of the cervical centrum (1), contrasting with teleosauroids (e.g.,* Steneosaurus*). However, in* Pelagosaurus*, the transverse processes are not as slender and does not project as ventrally.*  *States (1+2) occur in Thalattosuchia.*  *State (1) occurs in teleosauroids.*  *State (2) is a putative apomorphy of* Pelagosaurus *+ Metriorhynchidae.*  0. level with the vertebral centrum  1. transverse processes of sacral vertebra one lateroventrally directed, ventral relative to the vertebral centrum  2. transverse processes of both sacral vertebrae are lateroventrally directed, ventral relative to the vertebral centrum. In these taxa, the lateral ends of the transverse processes of both sacral vertebrae are typically significantly ventrally arched. |
| 366 | **Chevrons (= haemal arches), shape near the distal end of the caudal series:**  *Young & Andrade (2009, ch. 164 modified); Young et al. (2011, ch. 164 modified); Young et al. (2013a, ch. 165 modified); Young et al. (2012, ch. 197 modified); Young (2014, ch. 206 modified); Young et al. (2016, ds 2, ch. 245 modified); Ristevski et al. (2018, ds 2, ch. 314 modified); Smith et al. (in review, ds 1, ch. 319 modified); Ősi et al. (in review, ds 1, ch. 363).*  *State (1) is a putative apomorphy of Metriorhynchidae.*  *The distal chevrons are unknown in* Magyarosuchus fitosi*.*  *This character defines the change to the chevrons that stiffen the distal tail (seen ventral to ‘flexural’ and anterior post-flexural vertebrae).*  *All the characters relating to the tail fin morphological complex are present in known metriorhynchids; however, in plesiosaurians the presence of these characters is variable between taxa, with no taxon having all the character states (Smith, 2013). Moreover, ichthyosaurs also show a gradual evolution of the tail bend and fin (see Motani, 2005). It is likely the morphological adaptations for a tail fin evolved in a mosaic manner in basal metriorhynchoids as well.*  *This character helps score the modification of the distal tail into a tail fin.*  0. in lateral view they are either sub-triangular in shape or rod-like, in anterior view they are either ‘V’ or ‘Y’ shaped  1. in lateral view the main body of the chevron is mediolaterally compressed, deepening it dorsoventrally. In anterior view, some chevrons will have a slight ‘W’ shape, created by the midline anterior process being oriented anterodorsally |
| 367 | **Chevrons (=haemal arches), presence of a notch on the ventral margin of the distal chevrons:**  *Ősi et al. (in review, ds 1, ch. 364).*  *State (1) is a putative apomorphy of* Metriorhynchus superciliosus. *However, note few metriorhynchids are known to preserve all/most of the flexural and postflexural chevrons. All studied metriorhynchid specimens preserved in limestone from the Late Jurassic of Germany lack these notches.*  *This character can only be scored if there are multiple distal chevrons preserved, and they have the complete ventral margin.*  0. absent  1. present |

**Appendicular skeleton: pectoral girdle and forelimbs** (Ch. 366 – 388; 5.066% of characters)

*[pectoral elements (ossa coracoidea & ossa scapula); stylopodia (ossa humeri), zeugopodia (ossa radii & ossa ulnae), autopodia (ossa radialia/ulnaria, ossa metacarpalia,* *& ossa digitorum manus)]*

| # | Description |
| --- | --- |
| 368 | **Coracoid, shape:**  *Young (2006, ch. 40); Wilkinson et al. (2008, ch. 69); Young & Andrade (2009, ch. 69); Young et al. (2011, ch. 69); Young et al. (2013a, ch. 166); Young et al. (2012, ch. 198); Young (2014, ch. 207); Young et al. (2016, ds 2, ch. 246); Ristevski et al. (2018, ds 2, ch. 315); Smith et al. (in review, ds 1, ch. 320); Ősi et al. (in review, ds 1, ch. 365).*  *State (1) occurs in teleosauroids.*  *State (2) occurs in Metriorhynchoidea.*  0. neither proximal (i.e. glenoid region) nor distal (i.e. postglenoid process) ends are fan-shaped, having angular margins  1. distal end convex, forming a gentle fan-shape while the proximal end is triangular in shape with blunt ends  2. both proximal and distal ends are convex |
| 369 | **Coracoid, postglenoid process:**  *Nesbitt (2011, ch. 223); Young et al. (2016, ds 2, ch. 247); Ristevski et al. (2018, ds 2, ch. 316); Smith et al. (in review, ds 1, ch. 321); Ősi et al. (in review, ds 1, ch. 366).*  *State (0) occurs in non-crocodylomorphs.*  *State (1) occurs in 'sphenosuchians'.*  *Sstate (2) is a putative apomorphy of Crocodyliformes.*  0. short  1. elongate and expanded posteriorly only  2. elongate and expanded anteriorly and posteriorly |
| 370 | **Coracoid, posteroventral edge, deep groove:**  *Nesbitt (2011, ch. 224); Young et al. (2016, ds 2, ch. 248); Ristevski et al. (2018, ds 2, ch. 317); Smith et al. (in review, ds 1, ch. 322); Ősi et al. (in review, ds 1, ch. 367).*  *State (1) occurs in Rauisuchiae and most ‘sphenosuchians’.*  0. absent  1. present |
| 371 | **Scapula blade:**  *Young et al. (2012, ch. 199 modified); Young (2014, ch. 208, modified); Young et al. (2016, ds 2, ch. 249 modified); Ristevski et al. (2018, ds 2, ch. 318 modified); Smith et al. (in review, ds 1, ch. 323 modified); Ősi et al. (in review, ds 1, ch. 368).*  *State (1) is a putative apomorphy of Teleosauroidea*.  *State (2) is a putative apomorphy of Metriorhynchidae.*  0. scapula blade large: approximately twice the width of the scapular shaft, and generally wider than the distal glenoid region  1. scapula blade reduced: being as wide as, or narrower than, the glenoid region; and the scapular blade is less than 1.5 times the width of the scapular shaft.  2. scapula blade reduced: blade broadens both anteriorly and posteriorly, but is still as wide as, or narrower than, the glenoid region. |
| 372 | **Scapula, anterior and posterior margins in lateral aspect:**  *Young & Andrade (2009, ch. 105 modified); Young et al. (2011, ch. 105 modified); Young et al. (2013a, ch. 167 modified); Young et al. (2012, ch. 200); Young (2014, ch. 209); Young et al. (2016, ds 2, ch. 250); Ristevski et al. (2018, ds 2, ch. 319); Smith et al. (in review, ds 1, ch. 324); Ősi et al. (in review, ds 1, ch. 369).*  0. symmetrically concave in lateral view  1. anterior edge more strongly concave than posterior edge  2. posterior edge more strongly concave than anterior edge |
| 373 | **Scapula, deltoid crest:**  *Young & Andrade (2009, ch. 106); Young et al. (2011, ch. 106); Young et al. (2013a, ch. 168); Young et al. (2012, ch. 201); Young (2014, ch. 210); Young et al. (2016, ds 2, ch. 251); Ristevski et al. (2018, ds 2, ch. 320); Smith et al. (in review, ds 1, ch. 325); Ősi et al. (in review, ds 1, ch. 370).*  0. present  1. absent |
| 374 | **Scapula/Humerus, size:**  *Young (2006, ch. 39); Wilkinson et al. (2008, ch. 68); Young & Andrade (2009, ch. 68); Young et al. (2011, ch. 68); Young et al. (2013a, ch. 169); Young et al. (2012, ch. 202); Young (2014, ch. 211); Young et al. (2016, ds 2, ch. 252); Ristevski et al. (2018, ds 2, ch. 321); Smith et al. (in review, ds 1, ch. 326); Ősi et al. (in review, ds 1, ch. 371).*  0. humerus longer than scapula (greater than 15%)  1. humerus and scapula subequal in length (± 13%)  2. humerus shorter in length than scapula (less than 15%) |
| 375 | **Limb bones (forelimbs), proportional length of ulna relative to the humerus: (ORDERED)**  *Andrade et al. (2011, ch. 452); Ristevski et al. (2018, ds 2, ch. 322); Smith et al. (in review, ds 1, ch. 327); Ősi et al. (in review, ds 1, ch. 372).*  *State (2) is a putative apomorphy of Thalattosuchia (not Teleosauroidea as putatively put forward by Andrade et al., 2011).*  *In Thalattosuchia the ulna is typically between 48%*–*72% of the length of the humerus (perhaps being longer in juvenile specimens).*  *State (2) also occurs in the* Pachycheilosuchus *+* Pietraroiasuchus *clade.*  0. ulna clearly longer than humerus  1. ulna subequal to humerus (distal/proximal = 75–125%)  2. ulna clearly shorter than the humerus |
| 376 | **Humerus, proximal region:**  *Nesbitt (2011, ch. 232 modified); Young et al. (2013a, ch. 170); Young et al. (2012, ch. 203 modified); Young (2014, ch. 212); Young et al. (2016, ds 2, ch. 253 - added state 2); Ristevski et al. (2018, ds 2, ch. 323); Smith et al. (in review, ds 1, ch. 328); Ősi et al. (in review, ds 1, ch. 373).*  *In Thalattosuchia, derived teleosauroids (*Aeolodon priscus, S. bollensis, S. leedsi, S. edwardsi*) have state (2) - the posterior deflection being much more pronounced than in other thalattosuchians.*  *In Geosaurini and Rhacheosaurini taxa change to state (0).*  0. confined to the proximal surface  1. posteriorly expanded and hooked  2. very strongly posteriorly deflected and hooked, with the proximal epiphysis noticeably posterior to the distal epiphysis |
| 377 | **Humerus, proximomedial articular surface:**  *Young & Andrade (2009, ch. 107); Young et al. (2011, ch. 107); Young et al. (2013a, ch. 171); Young et al. (2012, ch. 204); Young (2014, ch. 213); Young et al. (2016, ds 2, ch. 254); Ristevski et al. (2018, ds 2, ch. 324); Smith et al. (in review, ds 1, ch. 329); Ősi et al. (in review, ds 1, ch. 374).*  *State (1) occurs in* Rhacheosaurus *and* Cricosaurus*.*  0. strongly convex  1. weakly convex |
| 378 | **Humerus, deltopectoral crest:**  *Young (2006, ch. 38 modfied); Wilkinson et al. (2008, ch. 66 modified); Young & Andrade (2009, ch. 66 modified); Young et al. (2011, ch. 66 modified); Young et al. (2013a, ch. 172); Young et al. (2012, ch. 205); Young (2014, ch. 214); Young et al. (2016, ds 2, ch. 255); Ristevski et al. (2018, ds 2, ch. 325); Smith et al. (in review, ds 1, ch. 330); Ősi et al. (in review, ds 1, ch. 375).*  *State (1) is a putative apomorphy of Metriorhynchidae.*  *Young et al. (2013a) removed state (2) (absent/vestigial) as metriorhynchids of the subclade Rhacheosaurini do indeed have a deltopectoral crest on their humeri.*  0. present and distinct from the proximal surface  1. present, but continuous with the proximal surface |
| 379 | **Humerus, shape:**  *Young et al. (2012, ch. 206); Young (2014, ch. 215); Young et al. (2016, ds 2, ch. 256); Ristevski et al. (2018, ds 2, ch. 326); Smith et al. (in review, ds 1, ch. 331); Ősi et al. (in review, ds 1, ch. 376).*  *State (1) is a putative apomorphy of Metriorhynchidae.*  *This character helps score the modification of the manus into paddles, and the general reduction of the forelimbs, in Metriorhynchidae.*  0. has typical long bone morphology (longer than wide at distal end)  1. broadly expanded and plate-like |
| 380 | **Humerus, length of the diaphysis relative to total humerus length:**  *Wilkinson et al. (2008, ch. 67); Young & Andrade (2009, ch. 67); Young et al. (2011, ch. 67); Young et al. (2013a, ch. 173); Young et al. (2012, ch. 207); Young (2014, ch. 216); Young et al. (2016, ds 2, ch. 257); Ristevski et al. (2018, ds 2, ch. 327); Smith et al. (in review, ds 1, ch. 332); Ősi et al. (in review, ds 1, ch. 377).*  *This character quantifies the reduction in humeral shaft size in Metriorhynchidae.*  *This character helps score the modification of the manus into paddles, and the general reduction of the forelimbs, in Metriorhynchidae.*  0. diaphysis contributing more than 50% of total humeral length  1. diaphysis contributes 35–38% of total humeral length  2. diaphysis contributes less than 25% of total humeral length |
| 381 | **Humerus-antebrachium joint surface:**  *Young et al. (2011, ch. 180); Young et al. (2013a, ch. 174); Young et al. (2012, ch. 208); Young (2014, ch. 217); Young et al. (2016, ds 2, ch. 258); Ristevski et al. (2018, ds 2, ch. 328); Smith et al. (in review, ds 1, ch. 333); Ősi et al. (in review, ds 1, ch. 378).*  *State (1) is a putative apomorphy of Metriorhynchidae.*  *This character helps score the modification of the manus into paddles, and the general reduction of the forelimbs, in Metriorhynchidae.*  0. complex, allowing one degree of motion – i.e. the humeral epiphyses are ossified  1. planar, limiting possible motion – i.e. the humeral epiphyses are unossified |
| 382 | **Radius and/or ulna, shape:**  *Young (2006, ch. 37); Wilkinson et al. (2008, ch. 65); Young & Andrade (2009, ch. 65); Young et al. (2011, ch. 65 + 176); Young et al. (2013a, ch. 175 + 177); Young et al. (2012, ch. 209 + 211); Young (2014, ch. 218 + 220); Young et al. (2016, ds 2, ch. 259 + 261); Ristevski et al. (2018, ds 2, ch. 329); Smith et al. (in review, ds 1, ch. 334); Ősi et al. (in review, ds 1, ch. 379).*  *State (1) is a putative apomorphy of Metriorhynchidae.*  *This character helps score the modification of the manus into paddles, and the general reduction of the forelimbs, in Metriorhynchidae.*  0. typical long bone morphology (proximodistal length noticeably greater than width at distal end)  1. broadly expanded and plate-like |
| 383 | **Ulna, axis length:**  *Ősi et al. (in review, ds 1, ch. 380).*  *State (1) is a putative apomorphy of Rhacheosaurini.*  *This character helps score the modification of the manus into paddles, and the general reduction of the forelimbs, in Metriorhynchidae.*  0. the proximodistal axis length of the ulna is greater than the length of the anteroposterior axis  1. the anteroposterior axis length of the ulna is greater than the length of the proximodistal axis |
| 384 | **Ulna, morphology of olecranon process:**  *Brochu (1999, ch. 27); Turner & Buckley (2008, ch. 260); Andrade et al. (2011, ch. 457); Ristevski et al. (2018, ds 1, ch. 459); Smith et al. (in review, ds 2, ch. 459); Ősi et al. (in review, ds 1, ch. 381).*  0. narrow and subangular  1. wide and rounded |
| 385 | **Ulna, olecranon process mediolaterally compressed and greatly expanded, creating a very broad proximal ulna:**  *Ősi et al. (in review, ds 1, ch. 382).*  *State (1) occurs in derived teleosauroids.*  *Basal teleosauroids (such as* Platysuchus multiscorbiculatus *and* Steneosaurus bollensis*) score as (0).*  0. no  1. yes |
| 386 | **Radiale and/or ulnare, shape:**  *Young et al. (2011, ch. 177 + 179); Young et al. (2013a, ch. 176 + 178); Young et al. (2012, ch. 210 + 212); Young (2014, ch. 219 + 221); Young et al. (2016, ds 2, ch. 260 + 262); Ristevski et al. (2018, ds 2, ch. 330); Smith et al. (in review, ds 1, ch. 335); Ősi et al. (in review, ds 1, ch. 383).*  *State (1) is a putative apomorphy of Metriorhynchidae.*  *This character helps score the modification of the manus into paddles, and the general reduction of the forelimbs, in Metriorhynchidae.*  0. typical long bone morphology (proximodistal length noticeably greater than width at distal end)  1. broadly expanded and plate-like |
| 387 | **Manus, metacarpal general structure: (*)**  *Buscalioni (2017, ch. 424 part modified); Ősi et al. (in review, ds 1, ch. 384).*  *Here we have modified the character from Buscalioni (2017) to help quantify the manus morphological changes occurring at the transition from basal crocodyliforms to metasuchians. Here it samples overall robustness, not relative length.*  *This character is not applicable for taxa that do not have all five manual digits.*  0. metacarpals IV and V not strongly differentiated from II-III in terms of overall robusticity  1. metacarpals II-III are noticeably more robust than those of IV-V (due to metacarpal I being greatly enlarged relative to all other metacarpals in some clades, it is not used in this character) |
| 388 | **Manus, shape of metacarpal I: (*)**  *Young (2006, ch. 41); Wilkinson et al. (2008, ch. 70); Young & Andrade (2009, ch. 70); Young et al. (2011, ch. 70); Young et al. (2013a, ch. 179); Young et al. (2012, ch. 213); Young (2014, ch. 222); Young et al. (2016, ds 2, ch. 263); Ristevski et al. (2018, ds 2, ch. 331); Smith et al. (in review, ds 1, ch. 336); Ősi et al. (in review, ds 1, ch. 385).*  *State (1) is a putative apomorphy of Metriorhynchidae.*  *This character helps score the modification of the manus into paddles, and the general reduction of the forelimbs, in Metriorhynchidae.*  *This character is not applicable for taxa that lack digit I.*  0. elongate, more than twice as long as wide  1. broadly expanded, maximum width at least 60% of total length |
| 389 | **Manus, digit I:**  *Ősi et al. (in review, ds 1, ch. 386).*  *State (1) is a putative apomorphy of* Junggarsuchus*.*  *This character helps score the modification of the manus into being functionally tridactyl.*  0. present  1. absent |
| 390 | **Manus, relative length of digit V: (*)**  *Buscalioni (2017, ch. 424 part modified); Ősi et al. (in review, ds 1, ch. 387).*  *Here we have modified the character from Buscalioni (2017) to help quantify the manus morphological changes occurring at transition from basal crocodyliforms to metasuchians.*  *This character is not applicable for taxa that do not have all five manual digits.*  0. digit V longer than digit I, being comparable in length to digits II-IV  1. digit V reduced in length, being evidently shorter than digits II-IV and comparable in length to digit I |

**Appendicular skeleton: pelvic girdle and hind limbs** (Ch. 389 – 425; 8.150% of characters)

*[pelvic elements (ossa pubes, ossa ilia, & ossa ischia); stylopodia (ossa femora), zeugopodia (ossa tibiae), autopodia (ossa calcis, ossa metatarsalia, & ossa digitorum pedis)]*

| # | Description |
| --- | --- |
| 391 | **Pubis, exclusion from acetabulum:**  *Turner & Sertich (2010, ch. 86 part); Andrade et al. (2011, ch. 445); Young et al. (2013a, ch. 180 part); Young et al. (2012, ch. 214 part); Young (2014, ch. 223 part); Young et al. (2016, ds 2, ch. 264 part); Ristevski et al. (2018, ds 2, ch. 332); Smith et al. (in review, ds 1, ch. 337); Ősi et al. (in review, ds 1, ch. 388).*  *Following Claesson (2004) state (1) occurs in Crocodyliformes.*  *This character scores the pubis articulation with the acetabulum (state 0), and the mobile pubis articulating with the ischium anterior process (state 1).*  0. pubis not excluded, participating at least marginally to the anteroventral rim of the acetabulum  1. pubis excluded, acetabulum composed exclusively by the ischium and illium |
| 392 | **Pubis, presence of exclusive proximal contact with ischium:**  *Andrade et al. (2011, ch. 446) – based on Andrews (1913) and Clark (1994, ch. 86); Ristevski et al. (2018, ds 2, ch. 333); Smith et al. (in review, ds 1, ch. 338); Ősi et al. (in review, ds 1, ch. 389).*  *Note that in Metasuchia this character correlates with the pubic exclusion from the acetabulum; however, thalattosuchians also have the pubis excluded from the acetabulum, but the pubis articulates between the ischium pubic process and the ilium anterior peduncle.*  0. absent, pubis supported by both ilium and ischium  1. present, proximal head of pubis contacts only the ischium |
| 393 | **Pubis, length:**  *Nesbitt (2011, ch. 278); Young et al. (2016, ds 2, ch. 265); Ristevski et al. (2018, ds 2, ch. 334); Smith et al. (in review, ds 1, ch. 339); Ősi et al. (in review, ds 1, ch. 390).*  *State (0) is a putative apomorphy of Crocodyliformes.*  0. less than 70% of femoral length  1. 70% or more of femoral length |
| 394 | **Pubis, expansion of distal end:**  *Clark (1994, ch. 85 modified); Andrade et al. (2011, ch. 447 modified); Nesbitt (2011, ch. 283 modified); Ristevski et al. (2018, ds 2, ch. 335); Smith et al. (in review, ds 1, ch. 340); Ősi et al. (in review, ds 1, ch. 391).*  *Note that* Postosuchus *has a pubic boot (along with other non-crocodylomorph pseudosuchians; Nesbitt, 2011; Weinbaum, 2013). Here we test the homology of this pubic boot with that seen in crocodylomorphs (the* Protosuchus *distal expansion, and the ‘fan’-like pubic blade seen in other crocodyliforms). Nesbitt (2011) reports that a small posterior expansion is present in the holotype of* Hesperosuchus agilis*, suggesting the lack of an expansion in* Terristrisuchus *is apomorphic.*  *State (2) is a putative apomorphy of Mesoeucrocodylia.*  0. absent  1. expanded relative to the shaft (= pubic boot)  2. a “fan-like” expansion creating a distinct pubic blade |
| 395 | **Pubis, presence of an obturator foramen:**  *Leardi et al. (2017, ch. 126); Ősi et al. (in review, ds 1, ch. 392).*  *State (1) occurs in Crocodyliformes.*  0. present  1. absent |
| 396 | **Ilium, presence of a distinct anterior acetabular flange, created by the anterior acetabular margin projecting anteriorly such that it is anterior to the iliac anterior margin:**  *Smith et al. (in review, ds 1, ch. 341); Ősi et al. (in review, ds 1, ch. 393).*  *State (1) occurs in basal metriorhynchoids*.  *Note, this condition is different from that of* Dyrosaurus maghribensis*, as there the entire anterior margin of the ilium bulges anteriorly, not just the acetabular margin (which in* Pelagosaurus typus *creates the thin acetabular flange).*  0. absent  1. present |
| 397 | **Ilium, relative length of anterior and posterior processes: (*)**  *Clark (1994, ch. 84); Lauprasert et al. (2007, ch. 68); Andrade et al. (2011, ch. 441); Ristevski et al. (2018, ds 1, ch. 441); Smith et al. (in review, ds 2, ch. 441); Ősi et al. (in review, ds 1, ch. 394).*  *This character is not applicable for taxa that lack the posterior process of the ilium.*  0. subequal, anterior and posterior processes similar in length  1. unequal, with anterior process relatively small, one quarter or less than the length of the posterior process |
| 398 | **Ilium, presence of indentation at the dorsal margin of iliac blade:**  *Brochu (1999, ch. 28 modified, part); Andrade et al. (2011, ch. 442); Ristevski et al. (2018, ds 1, ch. 442); Smith et al. (in review, ds 2, ch. 442); Ősi et al. (in review, ds 1, ch. 395).*  *Andrade et al. (2011) divded this character to separate diverse aspects of the morphology of the anterior end of iliac blade. This character samples the indentation at the dorsal edge of the anterior process.*  0. absent, dorsal edge convex or straight in lateral view  1. present as a shallow or modest dorsal indentation  2. present as a strong dorsal indentation (“wasp-waisted”) |
| 399 | **lium, presence of a distinct 'bulge' that fuses the anterior regions of the supraacetabular and dorsal iliac crests: (*)**  *Ristevski et al. (2018, ds 2, ch. 336); Smith et al. (in review, ds 1, ch. 342); Ősi et al. (in review, ds 1, ch. 396).*  *State (1) occurs in* Anteophthalmosuchus hooleyi *and* Crocodylus.  *This character is not applicable for taxa that lack the dorsal iliac crest.*  0. anterior region of the supraacetabular crest does not fuse with the anterior margin of the iliac dorsal crest, as there is no anterior ‘bulge’  1. anterior region of the crest bulges laterally (slightly overhanging the acetabular fossa), and is contiguous with the anterior margin of the iliac dorsal crest |
| 400 | **Ilium, postacetabular (= posterior) process presence:**  *Young & Andrade (2009, ch. 128 modified), Young et al. (2011, ch. 128 modified); Young et al. (2013a, ch. 181 modified); Young et al. (2012, ch. 215 modified); Young (2014, ch. 224); Wilberg (2015b, ch. 368); Young et al. (2016, ds 2, ch. 266 modified); Ristevski et al. (2018, ds 2, ch. 337); Smith et al. (in review, ds 1, ch. 343); Ősi et al. (in review, ds 1, ch. 397).*  *State (1) is a putative apomorphy of Metriorhynchidae.*  0. present  1. absent/extremely reduced |
| 401 | **Ilium, postacetabular (= posterior) process expanded into a thin “fan”-shape: (*)**  *Young et al. (2012, ch. 216); Young (2014, ch. 225); Wilberg (2015b, ch. 369); Young et al. (2016, ds 2, ch. 267); Ristevski et al. (2018, ds 2, ch. 338); Smith et al. (in review, ds 1, ch. 344); Ősi et al. (in review, ds 1, ch. 398).*  *State (1) is a putative apomorphy of derived teleosauroids (not seen in basal taxa* Platysuchus multiscrobiculatus, Teleosaurus cadomensis, Steneosaurus gracilirostris *and* S. bollensis *where the process is still elongate and distinctly process-like). This structure is a modification of the postacetabular (=posterior) process in these taxa.*  *This character is not applicable for taxa that lack the postacetabular process.*  0. no  1.yes, posterior margin is expanded (typically resembling a “fan”-shape), being mediolaterally compressed and extends from the iliac crest towards the posterior peduncle |
| 402 | **Ilium, postacetabular (= posterior) process, presence of constrictions (‘wasp-waisting’) on both the dorsal and ventral margins near the distal terminus: (*)**  *Ristevski et al. (2018, ds 2, ch. 339); Smith et al. (in review, ds 1, ch. 345); Ősi et al. (in review, ds 1, ch. 399).*  *State (1) occurs in* Anteophthalmosuchus epikrator *and* Crocodylus*.*  *This character is not applicable for taxa that lack the postacetabular process.*  0. absent  1. present |
| 403 | **Illium, size:**  *Young (2006, ch. 42); Wilkinson et al. (2008, ch. 71); Young & Andrade (2009, ch. 71); Young et al. (2011, ch. 71); Young et al. (2013a, ch. 182); Young et al. (2012, ch. 217); Young (2014, ch. 226); Young et al. (2016, ds 2, ch. 268); Ristevski et al. (2018, ds 2, ch. 340); Smith et al. (in review, ds 1, ch. 346); Ősi et al. (in review, ds 1, ch. 400).*  *State (1) is a putative apomorphy of Metriorhynchidae.*  0. large (length of dorsal border more than 28%, and typically at least 30% of femur length)  1. small (length of dorsal border less than 21% of femur length) |
| 404 | **Ilium, in lateral view, the orientation of the dorsal margin of the articulation facet that contributes to the acetabulum is:**  *Young (2014, ch. 227); Young et al. (2016, ds 2, ch. 269); Ristevski et al. (2018, ds 2, ch. 341); Smith et al. (in review, ds 1, ch. 347); Ősi et al. (in review, ds 1, ch. 401).*  *State (1) is a putative autapomorphy of* Tyrannoneustes lythdrodectikos.  0. ventrally orientated  1. horizontally orientated |
| 405 | **Ilium, dorsal border length in lateral view:**  *Young (2014, ch. 228); Young et al. (2016, ds 2, ch. 270); Ristevski et al. (2018, ds 2, ch. 342); Smith et al. (in review, ds 1, ch. 348); Ősi et al. (in review, ds 1, ch. 402).*  *State (1) is a putative autapomorphy of* Tyrannoneustes lythdrodectikos.  0. long, terminates at least level to the articulation facet that contributes to the acetabulum  1. short, terminates prior to the articulation facet that contributes to the acetabulum |
| 406 | **Ilium, ventral margin:**  *Ristevski et al. (2018, ds 2, ch. 343); Smith et al. (in review, ds 1, ch. 349); Ősi et al. (in review, ds 1, ch. 403).*  *State (1) is a putative apomorphy of Metriorhynchidae.*  0. distinct ilium and ischium peduncles separated by an acetabular incision/depression  1. lacks an acetabular depression, with the peduncles being contiguous with the ventral margin |
| 407 | **Ischium, presence of pubic (= anterior) process:**  *Andrade et al. (2011, ch. 444) – reformulated from Clark (1994, ch. 86) and Andrews (1913); Ristevski et al. (2018, ds 1, ch. 446); Smith et al. (in review, ds 2, ch. 446); Ősi et al. (in review, ds 1, ch. 404).*  0. pubic process absent, or incipient and small, not restricting the participation of the pubis to the acetabulum  1. anterior process well developed, robust and with a round head, at least partially restricting the participation of pubis in the acetabulum |
| 408 | **Ischium, morphology of pubic (= anterior) process: (*)**  *Young (2006, ch. 43); Wilkinson et al. (2008, ch. 72); Young & Andrade (2009, ch. 72); Young et al. (2011, ch. 72); Young et al. (2013a, ch. 183); Young et al. (2012, ch. 218); Young (2014, ch. 229); Young et al. (2016, ds 2, ch. 271); Ristevski et al. (2018, ds 2, ch. 344); Smith et al. (in review, ds 1, ch. 350); Ősi et al. (in review, ds 1, ch. 405).*  *State (1) is a putative apormorphy of Metriorhynchidae.*  *State (2) is a putative apomorphy of* Cricosaurus*.*  *This character is not applicable for taxa that lack, or have incipient pubic processes.*  0. developed – with clearly defined articulation facets for pubis and ilium; additionally, anterior process is at least half as wide as the posterior process  1. reduced – lacks both articulation facets, and is between 30–50% as wide as the posterior process  2. highly reduced – lacking both articulation facets, and is less than 25% as wide as the posterior process |
| 409 | **Ischium, morphology of anterior process of iliac blade, in lateral view:**  *Brochu (1999, ch. 28 modified, part); Andrade et al. (2011, ch. 443); Ristevski et al. (2018, ds 1, ch. 443); Smith et al. (in review, ds 2, ch. 443); Ősi et al. (in review, ds 1, ch. 406).*  *Andrade et al. (2011) divded this character to separate diverse aspects of the morphology of the anterior end of iliac blade. This character samples the morphology of the anterior process. Among eusuchians, state (1) is a somewhat generalised condition; state (0) is putative apomorphy of* Paleosuchus*; and state (2) is putative apomorphy of* Diplocynodon*.*  0. very narrow relative the main body of the iliac blade  1. rounded and moderately broad relative the main body of the iliac blade  2. very broad and deep, at least half the height of the main body of the iliac blade |
| 410 | **Limb bones, length relative to trunk, at maturity: (ORDERED)**  *Brochu (1999, ch. 33 modified); Andrade et al. (2011, ch. 448); Ristevski et al. (2018, ds 1, ch. 450); Smith et al. (in review, ds 2, ch. 450); Ősi et al. (in review, ds 1, ch. 407).*  *Andrade et al. (2011) modified this character to sample length relative to trunk, not overall robustness.*  *Within Eusuchia, Brochu (1999) considers that state (2) only occurs in* Borealosuchus*.*  0. limb bones relatively short  1. limb bones moderately long  2. limb bones very long |
| 411 | **Limb bones, general structure:**  *Brochu (1999, ch. 33 part); Andrade et al. (2011, ch. 449); Ristevski et al. (2018, ds 1, ch. 451); Smith et al. (in review, ds 2, ch. 451); Ősi et al. (in review, ds 1, ch. 408).*  *Andrade et al. (2011) modified this character was to sample overall robustness, not relative length. Within Eusuchia, Brochu (1999) considers that state (2) only occurs in* Borealosuchus*.*  0. limb bones robust  1. limb bones overall slender, but not weak  2. gracile |
| 412 | **Limb bones, relative length of forelimbs/hindlimbs (*= humerus + radius : femur + tibia*):**  *Brochu (1999, ch. 33 part); Young & Andrade (2009, ch. 109 modified); Andrade et al. (2011, ch. 450 modified); Nesbitt (2011, ch. 212 modified); Young et al. (2011, ch. 109 modified); Young et al. (2013a, ch. 195 modified); Young et al. (2012, ch. 230 modified); Young (2014, ch. 241 modified); Young et al. (2016, ds 2, ch. 284 modified); Ristevski et al. (2018, ds 2, ch. 345); Smith et al. (in review, ds 1, ch. 351); Ősi et al. (in review, ds 1, ch. 409).*  *Andrade et al. (2011) modified the crocodylomorph variant of this character to sample relative length of limbs, not robustness or limb/trunk relative length. This version of the character is an amalgam of the ones in Andrade et al. (2011) and Nesbitt (2011), the latter which Young et al. (2016, ds 2) modified to include extra states to reflect the forelimb reduction in Thalattosuchia.*  *This character does not consider the autopodia (manus and pes), only the relation between the stylopodia and zeugopodia (humerus + ulna and femur + tibia, respectively).*  *States (3 + 4) reflects the extreme conditions found in Thalattosuchia. State (4) evolved twice, once in Metriorhynchidae, and also in derived teleosauroids (the Middle Jurassic ‘*Steneosaurus’ *clade).*  *Note that basal thalattosuchians (e.g.* Steneosaurus bollensis, Platysuchus *and* Pelagosaurus*) have state (2).*  *State (2) also occurs in* Gavialis *and* Terminonaris*.*  *State (3) also evolved in the* Pachycheilosuchus *+* Pietraroiasuchus *clade.*  *Basal crocodylomorphs also share state (2), while state (3) occurs in* Postosuchus*.*  *Within Eusuchia, Brochu (1999) considers that state (0) only occurs in* Borealosuchus*.*  0. forelimb and hindlimb subequal in length at maturity  1. forelimb slightly shorter than hindlimb at maturity  2. forelimb shorter than hindlimb at maturirty (between 90 and 55%)  3. forelimb noticeably shorter than hindlimb at maturity (between 45 and 55%)  4. forelimb significantly shorter than hindlimb at maturity (less than 45%) |
| 413 | **Limb bones (hindlimbs), proportional length of tibia relative to the femur: (ORDERED)**  *Clark et al. (2000, ch. 31 modified); Clark & Sues (2002, ch. 32 modified); Sues et al. (2003, ch. 32 modified); Clark et al. (2004, ch. 32 modified); Young (2006, ch. 44 modified); Wilkinson et al. (2008, ch. 73 modified); Young & Andrade (2009, ch. 73 modified); Andrade et al. (2011, ch. 453 modified); Young et al. (2012, ch. 225 + 231 modified); Pol et al. (2013, ch. 32 modified); Young et al. (2016, ds 2, ch. 278 modified); Leardi et al. (2017, ch. 32 modified); Ristevski et al. (2018, ds 1, ch. 455 modified); Smith et al. (in review, ds 2, ch. 455 modified); Ősi et al. (in review, ds 1, ch. 410).*  *This version of the character is an amalgam of the ones in Andrade et al. (2011), Young et al. (2016) and Leardi et al. (2017).*  *This character is designed to help elucidate variation in the proportions of the hind limb, and the changes that occur in Thalattosuchia (where the femur can be almost twice the size of tibia, i.e. in Metriorhynchidae). Thus states (2-5) are putative apomorphies of Thalattosuchia.*  *State (0) occurs in* Terrestrisuchus, *Hallopodidae, and* Gobiosuchus*.*  *In Thalattosuchia, state (4) is a putative apomorphy of both Metriorhynchinae and* Aeolodon priscus*, with derived metriorhynchines being state (5). Middle Jurassic teleosauroids (and the Late Jurassic genus* Machimosaurus*) and Geosaurinae score as state (3).*  *Thus, this character is scoring for the independent regression of the tibia (as a proportion of the hind limb) in Teleosauroidea and Metriorhynchidae.*  *State (2) also occurs in Dyrosauridae and* Terminonaris*.*  0. length uneven, tibia slightly longer than the femur (distal/proximal more than 105%)  1. tibia subequal to femur, or only slightly shorter (distal/proximal c. 75-100%)  2. length uneven, tibia evidently shorter than the femur (distal/proximal c. 50-74%)  3. length uneven, tibia evidently shorter than the femur (distal/proximal c. 40-50%)  4. length uneven, tibia evidently shorter than the femur (distal/proximal c. 30-40%)  5. length uneven, tibia evidently shorter than the femur (distal/proximal less than 30%) |
| 414 | **Femur, relative orientation between the proximal and distal heads:**  *Ortega et al. (2000, ch. 149), Andrade et al. (2011, ch. 455); Ristevski et al. (2018, ds 1, ch. 457); Smith et al. (in review, ds 2, ch. 457); Ősi et al. (in review, ds 1, ch. 411).*  0. femur with light torsion, proximal and distal articulation facets approximately at 30 degrees or less from each other  1. femur with evident torsion, proximal and distal articulation facets approximately at 60 degrees from each other |
| 415 | **Femur, general shape:**  *Andrade et al. (2011, ch. 464 modified); Ristevski et al. (2018, ds 2, ch. 347 modified); Smith et al. (in review, ds 1, ch. 353 modified); Ősi et al. (in review, ds 1, ch. 412).*  *State (1) is a putative apomorphy of Thalattosuchia.*  0. sigmoidal shape formed by either an unequal proximal and distal curvature, or a strong sigmoidal shape  1. sigmoidal shape formed by comparable curvatures proximally and distally, and forms a shallow ‘S’-shape |
| 416 | **Femur, proximal portion, posteromedial tuber:**  *Nesbitt (2011, ch. 301 modified – character states re-ordered); Young et al. (2013a, ch. 184); Young et al. (2012, ch. 219); Young (2014, ch. 230); Young et al. (2016, ds 2, ch. 272); Ristevski et al. (2018, ds 2, ch. 348); Smith et al. (in review, ds 1, ch. 354); Ősi et al. (in review, ds 1, ch. 413).*  *State (2) is a putative apomorphy of Metriorhynchoidea.*  *State (2) also occurs in non-paracrocodylomorph pseudosuchians.*  0. absent  1. present, and small  2. present, and largest of the proximal tubera |
| 417 | **Femur, proximal condylar fold:**  *Nesbitt (2011, ch. 312); Young et al. (2013a, ch. 185); Young et al. (2012, ch. 220); Young (2014, ch. 231); Young et al. (2016, ds 2, ch. 273); Ristevski et al. (2018, ds 2, ch. 349); Smith et al. (in review, ds 1, ch. 355); Ősi et al. (in review, ds 1, ch. 414).*  *State (1) occurs in Paracrocodylomorpha.*  *The proximal condylar fold is a straight ridge that connects the medioventral portion of the ventral head with the shaft on the anterolateral surface of the femur (Nesbitt 2011: p. 149).*  *Note that this fold can be hard to discern in Metriorhynchidae. It is possible that derived species of* Cricosaurus *lack this fold.*  0. absent  1. present |
| 418 | **Femur, ridge of attachment for the *M. caudofemoralis*:**  *Young & Andrade (2009, ch. 108 modified); Nesbitt (2011, ch. 315 modified); Young et al. (2011, ch. 108 modified); Young et al. (2013a, ch. 186); Young et al. (2012, ch. 221); Young (2014, ch. 232); Young et al. (2016, ds 2, ch. 274); Ristevski et al. (2018, ds 2, ch. 350); Smith et al. (in review, ds 1, ch. 356); Ősi et al. (in review, ds 1, ch. 415).*  *We follow Young et al. (2016, ds 2) in scoring thalattosuchians as state (0). Thalattosuchians lack a fourth trochanter sensu stricto, as they only have a large flattened rugose area for the muscle attachment, not a distinct process. Thus state (0) is a putative apomorphy of Thalattosuchia.*  0. absent, flattened rugose area  1. low and without a distinct medial asymmetrical apex (= fourth trochanter)  2. bladelike with a distinct asymmetric apex located medially |
| 419 | **Lateral edge of proximal articular surface of femur (lesser trochanter):**  *Young & Andrade (2009, ch. 117); Young et al. (2011, ch. 117); Young et al. (2013a, ch. 187); Young et al. (2012, ch. 222); Young (2014, ch. 233); Young et al. (2016, ds 2, ch. 275); Ristevski et al. (2018, ds 2, ch. 351); Smith et al. (in review, ds 1, ch. 357); Ősi et al. (in review, ds 1, ch. 416).*  *State (1) occurs in Metasuchia.*  0. rounded  1. ‘squared’ with enlarged scar for *Musculus ischiotrochantericus* |
| 420 | **Femur, medial condyle of the distal portion:**  *Nesbitt (2011, ch. 320 modified); Young et al. (2013a, ch. 188 modified); Young et al. (2012, ch. 223 modified); Young (2014, ch. 234 modified); Young et al. (2016, ds 2, ch. 276 modified); Ristevski et al. (2018, ds 2, ch. 352 modified); Smith et al. (in review, ds 1, ch. 358); Ősi et al. (in review, ds 1, ch. 417).*  *State (0) occurs in basal pseudosuchians.*  *State (1) occurs in Postosuchidae + Crocodylomorpha.*  *State (2) occurs in Metriorhynchidae.*  0. tapers to a point on the medial portion in distal view  1. smoothly rounded in distal view  2. condyle incompletely ossified, and typically poorly developed |
| 421 | **Femur, distal surface between the lateral and medial condyles:**  *Nesbitt (2011, ch. 321); Young et al. (2013a, ch. 189); Young et al. (2012, ch. 224); Young (2014, ch. 235); Young et al. (2016, ds 2, ch. 277); Ristevski et al. (2018, ds 2, ch. 353); Smith et al. (in review, ds 1, ch. 359); Ősi et al. (in review, ds 1, ch. 418).*  *State (1) occurs in crocodyliforms, and some ‘sphenosuchians’.*  *Within Crocodyliformes, state (0) is a putative apomorphy of Metriorhynchidae.*  0. nearly flat or flat  1. groove separating the medial condyle from the lateral condyle |
| 422 | **Calcaneum tuber, development:**  *Young (2006, ch. 45 modified); Wilkinson et al. (2008, ch. 74 modified); Young & Andrade (2009, ch. 74 modified); Andrade et al. (2011, ch. 466); Young et al. (2011, ch. 74 modified); Young et al. (2013a, ch. 191 modified); Young et al. (2012, ch. 226 modified); Young (2014, ch. 237 modified); Young et al. (2016, ds 2, ch. 279 - rephrased); Ristevski et al. (2018, ds 2, ch. 354); Smith et al. (in review, ds 1, ch. 360); Ősi et al. (in review, ds 1, ch. 419).*  *This character scores the regression of the tuber in metriorhynchines. Whether the calcaneal tuber regresses in geosaurine metriorhynchids is currently unknown.*  0. well developed with a long neck (typically subequal in length to main body of calcaneum)  1. poorly developed with a short neck (less than half length of calcaneum main body, and projects out in one plane from the calcaneum main body) |
| 423 | **Pes, length of metatarsals: (ORDERED)**  *Young (2006, ch. 46 modified); Wilkinson et al. (2008, ch. 75 modified); Young & Andrade (2009, ch. 75 modified); Young et al. (2011, ch. 75 modified); Young et al. (2013a, ch. 192 modified); Young et al. (2012, ch. 227 modified); Young (2014, ch. 238 modified); Young et al. (2016, ds 2, ch. 280 modified); Ristevski et al. (2018, ds 2, ch. 355 modified); Smith et al. (in review, ds 1, ch. 361 modified); Ősi et al. (in review, ds 1, ch. 420).*  *States (1-2) occur in Metriorhynchoidea.*  *State (2) occurs in Metriorhynchidae.*  *This character helps score the modification of the pes into paddles in Metriorhynchoidea.*  0. metatarsals I–IV longer than their repective digit phalanges (greater than 20%)  1. metatarsals II–IV sub-equal in length to their repspective digit phalanges (± 10%)  2. metatarsals II–IV shorter than their respective digit phalanges (less than 90%) |
| 424 | **Pes, proximal morphology of metatarsal I: (ORDERED)**  *Young (2006, ch. 47 modified); Wilkinson et al. (2008, ch. 76 modified); Young & Andrade (2009, ch. 76 modified); Andrade et al. (2011, ch. 467 modified); Young et al. (2011, ch. 76 modified); Young et al. (2013a, ch. 193 modified); Young et al. (2012, ch. 228 modified); Young (2014, ch. 239 modified); Young et al. (2016, ds 2, ch. 281 modified); Ristevski et al. (2018, ds 2, ch. 356 modified); Smith et al. (in review, ds 1, ch. 362 modified); Ősi et al. (in review, ds 1, ch. 421).*  *States (1-4) occur in Metriorhynchoidea.*  *This character scores the broadening of metatarsal I seen in metriorhynchines. The pes of geosaurine metriorhynchid is currently unknown.*  *This character helps score the modification of the pes into paddles in Metriorhynchoidea.*  0. proximal end not enlarged (typically no more than 10%, but depending on preservation up to 20%, wider than any other metatarsal)  1. proximal end enlarged (25-30% wider)  2. proximal end moderately enlarged (45-55% wider)  3. proximal end greatly enlarged (more than 75% wider) |
| 425 | **Pes, relative length of digits III and IV:**  *Young (2006, ch. 48); Wilkinson et al. (2008, ch. 77); Young & Andrade (2009, ch. 77); Andrade et al. (2011, ch. 465); Young et al. (2011, ch. 77); Young et al. (2013a, ch. 194); Young et al. (2012, ch. 229); Young (2014, ch. 240); Young et al. (2016, ds 2, ch. 283); Ristevski et al. (2018, ds 2, ch. 357); Smith et al. (in review, ds 1, ch. 363); Ősi et al. (in review, ds 1, ch. 422).*  *In crocodyliforms, the digits are usually in the following descending order: III-IV-II-I.*  *State (1) is putative apomorphy of Metriorhynchoidea, and with digit length arranged as IV-III-II-I (see Young & Andrade 2009, Appendix 2). Previously this has been considered to be a metriorhynchid apomorphy.*  *This character helps score the modification of the pes into paddles in Metriorhynchoidea.*  0. digit III is longer than digit IV  1. digit IV is longer than digit III (digit IV elongated, helping to create a paddle) |
| 426 | **Pes, digit IV, number of phalanges: (ORDERED)**  *Nesbitt (2011, ch. 396 modified); Ristevski et al. (2018, ds 2, ch. 358); Smith et al. (in review, ds 1, ch. 364); Ősi et al. (in review, ds 1, ch. 423).*  *State (0) is a putative apomorphy of* Postosuchus*.*  *State (1) occurs in most archosauriforms.*  *State (2) is a putative apomorphy of Crocodylomorpha.*  *Ristevski et al. (2018, ds 2) added state (0) as six pedal digit IV phalanges have been reported for specimens of* P. alisonae *Peyer* *et al*. *(2008) and* P. kirkpatricki *(Weinbaum, 2013).*  0. six  1. five  2. four or fewer |
| 427 | **Pes, digit V, metatarsals and phalanges:**  *Clark (1994, ch. 88 modified); Nesbitt (2011, ch. 399 re-phrased); Young et al. (2016, ds 2, ch. 282); Ristevski et al. (2018, ds 2, ch. 359); Smith et al. (in review, ds 1, ch. 365); Ősi et al. (in review, ds 1, ch. 424).*  *State (0) occurs in non-crocodylomorphs.*  *State (1) occurs in ‘sphenosuchians’.*  *State (2) is a putative apomorphy of Crocodyliformes.*  0. present and ‘‘fully’’ developed first phalanx  1. present and ‘‘poorly’’ developed first phalanx  2. without phalanges and metatarsal tapers to a point |

**Dermal ossifications: osteoderms** (Ch. 426 – 449; 5.286% of characters)

| # | Description |
| --- | --- |
| 428 | **Ornamentation (dorsal osteoderms), type of sculpture: (*)**  *Ortega et al. (2000, ch. 111); Andrade et al. (2011, ch. 19); Ristevski et al. (2018, ds 2, ch. 360); Smith et al. (in review, ds 1, ch. 366); Ősi et al. (in review, ds 1, ch. 425).*  *Ornamentation on the osteoderms is always present, and only in two possible forms. Note that Turner & Buckley (2008) considered that* Araripesuchus gomesii *and (possibly)* A. tsangatsangana *displayed the ‘fleur de lys’ pattern (anterolaterally and anteromedially directed “ridges”; Osmólska et al., 1997), according to the character by Pol & Norell (2004b, ch188). We consider that this pattern regards the disposition of the sculpturing (fabric), not the type of sculpturing.*  *This character is not* *applicable for taxa that lack dorsal osteoderms.*  0. vermiform-dendritic pattern  1. pitted pattern |
| 429 | **Ornamentation (dorsal osteoderms), distribution of pits on dorsal surface: (*)**  *Young et al. (2011, ch. 185 modified); Young et al. (2013a, ch. 201 modified); Young et al. (2012, ch. 239 modified); Young (2014, ch. 250 modified); Young et al. (2016, ds 2, ch. 297 modified); Ristevski et al. (2018, ds 2, ch. 361 modified); Smith et al. (in review, ds 1, ch. 367); Ősi et al. (in review, ds 1, ch. 426).*  *State (2) is a putative autapomorphy of* Magyarosuchus fitosi*.*  *State (3) is a putative apomorphy of Machimosaurini*.  *Ősi et al. (in review) added state (2) to accommodate the unusual osteoderm pit morphology seen in* Magyarosuchus fitosi*.*  *This character is not* *applicable for taxa that lack dorsal osteoderms, or pitted ornamentation.*  0. small round to ellipsoid pits, very densely distributed  1. large round to ellipsoid pits, well separated from one another  2. irregularly shaped pits (including circular, ellipsoid, bean-shaped, triangular and quadrangular shapes), with an extreme variation in size (from small to very large), with elongate pits present on the ventrolateral surface running from the keel to the lateral margin  3. pits variable in size and length, from small to large, but on osteoderms with a keel, the pits can become elongate grooves, especially along the lateral margins |
| 430 | **Presacral osteoderms, dorsal to the vertebral column:**  *Clark (1994, ch. 100 modified); Brochu (1999, ch. 39 part); Young (2006, ch. 51); Wilkinson et al. (2008, ch. 80); Young & Andrade (2009, ch. 80); Andrade et al. (2011, ch. 468 part); Nesbitt (2011, ch. 401); Young et al. (2011, ch. 80); Young et al. (2013a, ch. 196 part); Young et al. (2012, ch. 232 part); Young (2014, ch. 243 part); Wilberg (2015b, ch. 382); Young et al. (2016, ds 2, ch. 285); Wilberg (2017, ch. 394); Ristevski et al. (2018, ds 2, ch. 362); Smith et al. (in review, ds 1, ch. 368); Ősi et al. (in review, ds 1, ch. 427).*  *State (0) occurs in* Junggarsuchus *and Metriorhynchidae.*  0. absent  1. present |
| 431 | **Presacral ventral osteoderms (= gastral osteoderms), form a carapace in the trunk region:**  *Young (2006, ch. 50 modified); Wilkinson et al. (2008, ch. 79 modified); Young & Andrade (2009, ch. 79 modified); Andrade et al. (2011, ch. 468 part); Nesbitt (2011, ch. 409 re-phrased); Young et al. (2011, ch. 79 modified); Young et al. (2013a, ch. 199); Young et al. (2012, ch. 236 modified); Young (2014, ch. 247 modified); Young et al. (2016, ds 2, ch. 294); Ristevski et al. (2018, ds 2, ch. 374); Smith et al. (in review, ds 1, ch. 380); Ősi et al. (in review, ds 1, ch. 428).*  *Crocodyliformes have state (1), although with reversions.*  0. absent  1. present |
| 432 | **Nuchal armour, relation of nuchal osteoderms with the remaining dorsal armour and skull: (*)**  *Brochu (1999, ch. 38 modified, part); Andrade et al. (2011, ch. 469); Ristevski et al. (2018, ds 2, ch. 363); Smith et al. (in review, ds 1, ch. 369); Ősi et al. (in review, ds 1, ch. 429).*  *Note that a similar character was devised by Ortega et al. (2000, ch. 109), but to unite the undescribed Itaborai form and* Sebecus*. See also McAliley et al. (2006)* *for discussion on eusuchians*.  *This character is not* *applicable for taxa that lack dorsal osteoderms.*  0. large nuchal shields continuous from postoccipital region to trunk armour, with any given osteoderm contacting the anterior and posterior elements (except for the first postoccipital shield)  1. large nuchal shields continuous with trunk armour, but not reaching the postoccipital region  2. large nuchal shields discontinuous with dorsal trunk armour and absent from postoccipital region |
| 433 | **Nuchal armour, number and arrangement of nuchal shields: (*)**  *Brochu (1999, ch. 38 modified & revised in part); Andrade et al. (2011, ch. 470); Ristevski et al. (2018, ds 2, ch. 364); Smith et al. (in review, ds 1, ch. 370); Ősi et al. (in review, ds 1, ch. 430).*  *State (3), and the terminology 'cervical shield' is according to Marinho & Carvalho (2009).* *See also McAliley et al. (2006) for discussion on eusuchians.*  *This character is not* *applicable for taxa that lack dorsal osteoderms, or that lack a distinct nuchal shield (i.e. thalattosuchians).*  0. four paramedian nuchal shields, sided by two accessory shields, all enlarged relative to the remaining neck dermal armour  1. four paramedian nuchal shields enlarged relative to remaining neck shields, and no accessory shield enlarged  2. eight (or more) shields, arranged in two paramedian rows, enlarged relative to remaining neck shields, with no accessory shield enlarged  3. ten or more median osteoderms, combined with several lateral osteoderms, composing a distinct cervical shield |
| 434 | **Nuchal armour, morphology of nuchal shields relative to the remaining trunk dermal armour: (*)**  *Brochu (1999, ch. 38 modified in part); Andrade et al. (2011, ch. 471); Ristevski et al. (2018, ds 2, ch. 365); Smith et al. (in review, ds 1, ch. 371); Ősi et al. (in review, ds 1, ch. 431).*  *State (1) occurs in* Armadilosuchus *and Susisuchidae + Eusuchia (with a reversal in gavialoids).*  *This character is not* *applicable for taxa that lack dorsal osteoderms.*  0. nuchal and dorsal trunk shields undifferentiated, morphology grading continuously  1. nuchal shields clearly differentiated from dorsal trunk shields by size and general  morphology (regardless of contact between nuchal and trunk series) |
| 435 | **Presacral dorsal armour, presence of an anterior process (= anterolateral process, = stylofoveal process) to articulate with the anterior adjacent osteoderm, in medial dorsal elements: (*)**  *Norell & Clark (1990, ch. 13 revised); Clark (1994, ch. 96 modified); Brochu (1999, ch. 40 revised); Ortega et al. (2000, ch. 113 revised); Andrade et al. (2011, ch. 477 modified); Young et al. (2011, ch. 184); Young et al. (2013a, ch. 198); Young et al. (2012, ch. 233 modified); Young (2014, ch. 244); Young et al. (2016, ds 2, ch. 286); Ristevski et al. (2018, ds 1, ch. 483; ds 2, ch. 366 modified); Smith et al. (in review, ds 1, ch. 372; ds 2, ch. 483); Ősi et al. (in review, ds 1, ch. 432).*  *Scores for a similar morphology as Nesbitt (2011, ch. 403).*  *Note that this process does not include the lateral processes seen in dyrosaurids, as they articulate with the accessory osteoderms.*  *State (2) is a putative apomorphy of* Magyarosuchus fitosi*.*  *Here we modified this character by adding state (2), which is a modification of the distinct ‘peg-like’ anterolateral process seen in* Magyarosuchus fitosi*.*  *This character is not* *applicable for taxa that lack dorsal osteoderms.*  0. absent  1. present, as a distinct ‘peg-like’ process  2. present, but as an indistinct process, no longer being distinctly ‘peg-like’, as their lateral margin is contiguous with that of the osteoderm ventrolateral surface |
| 436 | **Presacral dorsal armour, surface of only the paravertebral osteoderms: (*)**  *Andrade et al. (2011, ch. 476); Nesbitt (2011, ch. 404); Young et al. (2012, ch. 235); Young (2014, ch. 246); Young et al. (2016, ds 2, ch. 287); Ristevski et al. (2018, ds 2, ch. 367); Smith et al. (in review, ds 1, ch. 373); Ősi et al. (in review, ds 1, ch. 433).*  *Crocodile-line archosaurs including, basal crocodylomorphs, have state (1).*  *In Thalattosuchia* Teleosaurus *and* Platysuchus *also have state (1).*  *This character is not* *applicable for taxa that lack dorsal osteoderms.*  0. either weakly arched or mostly straight, forming a flat osteoderm, either keeled or not  1. osteoderm either strongly curved, with convex surface, partially embracing the vertebrae from side to side, or the curvature is restricted to a distinct bend near the lateral edge |
| 437 | **Presacral dorsal armour, biserial or tetraserial dorsal shield: (*)**  *Young & Andrade (2009, ch. 147 part); Young et al. (2011, ch. 147 part); Young et al. (2013a, ch. 197 part); Young et al. (2012, ch. 232 part); Young (2014, ch. 243 part); Young et al. (2016, ds 2, ch. 289); Ristevski et al. (2018, ds 2, ch. 368); Smith et al. (in review, ds 1, ch. 374); Ősi et al. (in review, ds 1, ch. 434).*  *Susisuchidae + Eusuchia have state (1).*  *This character is not* *applicable for taxa that lack dorsal osteoderms.*  0. biserial dorsal shield (one pair of paramedian osteoderms per row)  1. tetraserial dorsal shield (two pairs of paramedian osterderms per row) |
| 438 | **Presacral dorsal armour, presence of accessory osteoderm columns that do not have a peg-like articulation with the paramedian column, and which are smaller in size than the paramedian column(s): (ORDERED) (*)**  *Ristevski et al. (2018, ds 2, ch. 369); Smith et al. (in review, ds 1, ch. 375); Ősi et al. (in review, ds 1, ch. 435).*  *This character is an amalgam of Andrade et al. (2011, ch. 472 + 473) and Young et al. (2016, ds 2, ch. 290).*  *Similar to the character in: Norell & Clark (1990, ch. 12 modified); Brochu (1999, ch. 37 modified); Ortega et al. (2000, ch. 107).*  *This character does not consider the accessory osteoderms of dyrosaurids to be homologous (see character relating to the ‘lateral process’).*  *This character does not consider the accessory osteoderms of notosuchians to be homologous, as their accessory osteoderms can retain the same size and shape as the paramedian column.*  *State (1) occurs in Bernissaartidae, Susisuchidae, and Eusuchia.*  *State (2) occurs in* Brachychampsa *and* Alligator mississippiensis.  *This character is not* *applicable for taxa that lack dorsal osteoderms.*  0. absent (either has: two paravertebral medial columns, the gobiosuchid, or notosuchian. or dyrosaurid morphology)  1. present, a lateral accessory column on either of the paramedian columns  2. present, two lateral accessory columns on either of the paramedian columns |
| 439 | **Presacral dorsal armour, presence of accessory osteoderm column that has a peg-like articulation with the paramedian column (through a ‘lateral process’ derived from the anterolateral margin of the paramedian osteoderms): (*)**  *Jouve et al. (2008, ch. 37 modified); Hastings et al. (2010, ch. 82 modified); Young et al. (2016, ds 2, ch. 291); Ristevski et al. (2018, ds 2, ch. 370); Smith et al. (in review, ds 1, ch. 376); Ősi et al. (in review, ds 1, ch. 436).*  *State (1) occurs in dyrosaurids.*  *This character was applied to test the homology of accessory osteoderms in dyrosaurids.*  *This character is not* *applicable for taxa that lack dorsal osteoderms.*  0. absent (either has: two paravertebral medial columns, the gobiosuchid or notosuchian or the advanced neosuchian morphology)  1. present, a lateral accessory column on either side of the paramedian columns, with articulations |
| 440 | **Presacral dorsal armour, presence of accessory osteoderm columns, anteriorly two lateral accessory columns which increase to four accessory columns in the trunk region: (*)**  *Ristevski et al. (2018, ds 1, ch. 477); Smith et al. (in review, ds 2, ch. 477); Ősi et al. (in review, ds 1, ch. 437).*  *State (1) occurs in* Gobiosuchus*.*  *This character is not* *applicable for taxa that lack dorsal osteoderms.*  0. absent (either has: two paravertebral medial columns, only two accessory columns, or the notosuchian morphology)  1. present |
| 441 | **Presacral dorsal armour, dimensions of the thoracic osteoderms: (*)**  *Clark (1994, ch. 95 modified); Nesbitt (2011, ch. 407); Young et al. (2012, ch. 234); Young (2014, ch. 245); Young et al. (2016, ds 2, ch. 292); Wilberg (2017, ch. 395 part); Ristevski et al. (2018, ds 2, ch. 371); Smith et al. (in review, ds 1, ch. 377); Ősi et al. (in review, ds 1, ch. 438).*  *Crocodile-line archosaurs, including basal crocodylomorphs, have state (1).*  *In Thalattosuchia, cervical osteoderms can be either state (0) or (1), so Young et al. (2016, ds 2) altered this character not to include the cervical osteoderms.*  *Crocodyliformes have state (2).*  *This character is not* *applicable for taxa that lack dorsal osteoderms.*  0. square shaped, length and width approximately equal  1. longer than wide  2. wider than long |
| 442 | **Presacral dorsal armour, transverse elongation of the thoracic osteoderms: (*)**  *Wilberg (2017, ch. 395 part); Ristevski et al. (2018, ds 2, ch. 372); Smith et al. (in review, ds 1, ch. 378); Ősi et al. (in review, ds 1, ch. 439).*  *State (1) occurs in goniopholidids and pholidosaurids (reversal in dyrosaurids).*  *This character can only be scored for those osteoderms that overlay the thoracic vertebrae, and come from the middle region of the trunk.*  *This character is not* *applicable for taxa that lack dorsal osteoderms.*  0. transverse width of these osteoderms is either small or sub-equal to the anteroposterior length, or only slightly wider  1. considerably wider than long, such that the transverse width is approximately three times the anteroposterior length |
| 443 | **Presacral dorsal armour, type of contact between elements in a row: (*)**  *Clark (1994, ch. 98); Andrade et al. (2011, ch. 474); Ristevski et al. (2018, ds 2, ch. 373); Smith et al. (in review, ds 1, ch. 379); Ősi et al. (in review, ds 1, ch. 440).*  *State (1) occurs in crown-group Crocodylia.*  *This character is not* *applicable for taxa that lack dorsal osteoderms.*  0. imbricated, any given anterior trunk osteoderm partially overlays its following element  1. sutured, osteoderms do not cover adjacent dermal elements, and are sutured if in contact |
| 444 | **Presacral dorsal armour, presence of an anteroposteriorly directed keel on the dorsal surface of paramedial elements: (*)**  *Buscalioni et al. (1992, ch. 22); Clark (1994, ch. 101 revised, part); Brochu (1999, ch. 35); Andrade et al. (2011, ch. 478); Young et al. (2012, ch. 240 modified); Young (2014, ch. 251 modified); Young et al. (2016, ds 2, ch. 298 modified); Ristevski et al. (2018, ds 2, ch. 378 modified); Smith et al. (in review, ds 1, ch. 384); Ősi et al. (in review, ds 1, ch. 441).*  *State (0) occurs in* Pelagosaurus.  *In Thalattosuchia the cervical and anterior dorsal osteoderms can have reduced keels, which can make it look as though they are absent. However, in* Pelagosaurus*, the anterior dorsal osteoderms lack keels, while the mid dorsal osteoderms are very poorly keeled (hard to discern from the interpit laminae). In Thalattosuchia the sacral and anterior-mid caudal osteoderms have raised keels, which along with the ventral caudal osteoderms are the most readily identifiable.*  *This character is not* *applicable for taxa that lack dorsal osteoderms.*  0. absent on approximately half to all of the paravertebral osteoderms, or if present in the anterior half of the presacral dorsal armour hard to discern from the interpit laminae  1. present along more than half, to all, of the paravertebral osteoderms |
| 445 | **Presacral ventral armour, presence of ventral collar scales: (*)**  *Poe (1997); Brochu (1999, ch. 156); Andrade et al. (2011, ch. 479); Ristevski et al. (2018, ds 2, ch. 379); Smith et al. (in review, ds 1, ch. 385); Ősi et al. (in review, ds 1, ch. 442).*  *This character is not* *applicable for taxa that lack osteoderms.*  0. absent, no shield enlarged relative to other ventral scales  1. present, forming a single row of enlarged scales  2. present, forming two parallel rows of enlarged scales |
| 446 | **Presacral ventral armour, presence of paired ossifications:**  *Buscalioni et al. (1992, ch. 21); Brochu (1999, ch. 39); Andrade et al. (2011, ch. 480); Ristevski et al. (2018, ds 2, ch. 380); Smith et al. (in review, ds 1, ch. 386); Ősi et al. (in review, ds 1, ch. 443).*  0. single or absent  1. present, pairs sutured together |
| 447 | **Postsacral (= caudal) armour, distribution of dorsal tail osteoderms:**  *Clark (1994, ch. 99 modified); Young (2006, ch. 49 part); Wilkinson et al. (2008, ch. 78 part); Young & Andrade (2009, ch. 78 part); Young et al. (2011, ch. 78 part); Young et al. (2013a, ch. 200 part); Young et al. (2012, ch. 237 modified); Young (2014, ch. 248 modified); Young et al. (2016, ds 2, ch. 295 modified); Ristevski et al. (2018, ds 2, ch. 375 modified); Smith et al. (in review, ds 1, ch. 381 modified); Ősi et al. (in review, ds 1, ch. 444).*  *Young et al. (2012) split the dorsal and ventral tail osteoderm character as* Pelagosaurus *and* Pietraroiasuchus *lack ventral tail osteoderms, but have dorsal tail osteoderms.*  0. present  1. absent |
| 448 | **Postsacral (= caudal) armour, distribution of ventral tail osteoderms:**  *Young (2006, ch. 49 part); Wilkinson et al. (2008, ch. 78 part); Young & Andrade (2009, ch. 78 part); Young et al. (2011, ch. 78 part); Young et al. (2013a, ch. 200 part); Young et al. (2012, ch. 238); Young (2014, ch. 249); Young et al. (2016, ds 2, ch. 296); Ristevski et al. (2018, ds 2, ch. 376); Smith et al. (in review, ds 1, ch. 382); Ősi et al. (in review, ds 1, ch. 445).*  *State (1) is a putative apomorphy of* Pelagosaurus *+ Metriorhynchidae, and occurs in* Pietraroiasuchus.  0. present  1. absent |
| 449 | **Postsacral (= caudal) armour, distribution when present: (*)**  *Clark (1994, ch. 99 modified); Young (2006, ch. 49 part); Wilkinson et al. (2008, ch. 78 part); Young & Andrade (2009, ch. 78 part); Andrade et al. (2011, ch. 481); Ristevski et al. (2018, ds 2, ch. 377); Smith et al. (in review, ds 1, ch. 383); Ősi et al. (in review, ds 1, ch. 446).*  *This character is not* *applicable for taxa that lack caudal osteoderms.*  0. a pair of rows, covering the vertebral column  1. several rows, enclosing the tail surface |
| 450 | **Postsacral (= caudal) armour, presence of an anteroposteriorly directed keel on the dorsal surface of paramedial elements: (*)**  *Clark (1994, ch. 101 revised part); Andrade et al. (2011, ch. 482); Ristevski et al. (2018, ds 2, ch. 381); Smith et al. (in review, ds 1, ch. 387); Ősi et al. (in review, ds 1, ch. 447).*  *This character is not* *applicable for taxa that lack dorsal osteoderms.*  0. absent  1. present |
| 451 | **Appendicular armour, presence of osteoderms on the limbs (at least in part):**  *Pol & Norell (2004b, ch. 190); Nesbitt (2011, ch. 405); Young et al. (2016, ds 2, ch. 288); Wilberg (2017, ch. 406); Ristevski et al. (2018, ds 2, ch. 382); Smith et al. (in review, ds 1, ch. 388); Ősi et al. (in review, ds 1, ch. 448).*  *Crocodyliformes have state (1), but perhaps with reversals in some clades.*  *Limb osteoderms are rarely preserved, but have been mentioned for some dyrosaurids and advanced neosuchians.*  0. absent  1. present |

**Dermal ossifications: gastralia** (Ch. 450; 0.22% of characters)

| # | Description |
| --- | --- |
| 452 | **Gastralia:**  *Nesbitt (2011, ch. 412); Ristevski et al. (2018, ds 2, ch. 383); Smith et al. (in review, ds 1, ch. 389); Ősi et al. (in review, ds 1, ch. 449).*  *State (0) occurs in Postosuchus, ‘sphenosuchians’, and* Protosuchus*.*  *State (1) occurs in crocodyliforms more derived than* Protosuchus*.*  *State (2) occurs in* Simosuchus.  0. forming extensive ventral basket with closely packed elements  1. well-separated  2. absent |

**Soft tissue** (Ch. 451 – 454; 0.881% of characters)

*[Herein soft tissue characters are only scorable for extant taxa]*

| # | Description |
| --- | --- |
| 453 | **Iris colour: (*)**  *Brochu & Storrs (2012, ch. 182); Narváez et al. (2015, ch. 182); Ősi et al. (in review, ds 1, ch. 450).*  *State (0) occurs in* Mecistops, Crocodylus, Caiman, Melanosuchus, Gavialis *and* Alligator mississippiensis.  *State (1) occurs in* Osteolameus, Tomistoma, Paleosuchus *and* Alligator sinensis.  *This character cannot be scored for fossil taxa.*  *All data from Brochu & Storrs (2012) and Narváez et al. (2015).*  0. greenish/yellowish  1. brown |
| 454 | **Tongue, presence of keratinised surface: (*)**  *Brochu (1999, ch. 159); Andrade et al. (2011, ch. 483); Ristevski et al. (2018, ds 2, ch. 384); Smith et al. (in review, ds 1, ch. 390); Ősi et al. (in review, ds 1, ch. 451).*  *State (1) is a putative apomorphy of Alligatoridae/Alligatoroidea.*  *This character cannot be scored for fossil taxa.*  *Originally based on Taplin & Grigg (1989), apud Brochu (1999).*  0. absent  1. presence |
| 455 | **Functional lingual salt glands, presence: (*)**  *based on Taplin (1985); Taplin & Grigg (1989); Brochu (2007); Andrade et al. (2011, ch. 484); Ristevski et al. (2018, ds 2, ch. 385); Smith et al. (in review, ds 1, ch. 391); Ősi et al. (in review, ds 1, ch. 452).*  *State (0) is a putative apomorphy of Alligatoridae.*  *This character cannot be scored for fossil taxa.*  0. absent  1. present |
| 456 | ***M. caudofemoralis*, morphology: (*)**  *Frey et al. (1989); Brochu (1999, ch. 160); Andrade et al. (2011, ch. 486); Brochu & Storrs (2012, ch. 37); Narváez et al. (2015, ch. 37); Ristevski et al. (2018, ds 2, ch. 387); Smith et al. (in review, ds 1, ch. 393); Ősi et al. (in review, ds 1, ch. 454).*  *State (0) occurs in* Gavialis.  *State (1) is known for all other extant crocodylians.*  *This character cannot be scored for fossil taxa.*  0. with single head  1. with double head (*longus* and *brevis*) |

**S3) Character and OTUs breakdowns of the merged, and parent, datasets**

***Table (S3.1).*** Character break-down from the iterations of the Hastings dataset, ultimately merged into the Hastings and Young (H+Y) matrix. Hastings *et al*. (2015) utilised two datasets: 1) Hastings *et al*. (2010, 2011); and 2) adapted from Jouve *et al*. (2006). Young *et al*. (2016) utilised two datasets: 1) first iteration of a merged dataset, an updated version of the matrix of Hastings *et al*. (2015) with characters used by Young; and 2) an updated version of Young (2014) matrix.

| Type of characters | Hastings *et al*. (2010, 2011, 2015, ds 1-Hastings) | Hastings *et al*. (2015, ds 2-Jouve) | Young *et*  *al*. (2016, ds 1-Hastings) |
| --- | --- | --- | --- |
| Skull geometry & dimensions | 1 | 3 | 1 |
| Craniomandibular ornamentation | 2 | 3 | 2 |
| Cranial rostrum | 17 | 32 | 19 |
| Skull roof | 11 | 24 | 21 |
| Orbit & temporal region | 7 | 30 | 7 |
| Palate & perichoanal structures | 4 | 27 | 4 |
| Occipital | 5 | 9 | 5 |
| Braincase, basicranium & suspensorium | 8 | 28 | 14 |
| Mandibular geometry | - | 2 | - |
| Mandible | 9 | 22 | 9 |
| Dental & alveolar | 17 | 20 | 22 |
| Vertebrae & ribs | - | 6 | 5 |
| Pectoral girdle & forelimbs | - | 11 | 2 |
| Pelvic girdle & hind limbs | - | 7 | 1 |
| Osteoderms | 1 | 10 | 8 |
|  |  |  |  |
| **Total character number** | **82** | **234** | **120** |
|  |  |  |  |
| ***Total dental+craniomandibular*** | **81** | **200** | **104** |
| ***Total post-cranial*** | **1** | **34** | **16** |
|  |  |  |  |
| ***Dental+craniomandibular osteology %*** | ***98.8*** | ***85.47*** | ***86.667*** |
| ***Post-cranial osteology%*** | ***1.2*** | ***14.53*** | ***13.333*** |

***Table (S3.2)***. Character break-down from the major different iterations of the Young dataset, ultimately merged into the Hastings and Young (H+Y) matrix. Young *et al*. (2016) utilised two datasets: 1) first iteration of a merged dataset, an updated version of the matrix of Hastings *et al*. (2015) with characters used by Young; and 2) an updated version of Young (2014) matrix. ***** note, the analysis for Young *et al*. (2013a) is actually a precursor to the Young *et al*. (2012) paper, which ended up being published first.

| Type of characters | Young  (2006) | Wilkinson  *et al*. (2008) | Young (2009) / Young &  Andrade (2009) | Young *et al*. (2011) | Young *et al*. (2013a) ***** | Young *et*  *al*. (2012) | Young *et al*. (2013b) / Young (2014) | Young *et*  *al*. (2016, ds2-Young) |
| --- | --- | --- | --- | --- | --- | --- | --- | --- |
| Skull geometry & dimensions | 1 | 1 | 1 | 1 | 1 | 3 | 3 | 5 |
| Craniomandibular ornamentation | 1 | 1 | 2 | 2 | 2 | 2 | 2 | 2 |
| Craniomandibular pneumaticity | - | - | 2 | 2 | 2 | 3 | 3 | 3 |
| Rostral neurovascular foramina | - | - | - | - | - | - | - | 1 |
| Cranial rostrum | 9 | 11 | 21 | 22 | 25 | 29 | 31 | 35 |
| Skull roof | 5 | 14 | 33 | 33 | 31 | 34 | 34 | 41 |
| Orbit & temporal region | 4 | 7 | 16 | 16 | 15 | 19 | 19 | 22 |
| Palate & perichoanal structures | - | 3 | 7 | 10 | 9 | 13 | 14 | 15 |
| Occipital | - | 3 | 6 | 7 | 8 | 8 | 8 | 9 |
| Braincase, basicranium & suspensorium | - | 2 | 10 | 10 | 13 | 14 | 15 | 17 |
| Mandibular geometry | - | - | - | 2 | 2 | 2 | 2 | 2 |
| Mandible | 6 | 9 | 16 | 18 | 18 | 22 | 22 | 26 |
| Dental & alveolar | 7 | 9 | 13 | 20 | 20 | 26 | 30 | 43 |
| Vertebrae & ribs | 6 | 6 | 15 | 17 | 18 | 22 | 23 | 24 |
| Pectoral girdle & forelimbs | 5 | 6 | 9 | 13 | 14 | 16 | 16 | 18 |
| Pelvic girdle & hind limbs | 7 | 7 | 11 | 11 | 16 | 18 | 20 | 21 |
| Osteoderms | 3 | 3 | 4 | 6 | 6 | 9 | 9 | 14 |
|  |  |  |  |  |  |  |  |  |
| **Total character number** | **54** | **82** | **166** | **190** | **201** | **240** | **251** | **298** |
|  |  |  |  |  |  |  |  |  |
| ***Total dental+craniomandibular*** | **33** | **60** | **127** | **143** | **147** | **175** | **183** | **221** |
| ***Total post-cranial*** | **21** | **22** | **39** | **47** | **54** | **65** | **68** | **77** |
|  |  |  |  |  |  |  |  |  |
| ***Dental+craniomandibular osteology %*** | ***61.111*** | ***73.171*** | ***76.506*** | ***75.263*** | ***73.134*** | ***72.917*** | ***72.908*** | ***74.161*** |
| ***Post-cranial osteology%*** | ***38.889*** | ***26.829*** | ***23.494*** | ***24.737*** | ***26.866*** | ***27.083*** | ***27.092*** | ***25.839*** |

***Table (S3.3).*** Character break-down from the different iterations of the merged Hastings + Young matrix.

| Type of characters | Ristevski *et al*. (2018) | Smith *et al*. (in review) | Ősi *et al*. (in review) / current |
| --- | --- | --- | --- |
| Skull geometry & dimensions | 6 | 7 | 10 |
| Craniomandibular ornamentation | 4 | 4 | 6 |
| Internal neuroanatomy & sensory systems | 1 | 1 | 1 |
| Craniomandibular pneumaticity | 4 | 4 | 4 |
| Rostral neurovascular foramina | 2 | 2 | 6 |
| Cranial rostrum | 53 | 53 | 58 |
| Skull roof | 50 | 51 | 52 |
| Orbit & temporal region | 27 | 29 | 29 |
| Palate & perichoanal structures | 19 | 19 | 22 |
| Occipital | 13 | 13 | 15 |
| Braincase, basicranium & suspensorium | 26 | 26 | 26 |
| Mandibular geometry | 4 | 4 | 8 |
| Mandible | 28 | 29 | 32 |
| Dental & alveolar | 52 | 52 | 65 |
| Vertebrae & ribs | 26 | 26 | 31 |
| Pectoral girdle & forelimbs | 17 | 17 | 23 |
| Pelvic girdle & hind limbs | 28 | 29 | 37 |
| Osteoderms | 23 | 23 | 24 |
| Gastralia | 1 | 1 | 1 |
| Soft tissue | 3 | 3 | 4 |
|  |  |  |  |
| **Total character number** | **387** | ***393*** | **454** |
|  |  |  |  |
| ***Total dental+craniomandibular*** | ***289*** | ***294*** | ***334*** |
| ***Total post-cranial*** | ***95*** | ***96*** | ***116*** |
| ***Total soft tissue*** | ***3*** | ***3*** | ***4*** |
|  |  |  |  |
| ***Dental+craniomandibular osteology %*** | ***74.677*** | ***74.809*** | ***73.568*** |
| ***Post-cranial osteology%*** | ***24.548*** | ***24.427*** | ***25.551*** |
| ***Soft tissue %*** | ***0.763*** | ***0.763*** | ***0.881*** |

***Table (S3.4)*** Break-down of the OTUs per clade from iterations of the Hastings dataset, ultimately merged into the Hastings and Young (H+Y) matrix. Hastings *et al*. (2015) utilised two datasets: 1) matrix of Hastings *et al*. (2010, 2011); and 2) adapted from Jouve *et al*. (2006). Young *et al*. (2016) utilised two datasets: 1) first iteration of a merged dataset, an updated version of the matrix of Hastings *et al*. (2015) with characters used by Young; and 2) an updated version of Young (2014) matrix. Note, the taxonomic break-down is based on the current topology, which for some OTUs will differ from the position they had in earlier less complete analyses.

| Clades of OTUs | Hastings *et al*. (2010) | Hastings *et al*. (2011) | Hastings *et al*. (2015, ds 1-Hastings) | Hastings *et al*. (2015, ds 2-Jouve) | Young *et*  *al*. (2016, ds 1-Hastings) |
| --- | --- | --- | --- | --- | --- |
| Non-crocodylomorph outgroup | - | - | - | - | 1 |
| ‘Sphenosuchia’ s. l. | - | - | - | 2 | 2 |
| Basal crocodyliforms | - | - | - | 7 | 1 |
| Notosuchia s. l. | - | - | - | 15 | - |
| Teleosauroidea | - | - | - | 1 | 1 |
| Basal metriorhynchoids | - | - | - | 1 | 1 |
| Basal metriorhynchines | - | - | - | 1 | 1 |
| Indet. Neosuchia | - | - | - | 1 | - |
| Atoposauridae | - | - | - | 1 | - |
| Bernissartiidae | - | - | - | 1 | - |
| Paralligatoridae | - | - | - | 2 | - |
| Hylaeochampsidae | - | - | - | 1 | - |
| Crown-Crocodylia | - | - | - | 3 | 2 |
| Goniopholididae | - | - | - | 4 | 2 |
| Pholidosauridae | 3 | 3 | 3 | 5 | 8 |
| Basal to dyrosaurids | - | - | - | - | 3 |
| Dyrosauridae | 13 | 14 | 15 | 4 | 15 |
|  |  |  |  |  |  |
| **Total number of OTUs** | **16** | **17** | **18** | **49** | **37** |
|  |  |  |  |  |  |
| **Total character number** | **82** | **82** | **82** | **234** | **120** |
|  |  |  |  |  |  |
| ***OTU # / Characters #*** | ***5.125 : 1*** | ***4.824 : 1*** | ***4.556 : 1*** | ***4.776 : 1*** | ***3.243 : 1*** |

***Table (S3.5).*** Break-down of the OTUs per clade from the major different iterations of the Young dataset, ultimately merged into the Hastings and Young (H+Y) matrix. Young *et al*. (2016) utilised two datasets: 1) first iteration of a merged dataset, an updated version of the matrix of Hastings *et al*. (2015) with characters used by Young; and 2) an updated version of Young (2014) matrix. Note, the taxonomic break-down is based on the current topology, which for some OTUs will differ from the position they had in earlier less complete analyses. ***** note, the analysis for Young *et al*. (2013a) is actually a precursor to the Young *et al*. (2012) paper, which ended up being published first.

| Clades of OTUs | Young  (2006) | Wilkinson  *et al*. (2008) | Young (2009) / Young &  Andrade (2009) | Young *et al*. (2011) | Young *et al*. (2013a) ***** | Young *et*  *al*. (2012) | Young *et al*. (2013) / Young (2014) | Young *et*  *al*. (2016, ds2-Young) |
| --- | --- | --- | --- | --- | --- | --- | --- | --- |
| Non-crocodylomorph outgroup | - | - | 1 | 1 | 1 | 1 | 1 | 1 |
| ‘Sphenosuchia’ s. l. | - | 2 | 3 | 1 | 1 | 3 | 3 | 4 |
| Basal crocodyliforms | 1 | 1 | 1 | 1 | 1 | 1 | 1 | 1 |
| Notosuchia s. l. | - | - | 11 | - | - | 11 | 11 | 12 |
| Atoposauridae | 1 | 1 | 2 | - | - | 2 | 2 | 2 |
| Goniopholididae | 1 | 1 | 5 | 3 | 3 | 4 | 4 | 5 |
| Susisuchidae | - | - | 2 | 1 | 1 | 2 | 2 | 2 |
| Hylaeochampsidae | - | - | - | - | - | - | - | 2 |
| Crown-Crocodylia | - | 2 | 4 | 3 | 3 | 3 | 3 | 4 |
| Pholidosauridae | - | - | 6 | 1 | 1 | 1 | 1 | 7 |
| Basal to dyrosaurids | - | - | - | - | - | - | - | 3 |
| Dyrosauridae | - | - | 7 | - | - | - | - | 8 |
| Teleosauroids | 1 | 1 | 4 | 1 | 1 | 9 | 9 | 12 |
| Basal metriorhynchoids | 2 | 2 | 6 | 6 | 6 | 6 | 6 | 6 |
| Basal metriorhynchines | 4 | 5 | 6 | 5 | 5 | 5 | 5 | 3 |
| Rhacheosaurini | 4 | 5 | 12 | 11 | 11 | 11 | 11 | 13 |
| Basal geosaurines | 3 | 3 | 5 | 5 | 5 | 5 | 5 | 5 |
| Geosaurini | 4 | 5 | 11 | 11 | 12 | 9 | 11 | 14 |
|  |  |  |  |  |  |  |  |  |
| **Total number of OTUs** | **21** | **28** | **86** | **50** | **51** | **73** | **75** | **104** |
|  |  |  |  |  |  |  |  |  |
| **Total character number** | **54** | **82** | **166** | **190** | **201** | **240** | **251** | **298** |
|  |  |  |  |  |  |  |  |  |
| ***OTU # / Characters #*** | ***2.571 : 1*** | ***2.929 : 1*** | ***1.930 : 1*** | ***3.800 : 1*** | ***3.941 : 1*** | ***3.288 : 1*** | ***3.467 : 1*** | ***2.865 : 1*** |

***Table (S3.6).*** Break-down of the OTUs per clade from the different iterations of the merged Hastings + Young matrix. Note, the taxonomic break-down is based on the current topology, which for some OTUs will differ from the position they had in earlier less complete analyses.

| Clades of OTUs | Ristevski *et al*. (2018) | Smith *et al*. (in review) | Ősi *et al*. (in review) | Current |
| --- | --- | --- | --- | --- |
| Non-crocodylomorph outgroup | 1 | 1 | 1 | 1 |
| ‘Sphenosuchia’ s. l. | 5 | 5 | 5 | 5 |
| Basal crocodyliforms | 5 | 5 | 5 | 5 |
| Notosuchia s. l. | 12 | 12 | 12 | 12 |
| Atoposauridae | 2 | 2 | 2 | 2 |
| Goniopholididae | 8 | 6 | 7 | 7 |
| Bernissartiidae | 2 | 2 | 2 | 2 |
| Susisuchidae | 2 | 2 | 2 | 2 |
| Hylaeochampsidae | 2 | 2 | 2 | 2 |
| Crown-Crocodylia | 4 | 4 | 4 | 4 |
| Pholidosauridae | 10 | 11 | 11 | 11 |
| Basal to dyrosaurids | 1 | 2 | 2 | 2 |
| Dyrosauridae | 16 | 17 | 17 | 17 |
| Teleosauroids | 18 | 18 | 18 | 20 |
| Basal metriorhynchoids | 7 | 7 | 8 | 8 |
| Basal metriorhynchines | 4 | 4 | 4 | 4 |
| Rhacheosaurini | 14 | 14 | 14 | 14 |
| Basal geosaurines | 5 | 5 | 5 | 5 |
| Geosaurini | 19 | 19 | 19 | 19 |
|  |  |  |  |  |
| **Total number of OTUs** | **137** | **138** | **140** | **142** |
|  |  |  |  |  |
| **Total character number** | **387** | **393** | **454** | **454** |
|  |  |  |  |  |
| ***OTU # / Characters #*** | ***2.825 : 1*** | ***2.848 : 1*** | ***3.243 : 1*** | ***3.197 : 1*** |

**S4) Supplementary references**

**Aguilera E, Salas H, Peña E. 1989.** La Formación Cajones: Cretácico terminal del subandino central de Bolivia. *Revista Técnica YPFB* **10**: 131–148.

**Allen ER.** **2012.** *Analysis of North American goniopholidid crocodyliforms in a phylogenetic context.* Unpublished MS (Master of Science) thesis. University of Iowa, 100 pp.

**Andrade MB. 2005.** *Revisão sistemática e taxonômica dos Notosuchia (Metasuchia, Crocodylomorpha)*. Unpublished MS (Master of Science) thesis. Universidade Estadual Paulista, Rio Claro, 239 pp.

**Andrade MB. 2009.** Solving a century-old mystery: the structure and function of the maxillary depressions of *Goniopholis* (Crocodylomorpha, Neosuchia). *Journal of Vertebrate* *Paleontology* **29** (3): 54A–55A.

**Andrade MB. 2010.** *The evolution of Gondwanan Mesoeucrocodylia (Crurotarsi, Crocodylomorpha) from Jurassic to Cretaceous*. Unpublished PhD thesis, University of Bristol, Bristol, 256 pp.

**Andrade MB, Bertini RJ. 2005.** Bibliographic revision of *Uruguaysuchus* (Mesoeucrocodylia: Crocodylomorpha): is *Uruguaysuchus terrai* a valid species?. Pp. 21–22 in Congresso Latino Americano de Paleontologia de Vertebrados, 2. *Boletim*, Rio de Janeiro, Museu Nacional/UFRJ.

**Andrade MB, Bertini RJ. 2008a.** A new *Sphagesaurus* (Mesoeucrocodylia: Notosuchia) from the Upper Cretaceous of Monte Alto City (Bauru Group, Brazil), and a revision of the Sphagesauridae. *Historical Biology* **20** (2): 101–136. DOI: 10.1080/08912960701642949.

**Andrade MB, Bertini RJ. 2008b.** Morphological and anatomical observations about *Mariliasuchus amarali* and *Notosuchus terrestris* (Mesoeucrocodylia), and their phylogenetical relationships with other South American notosuchians. *Arquivos do Museu Nacional* **66** (1): 5–62.

**Andrade MB, Bertini RJ. 2008c.** Morphology of the dental carinae in *Mariliasuchus amarali* (Crocodylomorpha, Notosuchia) and the pattern of tooth serration among basal Mesoeucrocodylia. *Arquivos do Museu Nacional* **66** (1): 63–82.

**Andrade MB, Bertini RJ. 2008d.** The *Chimaerasuchus* paradox: critical revision of a poorly known fossil crocodylomorph. Pp. 13 in Calvo, JO, Valieri, RJ, Porfiri, JD, Santos, D. Congreso Latino-Americano de Paleontologia de Vertebrados, 3. *Actas*, Neuquén, Universidad Nacional del Comahue.

**Andrade MB, Edmonds R, Benton MJ, Schouten R.** **2011.** A new Berriasian species of *Goniopholis* (Mesoeucrocodylia, Neosuchia) from England, and a review of the genus. *Zoological Journal of the Linnean Society* **163**: 66–108.

**Andrade MB, Hornung JJ.** **2011.** A new look into the periorbital morphology of *Goniopholis* (Mesoeucrocodylia, Neosuchia) and related forms. *Journal of Vertebrate Paleontology* **31**: 352–368.

**Andrade MB, Young MT, Desojo JB, Brusatte SL. 2010.** The evolution of extreme hypercarnivory in Metriorhynchidae (Mesoeucrocodylia: Thalattosuchia) based on evidence from microscopic denticle morphology. *Journal of Vertebrate Paleontology* **30** (5): 1451–1465.

**Andrews CW. 1909.** XXXVIII. - On some new Steneosaurs from the Oxford Clay of Peterborough. *Annals and Magazine of Natural History* **3**: 299–308.

**Andrews CW. 1913.** *A descriptive catalogue of the marine reptiles of the Oxford Clay based on the Leeds Collection in the Brittish Museum (Natural History), London – Part II*. London: Taylor, Francis. 206 pp.

**Arruda JT, Carvalho IS, Vasconcellos FM. 2004.** Baurusuquídeos da Bacia Bauru (Cretáceo Superior, Brasil). *Anuário do Instituto de Geociências* **27**: 65–75.

**Averianov AO.** **2000.** *Sunosuchus* sp. (Crocodylomorpha, Goniopholididae) from the Middle Jurassic of Kirghisia. *Journal of Vertebrate* *Paleontology* **20**: 776–779.

**Avilla LS, Fernandes R, Ramos DFB.** **2004.** Bite marks on a crocodylomorph from the Upper Cretaceous of Brazil: evidence of social behavior? *Journal of Vertebrate Paleontology* **24**: 971–973.

**Barbosa JA, Kellner, AWA, Viana MSS. 2008.** New dyrosaurid crocodylomorph and evidences for faunal turnover at the K–P transition in Brazil. *Proceeding of the Royal Society B* **275** (1641): 1385–1391.

**Barrientos-Lara JI, Herrera Y, Fernández MS, Alvarado-Ortega, J. 2016.** Occurrence of *Torvoneustes* (Crocodylomorpha, Metriorhynchidae) in marine Jurassic deposits of Oaxaxa, Mexico. *Revista Brasileira de Paleontologia* **19** (3): 415–424.

**Benton MJ, Clark JM.** **1988.** Archosaur phylogeny and the relationships of the Crocodylia. Pp. 295–338 in Benton, M. J. (ed.) *The phylogeny and classification of the Tetrapods, Volume 1: Amphibians, Reptiles, Birds*. Clarendon Press, Oxford.

**Benton MJ, Walker AD. 2002.** *Erpetosuchus*, a crocodile-like basal archosaur from the Late Triassic of Elgin, Scotland. *Zoological Journal of the Linnean Society* **136**: 25–47.

**Berckhemer F. 1929.** Beiträge zur Kenntnis der Krokodilier des schwäbischen oberen Lias. *Neues Jahrbuch für Mineralogie* **64**:1–59.

**Bergounioux FM. 1955.** Les crocodiliens fossiles des dépôts phosphatés du sud-Tunisien, *Comptes Rendus de l’Academie des Sciences de Paris* **240**: 1917–1918.

**de Blainville HD. 1853.** Letter by H. D. de Blainville. In: Eudes-Deslongchamps JA, ed. Lettres sur les crocodiles vivants et fossiles. *Bulletin de la Société Linnéenne de Normandie* (Années 1849–1853) **IX**: 109–120.

**Blake JF. 1876.** Reptilia; pp. 243-254. In: R. Tate and J.F. Blake (eds.) *The Yorkshire Lias*. John van Voorst, London, 475 pp.

**Bonaparte JF. 1971.** Los tetrapodos del sector superior de la Formacion Los Colorados, La Rioja, Argentina. (Triásico Superior). *Opera Lilloana* **22**: 1–184.

**Bonaparte JF. 1991.** Los vertebrados fosiles de la Formación Rio Colorado, de la Ciudad de Neuquen y cercanias, Cretácico Superior, Argentina. *Revista del Museo Argentino de Ciências Naturales* **4** (3): 31–63.

**Bonaparte JF. 1996.** Cretaceous tetrapods of Argentina. *Münchner Geowissenschaften* **30**: 73–130.

**Brochu CA. 1997a.** Morphology, fossils, divergence timing, and the phylogenetic relationships of *Gavialis*. *Systematic Biolology* **46**: 479–522.

**Brochu CA. 1997b.** A review of ‘*Leidyosuchus’* (Crocodyliformes, Eusuchia) from the Cretaceous through Eocene of North America. *Journal of Vertebrate Paleontology* **17**: 679–697.

**Brochu CA. 1999.** Phylogenetics, taxonomy, and historical biogeography of Alligatoroidea. *Journal of Vertebrate Paleontology* – *Supplement (Society of Vertebrate Paleontology Memoir 6)*, **19**: 9–100.

**Brochu CA. 2001.** Crocodylian snouts in space and time: phylogenetic approaches toward adaptive radiation. *American Zoologist* **41**: 564–585.

**Brochu CA.** **2007.** Morphology, relationships, and biogeographical significance of an extinct horned crocodile (Crocodylia, Crocodylidae) from the Quaternary of Madagascar. *Zoological Journal of the Linnean Society* **150**: 835–863.

**Brochu CA, Bouare ML, Sissoko F, Roberts EM, O'Leary MA. 2002.** A dyrosaurid crocodyliform braincase from Mali. *Journal of Paleontology* **76**: 1060–1071.

**Brochu CA, Storrs GW. 2012.** A giant crocodile from the Plio-Pleistocene of Kenya, the phylogenetic relationships of Neogene African crocodylines, and the antiquity of *Crocodylus* in Africa. *Journal of Vertebrate Paleontology* **32** (3): 587–602.

**de Broin F, Taquet P. 1966.** Découverte d’un crocodilien nouveau dans le Crétacé inférieur du Sahara. *Comptes Rendus de l’Académie des Sciences de Paris* – *Ser. D*, **262** (22): 2326–2329.

**Bronn HG. 1841.** Untersuchung zweier Gavial-Skelette und der Gaumen zweier andern aus den *Boller* Lias-Schiefen, mit Rücksicht auf Geoffroy's genus *Teleosaurus*. pp. 5–30 In: H.G. Bronn and J.J. Kaup (eds). *Abhandlungen uber die Gavial-artigen Reptilien der Lias-Formation*. E. Schweizerbart'sche Verlagshandlung, Stuttgart, 47 pp.

**Buchy M-C, Vignaud P, Frey E, Stinnesbeck W, González AHG. 2006.** A new thalattosuchian crocodyliform from the Tithonian (Upper Jurassic) of northeastern Mexico. *Comptes Rendus Palevol* **5**: 785–794.

**Buchy M-C, Young MT, Andrade MB. 2013.** A new specimen of *Cricosaurus* *saltillensis* (Crocodylomorpha: Metriorhynchidae) from the Upper Jurassic of Mexico: evidence for craniofacial convergence within Metriorhynchidae. *Oryctos* **10**: 9–21.
[truncated: 73,388 more chars]
